# On the interactions of leflunomide and teriflunomide within receptor cavity — NMR studies and energy calculations

**DOI:** 10.1007/s00894-015-2643-z

**Published:** 2015-04-08

**Authors:** Jacek Kujawski, Marek K. Bernard, Elżbieta Jodłowska, Kornelia Czaja, Beata Drabińska

**Affiliations:** Department of Organic Chemistry, Faculty of Pharmacy, Poznan University of Medical Sciences, Grunwaldzka 6 street, 60-780, Poznań, Poland

**Keywords:** DFT calculations, Hydrogen bond, Leflunomide, NMR calculations, Teriflunomide

## Abstract

Leflunomide is a disease-modifying antirheumatic drug with antiinflammatory and immunosuppressive activity used for the treatment of psoriatic and rheumatoid arthritis. It undergoes rapid metabolization to teriflunomide, a metabolite that is responsible for the biological activity of leflunomide. Continuing our investigations on the interactions of biologically important azahetarenes with the environment, we focused on leflunomide and its active metabolite, teriflunomide, considering the interactions teriflunomide–amino acid within the target protein (dihydroorotate dehydrogenase) using density functional theory, as well as ONIOM techniques. The results of theoretical studies have shown that the interactions of teriflunomide with tyrosine and arginine involve principally the amide fragment of teriflunomide. The presence of the internal hydrogen bond between (*Z*)-teriflunomide carbonyl oxygen and enolic hydroxyl decreases the interaction strength between teriflunomide and tyrosine or arginine. Even the *E* isomer of teriflunomide would usually provide a stronger interaction teriflunomide—amino acid than the *Z* isomer with the internal hydrogen bond.

**Electronic supplementary material:**

The online version of this article (doi:10.1007/s00894-015-2643-z) contains supplementary material, which is available to authorized users.

**Graphical Abstract:**
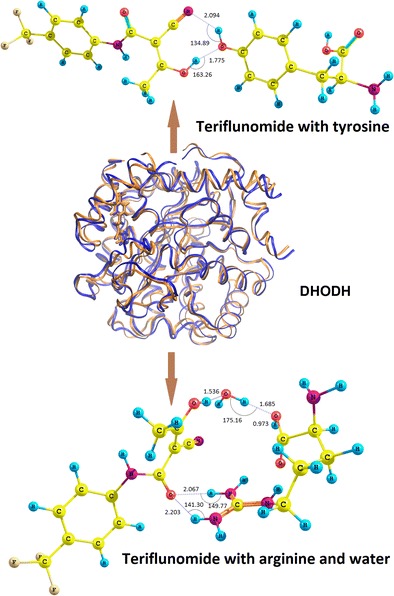
The interactions of leflunomide and teriflunomide within receptor cavityᅟ

## Introduction

**Scheme 1:**
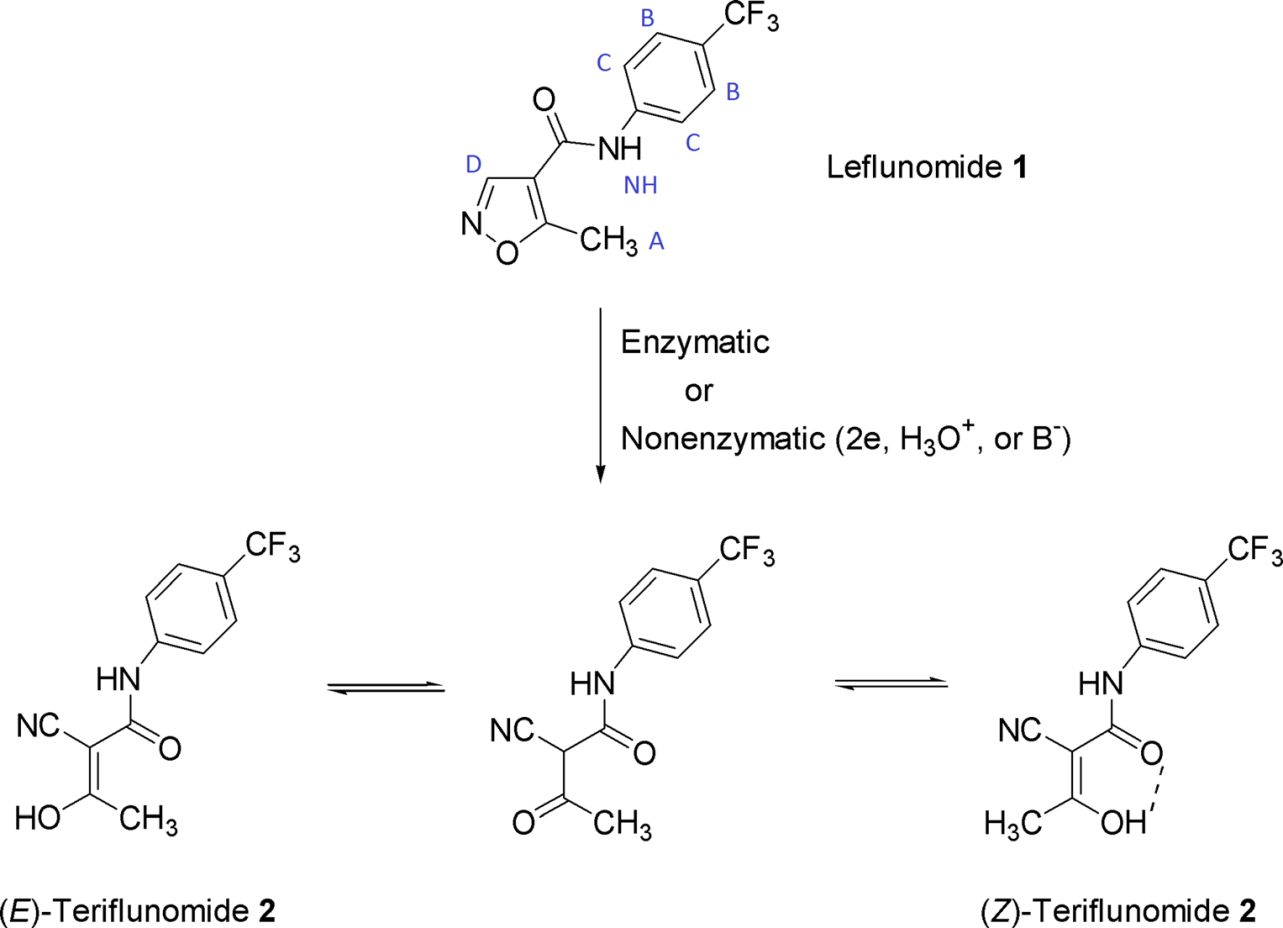
Mechanism of leflunomide **1** metabolizationᅟ

Leflunomide **1** (Scheme 1) is a disease-modifying antirheumatic drug (DMARD) with antiinflammatory and immunosuppressive activity used for the treatment of psoriatic and rheumatoid arthritis since 1998 (USA) and 1999 (EU) [1–3]. Leflunomide undergoes rapid metabolization to teriflunomide **2** (Scheme 1), a metabolite that is responsible for the biological activity of leflunomide [4]. Teriflunomide **2** is a noncompetitive inhibitor of dihydroorotate dehydrogenase (DHODH), an enzyme involved in the conversion of dihydroorotate (DHO) to orotate by utilizing a flavin mononucleotide (FMN) cofactor in the redox reaction present in the pyrimidine *de novo* biosynthesis pathway. This leads to inhibition of β-lymphocyte proliferation and immunomodulatory effect. Moreover, teriflunomide **2** suppresses T-cell proliferation by blocking the synthesis of immunosuppressive cytokines. Teriflunomide itself is used in the management of relapsing multiple sclerosis as an oral drug [5, 6]. Due to its interaction with the immune system, leflunomide **1** has also been investigated for anticancer activity. It was shown that leflunomide might be a potential new candidate for targeted therapy in multiple myeloma [7] and, more recently, in neuroblastoma [8]. The pharmacological profile of leflunomide **1** seems to be an inspirational factor that stimulates many scientific groups around the world for searching of new synthetic methods of this drug as well as its analogues [9–13].

Albeit the detailed mechanism of leflunomide **1** metabolization is not known, the experimental studies indicated that the presence of unsubstituted C-3 position in the isoxazole ring is crucial for the ring opening [4]. Most probably the ring cleavage occurs through a two-electron reduction to an imine intermediate. The imine is further converted via a P450-catalyzed dehydration to teriflunomide **2**. The latter compound can exist in two geometric forms, i.e., *Z* and *E* isomers, that can equilibriate through a keto form (Scheme 1). The *Z* isomer is energetically favored due to the presence of an internal hydrogen bond between the keto and hydroxyl group. The existence of this bond is also helpful in the penetration of teriflunomide through cell membranes but it is believed that such bond interferes with the interaction of teriflunomide with the target enzyme—DHODH.

The interaction of teriflunomide **2** with DHODH has been a subject of several investigations, both from the experimental and theoretical point of view. Liu et al. [14] analyzed the crystal structure of the teriflunomide–human DHODH complex and found that teriflunomide **2** interacts with amino acids Tyr356 and Arg136 in the enzyme domain. The carbonyl oxygen is hydrogen bonded through a water molecule to Arg136, whereas the enolic hydroxyl is directly linked to Tyr356. In a more recent mostly docking studies, Leban et al. as well as Davies et al. [15, 16] concluded that in the **2**–HSDHODH complex, three hydrogen bonds could be observed. Apart from the above direct bonding to Tyr365, there are two water-mediated hydrogen contacts to Arg265 and Gln47. From a comprehensive theoretical analysis of compounds **1** and **2** as well as several teriflunomide analogues, Panek et al. [17] inferred that the primary acceptors of the external interactions are the amide and nitrile groups.

The interactions between drug molecules and their environment can be investigated with a variety of analytical methods including NMR, IR, Raman, mass, and scanning tunneling spectroscopy (STS). Computational chemistry is an invaluable complement to nuclear magnetic resonance spectroscopy as it allows for rapid visualization of the solvation phenomena. We successfully applied the methodology that involve computations and NMR for the estimation of interaction sites of an indazole–magnesium complex [18]. These interactions are important because of the relationship between magnesium and oncogenesis [19]. Moreover, the ^1^H NMR technique, compared to other methods, is fast and cheap, and enables to follow changes in chemical shifts with no need for a time-consuming alternative approach. Herein it must be added that the use of ^15^N or ^17^O NMR techniques would result in serious errors and could not be such informative as there are only two nitrogen and oxygen atoms in the structure of **1** and **2**. Moreover, very low natural abundance and a relatively large quadrupole moment renders ^17^O NMR method difficult for routine NMR measurements. The first drawback is relevant to ^15^N NMR as well. This substantiates once more the assumption that the use of proton NMR spectroscopy is the most appropriate for the studies described in the present paper. However, the investigations and understanding of the relationship between molecular structure and NMR parameters can sometimes be quite difficult, and therefore are often supported by theoretical calculations [17, 18]. Applications of *in silico* techniques are very wide, e.g., the DFT calculations at the B3LYP/6-31G level of theory were recently used to study leflunomide adsorption to nanotubes [20]. The approach that involves computations and ^1^H NMR spectroscopy for the estimation of interaction sites of analyte able to affect the environment seems to be a right choice. On this account we decided to extend the goal of our study to investigate the NMR spectrum of the pro-drug **1**, especially the influence of solvent molecules on amide moiety within cell. Then, we focused our attention on the interactions of active metabolite, teriflunomide **2**, with selected amino acids in the enzymatic binding site of DHODH. To validate conclusions, we carried out an ONIOM analysis to confirm the results of the DFT investigation, especially that, to the authors’ knowledge, there have been no prior studies of teriflunomide and DHODH using the ONIOM technique.

## Results and discussion

Continuing our investigations on the interactions of biologically important azahetarenes with environment, we focused on leflunomide and its active metabolite, teriflunomide. The present study deals, *inter alia*, with simulation of the ^1^H NMR spectrum of **1**. Particular attention was given to the amide bond and isoxazole ring in the first solvation sphere. The obtained results were correlated with the experimental data of Faragher et al. [21]. Furthermore, we estimated the interaction energy of teriflunomide **2** with tyrosine and, through a water molecule, with arginine, both in the enzymatic binding site. To achieve the above aims, we performed a geometric analysis using *Gaussian G09 D.01* suite [22]. The conformers were obtained by rotating the bonds C9-N1, N1-C2, and C2-C11 (leflunomide **1**) or C12-N2, N2-C5, and C5-C3 (teriflunomide **2**) in dihedral angle increments of 20°. The conformers were further optimized in a solvation model — conductor-like polarizable continuum model (CPCM) — with water as a solvent at the a) DFT/B3LYP/6-31G(d,p) and b) DFT/B3LYP/6-311+G(d,p) level of theory [23–25].

Herein we have to add that the structural analysis of compounds **1** and **2** was carried out previously [17], but the authors examined only geometric arrangements of these compounds and their electronic properties. In this study our attention was focused only on the ^1^H NMR spectra of leflunomide **1** and the interactions of teriflunomide **2** as a ligand with selected amino acids in the enzymatic binding site.

The theoretical ^1^H NMR spectrum was generated for all rotamers using the gauge-including atomic orbital (GIAO) method [26], implemented in *Gaussian G09 D.01*. To show the results more clearly, only four rotamers of the lowest energy were considered, optimized at the B3LYP/6-31G(d,p) (conformers **I**–**IV**, Tables S1, supplementary material) and B3LYP/6-311+G(d,p) (conformers **V**–**VIII**, Table S2, respectively, supplementary material) level of theory (CPCM solvation model and water as solvent). The calculated values (Table S1 and S2) show a strong correlation with the NMR experimental data for compound **1** [21]. Only the amide proton has a high relative error of the chemical shift, equal to 29 % at the B3LYP/6-31G(d,p)/CPCM level of theory and to 28 % at the B3LYP/6-311+G(d,p)/CPCM level of theory. These errors may be due to the steric effects connected with the proximity of rotating methylisoxazole and phenyl groups, proton mobility and its ability to interact with the solvent. The use of more complex basis sets or diffuse functions during optimization does not increase the correlation between the calculated and experimental values of chemical shifts. Taking into consideration the accuracy of calculations as well as time and cost required to complete them, the use of DFT/B3LYP/6-31G(d,p)/GIAO in the NMR analysis of rotamers of **1** seems to be a reasonable and justified choice. It is worth mentioning that more time-consuming basis (B3LYP/6-311++G(3df,2pd)/CPCM) applied for this type of calculations did not result in a significant improvement of accuracy (Table S4, supplementary material).

**Fig. 1:**
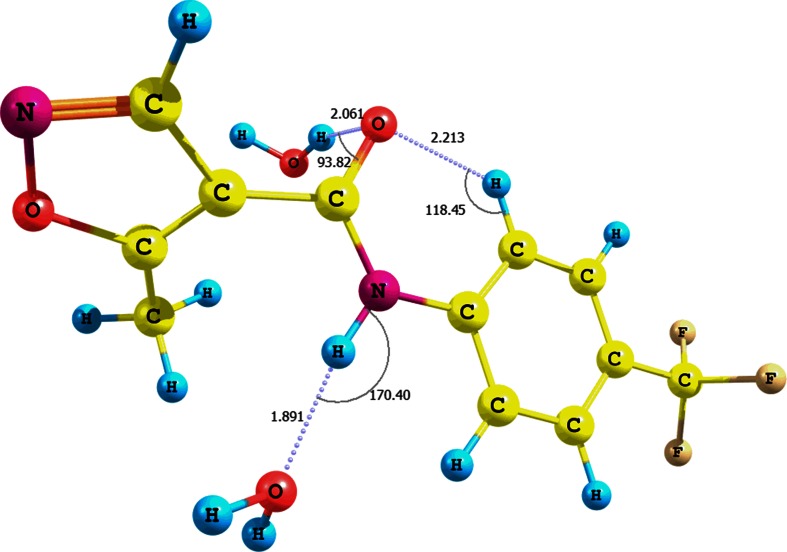
Interaction of **1** with water molecules and formation of NH^…^H_2_O hydrogen bond (conformer **IX**)

The influence of solvent on the relative error of the chemical shift of the amide proton is supported by a simulation of the NH^…^H_2_O interactions (conformer **IX**, Fig. 1, Table S3 in the supplementary material; CPCM solvation model and water as solvent). In this case the presence of water molecules causes a change of the N-H bond length before and after optimization at the B3LYP/6-31G(d,p)/CPCM level of theory by approximately 0.01 Å. It results in a considerable decrease of the chemical shift relative error of the amide proton to approximately 5 % and in the formation of an N-H^…^O strong hydrogen contact (r_N-H_ = 1.025 Å, d_H-O_ = 1.891 Å, θ =170.4°). It is noteworthy that leflunomide **1**–H_2_O adduct has a relatively high interaction energy, equal to -10.30 kcal mol^-1^ (including basis set superposition error, BSSE) [27, 28].

**Fig. 2:**
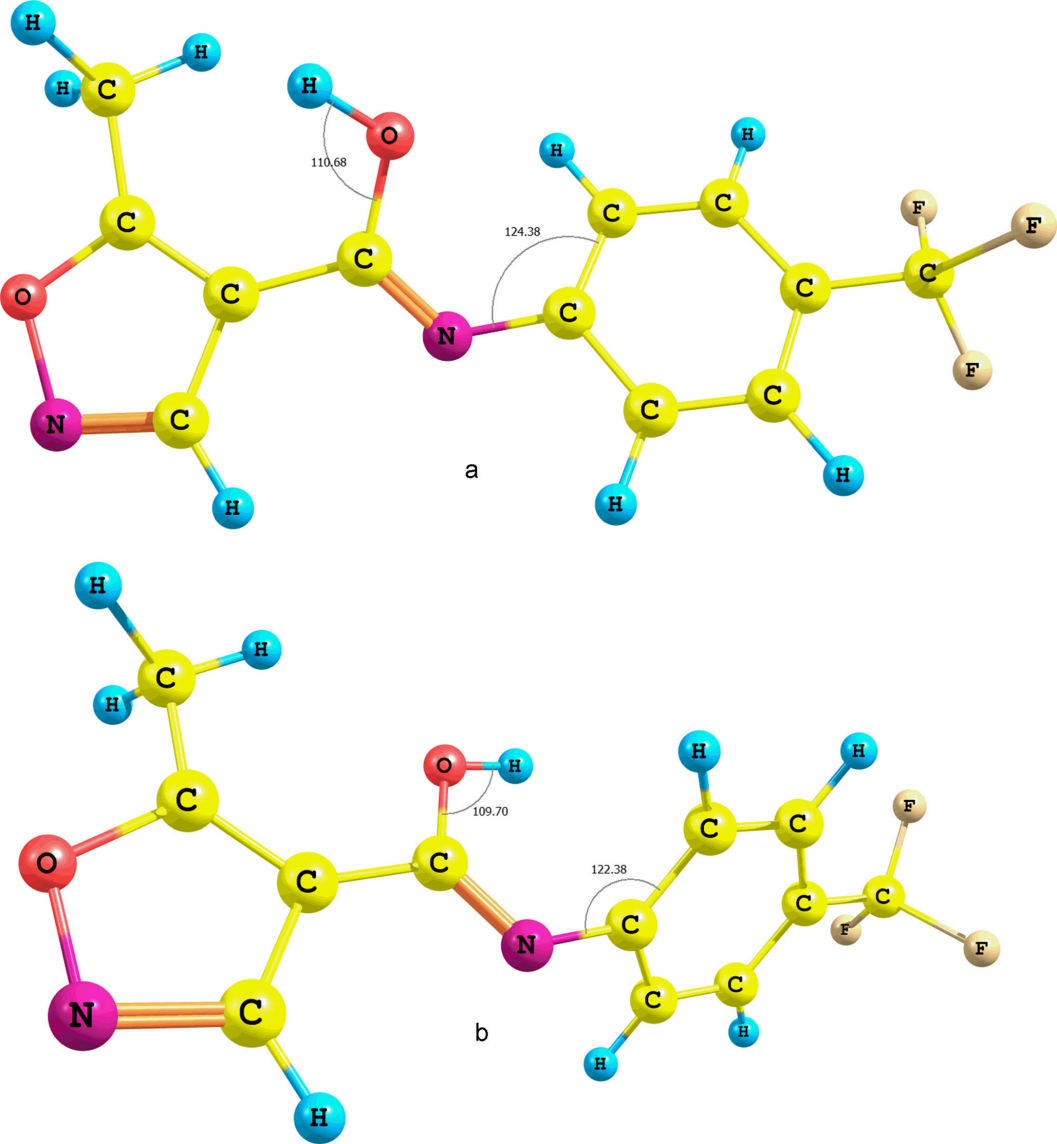
Tautomers of **1** with different positions (**a** and **b**) of hydroxyl groups

The mobility of the amide proton of leflunomide **1** could also be caused by keto-enol tautomerism and the formation of a N=C-OH bond. To check whether this is the case, the energy of the enol form of **1** was estimated and optimized at the B3LYP/6-31G(d,p) level of theory (CPCM solvation model and water as solvent) for two isomers with the hydroxyl hydrogen *anti* or *syn* to the C=N bond (Fig. 2a and b). The energy difference between the enol forms (a) and (b) of **1** is approximately 3.28 kcal mol^-1^. The more stable conformer is the one with the *anti* hydroxyl proton (Fig. 2a). Undoubtedly, this energy difference is partly due to a forced rotation of the phenyl ring of the second enol form (Fig. 2b). The energy difference between the amide form of **1** and its stable tautomer (Fig. 2a) is approximately 17.73 kcal mol^-1^ at the same level of theory. The influence of the trifluoromethyl group attached at the phenyl ring of **1** on tautomerism is negligible. This is because the energy difference between the enol form analogues lacking the CF_3_ group is approximately 3.74 kcal mol^-1^. On the other hand, the difference between the enol and amide analogues of **1** without the CF_3_ group is approximately 18.8 kcal mol^-1^. These results indicate that the secondary amide bond of leflunomide **1** is by far a more stable one, despite its susceptibility to a polar solvent that can impact its subtle electron structure.

After investigating the properties of protons of leflunomide **1**, we examined their influence on the binding between teriflunomide **2** and amino acids in the DHODH cavity as hydrogen atoms are commonly involved in intramolecular interactions. Docking studies showed that teriflunomide **2** interacts with tyrosine Tyr356 and, through a water molecule, with arginine Arg136 in the binding site of DHODH [16, 29]. After optimization of the teriflunomide–tyrosine adduct at the B3LYP/6-31G(d,p)/CPCM (water as solvent) level of theory, we found that both molecules can interact through several hydrogen contacts.

As we have mentioned in the introductory section, previous studies on the structure of teriflunomide **2** suggested that compound **2** and some of its analogues existed mainly in a *Z* configuration stabilized by a strong intramolecular hydrogen bond (Scheme 1) [30–32]. Other reports, both experimental and theoretical, pointed out that the active form of teriflunomide was the *Z* configuration lacking the internal hydrogen contact [14, 17]. However, there are no reports that deal with the possibility of the *E* isomer formation and its interaction with DHODH. As we have indicated above, the exact physiological mechanism of the isoxazole ring opening in leflunomide is not known in detail and it is possible that this ring cleavage may provide both *Z* and *E* isomers. Besides, these isomers can be in equilibrium via the keto form (Scheme 1). Thus, we investigated these interactions by optimization of adducts between teriflunomide **2** and tyrosine, and both *E* and *Z* configurations were taken into consideration even though *E* and *Z* isomers have almost identical internal energy (-641,439.70 kcal mol^-1^).

In the first adduct, two hydrogen contacts were found (adduct **X**, Fig. S1 in the supplementary material; optimization using CPCM solvation model and water as solvent). These contacts involve the nitrile and hydroxyl groups of teriflunomide **2**, and hydroxyl group of tyrosine; the latter group acts as a donor and acceptor. The interaction energy of adduct **X** is −10.94 kcal mol^-1^.

The interaction between the tyrosine hydroxyl group and the amide proton of **2** (adduct **XI**, Fig. S2 in the supplementary material; optimization using CPCM solvation model and water as solvent) results in the formation of a N-H^…^O type of hydrogen contact (r = 1.018 Å, d = 2.038 Å, θ =173.2°). The interaction energy of the adduct is −3.53 kcal mol^-1^.

In adduct **XII** (Fig. S3, supplementary material; optimization using CPCM solvation model and water as solvent) between tyrosine and teriflunomide **2**, a hydrogen bond is formed between the teriflunomide carbonyl oxygen and tyrosine phenolic hydroxyl. The interaction energy is a little higher (−5.84 kcal mol^-1^) than that for the previous adduct.

The above results show that the highest interaction energy involves the hydroxyl groups of teriflunomide **2** and tyrosine (**X**). This is probably due to the relatively largest partial positive charge on the hydrogen atom of the teriflunomide hydroxyl group that lies in the proximity of the electron withdrawing nitrile and amide functionalities. The interaction between the nitrile group and tyrosine hydroxyl is of minor importance because this type of hydrogen bonding is usually classified as a weak to medium strong interaction (the *sp* hybridization of nitrogen atom) [33]. Moreover, the calculated angle θ (ca 135°) differs significantly from the typical angle for H-bonded nitriles that is usually close to linearity [34]. Nevertheless, the CN^…^HO hydrogen bond can still improve stability of the teriflunomide–tyrosine adduct.

The assumption concerning the contribution of the CN^…^HO hydrogen contact to the overall strength of the adduct is supported by the results obtained for the interaction between tyrosine amino and teriflunomide **2** nitrile groups (adduct **XIII**, Fig. S4 in the supplementary material; optimization using CPCM solvation model and water as solvent). Here, the interaction energy is only −2.41 kcal mol^-1^, so it is the lowest value from the interactions discussed above.

**Fig. 3:**
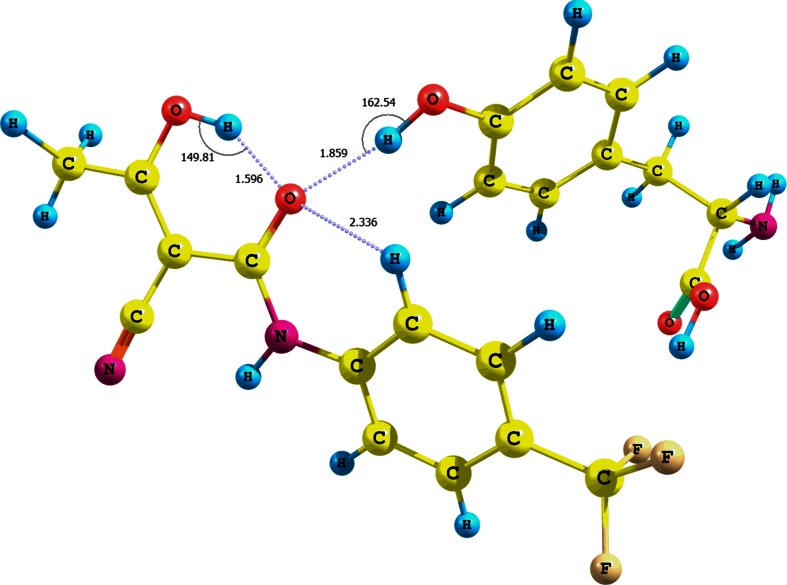
Structure of the (*Z*)-teriflunomide **2**–tyrosine adduct **XIV**; interaction between teriflunomide carbonyl group and tyrosine hydroxyl group

**Fig. 4:**
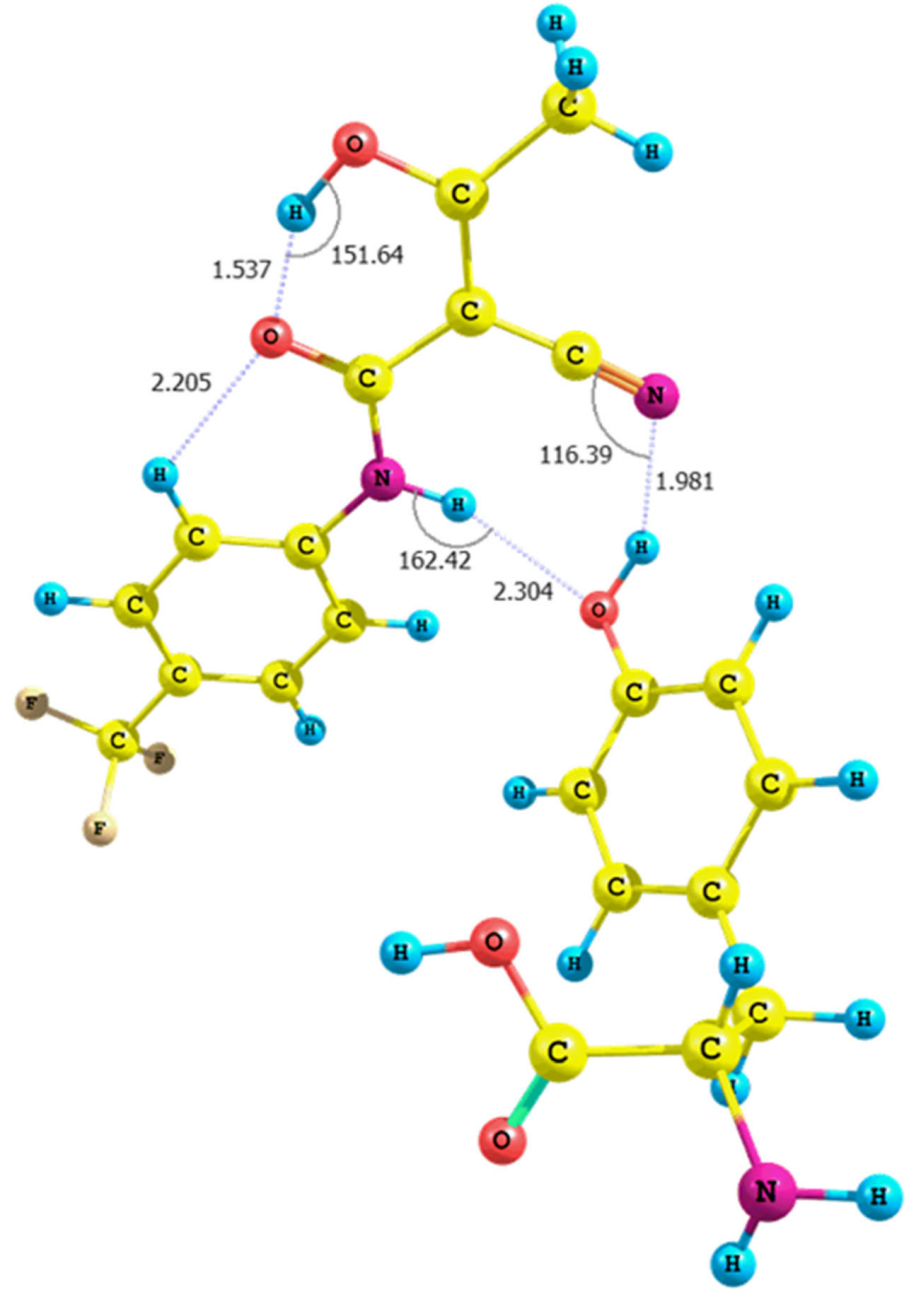
Structure of the (*Z*)-teriflunomide **2**–tyrosine adduct **XV**; interaction between teriflunomide amino group and tyrosine hydroxyl group

We also investigated the interactions of the tyrosine hydroxyl group with the hydroxyl and amide functionalities of teriflunomide **2** in a *Z* configuration, as well as on the interaction of the tyrosine amine group with the nitrile function of (*Z*)-teriflunomide (adducts **XIV**−**XVI**, Figs. 3 and 4, Fig. S7 in the supplementary material, Table 1; optimization using CPCM solvation model and water as solvent).

**Table 1:** Comparison of interaction energies for adducts involving (*Z*) and (*E*)-teriflunomide **2**; B3LYP/6-31G(d,p) level of theory

Teriflunomide **2**	*E* isomer	*Z* isomer			
Group involved in interaction	Adduct	Interaction energy [kcal mol^-1^]	Adduct	Interaction energy conformer **2** with internal H-bond [kcal mol^-1^]	Interaction energy conformer **2** without internal H-bond [kcal mol^-1^]
**OH**	**X**	−10.94	**XIV**	−4.90	−7.28
**NH**	**XI**	−3.53	**XV**	−7.14	−19.95
**CO**	**XII**	−5.84	**XVI**	−3.55	−7.67
**CN**	**XIII**	−2.41	**XVII**	−2.10	

The optimization of the adduct in which the (*Z*)-teriflunomide interacts with tyrosine hydroxyl results in a contact shift toward the teriflunomide carbonyl group (adduct **XIV**, Fig. 3). The interaction energy between the contacting molecules is −4.90 kcal mol^-1^. On the other hand, the rotation of the teriflunomide hydroxyl group by 180°, which breaks the intramolecular hydrogen bond, increases the interaction energy to −7.28 kcal mol^-1^ (Table 1). This evidently shows that if the intramolecular hydrogen contact existed it would weaken the intermolecular hydrogen bond between hydroxyl functionalities of teriflunomide and tyrosine.

The adduct **XV** is formed when the tyrosine hydroxyl group interacts with the amide nitrogen of (*Z*)-teriflunomide (the intramolecular hydrogen bond parameters: O-H^…^O = C (r = 1.019 Å, d = 1.537 Å, θ = 151.6 °); its energy is equal to −7.14 kcal mol^-1^ (Fig. 4). The interacting molecules are linked through two hydrogen bonds, i.e., a hydrogen HO_Tyr_^…^NH_teriflunomide_ contact (r = 2.027 Å, d = 2.304 Å, θ =162.4°) and a weaker one between the tyrosine hydroxyl group and nitrile π electrons (r = 0.976 Å, d = 1.981 Å, θ =116.4°). The optimization of the adduct led to lengthening of the tyrosine OH bond of 0.008 Å and an insignificant lengthening of the teriflunomide NH bond (0.002 Å). Reshaping of the teriflunomide hydroxyl configuration resulted in cleavage of the intramolecular OH^…^O=C bond and formation of a complex with the interaction energy of −19.95 kcal mol^-1^ (Table 1; optimization using CPCM solvation model and water as solvent). The teriflunomide NH bond is lengthened by 0.006 Å and linked with the tyrosine carboxyl group.

The calculated interaction energy between the tyrosine hydroxyl and (*Z*)-teriflunomide carbonyl is −3.55 kcal mol^-1^ (adduct **XVI**, Fig. S7 given in the supplementary material). The interacting molecules are connected through a HO_Tyr_^…^OC_teriflunomide_ hydrogen bond and the calculated change in the OH bond is quite small (0.003 Å). On the other hand, the interaction energy between the same functionalities but with the (*E*)-teriflunomide contribution (without the internal hydrogen bond) is −7.67 kcal mol^-1^ (Table 1). The tyrosine OH bond is lengthened by a similar value as for the *Z* isomer (0.002 Å). A visible structural difference between the *Z* and *E* isomers of teriflunomide is the spatial arrangement of the *p*-trifluoromethylphenyl ring. This ring is coplanar with the amide bond in the isomer *Z* but it is twisted from coplanarity in the isomer *E*. This deviation might also influence to some extent the strength of the intermolecular hydrogen bonding between teriflunomide and tyrosine.

Next, we compared the interactions between the amino group of tyrosine and nitrile of *Z* (adduct **XVII**, Fig. S8 given in the supplementary material) or *E* teriflunomide (adduct **XIII**, Fig. S4 in the supplementary material; optimization using CPCM solvation model and water as solvent). The interaction energies for both isomers is similar and equals to −2.41 and −2.10 kcal mol^-1^. Thus, the presence of the intramolecular C=O^…^HO hydrogen bond does not significantly affect the strength of the weakest interaction discussed here.

Previous studies showed that teriflunomide **2** also interacts with arginine through a water molecule in the active site of DHODH [30–32]. To examine whether this is a significant feature, we optimized the adducts of compound **2** with water, in which a single molecule of water interacted separately with the OH, NH, and CO groups of **2**. The respective BSSE corrected interaction energy values were −14.24, −5.80, and −4.18 kcal mol^-1^ (Table 2).

**Table 2:** Calculated interaction energy (kcal mol^-1^) of (*Z*)- or (*E*)-teriflunomide (**2**) with arginine via water molecule – conformers **XVIII**−**XXIII**; E_H2O_ – interaction energy of **2** with water; E – interaction energy of **2**–water–arginine adduct; B3LYP/6-31G(d,p) level of theory

Teriflunomide 2	*E* isomer	*Z* isomer	*E* isomer		*Z* isomer	
Complex	2–water		2–water–arginine			
Group of 2	*E*_H2O_ [kcal mol^-1^]	*E*_H2O_ [kcal mol^-1^]	Conformer	*E* [kcal mol^-1^]	Conformer	*Z* [kcal mol^-1^]
**OH**	−14.24	−11.50	**XVIII**	−23.95	**XXI**	−76.70
**NH**	−5.80	−6.50	**XIX**	−11.20	**XXII**	−8.82
**CO**	−4.18	−1.89	**XX**	−3.26	**XXIII**	−11.59

**Fig. 5:**
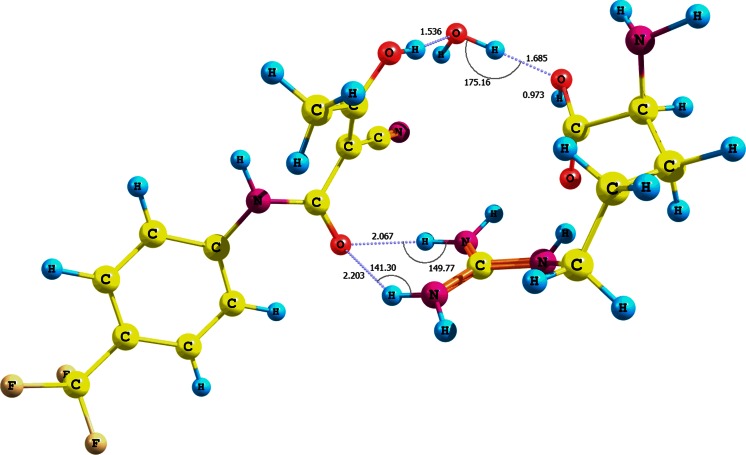
Structure of the (*E*)-teriflunomide **2**–water–arginine adduct **XVIII**; interaction of arginine carboxyl group with teriflunomide hydroxyl group via water molecule

Moreover, we analyzed the adducts of teriflunomide **2** (*E* configuration) and arginine with water participation and with particular attention to the amino acid carboxylic group (optimization using CPCM solvation model and water as solvent). The interaction energies of the adducts **XVIII**−**XX** are −23.95, −11.20, and −3.26 kcal mol^-1^, respectively (Fig. 5, Fig. S10 − S11 in the supplementary material, Table 2). The higher value for the adduct **XVIII** is probably due not only to the three hydrogen bonds, i.e., teriflunomide hydroxyl−water−arginine hydroxyl (r = 0.998 Å, d = 1.685 Å, θ =175.2°) and two teriflunomide CO−NH guanidine contacts (d_1_ = 1.015 Å, r_1_ = 2.067 Å, θ =149.8°, as well as r_2_ = 1.012 Å, d_2_ = 2.203 Å, θ =141.3°), but also to a quasi-ionic interaction between a protonated guanidine fragment of the amino acid and the polarized amide carbonyl. The structure of the adduct is in agreement with the docking studies [16, 29].

The adduct teriflunomide **2**−water−arginine **XIX** (Fig. S10 in the supplementary material) is stabilized by two interactions: a. a strong hydrogen bond between the amide NH and arginine carboxylic group with participation of water; b. a weak contact between the nitrile group and carboxylic hydroxyl.

The adduct **XX** (Fig. S11, supplementary material) with the lowest interaction energy is, in turn, stabilized by hydrogen bonding that involves the carboxylic group (arginine) and amide carbonyl group (**2**) through a water molecule. The arginine carboxylic group is linked to water through both carbonyl and hydroxyl elements of this functionality. The interaction associated with the carboxylic hydroxyl seems to be a weaker one because of a comparatively longer distance *d* (1.933 Å). The energy relative to the OH, NH, and CO groups of (*Z*)-teriflunomide interacting with water is −11.50, −6.50, and −1.89 kcal mol^-1^, respectively (Table 2). Similarly to the interaction of (*E*)-teriflunomide with water, the weakest affinity to water can be observed for the NH group of (*Z*)-teriflunomide.

**Fig. 6:**
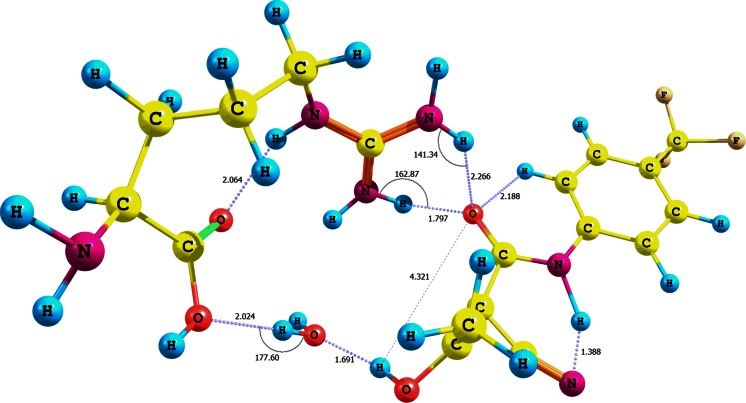
Structure of the (*Z*)-teriflunomide **2** −water–arginine adduct **XXI**; interaction of arginine carboxyl group with teriflunomide hydroxyl group via water molecule

Somewhat different conclusions can be drawn from the analysis of the adducts (*Z*)-teriflunomide−water−arginine **XXI**−**XXIII** (Fig. 6, Figs. S13 and S14 in the supplementary material, Table 2; optimization using CPCM solvation model and water as solvent). The individual interaction energies of arginine with the OH, NH, or CO groups of (*Z*)-teriflunomide with the contribution of water are as follows: −76.70, −8.82, or −11.59 kcal mol^-1^. On the other hand, the values for the same interactions for teriflunomide with a 180° rotated hydroxyl group are −52.84, −9.81, and −13.01 kcal mol^-1^, respectively.

Analogously to (*E*)-teriflunomide, the (*Z*) configuration of this metabolite generates a similar set of hydrogen bonds (**XXI**). A hydrogen contact is present between the hydroxyl group of arginine and water (d = 2.024 Å, r = 0.972 Å, θ =177.6°). The adduct is also stabilized by a bifurcated contact between the guanidine residue and teriflunomide carbonyl (r_1_ = 1.029 Å, d_1_ = 1.797 Å, θ =162.9° oraz r_2_ = 1.015 Å, d_2_ = 2.266 Å, θ =141.3°). The almost twice lower interaction energy for the adduct (*Z*)-teriflunomide−arginine in comparison with the adduct that involves the *E* isomer is probably due to a lower electron density of the carbonyl oxygen and lower accessibility of the hydroxyl hydrogen, both engaged in the intramolecular hydrogen bond. Analyzing the data depicted in Table 2, we can conclude that the contribution of water to the overall interaction energy of the (*Z*)-teriflunomide−water−arginine complex is higher than the analogous contribution to the adduct involving *E* isomer. Thus, the *E* configuration is preferred for the direct interaction teriflunomide−arginine.

Our results clearly indicate that the hydroxyl, nitrile, and amide groups contribute to the interactions of teriflunomide **2** with arginine through water and are in agreement with the previous reports [16, 29].

**Fig. 7:**
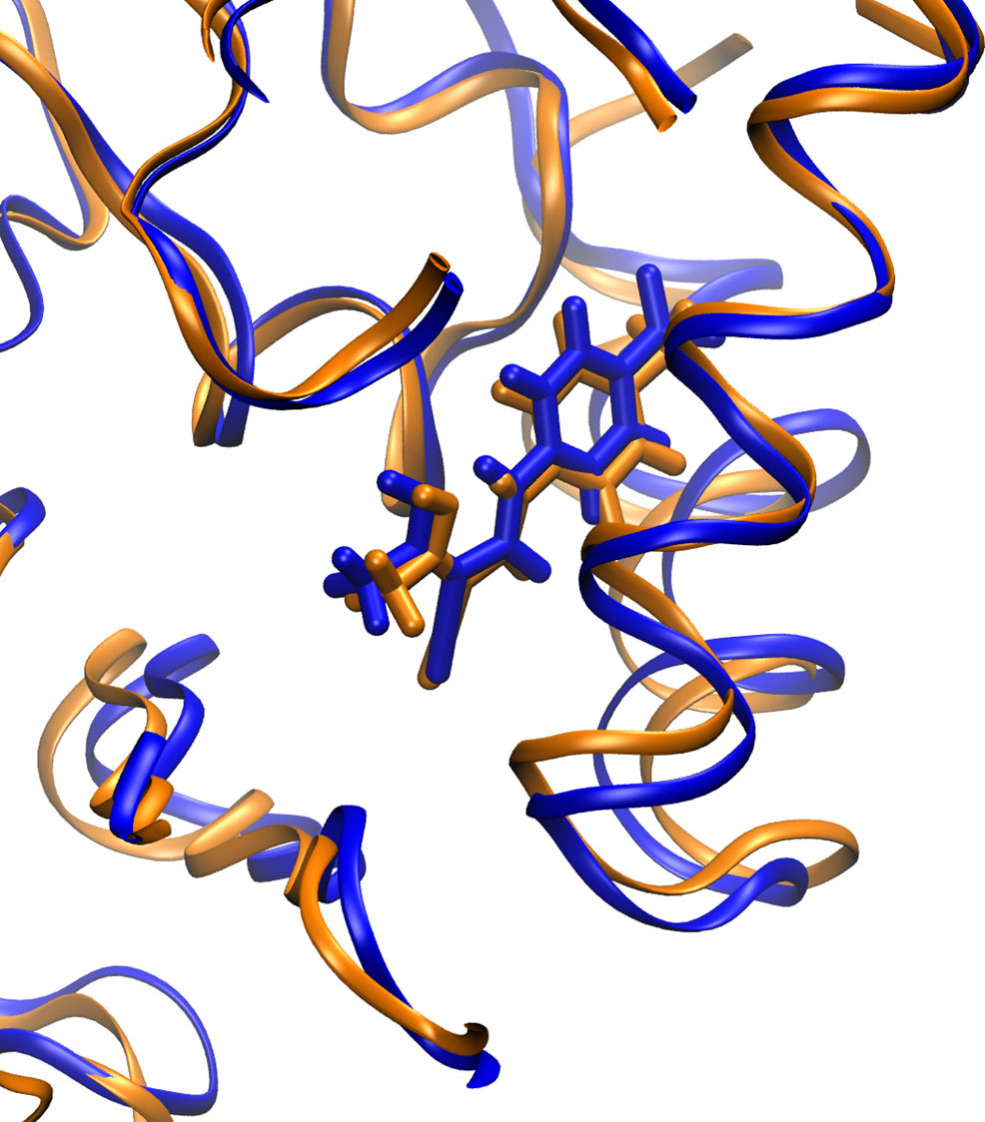
Structure of the teriflunomide **2**−DHODH complex (enhanced) optimized using ONIOM method; blue – optimized model, orange – original structure of DHODH complex (3GOU.pdb file)

In order to prove a crucial role of the amide functionality in the stability of teriflunomide−amino acid adduct within the receptor cavity we carried out the quantum mechanics/molecular mechanics (QM:MM) calculations using the ONIOM method [35] implemented in the *Gaussian* software. The human dihydroorotate dehydrogenase (DHODH) in complex with a leflunomide derivative inhibitor 4 taken from the Protein Data Bank base (3GOU.pdb) was chosen as the biological target [16, 36]. An initial target for further optimization was prepared by removing the internal ligand from the 3GOU.pdb file ((2Z)-N-(3-chloro-2'-methoxybiphenyl-4-yl)-2-cyano-3-hydroxybut-2-enamide, an analogue of teriflunomide), but keeping the internal coordinates unchanged. Then the internal ligands were replaced by the optimized structure of **2** and additionally the residues were saturated with hydrogen atoms. In this manner we prepared two input models. For the low layer we chose the UFF force field (MM calculations) [37], and for the high layer the semi-empiric PM6 method (QM calculations) [38]. The main difference between these ONIOM models is that in the first one all atoms are optimized, whereas in the second one only the linking atoms as well as the QM layer undergo optimization. The QM layer consisted of the ligand and all residues containing atoms connected with the ligand (closer than 4 Å). In the supplementary material two inputs for the ONIOM models are given. The results of our calculations are depicted in Fig. 7 and Table 3. The outputs were visualized using the VMD package [39]. The results of our calculations prove the importance of the amide bond for the stability of the **2**−amino acid adduct within DHODH cavity and are in good agreement with the experimental data involving the original receptor. This suggests that the binding mode of **2** and an analogue of teriflunomide (3GOU protein with internal ligand) are similar. Furthermore, it seems that the *E* isomer of teriflunomide might provide a stronger teriflunomide−amino acid type of interaction.

**Table 3:** Calculated distances (Å) between optimized teriflunomide (**2**) and corresponding amino acid within DHODH cavity; 3GOU – original receptor DHODH taken from the PDB data base (non optimized), FMN – flavin mononucleotide (cofactor), model 1 – first calculated (ONIOM PM6:UFF) model (RMS = 1.763), model 2 – second calculated (ONIOM PM6:UFF) model (RMS = 0.187), Tyr – tyrosine, Arg – arginine, Pro – proline, Leu – leucine, Val – valine

Interaction 2−amino acid	3GOU	Model 1	Model 2
**OH** ^…^ **O**_**Tyr356**_	2.710	2.546	2.570
**CN** ^…^ **H**-**N**_ε(**Arg136**)_	3.499	3.106	3.076
**CH**_**3**_^…^ **CH**_**3**(**FMN**)_	4.037	3.420	4.112
**CH**_**3**_^…^ **CH**_**3**(**Val134**)_	4.006	3.615	4.037
**CN** ^…^ **O**_**Pro52**_	4.100	4.853	4.128
**CH**_**phenyl**_^…^ **CH**_**3**(**Leu46**)_	3.847	3.401	3.589
**F**_**3**_ **C** ^…^ **C**_**ring**(**Pro364**)_	4.058	4.716	4.476
**C** = **O**_**amide**_^…^(**H**_**2**_ **N**)_**2**_ **C**_**Arg136**_	4.308	2.862	2.823

## Conclusions

The NMR estimation proved that the amide bond of leflunomide might be involved in the hydrogen bond forming. Our investigations have shown that the use of more complex basis sets or diffuse functions during optimization does not increase the correlation between the calculated and experimental values of chemical shifts. Taking into consideration the accuracy of calculations as well as time and cost required to complete them, the use of GIAO method in the NMR analysis of rotamers of leflunomide seems to be the appropriate choice.

The results of theoretical studies have also shown that the interactions of teriflunomide with tyrosine and arginine involve principally the amide fragment of teriflunomide. The teriflunomide nitrile functionality is a minor contributor to these interactions. Our calculations confirm that the presence of the internal hydrogen bond between (*Z*)-teriflunomide carbonyl oxygen and enolic hydroxyl decreases the interaction strength between teriflunomide and tyrosine or arginine. Moreover, even the *E* isomer of teriflunomide, if ever formed under physiological conditions, would usually provide a stronger interaction teriflunomide−amino acid than the *Z* isomer with the internal hydrogen bond.

## Computational section

Density functional calculations were executed and the geometries of compounds were optimized at the DFT level of theory using the *Gaussian 09 D.01* program [22], B3LYP functional, 6-31G(d,p) and 6-311+G(d,p) basis set, and conductor-like polarizable continuum model (CPCM, water as a solvent) [23–25]. The vibrational frequencies and thermodynamic properties were calculated by applying the ideal gas, rigid rotor, and harmonic oscillator approximations, energy minimum was confirmed by the frequency calculation for all conformers, no negative frequencies were detected in generated vibrational spectrum of analyzed conformers. The conformers were obtained by rotating the bonds C9-N1, N1-C2, and C2-C11 (leflunomide **1**) or C12-N2, N2-C5, and C5-C3 (teriflunomide **2**) in dihedral angle increments of 20°, a total of 52 conformers were obtained. NMR shielding for proton (H^ref^) was calculated for TMS at B3LYP/6-31G(d,p) level of theory (CPCM solvation model and water as solvent). The compound of interest (**1**) and reference compound (TMS) were calculated using the same method, and the reference compound was used to obtain the chemical shifts of **1** according to the following equation: δ_i_ = σ_ref_ − σ_i_, where δ_i_ was chemical shift of i-nuclei of **1**, σ_ref_ and σ_i_ were the calculated isotropic magnetic shielding tensor for the TMS and **1**, respectively [18, 40]. The calculated chemical shifts for the homotopic protons of methyl groups A or B (Scheme 1) were averaged. The calculated chemical shifts of protons C or D were averaged as well. Interaction energy was calculated using counterpoise method based on the basis set superposition error (BSSE) at B3LYP/6-31G(d,p) level of theory [27, 28]. The *Chemcraft 1.7* software was utilized for the visualization of all optimized conformers [41]. All ONIOM (PM6:UFF) calculations were carried out as implemented in the *Gaussian 09 D.01* program [35]. The human dihydroorotate dehydrogenase in complex with a leflunomide derivative inhibitor 4, acquired from the Protein Data Bank base (3GOU.pdb), was selected as the biological target [16, 36]. An initial target for further optimization was prepared by removing the internal ligand from the 3GOU.pdb file (an analogue of teriflunomide), but keeping the internal coordinates unchanged. Then the internal ligands were replaced by the optimized structure of **2** and additionally the residues were saturated with hydrogen atoms. Two input models were prepared in this manner. The outputs were visualized using the *VMD* package [39]. The calculations were carried out using resources provided by Poznan Supercomputing and Networking Center (*Reef* cluster), as well as Wrocław Center for Networking and Supercomputing (*Supernova* cluster).

## Electronic supplementary material

Below is the link to the electronic supplementary material.

ESM 1 (DOCX 4099 kb)
