# Supplementary material for: On the interactions of leflunomide and teriflunomide within receptor cavity — NMR studies and energy calculations
Source: J Mol Model. 2015 Apr 8;21(5):105. doi: 10.1007/s00894-015-2643-z (PMC4391734; doi:10.1007/s00894-015-2643-z)
Supplement: Supplementary file 1 — (DOCX 4099 kb) [file 894_2015_2643_MOESM1_ESM.docx]

**Supporting Information**

**On the interactions of leflunomide and teriflunomide within receptor cavity – NMR studies and energy calculations**

*Jacek Kujawski*, Marek K. Bernard, Elżbieta Jodłowska, Kornelia Czaja, Beata Drabińska*

Department of Organic Chemistry, Faculty of Pharmacy, Poznan University of Medical Sciences, Grunwaldzka 6 street, 60-780 Poznań, Poland

*corresponding author: jacekkuj@ump.edu.pl, phone 486185466701, fax 48618546680

Table of Contents:

[Table S1. Calculated and experimental data for leflunomide conformers I–IV 3](#_Toc409212718)

[Table S2. Calculated and experimental data for leflunomide conformers V–VIII 3](#_Toc409212719)

[Table S3. Calculated and experimental data for leflunomide conformer IX 3](#_Toc409212720)

[Table S4. Calculated and experimental data for leflunomide conformers I–VIII 3](#_Toc409212721)

[Figure S1. Structure of the (E)-teriflunomide 2–tyrosine adduct X 4](#_Toc409212722)

[Figure S2. Structure of (E)-teriflunomide 2–tyrosine adduct XI 6](#_Toc409212723)

[Figure S3. Structure of (E)-teriflunomide 2–tyrosine adduct XII 8](#_Toc409212724)

[Figure S4. Structure of (E)-teriflunomide 2–tyrosine adduct XIII 10](#_Toc409212725)

[Figure S5. Structure of the (Z)-teriflunomide 2–tyrosine adduct XIV 12](#_Toc409212726)

[Figure S6. Structure of the (Z)-teriflunomide 2–tyrosine adduct XV 13](#_Toc409212727)

[Figure S7. Structure of the (Z)-teriflunomide 2–tyrosine adduct XVI 15](#_Toc409212728)

[Figure S8. Structure of the (Z)-teriflunomide 2−tyrosine adduct XVII 17](#_Toc409212729)

[Figure S9. Structure of the (E)-teriflunomide 2–water–arginine adduct XVIII 18](#_Toc409212730)

[Figure S10. Structure of the (E)-teriflunomide 2–water–arginine adduct XIX 21](#_Toc409212731)

[Figure S11. Structure of the (E)-teriflunomide 2–water–arginine adduct XX 23](#_Toc409212732)

[Figure S12. Structure of the (Z)-teriflunomide 2−water–arginine adduct XXI 25](#_Toc409212733)

[Figure S13. Structure of the (Z)-teriflunomide 2−water–arginine adduct XXII 27](#_Toc409212734)

[Figure S14. Structure of the (Z)-teriflunomide 2−water–arginine adduct XXIII 29](#_Toc409212735)

[Figure S15. Structure of the model 1 subjected for ONIOM calculations (PM6:UFF) 31](#_Toc409212736)

[Figure S16. Structure of the model 2 subjected for ONIOM calculations (PM6:UFF) 174](#_Toc409212737)

Table S1. Calculated and experimental data for leflunomide conformers I–IV; the following parameters were determined for their proton groups: experimental (Exp.) and calculated values of the chemical shifts, partial absolute errors (δ1-δ4), mean values of the absolute and relative percentage errors (∆δ and E, respectively); calculated NMR shielding for proton H^ref^ = 31.7397 ppm for TMS (B3LYP/6-31G(d,p)/GIAO/CPCM).

| **Proton signals** | **Exp.** | **I** | **II** | **III** | **IV** | **δ1** | **δ2** | **δ3** | **δ4** | **Δδ** | **E** |
| --- | --- | --- | --- | --- | --- | --- | --- | --- | --- | --- | --- |
| **A** | 2.62 | 2.80 | 2.82 | 2.81 | 2.76 | 0.18 | 0.20 | 0.19 | 0.14 | 0.18 | **7** |
| **NH** | 10.32 | 7.38 | 7.38 | 7.38 | 7.24 | 2.94 | 2.94 | 2.94 | 3.08 | 2.97 | ***29*** |
| **B** | 7.9 | 7.77 | 7.77 | 7.77 | 7.77 | 0.13 | 0.13 | 0.13 | 0.13 | 0.13 | **2** |
| **C** | 7.68 | 8.08 | 8.08 | 8.08 | 8.06 | 0.40 | 0.40 | 0.40 | 0.38 | 0.39 | **5** |
| **D** | 9.07 | 8.49 | 8.49 | 8.49 | 8.65 | 0.58 | 0.58 | 0.58 | 0.42 | 0.54 | **6** |

Table S2. Calculated and experimental data for leflunomide conformers V–VIII; the following parameters were determined for their proton groups: experimental (Exp.) and calculated values of the chemical shifts, partial absolute errors (δ5-δ8), mean values of the absolute and relative percentage errors (∆δ and E, respectively); calculated NMR shielding for proton H^ref^ = 31. 9753 ppm for TMS (B3LYP/6-311+G(d,p)/GIAO/CPCM).

| **Proton signals** | **Exp.** | **V** | **VI** | **VII** | **VIII** | **δ5** | **δ6** | **δ7** | **δ8** | **Δδ** | **E** |
| --- | --- | --- | --- | --- | --- | --- | --- | --- | --- | --- | --- |
| **A** | 2.62 | 2.86 | 2.86 | 2.88 | 2.78 | 0.24 | 0.24 | 0.26 | 0.16 | 0.22 | **8** |
| **NH** | 10.32 | 7.43 | 7.44 | 7.55 | 7.32 | 2.89 | 2.88 | 2.77 | 3.00 | 2.89 | ***28*** |
| **B** | 7.90 | 7.94 | 7.90 | 7.85 | 7.86 | 0.04 | 0.00 | 0.05 | 0.04 | 0.01 | **0.1** |
| **C** | 7.68 | 8.13 | 8.13 | 8.25 | 8.13 | 0.45 | 0.45 | 0.57 | 0.45 | 0.48 | **6** |
| **D** | 9.07 | 8.51 | 8.50 | 8.53 | 8.62 | 0.56 | 0.57 | 0.54 | 0.45 | 0.53 | **6** |

Table S3. Calculated and experimental data for leflunomide conformer IX; the following parameters were determined for its proton groups: experimental (Exp.) and calculated values of the chemical shifts, absolute error (δ9) and relative percentage error (E); calculated NMR shielding for proton H^ref^ = 31.7397 ppm for TMS (B3LYP/6-31G(d,p)/GIAO/CPCM).

| **Proton signals** | **Exp.** | ***GIAO* method** | | |
| --- | --- | --- | --- | --- |
|  |  | **IX** | **δ9** | **E** |
| **A** | 2.62 | 2.71 | 0.09 | **3** |
| **NH** | 10.32 | 10.89 | 0.57 | ***5*** |
| **B** | 7.90 | 7.80 | 0.10 | **1** |
| **C** | 7.68 | 8.39 | 0.71 | **9** |
| **D** | 9.07 | 8.42 | 0.65 | **7** |

Table S4. Calculated and experimental data for leflunomide conformers I–VIII
(**B3LYP/6-311++G(3df,2pd**); the following parameters were determined for their proton groups: experimental (Exp.) and calculated values of the chemical shifts, mean values of the absolute and relative percentage errors (δ and E, respectively); calculated NMR shielding for proton H^ref^ = 31.8821 ppm for TMS (B3LYP/6-311++G(3df,2pd)/GIAO; in vacuo) or NMR shielding for proton H^ref^ = 31.7256 ppm for TMS (B3LYP/6-311++G(3df,2pd)/GIAO/CPCM).

| **Proton signals** | **Exp.** | Gas (in vacuo) | | | PCM | | |
| --- | --- | --- | --- | --- | --- | --- | --- |
|  |  | **I**–**VIII** | δ | **E** | **I**–**VIII** | δ | **E** |
| **A** | 2.62 | 2.78 | 0.16 | **6** | 2.57 | 0.05 | **2** |
| **NH** | 10.32 | 6.65 | 3.67 | **36** | 7.23 | 3.09 | **30** |
| **B** | 7.9 | 7.69 | 0.21 | **3** | 7.65 | 0.25 | **3** |
| **C** | 7.68 | 7.89 | 0.21 | **3** | 7.89 | 0.21 | **3** |
| **D** | 9.07 | 8.06 | 1.01 | **11** | 8.34 | 0.73 | **8** |

Figure S1. Structure of the (E)-teriflunomide 2–tyrosine adduct X; interaction between teriflunomide hydroxyl and nitrile groups and tyrosine hydroxyl group.


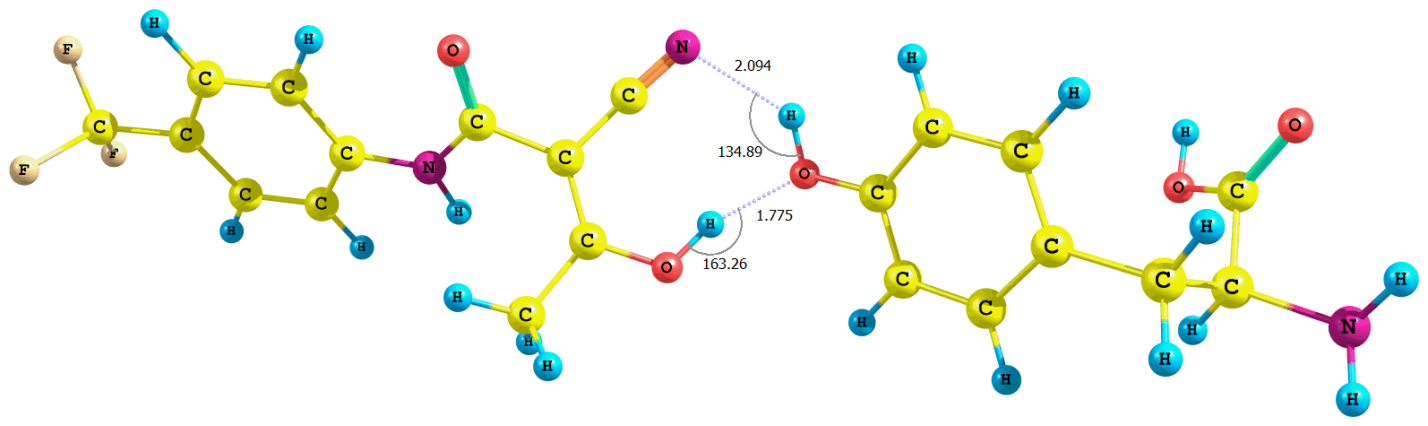
Cartesian coordinates:

O 0.654872000 1.374465000 0.324632000

N 0.538563000 -2.160349000 0.542235000

C -1.393827000 2.292570000 -0.294142000

C -0.614019000 1.068133000 0.082813000

C -1.120414000 -0.214517000 0.137056000

C -0.225650000 -1.299101000 0.354053000

O -2.740813000 -1.686633000 -0.774627000

C -2.527184000 -0.648799000 -0.151241000

N -3.515540000 0.153024000 0.367060000

F -9.582569000 0.776919000 -1.240415000

F -9.832061000 0.465470000 0.893797000

F -9.679760000 -1.243507000 -0.451468000

C -9.191074000 -0.006204000 -0.201483000

C -7.701987000 -0.010435000 -0.027141000

C -7.086206000 0.952411000 0.778950000

C -6.915797000 -0.939480000 -0.712436000

C -5.703092000 0.981081000 0.894398000

C -5.528040000 -0.921774000 -0.602201000

C -4.909701000 0.047070000 0.205341000

H 1.237950000 0.635233000 0.630620000

H -2.320696000 2.061740000 -0.814782000

H -1.621983000 2.882771000 0.600796000

H -0.762662000 2.912628000 -0.935464000

H -3.215855000 0.876633000 1.005557000

H -7.685504000 1.675570000 1.320716000

H -7.387194000 -1.693188000 -1.333719000

H -5.231668000 1.730982000 1.522866000

H -4.924956000 -1.649503000 -1.123655000

H 2.337487000 -1.235415000 1.084486000

O 2.635832000 -0.312490000 1.176183000

H 4.157651000 -2.173377000 -0.026538000

C 4.560474000 -1.164498000 -0.037344000

C 3.850547000 -0.117370000 0.552229000

C 4.362202000 1.182551000 0.529816000

H 3.795338000 1.987793000 0.985921000

H 5.974714000 2.441220000 -0.105621000

C 5.587759000 1.425656000 -0.085301000

C 6.325118000 0.391768000 -0.682629000

C 5.788376000 -0.901693000 -0.646704000

H 6.333968000 -1.721888000 -1.105519000

O 8.567378000 -0.646577000 1.623479000

H 8.675309000 -1.553683000 1.962463000

C 9.025860000 -0.629894000 0.360132000

O 9.499171000 -1.606098000 -0.189411000

C 8.864404000 0.725196000 -0.315895000

N 10.151305000 1.054461000 -0.933758000

H 10.375264000 0.330918000 -1.615395000

H 10.054934000 1.921910000 -1.457804000

H 8.642695000 1.471321000 0.452639000

H 7.901749000 -0.100900000 -2.065518000

C 7.670668000 0.665322000 -1.317166000

H 7.650652000 1.630049000 -1.836715000

Figure S2. Structure of (E)-teriflunomide 2–tyrosine adduct XI; interaction between teriflunomide NH group and tyrosine hydroxyl group.


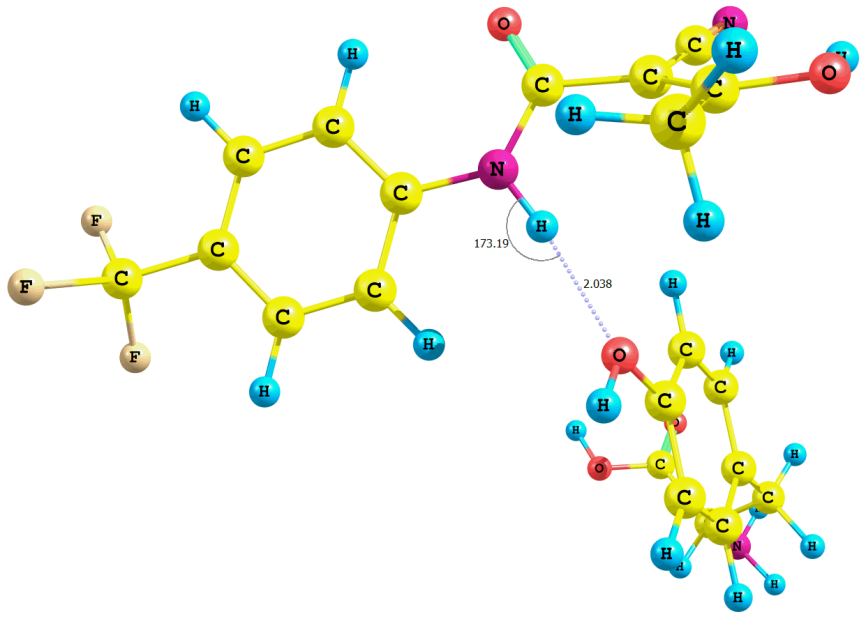


Cartesian coordinates:

O 0.388748000 5.122669000 0.804028000

N 0.360601000 4.686884000 -2.627670000

C -1.172499000 3.809799000 1.948512000

C -0.526748000 4.157153000 0.646456000

C -0.822576000 3.622325000 -0.583232000

C -0.190756000 4.193188000 -1.725968000

O -2.630825000 2.751588000 -1.845743000

C -1.869929000 2.589686000 -0.893700000

N -1.861579000 1.483493000 -0.082704000

F -6.186763000 -2.958056000 1.104804000

F -4.661092000 -4.205002000 0.195182000

F -6.114137000 -3.167481000 -1.056013000

C -5.339012000 -3.041714000 0.046065000

C -4.419438000 -1.860909000 -0.037711000

C -3.238359000 -1.840420000 0.711829000

C -4.756709000 -0.754629000 -0.820311000

C -2.409362000 -0.727695000 0.674608000

C -3.931664000 0.366122000 -0.866855000

C -2.746008000 0.387836000 -0.113574000

H 0.824294000 5.341775000 -0.038162000

H -2.065526000 3.201745000 1.821348000

H -0.458067000 3.269017000 2.576764000

H -1.433392000 4.737868000 2.464602000

H -1.059387000 1.374509000 0.534800000

H -2.960957000 -2.696367000 1.317360000

H -5.667986000 -0.766283000 -1.408040000

H -1.489007000 -0.718397000 1.248921000

H -4.191068000 1.211927000 -1.485366000

H 0.278429000 0.682692000 2.672391000

O 0.503271000 0.918228000 1.760648000

H 1.679422000 1.327903000 -0.511090000

C 2.289427000 0.780734000 0.199958000

C 1.781793000 0.492422000 1.468159000

C 2.560808000 -0.200044000 2.398124000

H 2.166391000 -0.418478000 3.387092000

H 4.451433000 -1.132583000 2.783029000

C 3.850043000 -0.601472000 2.049880000

C 4.383433000 -0.328064000 0.782894000

C 3.579345000 0.369043000 -0.130622000

H 3.968828000 0.600292000 -1.118479000

O 3.850041000 -3.079239000 -1.091709000

H 3.365954000 -3.117786000 -1.936435000

C 5.019512000 -2.458381000 -1.323181000

O 5.357009000 -2.062134000 -2.422285000

C 5.858560000 -2.278765000 -0.064944000

N 7.215548000 -2.729805000 -0.382680000

H 7.574631000 -2.156655000 -1.144802000

H 7.819226000 -2.553124000 0.417735000

H 5.449107000 -2.922863000 0.718706000

H 6.180200000 -0.167686000 -0.393079000

C 5.772225000 -0.795233000 0.407202000

H 6.444944000 -0.701453000 1.267290000

Figure S3. Structure of (E)-teriflunomide 2–tyrosine adduct XII; interaction between teriflunomide carbonyl group and tyrosine hydroxyl group.


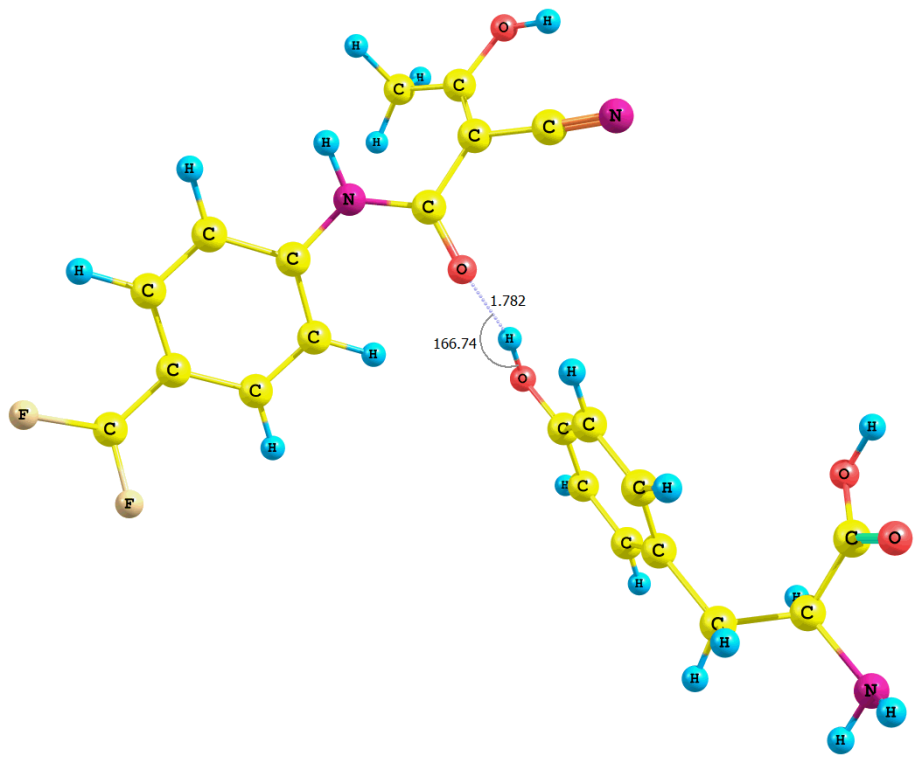


Cartesian coordinates:

O 2.023863000 5.924254000 0.339107000

N -0.694984000 4.534605000 -1.238001000

C 3.598309000 4.387711000 1.126264000

C 2.333161000 4.625710000 0.364756000

C 1.528989000 3.674057000 -0.223061000

C 0.293715000 4.112655000 -0.786610000

O 0.750833000 1.437954000 -0.193651000

C 1.727170000 2.197697000 -0.243043000

N 3.010534000 1.749851000 -0.366701000

F 5.788974000 -3.742340000 1.002629000

F 6.093063000 -3.744735000 -1.147617000

F 4.231478000 -4.503707000 -0.304303000

C 5.165794000 -3.531942000 -0.185342000

C 4.566340000 -2.159245000 -0.271287000

C 5.315594000 -1.095081000 -0.780958000

C 3.276418000 -1.926985000 0.212077000

C 4.773421000 0.183416000 -0.811678000

C 2.723924000 -0.649658000 0.188029000

C 3.475473000 0.416898000 -0.330229000

H 1.213818000 6.092736000 -0.174347000

H 3.683778000 3.364158000 1.485629000

H 4.465373000 4.621377000 0.497600000

H 3.621300000 5.073500000 1.976418000

H 3.702617000 2.435180000 -0.637454000

H 6.316616000 -1.263639000 -1.161446000

H 2.691131000 -2.749085000 0.609066000

H 5.356861000 1.005807000 -1.214705000

H 1.725594000 -0.485191000 0.564706000

H -0.338248000 0.822644000 1.075400000

O -0.804371000 0.301507000 1.760885000

H -1.702910000 0.515728000 -0.761063000

C -2.301233000 -0.109336000 -0.105111000

C -1.913599000 -0.284620000 1.230332000

C -2.687436000 -1.098833000 2.069223000

H -2.378258000 -1.235688000 3.100891000

H -4.412005000 -2.359242000 2.236278000

C -3.828071000 -1.724652000 1.573684000

C -4.233018000 -1.562212000 0.239610000

C -3.447876000 -0.744198000 -0.583762000

H -3.732808000 -0.601836000 -1.623455000

O -6.562976000 0.893613000 0.256476000

H -6.533704000 1.722383000 -0.254991000

C -6.798451000 -0.107191000 -0.609585000

O -6.960642000 0.069770000 -1.801957000

C -6.814171000 -1.478499000 0.051786000

N -8.024982000 -2.165045000 -0.407956000

H -7.980300000 -2.248131000 -1.422662000

H -8.023143000 -3.115735000 -0.043668000

H -6.880048000 -1.339146000 1.134807000

H -5.435802000 -2.344598000 -1.367447000

C -5.488457000 -2.228014000 -0.278895000

H -5.585115000 -3.232915000 0.148578000

Figure S4. Structure of (E)-teriflunomide 2–tyrosine adduct XIII; interaction between teriflunomide nitrile group and tyrosine amine group.


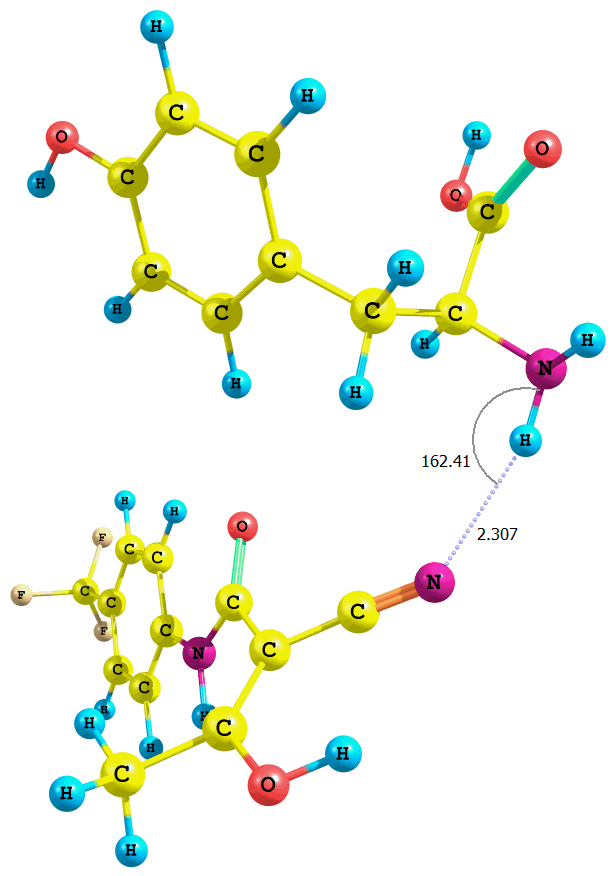


Cartesian coordinates:

O -0.616478000 4.807608000 1.656017000

N -2.120537000 3.271063000 -1.062458000

C 1.294645000 3.711653000 2.435946000

C 0.210558000 3.786218000 1.408337000

C 0.019753000 2.924810000 0.353672000

C -1.153359000 3.088594000 -0.436926000

O 0.295648000 0.668253000 -0.331295000

C 0.840763000 1.717761000 0.006408000

N 2.199589000 1.899173000 0.051500000

F 7.021944000 -2.125319000 0.604463000

F 7.563675000 -1.209168000 -1.286503000

F 6.191310000 -2.902659000 -1.246535000

C 6.528927000 -1.759583000 -0.607266000

C 5.372317000 -0.813241000 -0.482620000

C 5.610030000 0.550536000 -0.280049000

C 4.059300000 -1.286364000 -0.513660000

C 4.545154000 1.424096000 -0.110428000

C 2.982626000 -0.418173000 -0.349270000

C 3.222049000 0.949597000 -0.142506000

H -1.315485000 4.879322000 0.982788000

H 1.818901000 2.758677000 2.424422000

H 2.015267000 4.521864000 2.277663000

H 0.845812000 3.866109000 3.420806000

H 2.517330000 2.852458000 0.162559000

H 6.625122000 0.931714000 -0.260147000

H 3.868501000 -2.341344000 -0.675690000

H 4.737332000 2.481276000 0.046768000

H 1.968700000 -0.786760000 -0.387324000

O -2.793412000 -4.556819000 2.387564000

H -1.830659000 -4.653858000 2.380906000

C -3.131159000 -3.353232000 1.831936000

C -4.490827000 -3.030103000 1.764739000

H -5.221854000 -3.733274000 2.151140000

C -2.179617000 -2.450485000 1.343423000

H -1.121512000 -2.695267000 1.398687000

C -2.590491000 -1.236103000 0.793505000

H -1.836601000 -0.543146000 0.426819000

C -3.948461000 -0.894280000 0.716247000

C -4.885463000 -1.813707000 1.211276000

H -5.944614000 -1.572747000 1.167822000

C -4.384462000 0.412404000 0.090821000

H -3.670757000 1.205447000 0.337457000

H -5.360094000 0.717789000 0.486327000

O -6.766911000 -0.411938000 -1.694374000

C -4.492740000 0.385117000 -1.462372000

C -5.578973000 -0.597816000 -1.878856000

H -3.540116000 0.042778000 -1.876767000

H -5.851992000 -2.281981000 -2.674705000

O -5.093579000 -1.707577000 -2.464227000

N -4.822405000 1.678789000 -2.067594000

H -4.101126000 2.349564000 -1.803598000

H -5.696622000 2.010127000 -1.662526000

Figure S5. Structure of the (Z)-teriflunomide 2–tyrosine adduct XIV; interaction between teriflunomide carbonyl group and tyrosine hydroxyl group.

Cartesian coordinates:

O 5.109755000 -2.433058000 0.590190000

N 6.303585000 1.513882000 -1.825442000

C 7.105717000 -1.882336000 -0.538513000

C 5.721590000 -1.512305000 -0.121478000

C 5.101969000 -0.308157000 -0.438834000

C 5.789873000 0.672348000 -1.201502000

O 3.115342000 -0.900499000 0.703280000

C 3.728069000 -0.040954000 0.019579000

N 3.181587000 1.152544000 -0.327560000

F -2.878948000 2.887059000 -0.224500000

F -1.980824000 4.760450000 0.396861000

F -2.432118000 3.228685000 1.878829000

C -1.953545000 3.434424000 0.637027000

C -0.593160000 2.842500000 0.432044000

C 0.214224000 3.312038000 -0.609617000

C -0.143890000 1.797435000 1.241044000

C 1.452237000 2.730046000 -0.842040000

C 1.101236000 1.213305000 1.020625000

C 1.902276000 1.671518000 -0.035237000

H 4.197799000 -2.068619000 0.811475000

H 7.587053000 -1.099931000 -1.122978000

H 7.704262000 -2.090310000 0.353936000

H 7.066554000 -2.804152000 -1.127400000

H 3.745272000 1.733026000 -0.937259000

H -0.121742000 4.129741000 -1.237463000

H -0.760101000 1.439328000 2.057704000

H 2.073762000 3.092597000 -1.654996000

H 1.439840000 0.414850000 1.663367000

H 1.584103000 -1.814990000 1.226454000

O 0.921284000 -2.289321000 1.761384000

H 0.442405000 -1.516097000 -0.767453000

C -0.393898000 -1.982552000 -0.254802000

C -0.242277000 -2.426948000 1.064465000

C -1.321512000 -3.043788000 1.711685000

H -1.192808000 -3.396683000 2.730183000

H -3.362884000 -3.676665000 1.563866000

C -2.535942000 -3.194539000 1.047942000

C -2.713992000 -2.738639000 -0.267820000

C -1.619731000 -2.137537000 -0.902975000

H -1.724040000 -1.780978000 -1.924603000

O -4.110573000 0.249716000 0.271137000

H -3.733499000 1.107551000 -0.003432000

C -4.549474000 -0.387827000 -0.825042000

O -4.492735000 0.087445000 -1.944104000

C -5.099050000 -1.776616000 -0.526723000

N -6.393250000 -1.889311000 -1.205142000

H -6.242892000 -1.751883000 -2.203677000

H -6.740801000 -2.840216000 -1.097427000

H -5.260766000 -1.857492000 0.552222000

H -3.928874000 -2.773526000 -2.044423000

C -4.054835000 -2.851001000 -0.958587000

H -4.508979000 -3.827872000 -0.754322000

Figure S6. Structure of the (Z)-teriflunomide 2–tyrosine adduct XV; interaction between teriflunomide amino group and tyrosine hydroxyl group.

Cartesian coordinates:

O 3.027103000 4.743986000 -1.926366000

C 1.654664000 6.206890000 -0.680036000

C 2.174914000 4.830835000 -0.932726000

C 1.811859000 3.705381000 -0.197214000

O 3.227914000 2.311081000 -1.476729000

C 2.393091000 2.391076000 -0.544617000

N 1.994950000 1.307775000 0.176047000

F 2.738543000 -4.914292000 -0.569940000

F 3.833911000 -4.662067000 1.287441000

F 4.834305000 -4.347879000 -0.623905000

C 3.675556000 -4.147222000 0.045298000

C 3.284020000 -2.699809000 0.080768000

C 2.483258000 -2.216961000 1.120794000

C 3.666338000 -1.839864000 -0.950490000

C 2.077067000 -0.889336000 1.127759000

C 3.261960000 -0.507008000 -0.956747000

C 2.461049000 -0.022480000 0.089424000

H 3.303593000 3.763846000 -1.974048000

H 0.954809000 6.241660000 0.153689000

H 2.496515000 6.875801000 -0.476183000

H 1.158732000 6.572415000 -1.584749000

H 1.288844000 1.454208000 0.892390000

H 2.186595000 -2.873544000 1.931184000

H 4.289020000 -2.207255000 -1.758714000

H 1.463175000 -0.512491000 1.939057000

H 3.564548000 0.150179000 -1.757255000

H -0.432454000 2.081123000 2.471544000

O -0.447413000 1.110988000 2.368236000

H -1.096237000 -1.358008000 1.945707000

C -1.899078000 -0.658278000 1.737966000

C -1.683151000 0.710539000 1.924201000

C -2.716020000 1.619303000 1.667017000

H -2.551081000 2.682786000 1.813628000

H -4.748761000 1.867216000 1.035625000

C -3.952875000 1.151175000 1.224861000

C -4.191018000 -0.215947000 1.027238000

C -3.141215000 -1.107328000 1.293550000

H -3.297648000 -2.174094000 1.155360000

O -3.648352000 -0.854225000 -2.260600000

H -3.018274000 -1.502933000 -2.623288000

C -4.654593000 -1.541443000 -1.693267000

O -4.713090000 -2.756253000 -1.691667000

C -5.693944000 -0.644466000 -1.034575000

N -7.007310000 -1.082283000 -1.515333000

H -7.140225000 -2.055317000 -1.243411000

H -7.729225000 -0.549881000 -1.033885000

H -5.523796000 0.383897000 -1.366432000

H -5.694259000 -1.745354000 0.823618000

C -5.526327000 -0.707649000 0.513722000

H -6.338134000 -0.107124000 0.939780000

C 0.915479000 3.820889000 0.890667000

N 0.187781000 3.839739000 1.803497000

Figure S7. Structure of the (Z)-teriflunomide 2–tyrosine adduct XVI; interaction between teriflunomide carbonyl group and tyrosine hydroxyl group.


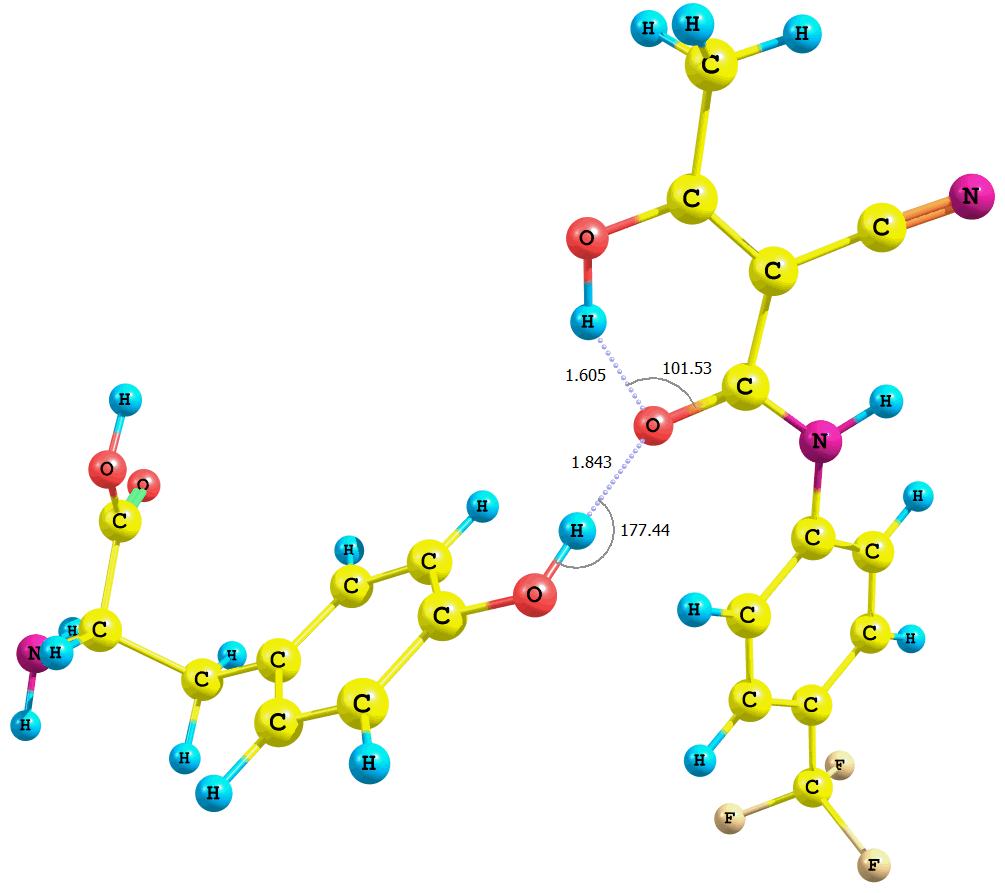


Cartesian coordinates:

O 0.246487000 4.283517000 0.098077000

C 1.671462000 6.146138000 -0.149071000

C 1.494822000 4.667462000 -0.055715000

C 2.534695000 3.746727000 -0.124873000

O 1.075874000 1.898696000 0.154262000

C 2.255394000 2.304191000 -0.023778000

N 3.311513000 1.462015000 -0.146778000

F 4.270802000 -4.677433000 0.931467000

F 4.476752000 -4.684235000 -1.231218000

F 2.504520000 -4.852871000 -0.317928000

C 3.703280000 -4.232058000 -0.218411000

C 3.564685000 -2.737531000 -0.233201000

C 4.547250000 -1.941070000 -0.827013000

C 2.477220000 -2.135126000 0.403927000

C 4.431545000 -0.557003000 -0.793161000

C 2.357213000 -0.749135000 0.450818000

C 3.336109000 0.048810000 -0.158345000

H 0.257028000 3.279204000 0.143042000

H 2.714831000 6.433342000 -0.268536000

H 1.091330000 6.522551000 -0.997518000

H 1.266950000 6.611662000 0.755126000

H 4.198096000 1.900839000 -0.366018000

H 5.394444000 -2.398325000 -1.324950000

H 1.714703000 -2.748311000 0.871580000

H 5.189231000 0.058449000 -1.268601000

H 1.521183000 -0.303872000 0.967078000

H -0.007007000 1.122261000 1.428014000

O -0.555081000 0.675741000 2.100613000

H -1.103360000 0.537500000 -0.528970000

C -1.834415000 0.071768000 0.125009000

C -1.639924000 0.089651000 1.511790000

C -2.579231000 -0.526688000 2.348384000

H -2.417314000 -0.515127000 3.421719000

H -4.414421000 -1.626515000 2.460895000

C -3.698089000 -1.146782000 1.798266000

C -3.915322000 -1.172148000 0.411933000

C -2.962705000 -0.552640000 -0.407874000

H -3.098156000 -0.561198000 -1.486570000

O -5.975856000 1.410026000 -0.284072000

H -5.800561000 2.137536000 -0.907979000

C -6.201125000 0.301034000 -1.009695000

O -6.196740000 0.289608000 -2.225912000

C -6.431952000 -0.936411000 -0.153314000

N -7.630946000 -1.601848000 -0.669993000

H -7.463460000 -1.853075000 -1.643232000

H -7.764691000 -2.477305000 -0.167874000

H -6.624404000 -0.614862000 0.874465000

H -4.967752000 -2.124009000 -1.208628000

C -5.149948000 -1.823742000 -0.170393000

H -5.394825000 -2.733270000 0.390399000

C 3.871961000 4.190289000 -0.303121000

N 4.988644000 4.495296000 -0.449093000

Figure S8. Structure of the (Z)-teriflunomide 2−tyrosine adduct XVII; interaction between teriflunomide nitrile group and tyrosine amine group.


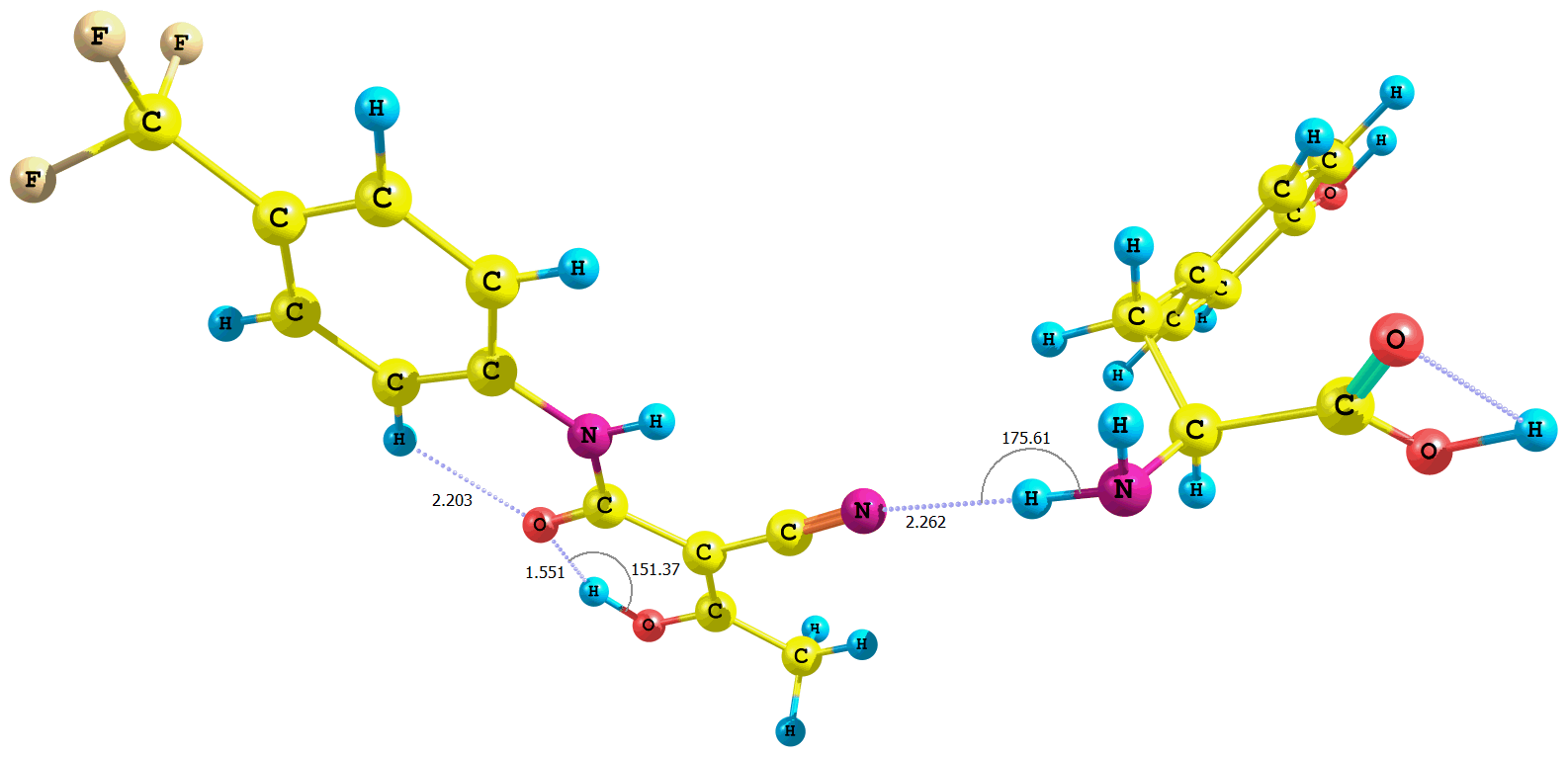


Cartesian coordinates:

O 2.008998000 5.102358000 -0.626800000

C -0.256197000 5.091087000 0.041548000

C 1.031676000 4.362616000 -0.154829000

C 1.222822000 3.012944000 0.124062000

O 3.486907000 3.097639000 -0.565271000

C 2.545507000 2.402887000 -0.117470000

N 2.677869000 1.077827000 0.165922000

F 7.174383000 -3.000628000 -1.573087000

F 6.948865000 -3.600530000 0.502175000

F 8.275342000 -1.952120000 -0.023888000

C 7.080190000 -2.522943000 -0.305249000

C 5.944142000 -1.555988000 -0.149376000

C 4.689730000 -2.001788000 0.276881000

C 6.121468000 -0.210809000 -0.481568000

C 3.630166000 -1.109263000 0.369586000

C 5.066509000 0.693078000 -0.392727000

C 3.806502000 0.244294000 0.035929000

H 2.814288000 4.487359000 -0.711106000

H -1.039533000 4.450080000 0.443264000

H -0.091343000 5.933128000 0.720949000

H -0.582618000 5.504816000 -0.917852000

H 1.847552000 0.615301000 0.515702000

H 4.540698000 -3.042111000 0.542522000

H 7.093888000 0.142890000 -0.806767000

H 2.659246000 -1.461729000 0.704685000

H 5.213222000 1.731546000 -0.647241000

C 0.160896000 2.226523000 0.636088000

N -0.673909000 1.529787000 1.058778000

H -8.572187000 -1.972979000 -3.234667000

O -8.114146000 -1.121701000 -3.278484000

C -7.081883000 -1.115443000 -2.381524000

C -6.786149000 -2.206689000 -1.558062000

H -7.387059000 -3.110868000 -1.616257000

C -5.714702000 -2.132103000 -0.667091000

H -5.494774000 -2.988783000 -0.035320000

C -4.918258000 -0.983116000 -0.573680000

C -5.231900000 0.098416000 -1.411798000

H -4.627188000 1.000982000 -1.368856000

C -6.297488000 0.042034000 -2.306349000

H -6.530043000 0.881119000 -2.954402000

C -3.779051000 -0.895163000 0.417459000

H -2.947133000 -0.325113000 -0.010695000

H -3.399894000 -1.896814000 0.650413000

C -4.138263000 -0.203006000 1.767921000

H -4.527951000 0.797403000 1.556932000

N -3.013945000 -0.080592000 2.697987000

H -2.253558000 0.412841000 2.230446000

H -2.670619000 -1.016504000 2.908709000

C -5.226608000 -1.002181000 2.471827000

O -5.037973000 -2.078495000 3.006447000

O -6.432587000 -0.407824000 2.427161000

H -7.066658000 -1.000378000 2.870046000

Figure S9. Structure of the (E)-teriflunomide 2–water–arginine adduct XVIII; interaction of arginine carboxyl group with teriflunomide hydroxyl group via water molecule.

Cartesian coordinates:

O -2.012292000 -2.459698000 1.450615000

N -1.664136000 -2.636414000 -2.187172000

C -0.173059000 -1.373437000 2.382059000

C -0.865998000 -1.890892000 1.151812000

C -0.359800000 -1.760375000 -0.131119000

C -1.088837000 -2.233082000 -1.254884000

O 0.851877000 -0.143227000 -1.398121000

C 0.878436000 -1.003101000 -0.502790000

N 2.012731000 -1.350930000 0.168412000

F 8.080591000 0.065660000 -0.836395000

F 7.945796000 0.604906000 1.262611000

F 7.386930000 2.045592000 -0.275564000

C 7.323367000 0.738274000 0.068212000

C 5.911641000 0.232072000 0.108695000

C 5.569696000 -0.822591000 0.960874000

C 4.946325000 0.771195000 -0.744112000

C 4.275411000 -1.324524000 0.959879000

C 3.645786000 0.273304000 -0.756269000

C 3.302761000 -0.782526000 0.102887000

H -2.638192000 -2.767446000 0.694526000

H 0.278384000 -2.208315000 2.930022000

H 0.598660000 -0.637535000 2.165624000

H -0.925772000 -0.926940000 3.035817000

H 1.928076000 -2.151026000 0.781187000

H 6.309580000 -1.245459000 1.630980000

H 5.205153000 1.590697000 -1.405539000

H 4.014202000 -2.137980000 1.630044000

H 2.905657000 0.688757000 -1.422993000

O -3.802651000 -3.257601000 -0.178435000

H -3.410709000 -3.469474000 -1.039152000

H -4.422016000 -2.499810000 -0.372208000

O -4.682923000 0.748766000 -1.612542000

O -5.486728000 -1.275777000 -0.828974000

N -6.290099000 -0.106844000 1.578701000

N -2.996660000 2.516600000 -0.486106000

N -1.871723000 1.017550000 -1.805814000

N -0.674579000 2.236575000 -0.230448000

C -5.710513000 2.170236000 0.822391000

C -4.358179000 2.303062000 1.569950000

C -6.099239000 0.743401000 0.387115000

C -3.299126000 3.121920000 0.813874000

C -5.074527000 0.101374000 -0.546296000

C -1.844645000 1.931486000 -0.822203000

H -5.723502000 2.812096000 -0.066786000

H -6.518116000 2.545929000 1.462011000

H -4.507312000 2.791347000 2.539579000

H -3.960144000 1.304823000 1.778203000

H -7.045555000 0.844398000 -0.190857000

H -3.656462000 4.146092000 0.654356000

H -2.387062000 3.188516000 1.412861000

H -6.494283000 -1.049355000 1.251151000

H -7.132632000 0.200928000 2.063163000

H -3.800743000 2.046189000 -0.961770000

H -2.795851000 0.607116000 -1.988072000

H -1.020445000 0.498193000 -1.995841000

H 0.163260000 1.773413000 -0.557224000

H -0.568593000 3.103718000 0.271167000

H -5.539453000 -1.304553000 -1.799939000

Figure S10. Structure of the (E)-teriflunomide 2–water–arginine adduct XIX; interaction of arginine carboxyl group with NH group of teriflunomide via water molecule.


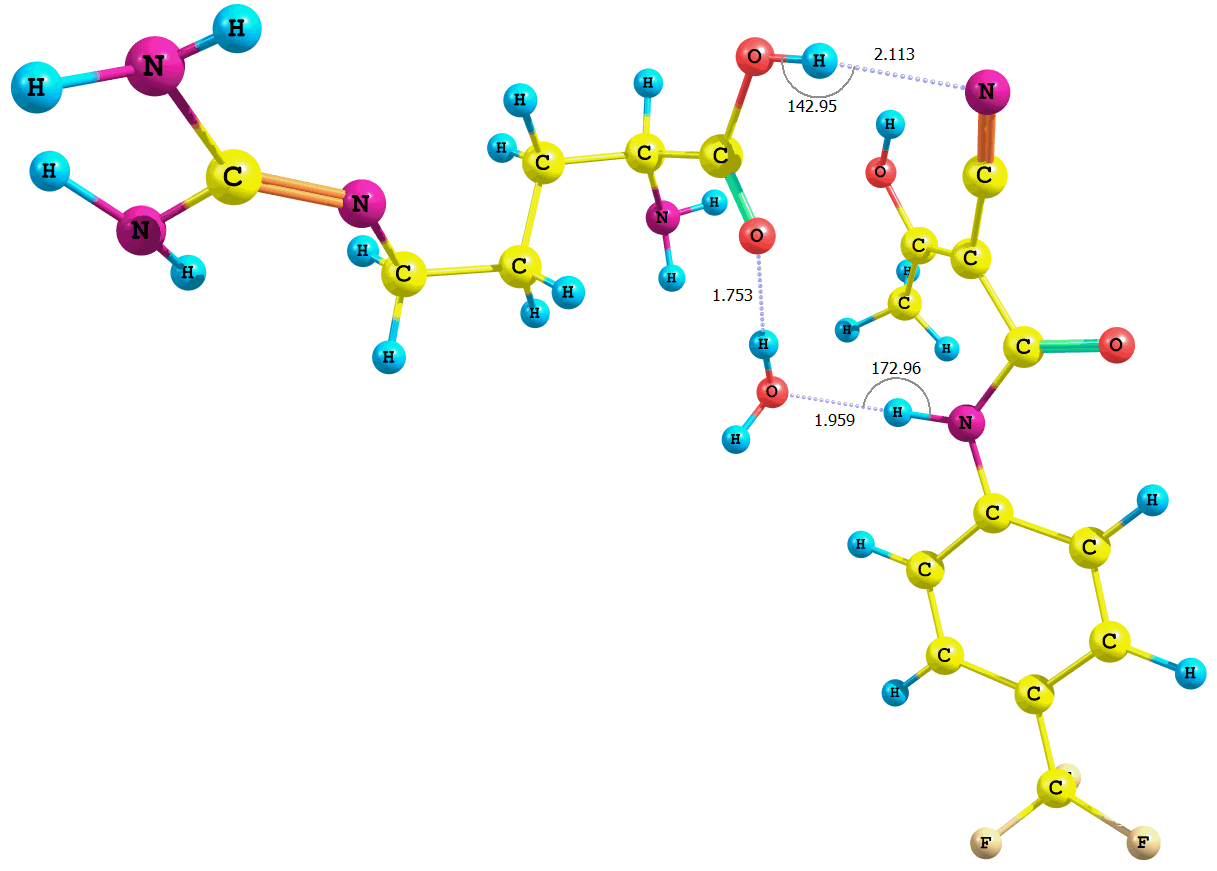


Cartesian coordinates:

O 1.008263000 -3.814601000 -0.961448000

N 1.108590000 -3.381111000 2.368741000

C -0.924509000 -3.008796000 -2.028791000

C -0.175742000 -3.202582000 -0.749039000

C -0.589930000 -2.830673000 0.500512000

C 0.282024000 -3.132383000 1.582680000

O -2.606670000 -2.607281000 1.739108000

C -1.901154000 -2.165588000 0.842760000

N -2.170056000 -1.050204000 0.087387000

F -7.639861000 1.928140000 -0.924131000

F -6.428561000 3.626987000 -0.323677000

F -7.416028000 2.398258000 1.184634000

C -6.752355000 2.354769000 0.007877000

C -5.540777000 1.471285000 0.076529000

C -4.463009000 1.695350000 -0.785186000

C -5.500935000 0.389124000 0.957698000

C -3.365142000 0.846875000 -0.760676000

C -4.403760000 -0.467151000 0.994902000

C -3.321093000 -0.241838000 0.128691000

H 1.480402000 -3.944705000 -0.119998000

H -1.984682000 -2.830491000 -1.856086000

H -0.511301000 -2.147466000 -2.565064000

H -0.795765000 -3.896854000 -2.652927000

H -1.403019000 -0.691612000 -0.488461000

H -4.482913000 2.536529000 -1.469599000

H -6.331692000 0.217302000 1.633435000

H -2.530985000 1.018385000 -1.434253000

H -4.368408000 -1.295804000 1.686770000

O 0.158984000 0.077993000 -1.385271000

H 0.163721000 1.043437000 -1.408770000

H 0.850326000 -0.162416000 -0.727876000

H 2.696028000 -2.153490000 -2.414021000

H 2.448890000 -0.560062000 -2.230991000

N 3.179263000 -1.266622000 -2.287418000

C 3.955978000 -1.305125000 -1.049625000

H 4.590142000 -2.196458000 -1.080987000

O 3.551251000 -2.318463000 1.085647000

H 2.901161000 -2.408900000 1.816859000

C 3.079011000 -1.439957000 0.195829000

O 2.029118000 -0.834643000 0.382076000

C 4.889468000 -0.080375000 -0.923585000

H 5.595391000 -0.144610000 -1.760355000

H 5.470179000 -0.162936000 0.001534000

C 4.198121000 1.286888000 -0.947823000

H 3.686891000 1.432329000 -1.907864000

H 5.942297000 2.402791000 -1.554584000

H 4.653083000 3.390527000 -0.856639000

H 3.442907000 1.343414000 -0.155883000

N 5.802680000 2.341760000 0.574970000

C 6.823811000 3.068436000 0.843955000

N 7.507196000 2.893454000 2.050389000

H 6.993731000 2.266526000 2.657608000

H 7.740568000 3.762150000 2.518063000

N 7.378511000 4.073086000 0.054773000

H 7.061867000 4.090256000 -0.903908000

H 8.382336000 4.168349000 0.136693000

C 5.192997000 2.436200000 -0.739530000

Figure S11. Structure of the (E)-teriflunomide 2–water–arginine adduct XX; interaction of arginine carboxyl group with teriflunomide carbonyl via water molecule.

**
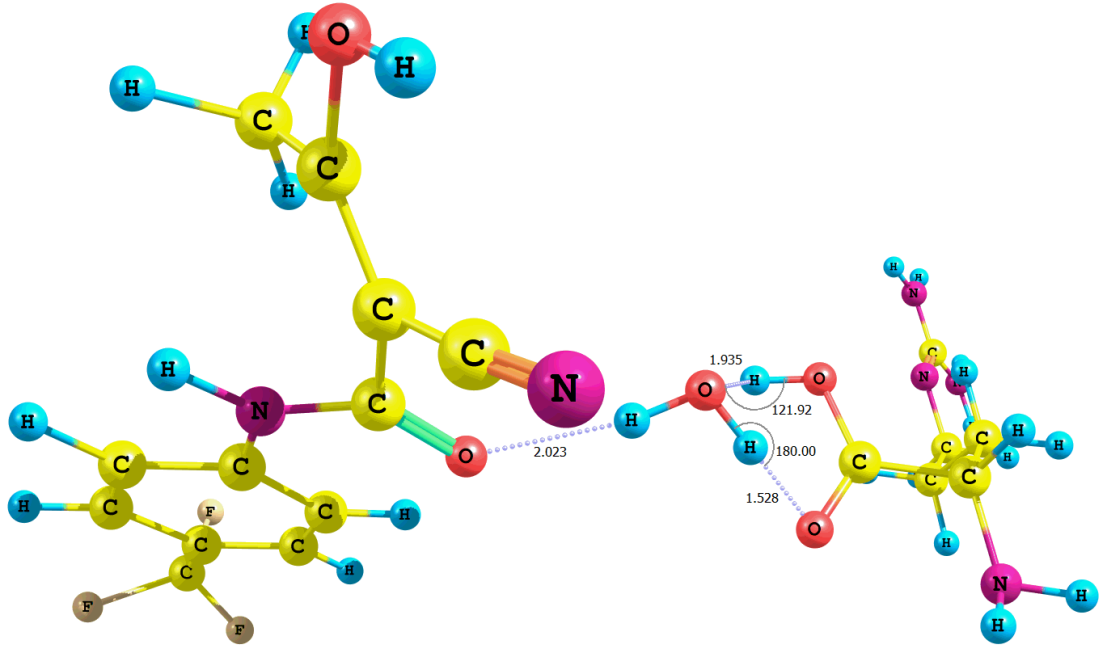
**

Cartesian coordinates:

O 2.395200000 5.290559000 1.678833000

N 0.988884000 4.816372000 -1.343601000

C 3.435988000 3.422159000 2.634809000

C 2.709223000 4.004771000 1.460760000

C 2.348175000 3.353838000 0.308959000

C 1.585232000 4.108193000 -0.634421000

O 1.713748000 1.207763000 -0.497101000

C 2.595512000 1.911655000 -0.018717000

N 3.878666000 1.473389000 0.225378000

F 6.195591000 -4.338659000 0.970714000

F 7.583643000 -3.617902000 -0.536485000

F 5.672664000 -4.485446000 -1.132467000

C 6.271168000 -3.692043000 -0.217601000

C 5.635162000 -2.332217000 -0.154741000

C 6.391503000 -1.220524000 0.225311000

C 4.273044000 -2.178793000 -0.422140000

C 5.786641000 0.023650000 0.337577000

C 3.655000000 -0.935861000 -0.315060000

C 4.415958000 0.178439000 0.072576000

H 1.903553000 5.652600000 0.918350000

H 3.442356000 2.333413000 2.621552000

H 4.468926000 3.789356000 2.665673000

H 2.947984000 3.770675000 3.548854000

H 4.547283000 2.192422000 0.460719000

H 7.451886000 -1.327465000 0.424703000

H 3.686339000 -3.037593000 -0.729166000

H 6.381729000 0.885761000 0.630203000

H 2.605259000 -0.817015000 -0.539380000

O -1.158924000 1.983601000 -0.285955000

H -1.739134000 1.525500000 -0.938187000

H -0.251360000 1.685366000 -0.458812000

O -3.422646000 0.813526000 0.194004000

O -2.638531000 0.815389000 -1.949227000

N -5.056933000 0.862696000 -3.076567000

N -7.168272000 -1.647288000 1.034044000

N -8.007511000 -1.998506000 3.142963000

N -9.065336000 -3.039943000 1.356420000

C -6.143984000 0.365732000 -0.920809000

C -6.087628000 -1.153204000 -1.111982000

C -5.018725000 1.123410000 -1.643540000

C -7.222786000 -1.887887000 -0.392324000

C -3.600541000 0.841095000 -1.141860000

C -8.047081000 -2.211535000 1.782496000

H -6.119689000 0.593899000 0.146671000

H -7.102933000 0.752673000 -1.296882000

H -6.104825000 -1.395245000 -2.179362000

H -5.135936000 -1.527452000 -0.713064000

H -5.117880000 2.199324000 -1.379659000

H -8.185878000 -1.554644000 -0.830150000

H -7.140578000 -2.964337000 -0.637618000

H -4.350336000 1.416900000 -3.551115000

H -5.968186000 1.142053000 -3.431150000

H -2.456561000 0.686659000 0.328654000

H -7.274790000 -1.399126000 3.479984000

H -8.657944000 -2.407105000 3.787839000

H -9.726024000 -3.457683000 1.987411000

H -9.158754000 -3.239892000 0.377421000

Figure S12. Structure of the (Z)-teriflunomide 2−water–arginine adduct XXI; interaction of arginine carboxyl group with teriflunomide hydroxyl group via water molecule.

Cartesian coordinates:

C 1.697316000 1.880927000 2.051129000

C 1.193640000 2.492847000 0.759269000

C -0.034080000 2.069312000 -0.048434000

O -0.899310000 0.002602000 -1.019084000

C -1.105764000 1.056272000 -0.349871000

N -2.339452000 1.480700000 0.056469000

F -8.120959000 -0.828232000 -1.089296000

F -8.114629000 -0.945663000 1.078518000

F -7.274876000 -2.571111000 -0.110723000

C -7.369408000 -1.220719000 -0.021569000

C -6.032579000 -0.557589000 0.016979000

C -5.869076000 0.673810000 0.666064000

C -4.939328000 -1.122453000 -0.647911000

C -4.638538000 1.310365000 0.652413000

C -3.702614000 -0.487106000 -0.671738000

C -3.515623000 0.754073000 -0.012429000

H 1.401024000 2.438812000 2.943801000

H 1.310566000 0.861885000 2.152603000

H 2.796090000 1.840844000 2.024450000

H -2.242784000 2.751057000 0.548989000

H -6.704651000 1.125933000 1.190571000

H -5.053851000 -2.075881000 -1.154331000

H -4.515587000 2.256126000 1.172187000

H -2.870648000 -0.930958000 -1.197330000

H 6.290536000 1.499856000 0.075651000

O 4.985325000 -0.605229000 -1.646735000

O 5.754426000 1.215457000 -0.677344000

N 7.304404000 -0.216963000 1.113058000

N 3.109957000 -2.391356000 -0.186003000

N 1.749345000 -0.859854000 -1.267945000

N 0.888642000 -2.068398000 0.488164000

C 6.106904000 -2.275312000 0.433604000

C 4.948950000 -2.124009000 1.454669000

C 6.759035000 -0.959711000 -0.031453000

C 3.694367000 -2.915171000 1.057023000

C 5.761067000 -0.128636000 -0.840010000

C 1.927313000 -1.766691000 -0.302089000

H 5.787451000 -2.822610000 -0.458151000

H 6.904447000 -2.885723000 0.869627000

H 5.272953000 -2.487599000 2.434485000

H 4.683776000 -1.071112000 1.598418000

H 7.539283000 -1.232320000 -0.764284000

H 3.946145000 -3.971368000 0.914444000

H 2.943466000 -2.861856000 1.847601000

H 7.885370000 0.553491000 0.781172000

H 7.977728000 -0.815960000 1.587832000

H 3.786742000 -2.104931000 -0.888405000

H 2.542235000 -0.317009000 -1.585194000

H 0.803881000 -0.463151000 -1.352765000

H 0.022597000 -1.560746000 0.337683000

H 0.861840000 -2.930967000 1.008605000

C -0.684382000 3.188905000 0.331364000

N -1.559520000 3.946873000 0.718451000

O 2.104083000 3.287925000 0.121229000

H 2.210216000 2.996609000 -0.826926000

O 3.229656000 2.223595000 -1.933389000

H 4.035556000 1.893808000 -1.501938000

H 3.329515000 1.972325000 -2.860098000

Figure S13. Structure of the (Z)-teriflunomide 2−water–arginine adduct XXII; interaction of arginine carboxyl group with teriflunomide NH group via water molecule.


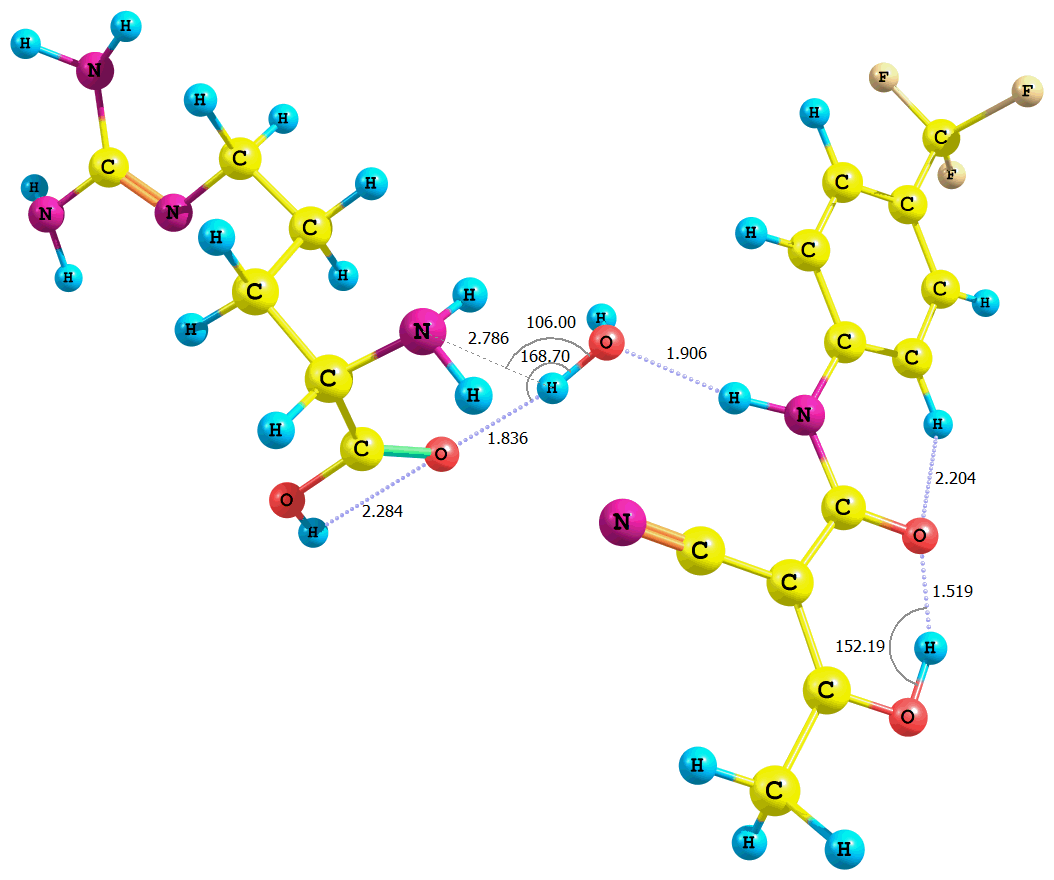


Cartesian coordinates:

O -3.983965000 4.876184000 -0.600190000

C -1.992918000 5.974363000 0.033833000

C -2.760645000 4.706735000 -0.152583000

C -2.259815000 3.434449000 0.113419000

C -0.965399000 3.301612000 0.677451000

O -4.316367000 2.431586000 -0.475820000

C -3.121728000 2.257692000 -0.124791000

N -2.586905000 1.022913000 0.056435000

F -5.941673000 -4.254914000 1.007049000

F -4.184983000 -5.125388000 0.070982000

F -5.806877000 -4.342314000 -1.157118000

C -5.081452000 -4.117658000 -0.035453000

C -4.423350000 -2.770432000 -0.052018000

C -3.169159000 -2.584918000 0.536192000

C -5.088357000 -1.677667000 -0.613675000

C -2.592280000 -1.321494000 0.562589000

C -4.517015000 -0.408408000 -0.597950000

C -3.257487000 -0.218978000 -0.003561000

H -4.392752000 3.939861000 -0.641868000

H -0.980358000 5.799456000 0.393464000

H -1.951660000 6.508962000 -0.920443000

H -2.525575000 6.615478000 0.743277000

H -1.572542000 0.939789000 0.205018000

H -2.641760000 -3.425216000 0.973384000

H -6.057298000 -1.815807000 -1.081552000

H -1.621614000 -1.179648000 1.024520000

H -5.035434000 0.427054000 -1.042359000

O 0.173271000 0.189514000 0.053372000

H 0.165071000 -0.551613000 -0.567661000

H 0.857687000 0.795065000 -0.301455000

H 1.974511000 2.022896000 1.780441000

H 2.121061000 0.458972000 1.413711000

N 2.639464000 1.250038000 1.790754000

C 3.771003000 1.565431000 0.931019000

H 4.241152000 2.481955000 1.307788000

O 4.387122000 2.458599000 -1.197221000

H 4.083839000 2.606045000 -2.110867000

C 3.387740000 1.871347000 -0.520298000

O 2.314526000 1.624250000 -1.050452000

C 4.846963000 0.454285000 0.968571000

H 5.178251000 0.368756000 2.010173000

H 5.710088000 0.772622000 0.377233000

C 4.378648000 -0.916994000 0.467798000

H 3.564589000 -1.293532000 1.098952000

H 5.891208000 -2.054141000 1.495738000

H 5.076235000 -2.936837000 0.204051000

H 3.981247000 -0.833654000 -0.552324000

N 6.554208000 -1.576276000 -0.475239000

C 7.641295000 -2.266362000 -0.488103000

N 8.708869000 -1.860977000 -1.291118000

H 8.414481000 -1.168452000 -1.968665000

H 9.211145000 -2.621821000 -1.734649000

N 7.899267000 -3.426290000 0.227589000

H 7.293172000 -3.598456000 1.017344000

H 8.872534000 -3.598598000 0.444586000

C 5.507500000 -1.955561000 0.462946000

N 0.088887000 3.196118000 1.165024000

Figure S14. Structure of the (Z)-teriflunomide 2−water–arginine adduct XXIII; interaction of arginine carboxyl group with teriflunomide carbonyl group via water molecule.


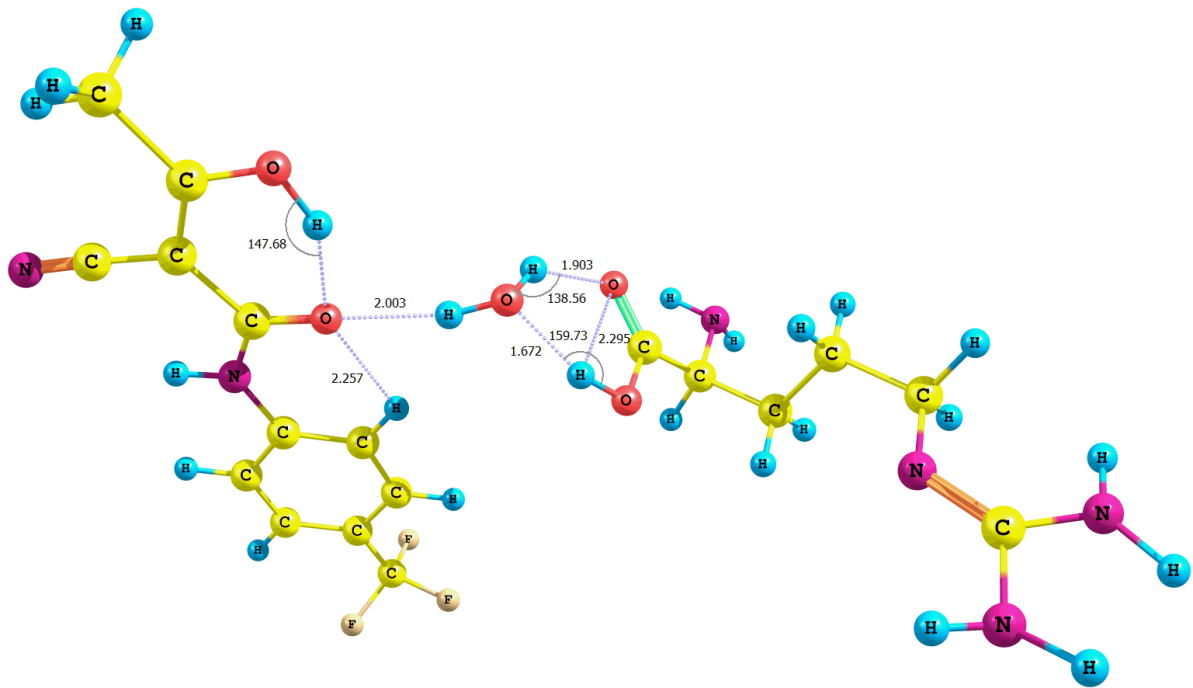


Cartesian coordinates:

O -2.598685000 -4.462393000 -0.041418000

N -7.044903000 -2.767471000 -0.471560000

C -4.671434000 -5.566198000 -0.234163000

C -3.896426000 -4.295579000 -0.182644000

C -4.480245000 -3.038523000 -0.254844000

C -5.892518000 -2.919123000 -0.368974000

O -2.397560000 -1.904375000 -0.146341000

C -3.645847000 -1.819273000 -0.219875000

N -4.298916000 -0.628314000 -0.290778000

F -2.667759000 5.319213000 1.054181000

F -3.297539000 5.591446000 -1.005160000

F -1.255623000 4.970002000 -0.560220000

C -2.549097000 4.804477000 -0.197338000

C -2.976153000 3.367270000 -0.259196000

C -4.319658000 3.045477000 -0.474660000

C -2.048539000 2.345465000 -0.045067000

C -4.722454000 1.716924000 -0.480507000

C -2.441100000 1.009006000 -0.052333000

C -3.789707000 0.685853000 -0.272062000

H -2.183837000 -3.548186000 -0.016997000

H -5.745283000 -5.393781000 -0.171859000

H -4.445579000 -6.079655000 -1.175483000

H -4.352672000 -6.218922000 0.583484000

H -5.305582000 -0.687759000 -0.387611000

H -5.049526000 3.829197000 -0.645603000

H -1.005291000 2.589034000 0.123424000

H -5.766606000 1.473441000 -0.653409000

H -1.713179000 0.230633000 0.112069000

O 0.519657000 -2.323006000 0.059172000

H 0.741054000 -2.243346000 1.013336000

H -0.402742000 -2.040374000 -0.028829000

O 2.330728000 -0.405951000 0.078442000

O 1.900114000 -1.226972000 2.128553000

N 3.741687000 0.348473000 3.337821000

N 6.625779000 -0.528396000 -1.049311000

N 7.403569000 -0.147313000 -3.193285000

N 8.961170000 -0.484901000 -1.508978000

C 4.835977000 0.566549000 1.121499000

C 5.605170000 -0.757423000 1.178370000

C 3.509022000 0.579662000 1.910315000

C 6.906567000 -0.717083000 0.366736000

C 2.513161000 -0.452810000 1.395727000

C 7.633651000 -0.408736000 -1.853929000

H 4.631710000 0.822166000 0.078176000

H 5.464472000 1.372874000 1.525135000

H 5.828412000 -1.009319000 2.221462000

H 4.979190000 -1.567011000 0.778886000

H 3.021789000 1.551419000 1.701219000

H 7.545804000 0.090382000 0.773757000

H 7.457368000 -1.658527000 0.551024000

H 2.845788000 0.236374000 3.807497000

H 4.175529000 1.179435000 3.734228000

H 1.633345000 -1.093093000 -0.146569000

H 6.452333000 -0.326868000 -3.475093000

H 8.095394000 -0.463463000 -3.856511000

H 9.688603000 -0.236391000 -2.160145000

H 9.222846000 -0.572781000 -0.541741000

Figure S15. Structure of the model 1 subjected for ONIOM calculations (PM6:UFF).


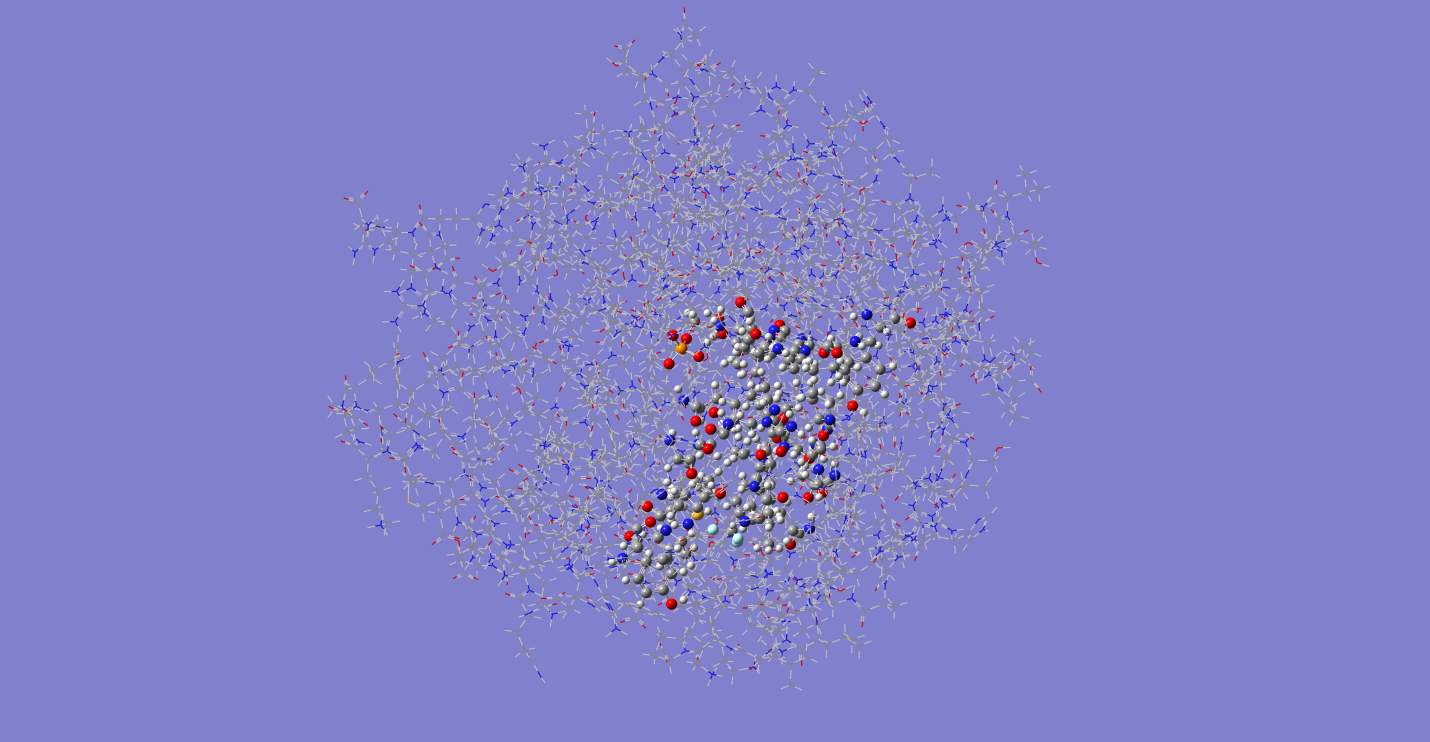


**Input file**:

%nproc=8

%mem=9000mb

# ONIOM(PM6:UFF)=EmbedCharge Opt

test teriflunomide-3G0U

2 1 -1 1

C 0 31.79600 9.48600 2.34800 H

C 0 32.36700 10.81100 2.21900 H

C 0 32.10100 11.65200 1.10200 H

C 0 32.72800 12.87200 1.09500 H

C 0 33.18700 11.28900 3.27300 H

C 0 33.81600 12.53800 3.26200 H

C 0 33.59300 13.34800 2.12700 H

N 0 34.17900 14.57900 1.99300 H

C 0 34.31700 15.39800 3.01800 H

O 0 33.93500 15.11700 4.14600 H

C 0 34.83700 16.75800 2.78600 H

C 0 34.88700 17.65300 3.90100 H

N 0 34.95700 18.44100 4.75600 H

C 0 35.22700 17.20600 1.38500 H

C 0 35.81400 18.51000 1.14400 H

O 0 35.14300 16.38200 0.33800 H

H 0 35.42700 16.83600 -0.44600 H

H 0 34.50900 14.85300 1.08100 H

H 0 36.01500 18.62600 0.08000 H

H 0 35.12200 19.28500 1.46900 H

H 0 36.74500 18.59800 1.70100 H

F 0 32.23500 8.90300 3.54200 H

F 0 30.39900 9.57900 2.36000 H

F 0 32.20200 8.69600 1.26600 H

H 0 31.43100 11.31400 0.31300 H

H 0 32.58100 13.55500 0.26000 H

H 0 33.35600 10.67200 4.15400 H

H 0 34.44000 12.84100 4.10200 H

N 0 34.90800 24.32700 -8.65800 H

C 0 35.71800 24.29200 -9.75100 H

O 0 35.32100 24.72000 -10.80300 H

N 0 36.98900 23.72700 -9.67300 H

C 0 37.49500 23.22500 -8.53800 H

O 0 38.61000 22.68600 -8.55800 H

C 0 36.72200 23.30400 -7.38000 H

N 0 37.21900 22.81800 -6.20200 H

C 0 36.41100 22.77700 -5.05000 H

C 0 36.89900 22.21700 -3.83500 H

C 0 36.04500 22.16300 -2.75000 H

C 0 36.38300 21.50300 -1.50400 H

C 0 34.71600 22.63100 -2.86900 H

C 0 33.78900 22.41500 -1.77500 H

C 0 34.22700 23.19300 -4.05700 H

C 0 35.07700 23.23300 -5.16800 H

N 0 34.59100 23.80000 -6.37200 H

C 0 35.38600 23.80100 -7.47100 H

C 0 33.24800 24.46200 -6.38300 H

C 0 32.11100 23.40400 -6.56300 H

O 0 32.38200 22.83300 -7.85500 H

C 0 30.74800 24.14800 -6.64300 H

O 0 30.81300 25.15700 -7.72600 H

C 0 30.50700 24.87800 -5.30400 H

O 0 31.08700 24.11200 -4.21500 H

C 0 29.00600 25.02700 -5.00000 H

O 0 28.40800 23.72000 -4.97500 H

P 0 28.05500 22.98100 -3.56400 H

O 0 29.32500 22.41900 -2.96700 H

O 0 27.12300 21.86800 -3.84400 H

O 0 27.41400 23.96700 -2.66000 H

H 0 37.53400 23.70600 -10.52100 H

H 0 37.91600 21.83700 -3.74700 H

H 0 33.21100 23.58300 -4.09700 H

H 0 35.55200 21.59600 -0.80700 H

H 0 36.58100 20.44900 -1.69400 H

H 0 37.27000 21.96700 -1.07600 H

H 0 32.81800 22.83600 -2.03400 H

H 0 33.68400 21.34600 -1.59400 H

H 0 34.16700 22.90000 -0.87700 H

H 0 33.09400 24.98700 -5.44100 H

H 0 33.20000 25.17300 -7.20700 H

H 0 32.11200 22.72200 -5.71400 H

H 0 31.73100 22.17000 -8.05100 H

H 0 29.94500 23.43300 -6.81700 H

H 0 29.98300 25.61600 -7.78100 H

H 0 30.96500 25.86400 -5.36400 H

H 0 30.40900 23.89100 -3.58700 H

H 0 28.53000 25.62900 -5.77300 H

H 0 28.87800 25.51100 -4.03300 H

N 0 23.69000 7.45000 7.79000 H

H 0 22.95000 7.18000 7.17000 H

C 0 24.81000 8.26000 7.27000 H

H 0 25.66000 7.80000 7.54000 H

C 0 24.74000 8.36000 5.74000 H

H 0 25.18000 9.21000 5.45000 H

H 0 23.78000 8.37000 5.46000 H

C 0 25.43000 7.21000 5.05000 H

C 0 24.83000 5.96000 4.96000 H

H 0 23.93000 5.83000 5.38000 H

C 0 25.44000 4.92000 4.32000 H

H 0 24.99000 4.03000 4.25000 H

C 0 26.69000 5.11000 3.77000 H

O 0 27.32000 4.07000 3.17000 H

H 0 28.21000 4.38000 2.83000 H

C 0 27.31000 6.32000 3.83000 H

H 0 28.20000 6.45000 3.40000 H

C 0 26.69000 7.37000 4.49000 H

H 0 27.15000 8.25000 4.56000 H

C 0 24.76000 9.64000 7.91000 H

O 0 25.76000 10.15000 8.40000 H

N 0 28.54000 10.00000 9.23000 H

H 0 27.55000 9.92000 9.11000 H

C 0 29.23000 11.23000 8.78000 H

H 0 30.00000 10.94000 8.21000 H

C 0 28.33000 12.13000 7.87000 H

H 0 28.86000 12.93000 7.59000 H

H 0 27.54000 12.42000 8.40000 H

C 0 27.84000 11.35000 6.57000 H

H 0 27.29000 12.00000 6.05000 H

H 0 27.26000 10.60000 6.90000 H

S 0 29.25000 10.72000 5.59000 H

C 0 29.98000 12.26000 4.94000 H

H 0 30.78000 12.04000 4.37000 H

H 0 29.31000 12.74000 4.38000 H

H 0 30.27000 12.85000 5.70000 H

C 0 29.75000 11.99000 10.02000 H

O 0 30.93000 12.27000 10.10000 H

N 0 32.74000 10.18000 11.32000 H

H 0 31.85000 9.91000 10.97000 H

C 0 33.83000 10.42000 10.39000 H

H 0 34.52000 9.73000 10.58000 H

C 0 33.30000 10.35000 8.97000 H

H 0 32.92000 11.23000 8.67000 H

H 0 32.61000 9.64000 8.87000 H

C 0 34.53000 10.01000 8.17000 H

H 0 35.39000 10.29000 8.61000 H

C 0 34.50000 8.48000 8.01000 H

H 0 35.30000 8.19000 7.48000 H

H 0 34.52000 8.05000 8.91000 H

H 0 33.67000 8.21000 7.53000 H

C 0 34.48000 10.78000 6.86000 H

H 0 35.29000 10.56000 6.32000 H

H 0 33.66000 10.51000 6.36000 H

H 0 34.46000 11.76000 7.05000 H

C 0 34.36000 11.80000 10.62000 H

O 0 35.56000 11.97000 10.69000 H

N 0 33.47000 12.78000 10.73000 H

H 0 32.50000 12.55000 10.72000 H

C 0 33.87000 14.22000 10.86000 H

H 0 34.49000 14.39000 10.09000 H

C 0 32.65000 15.19000 10.68000 H

H 0 32.95000 16.10000 10.98000 H

H 0 31.92000 14.86000 11.27000 H

C 0 32.06000 15.34000 9.18000 H

H 0 31.48000 14.54000 9.01000 H

H 0 32.84000 15.33000 8.55000 H

C 0 31.18000 16.67000 8.86000 H

O 0 30.22000 17.02000 9.63000 H

N 0 31.51000 17.40000 7.71000 H

H 0 32.27000 17.09000 7.13000 H

H 0 31.00000 18.22000 7.47000 H

C 0 34.64000 14.45000 12.20000 H

O 0 35.44000 15.37000 12.32000 H

N 0 39.04000 17.72000 7.56000 H

C 0 39.01000 19.00000 8.31000 H

H 0 39.84000 19.53000 8.12000 H

H 0 38.94000 18.83000 9.29000 H

C 0 37.78000 19.68000 7.78000 H

H 0 37.86000 20.67000 7.89000 H

H 0 36.97000 19.35000 8.26000 H

C 0 37.77000 19.26000 6.27000 H

H 0 38.41000 19.81000 5.74000 H

H 0 36.85000 19.33000 5.88000 H

C 0 38.24000 17.77000 6.33000 H

H 0 37.48000 17.12000 6.37000 H

C 0 39.05000 17.35000 5.09000 H

O 0 38.57000 16.55000 4.29000 H

N 0 39.41000 13.61000 5.21000 H

H 0 39.43000 14.46000 5.74000 H

C 0 38.16000 12.92000 4.98000 H

H 0 38.26000 11.98000 5.32000 H

C 0 37.01000 13.64000 5.79000 H

H 0 36.15000 13.17000 5.63000 H

H 0 37.23000 13.63000 6.76000 H

H 0 36.94000 14.59000 5.48000 H

C 0 37.90000 12.92000 3.40000 H

O 0 37.44000 11.96000 2.81000 H

N 0 38.21000 14.04000 2.75000 H

H 0 38.59000 14.82000 3.24000 H

C 0 37.99000 14.13000 1.29000 H

H 0 37.03000 13.93000 1.08000 H

C 0 38.37000 15.54000 0.89000 H

H 0 39.30000 15.73000 1.21000 H

H 0 37.73000 16.18000 1.34000 H

C 0 38.32000 15.81000 -0.58000 H

N 0 38.85000 16.99000 -1.12000 H

C 0 38.62000 17.00000 -2.43000 H

H 0 38.88000 17.74000 -3.06000 H

N 0 38.00000 15.87000 -2.77000 H

H 0 37.76000 15.59000 -3.70000 H

C 0 37.74000 15.14000 -1.61000 H

H 0 37.22000 14.29000 -1.55000 H

C 0 38.92000 13.13000 0.56000 H

O 0 38.50000 12.35000 -0.29000 H

N 0 37.54000 9.67000 1.03000 H

H 0 37.69000 10.53000 1.53000 H

C 0 36.32000 9.52000 0.23000 H

H 0 35.87000 8.72000 0.62000 H

C 0 35.38000 10.82000 0.31000 H

H 0 34.57000 10.67000 -0.26000 H

H 0 35.11000 10.97000 1.26000 H

H 0 35.89000 11.61000 -0.03000 H

C 0 36.74000 9.30000 -1.27000 H

O 0 36.12000 8.51000 -1.97000 H

N 0 35.52000 17.68000 -6.25000 H

H 0 34.77000 17.88000 -6.89000 H

C 0 35.58000 16.44000 -5.44000 H

H 0 35.81000 16.71000 -4.51000 H

C 0 34.23000 15.74000 -5.44000 H

H 0 33.91000 15.65000 -6.38000 H

H 0 33.58000 16.30000 -4.92000 H

C 0 34.29000 14.38000 -4.82000 H

C 0 34.44000 14.23000 -3.42000 H

H 0 34.44000 15.05000 -2.83000 H

C 0 34.61000 12.93000 -2.81000 H

H 0 34.71000 12.84000 -1.82000 H

C 0 34.64000 11.82000 -3.61000 H

H 0 34.75000 10.92000 -3.19000 H

C 0 34.51000 11.91000 -5.03000 H

H 0 34.53000 11.09000 -5.60000 H

C 0 34.34000 13.24000 -5.62000 H

H 0 34.26000 13.33000 -6.61000 H

C 0 36.64000 15.49000 -6.02000 H

O 0 37.52000 14.87000 -5.33000 H

N 0 40.91000 23.52000 4.05000 H

H 0 41.44000 23.17000 4.83000 H

C 0 39.52000 23.10000 3.90000 H

H 0 39.13000 23.73000 3.23000 H

C 0 39.35000 21.61000 3.39000 H

H 0 39.72000 20.94000 4.04000 H

C 0 37.80000 21.40000 3.08000 H

H 0 37.65000 20.47000 2.75000 H

H 0 37.27000 21.55000 3.91000 H

H 0 37.51000 22.06000 2.37000 H

C 0 40.15000 21.42000 2.13000 H

H 0 40.05000 20.48000 1.81000 H

H 0 39.82000 22.05000 1.43000 H

H 0 41.12000 21.60000 2.32000 H

C 0 38.92000 23.16000 5.32000 H

O 0 39.56000 22.67000 6.30000 H

N 0 35.20000 22.97000 7.78000 H

H 0 35.79000 22.65000 8.52000 H

C 0 33.78000 22.67000 7.78000 H

H 0 33.49000 22.75000 6.83000 H

C 0 33.58000 21.27000 8.34000 H

H 0 32.60000 21.10000 8.39000 H

H 0 33.97000 21.25000 9.26000 H

C 0 34.20000 20.11000 7.57000 H

H 0 35.19000 20.24000 7.49000 H

H 0 33.80000 20.05000 6.65000 H

C 0 33.82000 18.83000 8.47000 H

H 0 32.83000 18.69000 8.46000 H

H 0 34.12000 18.98000 9.41000 H

N 0 34.38000 17.55000 8.09000 H

H 0 33.95000 17.12000 7.31000 H

C 0 35.41000 16.85000 8.63000 H

N 0 35.66000 15.70000 8.05000 H

H 0 35.11000 15.41000 7.26000 H

H 0 36.40000 15.12000 8.38000 H

N 0 36.20000 17.23000 9.66000 H

H 0 36.06000 18.12000 10.10000 H

H 0 36.94000 16.63000 9.98000 H

C 0 33.02000 23.67000 8.71000 H

O 0 33.55000 24.07000 9.77000 H

N 0 30.89000 24.67000 3.76000 H

H 0 30.49000 25.08000 2.94000 H

C 0 32.32000 24.42000 3.79000 H

H 0 32.57000 24.28000 4.75000 H

C 0 32.71000 23.11000 2.94000 H

H 0 32.24000 23.11000 2.06000 H

C 0 34.26000 23.06000 2.57000 H

H 0 34.45000 22.23000 2.04000 H

H 0 34.50000 23.86000 2.02000 H

H 0 34.81000 23.05000 3.40000 H

C 0 32.23000 21.86000 3.71000 H

H 0 32.46000 21.04000 3.19000 H

H 0 32.67000 21.83000 4.60000 H

H 0 31.24000 21.90000 3.83000 H

C 0 33.00000 25.70000 3.30000 H

O 0 32.43000 26.42000 2.50000 H

N 0 41.78000 25.51000 -1.51000 H

H 0 40.81000 25.36000 -1.66000 H

C 0 42.80000 24.57000 -2.07000 H

H 0 43.29000 24.22000 -1.28000 H

C 0 42.07000 23.51000 -2.89000 H

H 0 42.65000 23.22000 -3.65000 H

H 0 41.22000 23.89000 -3.24000 H

C 0 41.73000 22.32000 -2.12000 H

C 0 40.44000 21.96000 -1.91000 H

H 0 39.70000 22.56000 -2.23000 H

C 0 40.12000 20.75000 -1.24000 H

H 0 39.16000 20.50000 -1.12000 H

C 0 41.11000 19.91000 -0.76000 H

O 0 40.73000 18.74000 -0.14000 H

H 0 41.55000 18.24000 0.15000 H

C 0 42.41000 20.20000 -0.93000 H

H 0 43.12000 19.59000 -0.58000 H

C 0 42.76000 21.44000 -1.65000 H

H 0 43.72000 21.67000 -1.81000 H

C 0 43.83000 25.22000 -2.96000 H

O 0 45.01000 24.89000 -2.89000 H

N 0 28.22000 19.15000 -4.44000 H

H 0 27.81000 19.98000 -4.06000 H

C 0 29.38000 18.59000 -3.80000 H

H 0 29.46000 17.63000 -4.08000 H

C 0 30.66000 19.31000 -4.29000 H

H 0 30.55000 20.29000 -4.10000 H

H 0 30.73000 19.18000 -5.28000 H

C 0 32.00000 18.85000 -3.66000 H

C 0 33.22000 19.53000 -3.99000 H

H 0 33.21000 20.28000 -4.65000 H

C 0 34.41000 19.16000 -3.40000 H

H 0 35.26000 19.63000 -3.63000 H

C 0 34.40000 18.13000 -2.47000 H

O 0 35.58000 17.80000 -1.93000 H

H 0 35.86000 17.65000 -2.98000 H

C 0 33.24000 17.42000 -2.14000 H

H 0 33.27000 16.65000 -1.51000 H

C 0 32.02000 17.83000 -2.73000 H

H 0 31.17000 17.37000 -2.48000 H

C 0 29.15000 18.66000 -2.29000 H

O 0 29.11000 17.61000 -1.61000 H

N 0 27.19000 16.20000 -0.13000 H

H 0 27.37000 16.74000 -0.95000 H

C 0 27.79000 14.81000 0.02000 H

H 0 27.06000 14.14000 0.15000 H

C 0 28.60000 14.50000 -1.26000 H

H 0 29.41000 15.09000 -1.27000 H

H 0 28.02000 14.72000 -2.05000 H

C 0 29.09000 13.09000 -1.47000 H

H 0 29.56000 12.68000 -0.70000 H

C 0 27.81000 12.28000 -1.79000 H

H 0 28.05000 11.32000 -1.94000 H

H 0 27.18000 12.34000 -1.01000 H

H 0 27.37000 12.65000 -2.61000 H

C 0 30.05000 13.22000 -2.73000 H

H 0 30.43000 12.32000 -2.95000 H

H 0 29.53000 13.56000 -3.51000 H

H 0 30.79000 13.86000 -2.52000 H

C 0 28.70000 14.77000 1.25000 H

O 0 28.80000 13.73000 1.91000 H

N 0 29.30000 15.90000 1.65000 H

H 0 29.11000 16.76000 1.17000 H

C 0 30.22000 15.89000 2.78000 H

H 0 30.72000 15.03000 2.66000 H

C 0 31.20000 17.09000 2.79000 H

H 0 31.96000 16.99000 3.43000 H

C 0 31.78000 17.26000 1.38000 H

H 0 32.41000 18.03000 1.37000 H

H 0 32.27000 16.42000 1.12000 H

H 0 31.04000 17.43000 0.73000 H

O 0 30.47000 18.26000 3.28000 H

H 0 31.08000 19.05000 3.29000 H

C 0 29.46000 15.88000 4.07000 H

O 0 30.01000 15.56000 5.12000 H

N 0 26.59000 12.19000 2.97000 H

H 0 27.00000 13.09000 2.83000 H

C 0 27.27000 11.01000 2.35000 H

H 0 27.30000 10.26000 3.01000 H

H 0 28.20000 11.26000 2.09000 H

C 0 26.49000 10.61000 1.14000 H

O 0 25.53000 11.30000 0.77000 H

N 0 26.91000 9.53000 0.48000 H

C 0 28.06000 8.72000 0.96000 H

H 0 27.79000 8.19000 1.77000 H

H 0 28.83000 9.31000 1.18000 H

C 0 28.36000 7.79000 -0.27000 H

H 0 28.75000 6.92000 0.03000 H

H 0 28.98000 8.23000 -0.91000 H

C 0 26.98000 7.57000 -0.92000 H

H 0 26.46000 6.87000 -0.42000 H

H 0 27.07000 7.29000 -1.87000 H

C 0 26.32000 9.00000 -0.77000 H

H 0 26.51000 9.68000 -1.48000 H

C 0 24.78000 8.90000 -0.83000 H

O 0 24.22000 9.08000 -1.91000 H

N 0 38.74300 26.11700 -7.20600 L

C 0 37.42200 26.52300 -7.53800 L

O 0 37.06600 26.75300 -8.72900 L

N 0 36.50000 26.75900 -6.62700 L

C 0 36.75200 26.57900 -5.30600 L

O 0 35.85200 26.73200 -4.43000 L

C 0 37.99700 26.16600 -4.92300 L

C 0 38.96900 25.91600 -5.85200 L

C 0 40.31900 25.56200 -5.30300 L

O 0 40.74000 26.22400 -4.31100 L

O 0 41.05100 24.78100 -5.91600 L

H 0 38.22500 26.03200 -3.86700 L

H 0 39.43100 25.99700 -7.93300 L

H 0 35.58600 27.08000 -6.90600 L

N 0 23.19000 2.27000 3.31000 L

H 0 24.04000 1.80000 3.09000 L

H 0 23.24000 2.65000 4.24000 L

H 0 22.43000 1.63000 3.25000 L

C 0 22.98000 3.38000 2.34000 L

H 0 22.93000 3.02000 1.41000 L

H 0 23.74000 4.03000 2.40000 L

C 0 21.70000 4.07000 2.70000 L

O 0 21.00000 4.60000 1.82000 L

N 0 21.39000 4.02000 3.99000 L

H 0 22.02000 3.52000 4.59000 L

C 0 20.21000 4.64000 4.61000 L

H 0 19.49000 4.68000 3.93000 L

C 0 19.75000 3.76000 5.75000 L

H 0 20.55000 3.42000 6.24000 L

H 0 19.23000 3.00000 5.38000 L

C 0 18.89000 4.47000 6.72000 L

O 0 19.41000 4.95000 7.76000 L

O 0 17.66000 4.49000 6.48000 L

C 0 20.55000 6.07000 5.09000 L

O 0 21.59000 6.33000 5.75000 L

N 0 19.70000 7.01000 4.65000 L

H 0 18.84000 6.74000 4.22000 L

C 0 20.06000 8.41000 4.79000 L

H 0 20.91000 8.52000 4.27000 L

C 0 19.00000 9.35000 4.21000 L

H 0 19.35000 10.29000 4.25000 L

H 0 18.17000 9.28000 4.78000 L

C 0 18.59000 9.09000 2.81000 L

H 0 18.56000 8.10000 2.67000 L

H 0 19.27000 9.49000 2.20000 L

C 0 17.22000 9.68000 2.50000 L

O 0 16.74000 10.59000 3.25000 L

O 0 16.63000 9.24000 1.49000 L

C 0 20.28000 8.71000 6.25000 L

O 0 21.24000 9.40000 6.60000 L

N 0 19.42000 8.15000 7.11000 L

H 0 18.74000 7.49000 6.79000 L

C 0 19.47000 8.50000 8.53000 L

H 0 19.47000 9.50000 8.52000 L

C 0 18.25000 7.96000 9.30000 L

H 0 18.51000 7.10000 9.74000 L

H 0 17.52000 7.80000 8.65000 L

C 0 17.73000 8.95000 10.41000 L

H 0 17.62000 9.86000 10.01000 L

H 0 18.40000 8.99000 11.15000 L

C 0 16.37000 8.50000 11.00000 L

H 0 16.04000 9.14000 11.69000 L

H 0 16.44000 7.58000 11.40000 L

N 0 15.42000 8.45000 9.92000 L

H 0 15.22000 9.32000 9.45000 L

C 0 14.78000 7.37000 9.48000 L

N 0 14.95000 6.19000 10.07000 L

H 0 15.56000 6.11000 10.86000 L

H 0 14.46000 5.39000 9.73000 L

N 0 13.95000 7.48000 8.45000 L

H 0 13.81000 8.37000 8.01000 L

H 0 13.47000 6.67000 8.11000 L

C 0 20.76000 8.08000 9.20000 L

O 0 21.41000 8.89000 9.92000 L

N 0 21.16000 6.84000 8.94000 L

H 0 20.61000 6.26000 8.33000 L

C 0 22.39000 6.30000 9.52000 L

H 0 22.24000 6.40000 10.50000 L

C 0 22.56000 4.84000 9.11000 L

H 0 22.54000 4.78000 8.11000 L

H 0 21.81000 4.31000 9.49000 L

C 0 23.86000 4.25000 9.59000 L

C 0 24.05000 3.98000 10.93000 L

H 0 23.31000 4.17000 11.58000 L

C 0 25.23000 3.45000 11.38000 L

H 0 25.36000 3.27000 12.35000 L

C 0 26.26000 3.18000 10.50000 L

H 0 27.12000 2.78000 10.83000 L

C 0 26.10000 3.44000 9.16000 L

H 0 26.84000 3.25000 8.51000 L

C 0 24.88000 3.98000 8.71000 L

H 0 24.76000 4.17000 7.73000 L

C 0 23.65000 7.08000 9.08000 L H 79

O 0 24.59000 7.27000 9.89000 L

N 0 23.58000 10.24000 7.88000 L H 98

H 0 22.80000 9.74000 7.51000 L

C 0 23.38000 11.61000 8.37000 L

H 0 24.05000 12.17000 7.90000 L

C 0 21.96000 12.06000 8.04000 L

H 0 21.81000 12.99000 8.37000 L

H 0 21.83000 12.03000 7.05000 L

H 0 21.30000 11.44000 8.48000 L

C 0 23.62000 11.74000 9.88000 L

O 0 24.39000 12.60000 10.34000 L

N 0 23.00000 10.86000 10.66000 L

H 0 22.47000 10.13000 10.24000 L

C 0 23.06000 10.94000 12.13000 L

H 0 23.14000 11.92000 12.33000 L

C 0 21.78000 10.33000 12.76000 L

H 0 21.83000 10.42000 13.75000 L

H 0 21.74000 9.36000 12.52000 L

C 0 20.51000 11.02000 12.25000 L

H 0 20.56000 11.09000 11.26000 L

H 0 20.47000 11.94000 12.65000 L

C 0 19.22000 10.28000 12.61000 L

O 0 19.12000 9.78000 13.76000 L

O 0 18.32000 10.22000 11.74000 L

C 0 24.30000 10.25000 12.71000 L

O 0 24.80000 10.66000 13.74000 L

N 0 24.81000 9.20000 12.07000 L

H 0 24.47000 8.93000 11.17000 L

C 0 25.88000 8.47000 12.74000 L

H 0 26.04000 9.02000 13.55000 L

C 0 25.38000 7.05000 13.11000 L

H 0 26.11000 6.55000 13.59000 L

H 0 25.13000 6.55000 12.28000 L

C 0 24.19000 7.08000 14.01000 L

N 0 22.91000 6.79000 13.57000 L

C 0 22.05000 6.99000 14.55000 L

H 0 21.06000 6.82000 14.50000 L

N 0 22.71000 7.44000 15.60000 L

H 0 22.31000 7.68000 16.48000 L

C 0 24.05000 7.51000 15.29000 L

H 0 24.79000 7.82000 15.89000 L

C 0 27.22000 8.45000 12.01000 L

O 0 28.28000 8.87000 12.56000 L

N 0 27.18000 7.94000 10.79000 L

H 0 26.31000 7.72000 10.37000 L

C 0 28.42000 7.69000 10.09000 L

H 0 28.95000 7.13000 10.72000 L

C 0 28.11000 6.94000 8.80000 L

H 0 27.73000 7.60000 8.15000 L

H 0 27.43000 6.24000 9.01000 L

C 0 29.26000 6.26000 8.11000 L

H 0 29.83000 5.73000 8.74000 L

C 0 28.65000 5.35000 7.05000 L

H 0 29.38000 4.87000 6.56000 L

H 0 28.05000 4.68000 7.49000 L

H 0 28.13000 5.90000 6.40000 L

C 0 30.09000 7.31000 7.48000 L

H 0 30.87000 6.89000 7.01000 L

H 0 29.54000 7.82000 6.82000 L

H 0 30.43000 7.94000 8.18000 L

C 0 29.22000 8.99000 9.80000 L H 100

O 0 30.43000 9.04000 10.07000 L

N 0 28.86000 12.23000 11.02000 L H 115

C 0 27.46000 11.78000 11.08000 L

H 0 27.41000 10.80000 11.26000 L

H 0 26.98000 11.99000 10.23000 L

C 0 26.90000 12.58000 12.25000 L

H 0 26.20000 12.05000 12.74000 L

H 0 26.49000 13.44000 11.93000 L

C 0 28.10000 12.87000 13.17000 L

H 0 28.20000 12.15000 13.85000 L

H 0 27.99000 13.75000 13.63000 L

C 0 29.33000 12.91000 12.24000 L

H 0 29.64000 13.83000 12.02000 L

C 0 30.53000 12.20000 12.87000 L

O 0 31.45000 12.85000 13.38000 L

N 0 30.53000 10.87000 12.85000 L

H 0 29.78000 10.37000 12.43000 L

C 0 31.66000 10.15000 13.45000 L

H 0 31.84000 10.59000 14.33000 L

C 0 31.25000 8.67000 13.62000 L

H 0 30.91000 8.26000 12.78000 L

C 0 32.43000 7.82000 14.11000 L

H 0 32.14000 6.87000 14.22000 L

H 0 33.18000 7.87000 13.45000 L

H 0 32.75000 8.17000 14.99000 L

O 0 30.18000 8.65000 14.58000 L

H 0 29.88000 7.71000 14.73000 L

C 0 32.93000 10.30000 12.63000 L H 117

O 0 34.05000 10.56000 13.13000 L

N 0 34.41000 13.57000 13.19000 L H 151

H 0 33.79000 12.80000 13.03000 L

C 0 35.07000 13.70000 14.49000 L

H 0 34.48000 13.30000 15.19000 L

H 0 35.21000 14.67000 14.69000 L

C 0 36.40000 12.99000 14.48000 L

O 0 37.23000 13.22000 15.33000 L

N 0 36.64000 12.12000 13.49000 L

H 0 35.97000 11.98000 12.77000 L

C 0 37.87000 11.40000 13.50000 L

H 0 38.13000 11.47000 14.46000 L

C 0 37.69000 9.96000 13.02000 L

H 0 38.60000 9.62000 12.82000 L

H 0 37.16000 10.03000 12.17000 L

C 0 37.00000 8.87000 13.84000 L

H 0 36.03000 9.06000 13.99000 L

C 0 37.16000 7.52000 13.06000 L

H 0 36.72000 6.79000 13.57000 L

H 0 36.73000 7.60000 12.16000 L

H 0 38.13000 7.32000 12.94000 L

C 0 37.67000 8.82000 15.20000 L

H 0 37.25000 8.12000 15.76000 L

H 0 38.65000 8.62000 15.08000 L

H 0 37.57000 9.71000 15.65000 L

C 0 38.93000 12.02000 12.60000 L

O 0 40.10000 11.82000 12.88000 L

N 0 38.55000 12.67000 11.50000 L

H 0 37.57000 12.85000 11.35000 L

C 0 39.52000 13.14000 10.51000 L

H 0 40.41000 12.92000 10.90000 L

C 0 39.22000 12.46000 9.18000 L

H 0 39.82000 12.85000 8.48000 L

H 0 38.26000 12.64000 8.94000 L

C 0 39.43000 10.94000 9.20000 L

H 0 39.11000 10.50000 10.04000 L

C 0 38.63000 10.38000 8.07000 L

H 0 38.74000 9.38000 8.05000 L

H 0 37.67000 10.61000 8.20000 L

H 0 38.96000 10.77000 7.21000 L

C 0 40.91000 10.64000 9.09000 L

H 0 41.06000 9.65000 9.10000 L

H 0 41.26000 11.02000 8.23000 L

H 0 41.40000 11.07000 9.85000 L

C 0 39.43000 14.64000 10.26000 L

O 0 38.35000 15.22000 10.34000 L

N 0 40.50000 15.28000 9.86000 L

H 0 41.41000 14.86000 9.87000 L

C 0 40.30000 16.66000 9.42000 L

H 0 39.75000 17.14000 10.10000 L

C 0 41.61000 17.38000 9.33000 L

H 0 42.07000 17.35000 10.22000 L

H 0 41.46000 18.33000 9.06000 L

C 0 42.51000 16.74000 8.31000 L

O 0 42.84000 15.55000 8.57000 L

O 0 42.80000 17.36000 7.24000 L

C 0 39.61000 16.59000 8.00000 L H 153

O 0 39.58000 15.50000 7.34000 L

N 0 40.29000 17.79000 4.94000 L H 165

H 0 40.71000 18.41000 5.59000 L

C 0 41.02000 17.31000 3.74000 L

H 0 40.41000 17.51000 2.96000 L

C 0 42.32000 18.08000 3.59000 L

H 0 42.93000 17.86000 4.35000 L

H 0 42.13000 19.06000 3.57000 L

C 0 42.96000 17.66000 2.28000 L

H 0 42.26000 17.67000 1.57000 L

H 0 43.31000 16.73000 2.39000 L

C 0 44.12000 18.57000 1.84000 L

O 0 44.93000 18.13000 1.00000 L

O 0 44.24000 19.72000 2.29000 L

C 0 41.27000 15.80000 3.74000 L

O 0 41.16000 15.09000 2.70000 L

N 0 41.67000 15.26000 4.92000 L

H 0 41.84000 15.84000 5.72000 L

C 0 41.83000 13.83000 5.02000 L

H 0 42.51000 13.64000 4.32000 L

C 0 42.27000 13.41000 6.45000 L

H 0 42.40000 12.42000 6.52000 L

H 0 41.61000 13.70000 7.14000 L

O 0 43.52000 14.03000 6.73000 L

H 0 43.83000 13.78000 7.65000 L

C 0 40.54000 13.11000 4.71000 L H 167

O 0 40.57000 12.01000 4.11000 L

N 0 40.20000 13.11000 0.93000 L H 192

H 0 40.53000 13.74000 1.63000 L

C 0 41.10000 12.16000 0.31000 L

H 0 41.05000 12.38000 -0.66000 L

C 0 42.53000 12.33000 0.87000 L

H 0 43.12000 11.61000 0.50000 L

H 0 42.51000 12.26000 1.87000 L

C 0 43.13000 13.68000 0.50000 L

H 0 42.50000 14.39000 0.79000 L

H 0 43.24000 13.71000 -0.49000 L

C 0 44.51000 13.90000 1.18000 L

H 0 44.42000 13.89000 2.17000 L

H 0 44.91000 14.77000 0.89000 L

N 0 45.45000 12.83000 0.80000 L

H 0 45.17000 12.21000 0.06000 L

C 0 46.65000 12.62000 1.37000 L

N 0 47.10000 13.42000 2.34000 L

H 0 46.55000 14.20000 2.65000 L

H 0 47.99000 13.25000 2.76000 L

N 0 47.41000 11.59000 0.96000 L

H 0 47.08000 10.99000 0.23000 L

H 0 48.30000 11.43000 1.38000 L

C 0 40.64000 10.73000 0.48000 L

O 0 40.77000 9.94000 -0.45000 L

N 0 40.20000 10.35000 1.69000 L

H 0 40.19000 11.01000 2.44000 L

C 0 39.71000 8.96000 1.92000 L

H 0 40.47000 8.37000 1.64000 L

C 0 39.36000 8.75000 3.38000 L

H 0 38.62000 9.39000 3.59000 L

H 0 40.17000 8.99000 3.91000 L

C 0 38.91000 7.37000 3.86000 L

H 0 38.10000 7.04000 3.35000 L

C 0 40.16000 6.41000 3.64000 L

H 0 39.92000 5.49000 3.93000 L

H 0 40.40000 6.40000 2.67000 L

H 0 40.93000 6.75000 4.18000 L

C 0 38.47000 7.45000 5.34000 L

H 0 38.17000 6.54000 5.65000 L

H 0 39.24000 7.76000 5.90000 L

H 0 37.71000 8.10000 5.43000 L

C 0 38.44000 8.68000 1.12000 L H 194

O 0 38.27000 7.57000 0.60000 L

N 0 37.73000 9.99000 -1.77000 L H 202

H 0 38.21000 10.66000 -1.22000 L

C 0 38.12000 9.74000 -3.20000 L

H 0 37.29000 9.82000 -3.76000 L

C 0 39.14000 10.82000 -3.67000 L

H 0 39.90000 10.82000 -3.02000 L

C 0 39.81000 10.49000 -5.02000 L

H 0 40.45000 11.21000 -5.27000 L

H 0 40.30000 9.62000 -4.94000 L

H 0 39.11000 10.40000 -5.73000 L

C 0 38.47000 12.26000 -3.66000 L

H 0 39.14000 12.94000 -3.96000 L

H 0 37.69000 12.26000 -4.28000 L

H 0 38.17000 12.48000 -2.73000 L

C 0 38.65000 8.30000 -3.35000 L

O 0 38.27000 7.54000 -4.27000 L

N 0 39.49000 7.90000 -2.40000 L

H 0 39.71000 8.53000 -1.65000 L

C 0 40.08000 6.57000 -2.43000 L

H 0 40.59000 6.53000 -3.29000 L

C 0 40.97000 6.34000 -1.20000 L

H 0 41.21000 5.37000 -1.14000 L

H 0 40.47000 6.61000 -0.38000 L

C 0 42.23000 7.10000 -1.22000 L

H 0 42.01000 8.06000 -1.04000 L

H 0 42.62000 7.02000 -2.14000 L

C 0 43.22000 6.50000 -0.11000 L

H 0 43.84000 5.83000 -0.53000 L

H 0 42.70000 6.05000 0.61000 L

N 0 44.08000 7.52000 0.55000 L

H 0 44.95000 7.74000 0.10000 L

C 0 43.82000 8.17000 1.70000 L

N 0 42.71000 7.94000 2.37000 L

H 0 42.05000 7.27000 2.03000 L

H 0 42.53000 8.44000 3.22000 L

N 0 44.69000 9.05000 2.20000 L

H 0 45.55000 9.23000 1.73000 L

H 0 44.48000 9.53000 3.06000 L

C 0 39.03000 5.52000 -2.38000 L

O 0 39.01000 4.55000 -3.18000 L

N 0 38.14000 5.65000 -1.42000 L

H 0 38.18000 6.44000 -0.80000 L

C 0 37.13000 4.68000 -1.27000 L

H 0 37.59000 3.82000 -1.06000 L

C 0 36.22000 5.16000 -0.17000 L

H 0 35.74000 5.98000 -0.48000 L

H 0 36.76000 5.38000 0.64000 L

C 0 35.22000 4.21000 0.23000 L

C 0 35.51000 3.29000 1.22000 L

H 0 36.42000 3.27000 1.63000 L

C 0 34.53000 2.38000 1.67000 L

H 0 34.76000 1.71000 2.37000 L

C 0 33.22000 2.42000 1.13000 L

H 0 32.53000 1.78000 1.45000 L

C 0 32.91000 3.36000 0.14000 L

H 0 31.99000 3.38000 -0.26000 L

C 0 33.91000 4.28000 -0.29000 L

H 0 33.69000 4.98000 -0.96000 L

C 0 36.28000 4.58000 -2.52000 L

O 0 35.86000 3.46000 -2.93000 L

N 0 35.92000 5.74000 -3.06000 L

H 0 36.27000 6.60000 -2.69000 L

C 0 35.02000 5.75000 -4.21000 L

H 0 34.20000 5.24000 -3.96000 L

C 0 34.69000 7.21000 -4.57000 L

H 0 35.52000 7.75000 -4.72000 L

C 0 33.84000 7.31000 -5.85000 L

H 0 33.65000 8.27000 -6.05000 L

H 0 34.34000 6.90000 -6.61000 L

H 0 32.98000 6.82000 -5.71000 L

O 0 34.02000 7.81000 -3.46000 L

H 0 33.79000 8.76000 -3.68000 L

C 0 35.74000 5.11000 -5.38000 L

O 0 35.13000 4.39000 -6.18000 L

N 0 37.02000 5.41000 -5.54000 L

H 0 37.50000 5.97000 -4.86000 L

C 0 37.71000 4.92000 -6.71000 L

H 0 37.11000 5.16000 -7.46000 L

C 0 39.03000 5.57000 -6.86000 L

H 0 38.97000 6.56000 -6.74000 L

H 0 39.45000 5.36000 -7.75000 L

O 0 39.81000 5.03000 -5.85000 L

H 0 40.73000 5.43000 -5.89000 L

C 0 37.97000 3.42000 -6.61000 L

O 0 38.14000 2.74000 -7.63000 L

N 0 38.05000 2.90000 -5.40000 L

H 0 37.98000 3.49000 -4.60000 L

C 0 38.23000 1.46000 -5.24000 L

H 0 38.90000 1.22000 -5.95000 L

C 0 38.78000 1.08000 -3.87000 L

H 0 38.78000 0.09000 -3.77000 L

H 0 38.20000 1.49000 -3.16000 L

C 0 40.19000 1.60000 -3.69000 L

H 0 40.14000 2.58000 -3.82000 L

C 0 40.68000 1.41000 -2.29000 L

H 0 41.61000 1.76000 -2.21000 L

H 0 40.08000 1.91000 -1.66000 L

H 0 40.66000 0.44000 -2.05000 L

C 0 41.20000 0.98000 -4.72000 L

H 0 42.11000 1.35000 -4.56000 L

H 0 41.21000 -0.01000 -4.62000 L

H 0 40.90000 1.22000 -5.65000 L

C 0 36.94000 0.73000 -5.48000 L

O 0 36.95000 -0.49000 -5.44000 L

N 0 35.83000 1.43000 -5.68000 L

H 0 35.85000 2.43000 -5.65000 L

C 0 34.61000 0.75000 -5.94000 L

H 0 34.79000 0.07000 -6.65000 L

H 0 33.96000 1.42000 -6.29000 L

C 0 34.02000 0.06000 -4.73000 L

O 0 33.13000 -0.80000 -4.86000 L

N 0 34.44000 0.49000 -3.54000 L

H 0 35.18000 1.16000 -3.50000 L

C 0 33.85000 -0.01000 -2.29000 L

H 0 33.69000 -0.99000 -2.42000 L

C 0 34.83000 0.21000 -1.13000 L

H 0 34.46000 -0.21000 -0.30000 L

H 0 34.94000 1.20000 -0.98000 L

C 0 36.19000 -0.39000 -1.42000 L

H 0 36.46000 -0.19000 -2.36000 L

C 0 37.27000 0.18000 -0.52000 L

H 0 38.15000 -0.24000 -0.74000 L

H 0 37.33000 1.17000 -0.66000 L

H 0 37.04000 -0.01000 0.44000 L

C 0 36.08000 -1.88000 -1.23000 L

H 0 36.97000 -2.31000 -1.42000 L

H 0 35.81000 -2.08000 -0.29000 L

H 0 35.40000 -2.25000 -1.86000 L

C 0 32.51000 0.66000 -2.00000 L

O 0 32.24000 1.10000 -0.90000 L

N 0 31.67000 0.78000 -2.99000 L

H 0 31.86000 0.37000 -3.88000 L

C 0 30.45000 1.53000 -2.77000 L

H 0 30.51000 1.91000 -1.84000 L

C 0 30.29000 2.61000 -3.85000 L

H 0 29.33000 2.90000 -3.79000 L

H 0 30.44000 2.15000 -4.72000 L

C 0 31.07000 3.91000 -3.97000 L

H 0 32.05000 3.75000 -3.81000 L

C 0 30.89000 4.44000 -5.41000 L

H 0 31.40000 5.29000 -5.51000 L

H 0 31.24000 3.76000 -6.06000 L

H 0 29.92000 4.61000 -5.58000 L

C 0 30.60000 4.91000 -2.89000 L

H 0 31.12000 5.76000 -2.97000 L

H 0 29.63000 5.11000 -3.02000 L

H 0 30.74000 4.52000 -1.98000 L

C 0 29.30000 0.59000 -2.96000 L

O 0 29.25000 -0.08000 -3.99000 L

N 0 28.34000 0.58000 -2.02000 L

C 0 28.07000 1.51000 -0.91000 L

H 0 28.53000 2.38000 -1.07000 L

H 0 28.39000 1.12000 -0.04000 L

C 0 26.55000 1.66000 -0.93000 L

H 0 26.27000 2.39000 -1.55000 L

H 0 26.20000 1.86000 -0.01000 L

C 0 26.07000 0.28000 -1.43000 L

H 0 25.17000 0.35000 -1.86000 L

H 0 26.03000 -0.38000 -0.68000 L

C 0 27.14000 -0.13000 -2.47000 L

H 0 27.28000 -1.12000 -2.53000 L

C 0 26.77000 0.43000 -3.84000 L

O 0 26.37000 1.34000 -3.91000 L

O 0 26.97000 -0.23000 -4.86000 L

N 0 18.49000 3.33000 -6.29000 L

H 0 18.32000 2.37000 -6.51000 L

H 0 19.45000 3.54000 -6.47000 L

H 0 18.30000 3.50000 -5.32000 L

C 0 17.63000 4.19000 -7.12000 L

H 0 16.71000 3.93000 -6.84000 L

C 0 17.87000 5.70000 -6.83000 L

H 0 18.77000 5.97000 -7.16000 L

H 0 17.82000 5.86000 -5.84000 L

C 0 16.85000 6.59000 -7.49000 L

C 0 15.64000 6.87000 -6.87000 L

H 0 15.46000 6.52000 -5.95000 L

C 0 14.67000 7.63000 -7.52000 L

H 0 13.80000 7.81000 -7.07000 L

C 0 14.92000 8.16000 -8.77000 L

H 0 14.23000 8.72000 -9.23000 L

C 0 16.14000 7.89000 -9.40000 L

H 0 16.33000 8.28000 -10.30000 L

C 0 17.07000 7.08000 -8.77000 L

H 0 17.91000 6.85000 -9.26000 L

C 0 17.78000 3.89000 -8.62000 L

O 0 18.83000 4.10000 -9.20000 L

N 0 16.73000 3.37000 -9.25000 L

H 0 15.94000 3.08000 -8.71000 L

C 0 16.71000 3.19000 -10.71000 L

H 0 17.64000 3.03000 -11.03000 L

C 0 15.84000 2.01000 -11.07000 L

H 0 15.72000 2.00000 -12.06000 L

H 0 14.95000 2.13000 -10.63000 L

C 0 16.41000 0.66000 -10.64000 L

H 0 15.70000 0.13000 -10.18000 L

H 0 17.18000 0.81000 -10.02000 L

C 0 16.91000 -0.10000 -11.87000 L

O 0 16.68000 0.35000 -13.01000 L

N 0 17.60000 -1.25000 -11.66000 L

H 0 17.77000 -1.56000 -10.73000 L

H 0 17.94000 -1.78000 -12.44000 L

C 0 16.08000 4.45000 -11.28000 L

O 0 14.98000 4.86000 -10.85000 L

N 0 16.79000 5.10000 -12.19000 L

H 0 17.64000 4.69000 -12.53000 L

C 0 16.36000 6.40000 -12.71000 L

H 0 16.15000 6.94000 -11.89000 L

C 0 17.47000 7.09000 -13.54000 L

H 0 17.12000 7.94000 -13.91000 L

H 0 17.73000 6.47000 -14.28000 L

C 0 18.74000 7.41000 -12.71000 L

O 0 19.82000 7.47000 -13.30000 L

O 0 18.63000 7.58000 -11.48000 L

C 0 15.12000 6.22000 -13.60000 L

O 0 15.14000 5.49000 -14.58000 L

N 0 14.05000 6.93000 -13.31000 L

H 0 14.00000 7.47000 -12.47000 L

C 0 12.94000 6.91000 -14.26000 L

H 0 12.87000 5.97000 -14.57000 L

C 0 11.67000 7.36000 -13.54000 L

H 0 11.69000 7.10000 -12.58000 L

H 0 10.86000 6.96000 -13.97000 L

O 0 11.56000 8.75000 -13.61000 L

H 0 10.72000 9.04000 -13.14000 L

C 0 13.21000 7.79000 -15.48000 L

O 0 14.00000 8.74000 -15.43000 L

N 0 12.55000 7.46000 -16.58000 L

H 0 12.01000 6.62000 -16.60000 L

C 0 12.62000 8.28000 -17.74000 L

H 0 13.57000 8.15000 -18.00000 L

C 0 11.56000 7.80000 -18.75000 L

H 0 11.47000 8.47000 -19.49000 L

H 0 10.68000 7.70000 -18.29000 L

C 0 11.92000 6.45000 -19.37000 L

O 0 11.14000 5.96000 -20.21000 L

O 0 12.98000 5.88000 -19.00000 L

C 0 12.34000 9.79000 -17.52000 L

O 0 12.81000 10.61000 -18.32000 L

N 0 11.51000 10.15000 -16.53000 L

H 0 11.13000 9.45000 -15.92000 L

C 0 11.16000 11.59000 -16.33000 L

H 0 10.72000 11.87000 -17.18000 L

C 0 10.23000 11.79000 -15.10000 L

H 0 10.12000 12.77000 -14.95000 L

H 0 10.67000 11.38000 -14.30000 L

C 0 8.82000 11.18000 -15.21000 L

H 0 8.97000 10.20000 -15.30000 L

H 0 8.45000 11.55000 -16.06000 L

S 0 7.81000 11.63000 -13.71000 L

C 0 7.84000 13.43000 -13.89000 L

H 0 7.33000 13.85000 -13.14000 L

H 0 8.79000 13.75000 -13.86000 L

H 0 7.43000 13.69000 -14.76000 L

C 0 12.43000 12.37000 -16.01000 L

O 0 12.41000 13.59000 -16.01000 L

N 0 13.50000 11.66000 -15.62000 L

H 0 13.48000 10.67000 -15.63000 L

C 0 14.72000 12.38000 -15.16000 L

H 0 14.40000 13.27000 -14.83000 L

C 0 15.39000 11.65000 -14.01000 L

H 0 16.29000 12.07000 -13.89000 L

H 0 15.50000 10.70000 -14.30000 L

C 0 14.69000 11.65000 -12.63000 L

H 0 13.91000 11.03000 -12.67000 L

C 0 15.64000 11.08000 -11.57000 L

H 0 15.19000 11.08000 -10.68000 L

H 0 15.90000 10.15000 -11.82000 L

H 0 16.47000 11.65000 -11.53000 L

C 0 14.17000 13.09000 -12.25000 L

H 0 13.72000 13.06000 -11.36000 L

H 0 14.94000 13.73000 -12.22000 L

H 0 13.51000 13.39000 -12.94000 L

C 0 15.69000 12.64000 -16.32000 L

O 0 16.67000 13.42000 -16.19000 L

N 0 15.39000 12.07000 -17.48000 L

H 0 14.52000 11.59000 -17.60000 L

C 0 16.35000 12.14000 -18.57000 L

H 0 17.25000 12.03000 -18.16000 L

C 0 16.06000 11.02000 -19.59000 L

H 0 15.10000 11.04000 -19.85000 L

H 0 16.28000 10.13000 -19.18000 L

C 0 16.92000 11.21000 -20.85000 L

H 0 17.88000 11.09000 -20.61000 L

H 0 16.78000 12.14000 -21.20000 L

C 0 16.51000 10.19000 -21.93000 L

O 0 16.16000 10.62000 -23.06000 L

O 0 16.48000 8.97000 -21.61000 L

C 0 16.31000 13.49000 -19.23000 L

O 0 15.25000 14.08000 -19.40000 L

N 0 17.44000 14.01000 -19.59000 L

H 0 18.29000 13.50000 -19.44000 L

C 0 17.48000 15.29000 -20.18000 L

H 0 16.54000 15.48000 -20.43000 L

C 0 18.17000 16.32000 -19.19000 L

H 0 19.05000 15.99000 -18.84000 L

C 0 18.44000 17.69000 -19.91000 L

H 0 18.87000 18.32000 -19.27000 L

H 0 19.04000 17.54000 -20.69000 L

H 0 17.57000 18.07000 -20.22000 L

C 0 17.37000 16.43000 -17.97000 L

H 0 17.80000 17.08000 -17.34000 L

H 0 16.45000 16.76000 -18.21000 L

H 0 17.30000 15.54000 -17.53000 L

C 0 18.47000 15.21000 -21.36000 L

O 0 19.52000 14.55000 -21.26000 L

N 0 18.13000 15.86000 -22.45000 L

H 0 17.25000 16.32000 -22.49000 L

C 0 19.01000 15.92000 -23.62000 L

H 0 19.76000 15.29000 -23.43000 L

C 0 18.19000 15.54000 -24.89000 L

H 0 17.29000 15.98000 -24.83000 L

H 0 18.07000 14.55000 -24.92000 L

C 0 18.87000 15.99000 -26.18000 L

H 0 19.84000 16.06000 -25.98000 L

H 0 18.50000 16.90000 -26.39000 L

C 0 18.68000 15.06000 -27.48000 L

H 0 18.76000 15.61000 -28.31000 L

H 0 17.79000 14.61000 -27.45000 L

N 0 19.72000 13.99000 -27.57000 L

H 0 19.43000 13.05000 -27.41000 L

C 0 21.01000 14.19000 -27.86000 L

N 0 21.47000 15.42000 -28.10000 L

H 0 20.84000 16.20000 -28.06000 L

H 0 22.43000 15.57000 -28.31000 L

N 0 21.86000 13.16000 -27.89000 L

H 0 21.52000 12.24000 -27.70000 L

H 0 22.82000 13.31000 -28.10000 L

C 0 19.56000 17.32000 -23.81000 L

O 0 18.75000 18.23000 -24.08000 L

N 0 20.87000 17.56000 -23.63000 L

H 0 21.47000 16.81000 -23.35000 L

C 0 21.44000 18.92000 -23.84000 L

H 0 20.78000 19.30000 -24.49000 L

C 0 21.72000 19.76000 -22.53000 L

H 0 22.71000 19.92000 -22.46000 L

C 0 21.09000 21.17000 -22.58000 L

H 0 21.29000 21.66000 -21.73000 L

H 0 21.46000 21.68000 -23.36000 L

H 0 20.09000 21.09000 -22.69000 L

C 0 21.61000 19.02000 -21.23000 L

H 0 21.81000 19.65000 -20.48000 L

H 0 20.69000 18.66000 -21.13000 L

H 0 22.27000 18.27000 -21.22000 L

C 0 22.88000 18.79000 -24.23000 L

O 0 23.42000 17.74000 -24.05000 L

N 0 23.54000 19.87000 -24.65000 L

H 0 23.03000 20.71000 -24.84000 L

C 0 25.00000 19.85000 -24.85000 L

H 0 25.18000 20.73000 -25.29000 L

C 0 25.80000 19.71000 -23.47000 L

H 0 26.78000 19.67000 -23.68000 L

H 0 25.52000 18.86000 -23.02000 L

C 0 25.56000 20.91000 -22.47000 L

H 0 24.57000 20.99000 -22.36000 L

C 0 26.15000 20.68000 -21.06000 L

H 0 25.95000 21.48000 -20.48000 L

H 0 25.73000 19.87000 -20.65000 L

H 0 27.14000 20.55000 -21.12000 L

C 0 26.01000 22.23000 -23.02000 L

H 0 25.83000 22.94000 -22.35000 L

H 0 26.99000 22.19000 -23.22000 L

H 0 25.51000 22.42000 -23.86000 L

C 0 25.41000 18.73000 -25.80000 L

O 0 26.59000 18.38000 -25.85000 L

N 0 24.45000 18.21000 -26.58000 L

H 0 23.51000 18.50000 -26.45000 L

C 0 24.78000 17.21000 -27.63000 L

H 0 25.66000 17.45000 -28.04000 L

H 0 24.07000 17.23000 -28.33000 L

C 0 24.86000 15.84000 -27.04000 L

O 0 25.45000 14.89000 -27.64000 L

N 0 24.31000 15.72000 -25.83000 L

H 0 23.96000 16.55000 -25.39000 L

C 0 24.19000 14.46000 -25.12000 L

H 0 24.21000 13.82000 -25.89000 L

C 0 25.42000 14.33000 -24.14000 L

H 0 25.35000 13.47000 -23.63000 L

H 0 25.42000 15.09000 -23.50000 L

C 0 26.73000 14.33000 -24.87000 L

N 0 27.28000 13.17000 -25.40000 L

C 0 28.41000 13.47000 -26.03000 L

H 0 29.02000 12.82000 -26.49000 L

N 0 28.59000 14.79000 -25.96000 L

H 0 29.35000 15.29000 -26.37000 L

C 0 27.57000 15.34000 -25.23000 L

H 0 27.46000 16.31000 -25.00000 L

C 0 22.81000 14.15000 -24.39000 L

O 0 22.00000 15.01000 -23.99000 L

N 0 22.61000 12.88000 -24.13000 L

H 0 23.23000 12.21000 -24.54000 L

C 0 21.55000 12.41000 -23.28000 L

H 0 20.77000 13.03000 -23.36000 L

C 0 21.18000 11.00000 -23.69000 L

H 0 22.02000 10.46000 -23.77000 L

H 0 20.72000 11.03000 -24.57000 L

C 0 20.27000 10.33000 -22.70000 L

H 0 19.56000 10.97000 -22.42000 L

H 0 20.80000 10.05000 -21.90000 L

C 0 19.63000 9.09000 -23.35000 L

H 0 19.31000 9.33000 -24.26000 L

H 0 18.85000 8.80000 -22.79000 L

C 0 20.59000 7.91000 -23.48000 L

H 0 20.96000 7.68000 -22.58000 L

H 0 21.34000 8.15000 -24.10000 L

N 0 19.79000 6.72000 -24.04000 L

H 0 20.39000 5.93000 -24.14000 L

H 0 19.04000 6.50000 -23.42000 L

H 0 19.42000 6.97000 -24.94000 L

C 0 22.15000 12.31000 -21.88000 L

O 0 23.28000 11.84000 -21.75000 L

N 0 21.38000 12.66000 -20.83000 L

H 0 20.45000 12.97000 -20.98000 L

C 0 21.92000 12.57000 -19.44000 L

H 0 22.85000 12.19000 -19.40000 L

C 0 21.97000 13.96000 -18.76000 L

H 0 22.08000 13.85000 -17.78000 L

H 0 21.11000 14.44000 -18.95000 L

C 0 23.10000 14.82000 -19.27000 L

C 0 23.05000 15.39000 -20.52000 L

H 0 22.25000 15.21000 -21.10000 L

C 0 24.09000 16.22000 -21.01000 L

H 0 24.01000 16.65000 -21.90000 L

C 0 25.26000 16.42000 -20.21000 L

H 0 26.03000 16.95000 -20.56000 L

C 0 25.30000 15.85000 -18.94000 L

H 0 26.07000 16.04000 -18.33000 L

C 0 24.25000 15.01000 -18.50000 L

H 0 24.34000 14.54000 -17.62000 L

C 0 20.88000 11.71000 -18.77000 L

O 0 19.71000 12.11000 -18.75000 L

N 0 21.22000 10.51000 -18.32000 L

H 0 22.15000 10.17000 -18.37000 L

C 0 20.16000 9.72000 -17.73000 L

H 0 19.53000 9.71000 -18.51000 L

C 0 20.64000 8.30000 -17.39000 L

H 0 21.35000 8.02000 -18.04000 L

H 0 19.87000 7.67000 -17.45000 L

C 0 21.22000 8.05000 -16.11000 L

H 0 20.69000 8.57000 -15.44000 L

H 0 22.16000 8.39000 -16.13000 L

C 0 21.15000 6.48000 -15.84000 L

H 0 20.20000 6.19000 -15.70000 L

H 0 21.70000 6.24000 -15.04000 L

N 0 21.68000 5.71000 -16.99000 L

H 0 21.52000 6.08000 -17.91000 L

C 0 22.34000 4.55000 -16.90000 L

N 0 22.56000 4.00000 -15.69000 L

H 0 22.23000 4.46000 -14.87000 L

H 0 23.06000 3.14000 -15.62000 L

N 0 22.77000 3.92000 -18.00000 L

H 0 22.60000 4.31000 -18.90000 L

H 0 23.27000 3.06000 -17.92000 L

C 0 19.43000 10.36000 -16.52000 L

O 0 18.21000 10.08000 -16.29000 L

N 0 20.13000 11.21000 -15.76000 L

H 0 21.12000 11.33000 -15.89000 L

C 0 19.40000 11.98000 -14.72000 L

H 0 18.45000 12.11000 -14.99000 L

C 0 19.35000 11.20000 -13.36000 L

H 0 19.04000 10.26000 -13.52000 L

H 0 18.70000 11.65000 -12.74000 L

C 0 20.68000 11.13000 -12.67000 L

O 0 21.26000 12.17000 -12.30000 L

N 0 21.23000 9.94000 -12.54000 L

H 0 20.75000 9.13000 -12.90000 L

H 0 22.11000 9.84000 -12.09000 L

C 0 20.19000 13.33000 -14.65000 L

O 0 21.35000 13.40000 -15.16000 L

N 0 19.56000 14.40000 -14.11000 L

C 0 18.24000 14.49000 -13.41000 L

H 0 18.27000 13.99000 -12.55000 L

H 0 17.52000 14.13000 -13.99000 L

C 0 18.08000 16.03000 -13.17000 L

H 0 18.37000 16.29000 -12.26000 L

H 0 17.13000 16.31000 -13.31000 L

C 0 18.98000 16.65000 -14.23000 L

H 0 19.24000 17.58000 -13.97000 L

H 0 18.54000 16.66000 -15.13000 L

C 0 20.20000 15.70000 -14.21000 L

H 0 20.81000 15.69000 -15.01000 L

C 0 21.11000 16.06000 -13.04000 L

O 0 21.47000 17.25000 -12.89000 L

N 0 21.49000 15.08000 -12.21000 L

H 0 21.25000 14.13000 -12.43000 L

C 0 22.25000 15.36000 -10.96000 L

H 0 22.19000 16.36000 -10.86000 L

C 0 21.63000 14.65000 -9.73000 L

H 0 21.70000 13.66000 -9.87000 L

C 0 22.40000 14.93000 -8.45000 L

H 0 21.96000 14.45000 -7.69000 L

H 0 23.34000 14.62000 -8.55000 L

H 0 22.39000 15.92000 -8.27000 L

C 0 20.14000 15.02000 -9.60000 L

H 0 19.75000 14.56000 -8.80000 L

H 0 20.05000 16.01000 -9.48000 L

H 0 19.65000 14.75000 -10.42000 L

C 0 23.70000 14.96000 -11.09000 L

O 0 24.01000 13.75000 -11.19000 L

N 0 24.58000 15.97000 -11.21000 L

H 0 24.28000 16.92000 -11.19000 L

C 0 26.02000 15.62000 -11.37000 L

H 0 26.38000 16.16000 -12.13000 L

H 0 26.07000 14.64000 -11.59000 L

C 0 26.85000 15.88000 -10.14000 L

O 0 26.42000 16.58000 -9.22000 L

N 0 28.09000 15.37000 -10.13000 L

H 0 28.38000 14.77000 -10.88000 L

C 0 29.03000 15.69000 -9.06000 L

H 0 28.51000 15.87000 -8.22000 L

C 0 29.97000 14.50000 -8.73000 L

H 0 29.36000 13.80000 -8.34000 L

C 0 30.72000 13.91000 -9.99000 L

H 0 31.30000 13.15000 -9.71000 L

H 0 30.05000 13.58000 -10.66000 L

H 0 31.28000 14.62000 -10.41000 L

C 0 31.00000 14.86000 -7.68000 L

H 0 31.46000 15.69000 -8.00000 L

H 0 31.66000 14.11000 -7.65000 L

C 0 30.37000 15.11000 -6.19000 L

H 0 31.10000 15.34000 -5.55000 L

H 0 29.71000 15.86000 -6.23000 L

H 0 29.91000 14.28000 -5.88000 L

C 0 29.78000 16.94000 -9.54000 L

O 0 30.31000 16.96000 -10.65000 L

N 0 29.76000 17.99000 -8.73000 L

H 0 29.27000 17.95000 -7.86000 L

C 0 30.47000 19.25000 -9.11000 L

H 0 30.14000 19.48000 -10.03000 L

C 0 30.13000 20.35000 -8.11000 L

H 0 30.61000 21.19000 -8.37000 L

H 0 29.15000 20.51000 -8.11000 L

H 0 30.42000 20.07000 -7.20000 L

C 0 32.01000 19.04000 -9.11000 L

O 0 32.56000 18.16000 -8.39000 L

N 0 32.69000 19.90000 -9.85000 L

H 0 32.20000 20.53000 -10.45000 L

C 0 34.15000 19.94000 -9.79000 L

H 0 34.44000 19.03000 -10.09000 L

C 0 34.68000 21.09000 -10.71000 L

H 0 35.68000 21.12000 -10.68000 L

H 0 34.39000 20.93000 -11.66000 L

H 0 34.32000 21.97000 -10.40000 L

C 0 34.64000 20.14000 -8.39000 L

O 0 33.96000 20.79000 -7.56000 L

N 0 35.86000 19.63000 -8.13000 L

H 0 36.32000 19.08000 -8.83000 L

C 0 36.52000 19.86000 -6.81000 L

H 0 36.00000 20.54000 -6.29000 L

H 0 37.45000 20.19000 -6.96000 L

C 0 36.54000 18.52000 -6.07000 L H 204

O 0 37.44000 18.27000 -5.24000 L

N 0 36.54000 15.32000 -7.33000 L H 222

H 0 35.80000 15.76000 -7.83000 L

C 0 37.52000 14.45000 -8.03000 L

H 0 38.07000 13.98000 -7.35000 L

C 0 36.77000 13.47000 -8.89000 L

H 0 36.35000 13.99000 -9.63000 L

H 0 36.05000 13.07000 -8.32000 L

C 0 37.71000 12.32000 -9.49000 L

O 0 38.89000 12.37000 -9.21000 L

O 0 37.20000 11.41000 -10.19000 L

C 0 38.38000 15.33000 -8.96000 L

O 0 38.11000 15.41000 -10.16000 L

N 0 39.36000 16.02000 -8.39000 L

H 0 39.56000 15.86000 -7.42000 L

C 0 40.17000 17.00000 -9.13000 L

H 0 39.52000 17.60000 -9.60000 L

C 0 41.10000 17.76000 -8.14000 L

H 0 41.88000 18.11000 -8.66000 L

H 0 41.42000 17.10000 -7.46000 L

C 0 40.32000 18.97000 -7.41000 L

H 0 39.40000 18.69000 -7.14000 L

H 0 40.26000 19.77000 -8.01000 L

C 0 41.07000 19.40000 -6.15000 L

H 0 42.00000 19.66000 -6.40000 L

H 0 41.09000 18.63000 -5.52000 L

C 0 40.33000 20.62000 -5.47000 L

H 0 40.74000 20.84000 -4.59000 L

H 0 39.36000 20.43000 -5.35000 L

N 0 40.51000 21.81000 -6.45000 L

H 0 40.06000 22.62000 -6.07000 L

H 0 41.49000 21.99000 -6.57000 L

H 0 40.10000 21.58000 -7.33000 L

C 0 41.03000 16.32000 -10.16000 L

O 0 41.34000 16.96000 -11.17000 L

N 0 41.44000 15.06000 -9.92000 L

H 0 41.04000 14.53000 -9.17000 L

C 0 42.51000 14.49000 -10.79000 L

H 0 42.77000 15.22000 -11.42000 L

C 0 43.78000 14.05000 -9.96000 L

H 0 44.58000 14.07000 -10.55000 L

H 0 43.64000 13.11000 -9.62000 L

C 0 44.08000 14.91000 -8.75000 L

N 0 44.38000 16.27000 -8.85000 L

C 0 44.58000 16.76000 -7.63000 L

H 0 44.88000 17.68000 -7.41000 L

N 0 44.30000 15.79000 -6.74000 L

H 0 44.31000 15.90000 -5.75000 L

C 0 44.01000 14.64000 -7.42000 L

H 0 43.79000 13.75000 -7.00000 L

C 0 41.93000 13.29000 -11.56000 L

O 0 42.67000 12.52000 -12.14000 L

N 0 40.61000 13.17000 -11.69000 L

H 0 39.99000 13.81000 -11.21000 L

C 0 40.06000 12.11000 -12.53000 L

H 0 40.49000 12.19000 -13.43000 L

H 0 39.08000 12.26000 -12.62000 L

C 0 40.31000 10.71000 -11.98000 L

O 0 40.44000 9.75000 -12.76000 L

N 0 40.28000 10.55000 -10.66000 L

H 0 40.12000 11.34000 -10.07000 L

C 0 40.50000 9.20000 -10.02000 L

H 0 40.94000 8.71000 -10.76000 L

C 0 41.31000 9.37000 -8.69000 L

H 0 41.54000 8.48000 -8.30000 L

H 0 40.80000 9.91000 -8.02000 L

C 0 42.56000 10.06000 -8.96000 L

H 0 42.34000 10.87000 -9.51000 L

H 0 43.14000 9.45000 -9.49000 L

C 0 43.26000 10.48000 -7.69000 L

O 0 42.86000 11.44000 -6.99000 L

O 0 44.25000 9.82000 -7.38000 L

C 0 39.30000 8.35000 -9.71000 L

O 0 39.44000 7.17000 -9.47000 L

N 0 38.09000 8.88000 -9.73000 L

H 0 37.92000 9.77000 -10.17000 L

C 0 36.99000 8.14000 -9.09000 L

H 0 37.31000 7.20000 -8.99000 L

C 0 36.63000 8.79000 -7.67000 L

H 0 35.88000 8.27000 -7.25000 L

H 0 37.43000 8.76000 -7.08000 L

H 0 36.34000 9.74000 -7.81000 L

C 0 35.79000 8.17000 -9.99000 L

O 0 34.66000 8.02000 -9.54000 L

N 0 36.02000 8.39000 -11.30000 L

H 0 36.95000 8.36000 -11.65000 L

C 0 34.92000 8.69000 -12.21000 L

H 0 34.51000 9.53000 -11.85000 L

C 0 35.41000 8.90000 -13.67000 L

H 0 35.94000 8.10000 -13.96000 L

C 0 34.19000 9.06000 -14.64000 L

H 0 34.53000 9.19000 -15.58000 L

H 0 33.62000 8.24000 -14.61000 L

H 0 33.65000 9.85000 -14.37000 L

C 0 36.35000 10.09000 -13.74000 L

H 0 36.65000 10.21000 -14.69000 L

H 0 35.87000 10.91000 -13.44000 L

H 0 37.14000 9.92000 -13.15000 L

C 0 33.92000 7.55000 -12.19000 L

O 0 32.72000 7.74000 -12.04000 L

N 0 34.41000 6.31000 -12.40000 L

H 0 35.39000 6.16000 -12.53000 L

C 0 33.48000 5.20000 -12.42000 L

H 0 32.82000 5.49000 -13.12000 L

C 0 34.24000 3.88000 -12.79000 L

H 0 33.63000 3.09000 -12.67000 L

H 0 35.04000 3.77000 -12.20000 L

C 0 34.70000 3.92000 -14.25000 L

O 0 35.89000 4.01000 -14.46000 L

O 0 33.88000 4.06000 -15.15000 L

C 0 32.72000 4.99000 -11.09000 L

O 0 31.55000 4.67000 -11.12000 L

N 0 33.39000 5.20000 -9.95000 L

H 0 34.34000 5.49000 -9.98000 L

C 0 32.70000 5.01000 -8.65000 L

H 0 33.38000 5.02000 -7.92000 L

H 0 32.24000 4.12000 -8.66000 L

C 0 31.70000 6.11000 -8.46000 L

O 0 30.69000 5.88000 -7.82000 L

N 0 31.92000 7.30000 -9.04000 L

H 0 32.80000 7.48000 -9.49000 L

C 0 30.86000 8.39000 -9.01000 L

H 0 30.51000 8.47000 -8.08000 L

C 0 31.48000 9.76000 -9.45000 L

H 0 30.76000 10.45000 -9.53000 L

H 0 31.95000 9.66000 -10.32000 L

C 0 32.49000 10.19000 -8.37000 L

H 0 32.99000 9.39000 -8.03000 L

C 0 33.50000 11.23000 -8.95000 L

H 0 34.15000 11.50000 -8.24000 L

H 0 33.99000 10.82000 -9.72000 L

H 0 33.00000 12.04000 -9.27000 L

C 0 31.67000 10.85000 -7.24000 L

H 0 32.29000 11.15000 -6.51000 L

H 0 31.18000 11.65000 -7.60000 L

H 0 31.01000 10.20000 -6.87000 L

C 0 29.66000 8.02000 -9.86000 L

O 0 28.55000 8.20000 -9.42000 L

N 0 29.83000 7.42000 -11.04000 L

H 0 30.74000 7.28000 -11.44000 L

C 0 28.59000 6.96000 -11.71000 L

H 0 28.06000 7.80000 -11.83000 L

C 0 28.87000 6.27000 -13.06000 L

H 0 28.03000 5.84000 -13.39000 L

H 0 29.58000 5.58000 -12.94000 L

C 0 29.34000 7.30000 -14.09000 L

C 0 30.57000 7.17000 -14.69000 L

H 0 31.14000 6.37000 -14.48000 L

C 0 31.05000 8.13000 -15.60000 L

H 0 31.96000 8.02000 -16.02000 L

C 0 30.27000 9.24000 -15.91000 L

O 0 30.77000 10.19000 -16.79000 L

H 0 30.09000 10.92000 -16.91000 L

C 0 29.03000 9.43000 -15.31000 L

H 0 28.48000 10.24000 -15.52000 L

C 0 28.56000 8.43000 -14.37000 L

H 0 27.67000 8.55000 -13.92000 L

C 0 27.91000 5.89000 -10.84000 L

O 0 26.65000 5.83000 -10.79000 L

N 0 28.72000 4.97000 -10.27000 L

H 0 29.71000 4.98000 -10.41000 L

C 0 28.08000 3.96000 -9.43000 L

H 0 27.44000 3.49000 -10.03000 L

C 0 29.19000 3.04000 -8.89000 L

H 0 29.85000 3.59000 -8.38000 L

H 0 29.64000 2.60000 -9.66000 L

C 0 28.66000 1.97000 -7.97000 L

H 0 27.94000 1.46000 -8.44000 L

H 0 28.29000 2.39000 -7.15000 L

C 0 29.78000 1.01000 -7.58000 L

H 0 30.30000 1.40000 -6.82000 L

H 0 30.39000 0.87000 -8.37000 L

C 0 29.21000 -0.34000 -7.15000 L

H 0 28.88000 -0.84000 -7.96000 L

H 0 28.45000 -0.20000 -6.51000 L

N 0 30.33000 -1.14000 -6.47000 L

H 0 29.98000 -2.03000 -6.18000 L

H 0 31.09000 -1.27000 -7.12000 L

H 0 30.67000 -0.63000 -5.68000 L

C 0 27.29000 4.58000 -8.22000 L

O 0 26.29000 3.98000 -7.75000 L

N 0 27.73000 5.75000 -7.70000 L

H 0 28.60000 6.12000 -8.03000 L

C 0 26.97000 6.49000 -6.66000 L

H 0 26.82000 5.85000 -5.91000 L

C 0 27.79000 7.71000 -6.25000 L

H 0 27.53000 8.49000 -6.82000 L

H 0 28.76000 7.52000 -6.40000 L

C 0 27.61000 8.11000 -4.82000 L

H 0 27.63000 7.24000 -4.32000 L

H 0 26.69000 8.48000 -4.79000 L

S 0 28.83000 9.26000 -4.20000 L

C 0 30.27000 8.37000 -4.25000 L

H 0 31.02000 8.93000 -3.92000 L

H 0 30.18000 7.56000 -3.67000 L

H 0 30.45000 8.08000 -5.19000 L

C 0 25.61000 7.00000 -7.18000 L

O 0 24.72000 7.37000 -6.39000 L

N 0 25.43000 7.06000 -8.49000 L

H 0 26.17000 6.86000 -9.12000 L

C 0 24.06000 7.43000 -8.98000 L

H 0 23.45000 7.49000 -8.19000 L

H 0 23.74000 6.71000 -9.59000 L

C 0 24.08000 8.78000 -9.72000 L

O 0 23.04000 9.23000 -10.16000 L

N 0 25.26000 9.43000 -9.86000 L

H 0 26.09000 9.03000 -9.46000 L

C 0 25.35000 10.71000 -10.58000 L

H 0 24.64000 11.30000 -10.19000 L

C 0 26.72000 11.40000 -10.42000 L

H 0 26.78000 12.16000 -11.07000 L

H 0 27.44000 10.73000 -10.62000 L

C 0 26.92000 11.93000 -9.02000 L

C 0 27.81000 11.30000 -8.13000 L

H 0 28.30000 10.48000 -8.43000 L

C 0 28.01000 11.82000 -6.80000 L

H 0 28.66000 11.39000 -6.17000 L

C 0 27.27000 12.96000 -6.40000 L

H 0 27.42000 13.35000 -5.49000 L

C 0 26.32000 13.57000 -7.25000 L

H 0 25.77000 14.35000 -6.95000 L

C 0 26.15000 13.02000 -8.59000 L

H 0 25.48000 13.43000 -9.21000 L

C 0 25.07000 10.47000 -12.02000 L

O 0 25.54000 9.48000 -12.60000 L

N 0 24.31000 11.36000 -12.62000 L

H 0 23.96000 12.13000 -12.09000 L

C 0 23.95000 11.27000 -14.04000 L

H 0 23.13000 11.82000 -14.19000 L

H 0 23.76000 10.32000 -14.26000 L

C 0 25.07000 11.77000 -14.91000 L

O 0 25.07000 11.53000 -16.14000 L

N 0 26.03000 12.49000 -14.31000 L

H 0 25.97000 12.71000 -13.34000 L

C 0 27.18000 12.94000 -15.12000 L

H 0 27.42000 12.15000 -15.69000 L

C 0 26.92000 14.10000 -16.10000 L

H 0 26.22000 13.81000 -16.74000 L

H 0 27.77000 14.29000 -16.59000 L

C 0 26.46000 15.43000 -15.46000 L

C 0 27.28000 16.55000 -15.46000 L

H 0 28.21000 16.47000 -15.83000 L

C 0 26.85000 17.78000 -14.95000 L

H 0 27.48000 18.56000 -14.94000 L

C 0 25.56000 17.91000 -14.46000 L

H 0 25.24000 18.81000 -14.14000 L

C 0 24.71000 16.82000 -14.40000 L

H 0 23.81000 16.90000 -13.97000 L

C 0 25.12000 15.56000 -14.97000 L

H 0 24.48000 14.79000 -15.01000 L

C 0 28.26000 13.32000 -14.13000 L

O 0 27.95000 13.58000 -12.97000 L

N 0 29.51000 13.19000 -14.57000 L

H 0 29.68000 12.91000 -15.52000 L

C 0 30.63000 13.46000 -13.67000 L

H 0 30.26000 13.75000 -12.79000 L

C 0 31.49000 12.18000 -13.54000 L

H 0 31.70000 11.80000 -14.44000 L

C 0 32.87000 12.52000 -12.78000 L

H 0 33.42000 11.68000 -12.69000 L

H 0 33.38000 13.20000 -13.30000 L

H 0 32.67000 12.88000 -11.87000 L

C 0 30.66000 11.15000 -12.75000 L

H 0 31.19000 10.31000 -12.64000 L

H 0 30.44000 11.52000 -11.85000 L

H 0 29.82000 10.95000 -13.25000 L

C 0 31.46000 14.57000 -14.22000 L

O 0 31.78000 14.54000 -15.41000 L

N 0 31.81000 15.56000 -13.38000 L

H 0 31.42000 15.63000 -12.46000 L

C 0 32.79000 16.55000 -13.86000 L

H 0 32.95000 16.39000 -14.84000 L

C 0 32.23000 17.95000 -13.62000 L

H 0 32.12000 18.06000 -12.63000 L

H 0 31.33000 17.99000 -14.06000 L

C 0 33.09000 19.13000 -14.17000 L

H 0 33.34000 18.95000 -15.12000 L

H 0 33.93000 19.21000 -13.62000 L

C 0 32.36000 20.46000 -14.12000 L

O 0 32.96000 21.40000 -13.55000 L

O 0 31.18000 20.52000 -14.62000 L

C 0 34.06000 16.38000 -13.02000 L

O 0 33.98000 16.36000 -11.78000 L

N 0 35.23000 16.26000 -13.66000 L

H 0 35.25000 16.19000 -14.65000 L

C 0 36.48000 16.23000 -12.91000 L

H 0 36.15000 16.07000 -11.98000 L

C 0 37.43000 15.12000 -13.39000 L

H 0 38.25000 15.11000 -12.82000 L

C 0 36.66000 13.74000 -13.32000 L

H 0 37.26000 13.01000 -13.63000 L

H 0 36.37000 13.56000 -12.38000 L

H 0 35.85000 13.78000 -13.92000 L

C 0 37.80000 15.38000 -14.87000 L

H 0 37.87000 16.37000 -15.01000 L

H 0 37.08000 15.01000 -15.46000 L

C 0 39.11000 14.75000 -15.28000 L

H 0 39.30000 14.96000 -16.24000 L

H 0 39.85000 15.12000 -14.71000 L

H 0 39.06000 13.76000 -15.15000 L

C 0 37.21000 17.53000 -12.99000 L

O 0 36.89000 18.35000 -13.84000 L

N 0 38.24000 17.72000 -12.16000 L

H 0 38.49000 17.00000 -11.51000 L

C 0 39.00000 18.99000 -12.19000 L

H 0 38.76000 19.48000 -13.02000 L

H 0 39.98000 18.77000 -12.20000 L

C 0 38.67000 19.85000 -10.95000 L

O 0 37.92000 19.41000 -10.05000 L

N 0 39.21000 21.09000 -10.90000 L

H 0 39.12000 21.62000 -10.06000 L

C 0 39.94000 21.68000 -12.05000 L

H 0 39.37000 21.46000 -12.84000 L

C 0 40.12000 23.20000 -11.79000 L

H 0 40.70000 23.61000 -12.50000 L

H 0 40.52000 23.37000 -10.89000 L

O 0 38.85000 23.88000 -11.84000 L

H 0 38.99000 24.86000 -11.67000 L

C 0 41.34000 21.08000 -12.23000 L

O 0 42.00000 20.71000 -11.26000 L

N 0 41.75000 20.93000 -13.48000 L

H 0 41.12000 21.17000 -14.23000 L

C 0 43.06000 20.44000 -13.81000 L

H 0 43.40000 20.08000 -12.94000 L

C 0 42.98000 19.35000 -14.90000 L

H 0 42.72000 19.80000 -15.76000 L

C 0 44.42000 18.65000 -15.09000 L

H 0 44.36000 17.94000 -15.80000 L

H 0 45.09000 19.33000 -15.36000 L

H 0 44.70000 18.22000 -14.23000 L

C 0 41.83000 18.31000 -14.56000 L

H 0 41.79000 17.61000 -15.27000 L

H 0 42.03000 17.87000 -13.68000 L

H 0 40.95000 18.79000 -14.50000 L

C 0 43.93000 21.58000 -14.38000 L

O 0 43.47000 22.35000 -15.24000 L

N 0 45.18000 21.71000 -13.91000 L

H 0 45.53000 21.06000 -13.24000 L

C 0 46.02000 22.80000 -14.38000 L

H 0 45.39000 23.45000 -14.80000 L

C 0 46.80000 23.48000 -13.22000 L

H 0 47.44000 24.16000 -13.58000 L

C 0 45.84000 24.11000 -12.20000 L

H 0 46.37000 24.54000 -11.47000 L

H 0 45.28000 24.80000 -12.66000 L

H 0 45.25000 23.40000 -11.82000 L

O 0 47.55000 22.49000 -12.49000 L

H 0 48.05000 22.93000 -11.75000 L

C 0 47.06000 22.17000 -15.37000 L

O 0 47.27000 20.94000 -15.38000 L

N 0 47.68000 23.00000 -16.20000 L

C 0 47.33000 24.41000 -16.54000 L

H 0 47.81000 25.04000 -15.93000 L

H 0 46.34000 24.55000 -16.45000 L

C 0 47.82000 24.54000 -18.06000 L

H 0 48.06000 25.48000 -18.29000 L

H 0 47.11000 24.21000 -18.70000 L

C 0 49.11000 23.59000 -18.08000 L

H 0 49.90000 24.02000 -17.66000 L

H 0 49.34000 23.29000 -19.01000 L

C 0 48.62000 22.39000 -17.22000 L

H 0 48.14000 21.70000 -17.77000 L

C 0 49.77000 21.59000 -16.59000 L

O 0 49.89000 20.41000 -16.87000 L

N 0 50.53000 22.20000 -15.67000 L

H 0 50.35000 23.16000 -15.46000 L

C 0 51.59000 21.52000 -14.97000 L

H 0 51.84000 20.74000 -15.54000 L

C 0 52.83000 22.46000 -14.76000 L

H 0 53.57000 21.92000 -14.38000 L

H 0 52.57000 23.17000 -14.10000 L

C 0 53.34000 23.17000 -16.05000 L

H 0 54.24000 23.58000 -15.88000 L

H 0 52.69000 23.89000 -16.32000 L

C 0 53.47000 22.14000 -17.22000 L

H 0 52.60000 21.66000 -17.29000 L

H 0 54.19000 21.49000 -16.96000 L

C 0 53.84000 22.79000 -18.67000 L

H 0 54.71000 22.41000 -19.00000 L

H 0 53.92000 23.78000 -18.59000 L

N 0 52.75000 22.48000 -19.75000 L

H 0 53.02000 22.90000 -20.62000 L

H 0 52.66000 21.49000 -19.86000 L

H 0 51.87000 22.86000 -19.46000 L

C 0 51.03000 21.13000 -13.61000 L

O 0 50.12000 21.80000 -13.09000 L

N 0 51.58000 20.06000 -13.03000 L

C 0 52.66000 19.21000 -13.63000 L

H 0 53.51000 19.73000 -13.71000 L

H 0 52.38000 18.89000 -14.54000 L

C 0 52.82000 18.05000 -12.67000 L

H 0 53.79000 17.82000 -12.58000 L

H 0 52.32000 17.26000 -13.01000 L

C 0 52.22000 18.58000 -11.28000 L

H 0 52.95000 18.92000 -10.68000 L

H 0 51.72000 17.85000 -10.81000 L

C 0 51.25000 19.74000 -11.64000 L

H 0 50.28000 19.53000 -11.58000 L

C 0 51.55000 20.92000 -10.69000 L

O 0 52.50000 21.68000 -10.88000 L

N 0 50.77000 21.05000 -9.65000 L

H 0 49.94000 20.50000 -9.56000 L

C 0 51.14000 22.02000 -8.62000 L

H 0 52.13000 21.94000 -8.49000 L

C 0 50.82000 23.45000 -9.06000 L

H 0 51.30000 23.63000 -9.93000 L

H 0 51.17000 24.08000 -8.36000 L

C 0 49.26000 23.67000 -9.26000 L

H 0 48.79000 23.39000 -8.42000 L

H 0 48.95000 23.12000 -10.03000 L

C 0 48.88000 25.14000 -9.54000 L

O 0 49.57000 25.83000 -10.25000 L

N 0 47.73000 25.60000 -9.01000 L

H 0 47.17000 24.99000 -8.45000 L

H 0 47.44000 26.54000 -9.17000 L

C 0 50.42000 21.67000 -7.32000 L

O 0 49.46000 20.91000 -7.31000 L

N 0 51.01000 22.08000 -6.22000 L

H 0 51.75000 22.75000 -6.26000 L

C 0 50.56000 21.54000 -4.95000 L

H 0 50.33000 20.61000 -5.21000 L

C 0 51.68000 21.66000 -3.88000 L

H 0 51.29000 21.58000 -2.97000 L

H 0 52.14000 22.55000 -3.97000 L

C 0 52.73000 20.56000 -4.06000 L

H 0 53.09000 20.61000 -4.99000 L

H 0 52.28000 19.67000 -3.93000 L

C 0 53.88000 20.70000 -3.05000 L

O 0 53.94000 21.74000 -2.31000 L

O 0 54.71000 19.76000 -3.00000 L

C 0 49.33000 22.26000 -4.35000 L

O 0 48.69000 21.67000 -3.47000 L

N 0 49.08000 23.47000 -4.77000 L

H 0 49.64000 23.85000 -5.51000 L

C 0 47.97000 24.32000 -4.19000 L

H 0 47.17000 23.74000 -4.01000 L

H 0 47.72000 25.03000 -4.85000 L

C 0 48.45000 24.97000 -2.88000 L

O 0 49.70000 25.03000 -2.61000 L

N 0 47.53000 25.53000 -2.07000 L

H 0 46.56000 25.46000 -2.31000 L

C 0 47.94000 26.27000 -0.82000 L

H 0 48.75000 26.81000 -1.07000 L

C 0 46.77000 27.15000 -0.31000 L

H 0 46.98000 27.51000 0.60000 L

H 0 45.93000 26.61000 -0.27000 L

C 0 46.53000 28.28000 -1.20000 L

O 0 47.50000 28.90000 -1.59000 L

N 0 45.27000 28.55000 -1.64000 L

H 0 44.51000 27.96000 -1.35000 L

H 0 45.10000 29.32000 -2.25000 L

C 0 48.39000 25.37000 0.27000 L

O 0 48.06000 24.21000 0.30000 L

N 0 49.12000 25.92000 1.26000 L

C 0 49.58000 27.30000 1.31000 L

H 0 48.80000 27.92000 1.25000 L

H 0 50.21000 27.49000 0.56000 L

C 0 50.27000 27.39000 2.69000 L

H 0 49.76000 28.00000 3.30000 L

H 0 51.21000 27.73000 2.59000 L

C 0 50.33000 26.03000 3.32000 L

H 0 49.97000 26.08000 4.25000 L

H 0 51.28000 25.72000 3.34000 L

C 0 49.47000 25.08000 2.46000 L

H 0 49.90000 24.22000 2.17000 L

C 0 48.27000 24.66000 3.26000 L

O 0 47.28000 25.40000 3.24000 L

N 0 48.37000 23.58000 4.04000 L

H 0 49.25000 23.10000 4.06000 L

C 0 47.30000 23.04000 4.84000 L

H 0 46.46000 23.31000 4.37000 L

C 0 47.44000 21.56000 4.93000 L

H 0 46.74000 21.19000 5.54000 L

H 0 48.34000 21.33000 5.30000 L

C 0 47.30000 20.93000 3.66000 L

H 0 48.07000 21.18000 3.07000 L

H 0 46.44000 21.20000 3.23000 L

C 0 47.31000 19.48000 3.95000 L

H 0 46.44000 19.20000 4.34000 L

H 0 48.05000 19.26000 4.58000 L

N 0 47.51000 18.77000 2.73000 L

H 0 47.92000 19.26000 1.96000 L

C 0 47.20000 17.48000 2.55000 L

N 0 46.62000 16.75000 3.55000 L

H 0 46.43000 17.17000 4.44000 L

H 0 46.39000 15.79000 3.39000 L

N 0 47.45000 16.94000 1.35000 L

H 0 47.86000 17.50000 0.63000 L

H 0 47.22000 15.98000 1.18000 L

C 0 47.36000 23.58000 6.26000 L

O 0 48.45000 23.87000 6.75000 L

N 0 46.22000 23.76000 6.92000 L

C 0 46.27000 24.13000 8.36000 L

H 0 46.86000 24.92000 8.52000 L

H 0 46.60000 23.36000 8.92000 L

C 0 44.81000 24.44000 8.63000 L

H 0 44.55000 25.34000 8.30000 L

H 0 44.58000 24.36000 9.61000 L

C 0 44.14000 23.38000 7.86000 L

H 0 43.16000 23.57000 7.77000 L

H 0 44.26000 22.49000 8.29000 L

C 0 44.87000 23.48000 6.50000 L

H 0 44.87000 22.63000 5.97000 L

C 0 44.23000 24.47000 5.49000 L

O 0 44.56000 25.66000 5.42000 L

N 0 43.44000 23.92000 4.55000 L

H 0 43.21000 22.95000 4.59000 L

C 0 42.93000 24.77000 3.48000 L

H 0 42.91000 25.72000 3.79000 L

C 0 43.89000 24.61000 2.22000 L

H 0 44.79000 24.97000 2.44000 L

H 0 43.50000 25.11000 1.45000 L

C 0 44.04000 23.10000 1.81000 L

H 0 43.20000 22.80000 1.35000 L

H 0 44.18000 22.55000 2.63000 L

C 0 45.30000 22.96000 0.82000 L

H 0 46.14000 23.30000 1.25000 L

H 0 45.15000 23.44000 -0.04000 L

N 0 45.51000 21.53000 0.52000 L

H 0 45.05000 20.84000 1.07000 L

C 0 46.29000 21.15000 -0.49000 L

N 0 46.37000 19.88000 -0.82000 L

H 0 45.85000 19.20000 -0.32000 L

H 0 46.96000 19.60000 -1.58000 L

N 0 46.97000 22.07000 -1.14000 L

H 0 46.89000 23.03000 -0.87000 L

H 0 47.57000 21.82000 -1.90000 L

C 0 41.49000 24.36000 3.18000 L H 224

O 0 40.93000 24.76000 2.17000 L

N 0 37.74000 23.77000 5.47000 L H 238

H 0 37.23000 24.06000 4.67000 L

C 0 37.18000 24.00000 6.81000 L

H 0 37.65000 23.38000 7.44000 L

C 0 37.38000 25.48000 7.24000 L

H 0 37.10000 25.60000 8.19000 L

H 0 36.84000 26.08000 6.65000 L

C 0 38.81000 25.90000 7.13000 L

C 0 39.29000 26.50000 5.98000 L

H 0 38.66000 26.72000 5.24000 L

C 0 40.64000 26.79000 5.87000 L

H 0 40.99000 27.19000 5.02000 L

C 0 41.53000 26.52000 6.93000 L

H 0 42.49000 26.76000 6.84000 L

C 0 41.09000 25.94000 8.08000 L

H 0 41.73000 25.74000 8.82000 L

C 0 39.71000 25.63000 8.20000 L

H 0 39.37000 25.22000 9.05000 L

C 0 35.69000 23.69000 6.77000 L H 240

O 0 34.97000 24.12000 5.85000 L

N 0 31.76000 24.01000 8.35000 L H 262

H 0 31.37000 23.63000 7.51000 L

C 0 30.95000 24.92000 9.16000 L

H 0 31.45000 25.16000 9.99000 L

C 0 30.61000 26.26000 8.43000 L

H 0 29.81000 26.66000 8.89000 L

H 0 30.36000 26.03000 7.49000 L

C 0 31.70000 27.38000 8.36000 L

H 0 31.98000 27.60000 9.29000 L

C 0 32.97000 26.85000 7.59000 L

H 0 33.66000 27.58000 7.55000 L

H 0 33.34000 26.06000 8.07000 L

H 0 32.71000 26.59000 6.66000 L

C 0 31.09000 28.68000 7.66000 L

H 0 31.79000 29.40000 7.62000 L

H 0 30.78000 28.46000 6.74000 L

H 0 30.31000 29.01000 8.20000 L

C 0 29.69000 24.10000 9.40000 L

O 0 28.65000 24.35000 8.75000 L

N 0 29.75000 23.13000 10.32000 L

C 0 30.93000 22.69000 11.12000 L

H 0 31.35000 23.47000 11.59000 L

H 0 31.62000 22.25000 10.54000 L

C 0 30.34000 21.70000 12.11000 L

H 0 30.14000 22.18000 12.97000 L

H 0 31.00000 20.97000 12.28000 L

C 0 28.99000 21.14000 11.41000 L

H 0 28.25000 21.01000 12.08000 L

H 0 29.16000 20.27000 10.93000 L

C 0 28.57000 22.22000 10.38000 L

H 0 28.40000 21.86000 9.46000 L

C 0 27.23000 22.96000 10.81000 L

O 0 26.18000 22.63000 10.35000 L

N 0 27.31000 23.98000 11.63000 L

H 0 28.20000 24.28000 11.96000 L

C 0 26.06000 24.71000 12.07000 L

H 0 25.42000 24.06000 12.47000 L

C 0 26.47000 25.79000 13.06000 L

H 0 25.67000 26.37000 13.22000 L

H 0 27.19000 26.33000 12.64000 L

C 0 26.97000 25.22000 14.44000 L

H 0 26.49000 24.36000 14.61000 L

H 0 26.74000 25.88000 15.15000 L

C 0 28.53000 24.95000 14.50000 L

O 0 28.90000 24.58000 15.63000 L

O 0 29.34000 25.04000 13.45000 L

C 0 25.43000 25.40000 10.87000 L

O 0 24.30000 25.89000 10.99000 L

N 0 26.19000 25.55000 9.74000 L

H 0 27.11000 25.18000 9.71000 L

C 0 25.64000 26.25000 8.59000 L

H 0 24.77000 26.61000 8.92000 L

C 0 26.57000 27.37000 8.14000 L

H 0 26.10000 27.92000 7.44000 L

H 0 27.40000 26.97000 7.74000 L

C 0 26.97000 28.29000 9.32000 L

O 0 26.06000 28.93000 9.86000 L

O 0 28.21000 28.44000 9.63000 L

C 0 25.43000 25.31000 7.40000 L

O 0 25.02000 25.75000 6.32000 L

N 0 25.79000 24.05000 7.58000 L

H 0 26.04000 23.76000 8.51000 L

C 0 25.87000 23.04000 6.50000 L

H 0 26.33000 22.23000 6.87000 L

C 0 24.43000 22.64000 6.02000 L

H 0 24.50000 22.03000 5.23000 L

H 0 23.93000 23.47000 5.76000 L

C 0 23.69000 21.90000 7.24000 L

H 0 23.73000 22.49000 8.05000 L

H 0 24.16000 21.04000 7.43000 L

C 0 22.20000 21.59000 6.93000 L

O 0 21.86000 21.10000 5.84000 L

N 0 21.32000 21.84000 7.91000 L

H 0 21.63000 22.21000 8.79000 L

H 0 20.34000 21.66000 7.77000 L

C 0 26.67000 23.65000 5.35000 L

O 0 26.25000 23.63000 4.19000 L

N 0 27.84000 24.17000 5.67000 L

H 0 28.19000 24.06000 6.60000 L

C 0 28.64000 24.89000 4.69000 L

H 0 28.27000 24.72000 3.78000 L

C 0 28.57000 26.40000 5.02000 L

H 0 29.11000 26.91000 4.35000 L

H 0 27.62000 26.71000 4.98000 L

H 0 28.93000 26.56000 5.94000 L

C 0 30.08000 24.37000 4.81000 L H 264

O 0 30.48000 23.78000 5.88000 L

N 0 34.24000 25.97000 3.77000 L H 278

H 0 34.61000 25.47000 4.56000 L

C 0 35.02000 27.01000 3.13000 L

H 0 34.47000 27.39000 2.39000 L

C 0 35.44000 28.11000 4.17000 L

H 0 35.80000 27.68000 5.00000 L

C 0 36.57000 29.09000 3.55000 L

H 0 36.82000 29.78000 4.23000 L

H 0 37.38000 28.55000 3.31000 L

H 0 36.22000 29.54000 2.73000 L

C 0 34.16000 28.91000 4.51000 L

H 0 33.46000 28.27000 4.81000 L

H 0 33.85000 29.38000 3.68000 L

C 0 34.43000 30.00000 5.66000 L

H 0 33.58000 30.49000 5.85000 L

H 0 34.73000 29.53000 6.49000 L

H 0 35.13000 30.64000 5.35000 L

C 0 36.32000 26.30000 2.68000 L

O 0 36.88000 25.49000 3.45000 L

N 0 36.71000 26.48000 1.42000 L

H 0 36.15000 27.00000 0.77000 L

C 0 37.98000 25.89000 1.01000 L

H 0 38.43000 25.60000 1.85000 L

C 0 37.73000 24.70000 0.06000 L

H 0 36.91000 24.20000 0.37000 L

H 0 38.52000 24.09000 0.08000 L

C 0 37.50000 25.18000 -1.38000 L

O 0 38.46000 25.36000 -2.16000 L

N 0 36.24000 25.44000 -1.73000 L

H 0 35.50000 25.32000 -1.07000 L

H 0 36.04000 25.76000 -2.66000 L

C 0 38.85000 26.90000 0.31000 L

O 0 38.34000 27.88000 -0.36000 L

N 0 40.18000 26.68000 0.43000 L

H 0 40.52000 25.95000 1.02000 L

C 0 41.11000 27.51000 -0.32000 L

H 0 40.59000 27.94000 -1.06000 L

C 0 41.69000 28.67000 0.52000 L

H 0 40.97000 29.34000 0.67000 L

H 0 42.43000 29.08000 -0.01000 L

C 0 42.27000 28.26000 1.92000 L

H 0 42.83000 27.44000 1.82000 L

H 0 41.52000 28.08000 2.55000 L

C 0 43.15000 29.43000 2.49000 L

H 0 42.61000 30.27000 2.56000 L

H 0 43.94000 29.59000 1.89000 L

N 0 43.66000 29.08000 3.84000 L

H 0 44.52000 28.58000 3.89000 L

C 0 43.05000 29.40000 4.99000 L

N 0 41.83000 29.96000 5.00000 L

H 0 41.36000 30.14000 4.13000 L

H 0 41.39000 30.18000 5.86000 L

N 0 43.62000 29.12000 6.16000 L

H 0 44.52000 28.68000 6.18000 L

H 0 43.16000 29.36000 7.01000 L

C 0 42.21000 26.56000 -0.80000 L H 280

O 0 43.42000 26.78000 -0.61000 L

N 0 43.39000 26.14000 -3.82000 L H 299

H 0 42.42000 26.36000 -3.83000 L

C 0 44.27000 26.85000 -4.75000 L

H 0 45.08000 27.14000 -4.23000 L

H 0 43.78000 27.65000 -5.08000 L

C 0 44.70000 25.95000 -5.94000 L

O 0 45.88000 25.96000 -6.36000 L

N 0 43.79000 25.13000 -6.46000 L

H 0 42.91000 25.04000 -6.00000 L

C 0 44.06000 24.36000 -7.69000 L

H 0 43.20000 23.87000 -7.87000 L

C 0 44.44000 25.28000 -8.88000 L

H 0 44.91000 24.72000 -9.56000 L

H 0 45.07000 25.98000 -8.53000 L

C 0 43.25000 26.02000 -9.58000 L

C 0 43.52000 27.13000 -10.42000 L

H 0 44.44000 27.51000 -10.47000 L

C 0 42.45000 27.73000 -11.21000 L

H 0 42.63000 28.46000 -11.88000 L

C 0 41.12000 27.22000 -10.99000 L

H 0 40.34000 27.68000 -11.43000 L

C 0 40.88000 26.13000 -10.20000 L

H 0 39.95000 25.78000 -10.11000 L

C 0 41.94000 25.50000 -9.51000 L

H 0 41.77000 24.67000 -8.98000 L

C 0 45.26000 23.41000 -7.45000 L

O 0 46.21000 23.38000 -8.26000 L

N 0 45.23000 22.66000 -6.37000 L

H 0 44.61000 22.89000 -5.62000 L

C 0 46.10000 21.50000 -6.27000 L

H 0 47.04000 21.83000 -6.32000 L

C 0 45.94000 20.81000 -4.93000 L

H 0 46.22000 21.43000 -4.20000 L

H 0 46.51000 19.99000 -4.91000 L

C 0 44.51000 20.40000 -4.67000 L

O 0 43.61000 21.21000 -4.76000 L

N 0 44.29000 19.14000 -4.35000 L

H 0 45.06000 18.50000 -4.30000 L

H 0 43.36000 18.82000 -4.18000 L

C 0 45.72000 20.55000 -7.43000 L

O 0 44.55000 20.19000 -7.62000 L

N 0 46.69000 20.17000 -8.24000 L

H 0 47.65000 20.40000 -8.04000 L

C 0 46.35000 19.39000 -9.45000 L

H 0 45.55000 18.84000 -9.21000 L

C 0 46.03000 20.36000 -10.61000 L

H 0 46.78000 21.01000 -10.76000 L

H 0 45.18000 20.87000 -10.44000 L

O 0 45.85000 19.56000 -11.81000 L

H 0 45.65000 20.16000 -12.58000 L

C 0 47.55000 18.47000 -9.85000 L

O 0 48.73000 18.91000 -9.82000 L

N 0 47.26000 17.21000 -10.17000 L

H 0 46.34000 16.87000 -9.98000 L

C 0 48.24000 16.32000 -10.79000 L

H 0 49.03000 16.30000 -10.17000 L

C 0 47.61000 14.95000 -10.91000 L

H 0 48.21000 14.37000 -11.46000 L

H 0 46.73000 15.04000 -11.37000 L

C 0 47.41000 14.31000 -9.57000 L

N 0 46.83000 13.06000 -9.41000 L

C 0 46.79000 12.78000 -8.12000 L

H 0 46.46000 11.92000 -7.73000 L

N 0 47.25000 13.82000 -7.44000 L

H 0 47.30000 13.88000 -6.44000 L

C 0 47.65000 14.79000 -8.32000 L

H 0 48.05000 15.68000 -8.09000 L

C 0 48.76000 16.82000 -12.16000 L

O 0 49.75000 16.27000 -12.69000 L

N 0 48.03000 17.77000 -12.76000 L

H 0 47.21000 18.13000 -12.32000 L

C 0 48.43000 18.30000 -14.06000 L

H 0 49.43000 18.23000 -14.12000 L

H 0 48.16000 19.26000 -14.09000 L

C 0 47.80000 17.56000 -15.23000 L

O 0 47.33000 16.42000 -15.12000 L

N 0 47.85000 18.19000 -16.41000 L

H 0 48.32000 19.07000 -16.49000 L

C 0 47.21000 17.60000 -17.58000 L

H 0 46.28000 17.47000 -17.24000 L

C 0 47.42000 18.55000 -18.82000 L

H 0 47.10000 18.08000 -19.64000 L

H 0 48.39000 18.76000 -18.91000 L

C 0 46.67000 19.90000 -18.75000 L

H 0 46.89000 20.36000 -17.89000 L

C 0 47.06000 20.85000 -19.92000 L

H 0 46.55000 21.71000 -19.84000 L

H 0 48.04000 21.03000 -19.89000 L

H 0 46.83000 20.41000 -20.79000 L

C 0 45.19000 19.62000 -18.77000 L

H 0 44.68000 20.48000 -18.72000 L

H 0 44.95000 19.14000 -19.62000 L

H 0 44.95000 19.05000 -17.99000 L

C 0 47.75000 16.22000 -17.99000 L

O 0 46.98000 15.35000 -18.35000 L

N 0 49.08000 16.05000 -17.98000 L

H 0 49.70000 16.77000 -17.66000 L

C 0 49.60000 14.73000 -18.50000 L

H 0 49.24000 14.64000 -19.43000 L

C 0 51.11000 14.71000 -18.57000 L

H 0 51.47000 13.82000 -18.83000 L

H 0 51.53000 15.00000 -17.71000 L

O 0 51.45000 15.64000 -19.55000 L

H 0 52.44000 15.67000 -19.65000 L

C 0 49.10000 13.54000 -17.68000 L

O 0 48.61000 12.50000 -18.21000 L

N 0 49.19000 13.68000 -16.37000 L

H 0 49.58000 14.51000 -15.97000 L

C 0 48.71000 12.63000 -15.52000 L

H 0 49.25000 11.86000 -15.86000 L

C 0 48.99000 12.92000 -14.00000 L

H 0 48.74000 13.85000 -13.77000 L

C 0 48.14000 11.94000 -13.12000 L

H 0 48.31000 12.12000 -12.16000 L

H 0 47.17000 12.09000 -13.32000 L

H 0 48.38000 11.00000 -13.34000 L

C 0 50.51000 12.78000 -13.71000 L

H 0 50.68000 12.96000 -12.74000 L

H 0 50.81000 11.85000 -13.93000 L

H 0 51.01000 13.44000 -14.26000 L

C 0 47.23000 12.35000 -15.73000 L

O 0 46.86000 11.20000 -15.88000 L

N 0 46.38000 13.39000 -15.72000 L

H 0 46.73000 14.32000 -15.62000 L

C 0 44.95000 13.17000 -15.86000 L

H 0 44.68000 12.51000 -15.16000 L

C 0 44.18000 14.53000 -15.67000 L

H 0 44.48000 15.25000 -16.30000 L

C 0 42.68000 14.31000 -16.01000 L

H 0 42.19000 15.18000 -15.89000 L

H 0 42.59000 14.01000 -16.96000 L

H 0 42.29000 13.62000 -15.40000 L

C 0 44.44000 14.97000 -14.18000 L

H 0 43.97000 15.83000 -14.00000 L

H 0 44.09000 14.27000 -13.56000 L

H 0 45.42000 15.09000 -14.03000 L

C 0 44.68000 12.64000 -17.26000 L

O 0 43.86000 11.75000 -17.43000 L

N 0 45.48000 13.10000 -18.25000 L

H 0 46.18000 13.78000 -18.06000 L

C 0 45.29000 12.57000 -19.62000 L

H 0 44.34000 12.80000 -19.84000 L

C 0 46.29000 13.25000 -20.59000 L

H 0 47.21000 13.10000 -20.22000 L

H 0 46.08000 14.22000 -20.60000 L

C 0 46.22000 12.67000 -22.12000 L

H 0 45.57000 13.20000 -22.66000 L

H 0 45.94000 11.71000 -22.11000 L

C 0 47.67000 12.79000 -22.75000 L

O 0 48.36000 13.72000 -22.39000 L

O 0 48.19000 11.91000 -23.43000 L

C 0 45.49000 11.03000 -19.68000 L

O 0 44.64000 10.29000 -20.26000 L

N 0 46.61000 10.56000 -19.09000 L

H 0 47.26000 11.17000 -18.63000 L

C 0 46.81000 9.11000 -19.14000 L

H 0 46.71000 8.83000 -20.09000 L

C 0 48.24000 8.81000 -18.65000 L

H 0 48.34000 7.82000 -18.50000 L

H 0 48.42000 9.30000 -17.80000 L

C 0 49.23000 9.24000 -19.70000 L

N 0 50.06000 10.35000 -19.56000 L

C 0 50.75000 10.53000 -20.68000 L

H 0 51.43000 11.25000 -20.84000 L

N 0 50.40000 9.59000 -21.54000 L

H 0 50.79000 9.47000 -22.46000 L

C 0 49.41000 8.80000 -20.98000 L

H 0 48.91000 8.06000 -21.42000 L

C 0 45.75000 8.35000 -18.36000 L

O 0 45.32000 7.29000 -18.72000 L

N 0 45.33000 8.91000 -17.24000 L

H 0 45.72000 9.78000 -16.94000 L

C 0 44.31000 8.28000 -16.43000 L

H 0 44.61000 7.34000 -16.27000 L

C 0 44.17000 9.14000 -15.10000 L

H 0 43.64000 9.97000 -15.29000 L

H 0 45.08000 9.39000 -14.78000 L

C 0 43.49000 8.48000 -13.93000 L

H 0 43.86000 7.55000 -13.85000 L

H 0 42.51000 8.44000 -14.13000 L

C 0 43.75000 9.30000 -12.60000 L

H 0 43.08000 9.04000 -11.90000 L

H 0 43.68000 10.28000 -12.78000 L

N 0 45.04000 9.12000 -11.98000 L

H 0 45.47000 8.21000 -12.04000 L

C 0 45.69000 10.07000 -11.35000 L

N 0 45.20000 11.35000 -11.33000 L

H 0 44.34000 11.56000 -11.79000 L

H 0 45.70000 12.07000 -10.85000 L

N 0 46.82000 9.80000 -10.75000 L

H 0 47.18000 8.87000 -10.77000 L

H 0 47.32000 10.52000 -10.27000 L

C 0 42.99000 8.16000 -17.21000 L

O 0 42.27000 7.14000 -17.16000 L

N 0 42.59000 9.20000 -17.92000 L

H 0 43.12000 10.05000 -17.92000 L

C 0 41.39000 9.08000 -18.70000 L

H 0 40.78000 8.64000 -18.04000 L

C 0 40.89000 10.47000 -19.19000 L

H 0 40.11000 10.32000 -19.80000 L

H 0 41.63000 10.91000 -19.70000 L

C 0 40.44000 11.41000 -17.99000 L

H 0 41.22000 11.64000 -17.41000 L

C 0 39.86000 12.74000 -18.59000 L

H 0 39.57000 13.34000 -17.85000 L

H 0 40.56000 13.19000 -19.14000 L

H 0 39.07000 12.52000 -19.17000 L

C 0 39.37000 10.67000 -17.08000 L

H 0 39.10000 11.27000 -16.33000 L

H 0 38.57000 10.44000 -17.63000 L

H 0 39.78000 9.83000 -16.71000 L

C 0 41.57000 8.19000 -19.93000 L

O 0 40.62000 7.48000 -20.34000 L

N 0 42.75000 8.23000 -20.54000 L

H 0 43.48000 8.85000 -20.23000 L

C 0 42.95000 7.32000 -21.71000 L

H 0 42.22000 7.55000 -22.35000 L

C 0 44.33000 7.55000 -22.33000 L

H 0 44.55000 6.73000 -22.86000 L

H 0 44.98000 7.66000 -21.58000 L

C 0 44.42000 8.83000 -23.28000 L

H 0 44.17000 9.65000 -22.77000 L

H 0 43.80000 8.72000 -24.06000 L

C 0 45.91000 8.96000 -23.80000 L

H 0 46.23000 8.10000 -24.20000 L

H 0 46.53000 9.24000 -23.06000 L

N 0 45.94000 9.98000 -24.84000 L

H 0 45.12000 10.53000 -25.00000 L

C 0 47.04000 10.22000 -25.57000 L

N 0 48.09000 9.49000 -25.29000 L

H 0 48.05000 8.81000 -24.56000 L

H 0 48.94000 9.62000 -25.80000 L

N 0 47.11000 11.23000 -26.45000 L

H 0 46.32000 11.84000 -26.58000 L

H 0 47.94000 11.37000 -26.98000 L

C 0 42.83000 5.86000 -21.32000 L

O 0 42.39000 5.04000 -22.15000 L

N 0 43.14000 5.53000 -20.07000 L

H 0 43.40000 6.23000 -19.42000 L

C 0 43.10000 4.10000 -19.65000 L

H 0 43.60000 3.60000 -20.36000 L

C 0 43.82000 3.91000 -18.24000 L

H 0 43.79000 2.95000 -17.97000 L

H 0 44.78000 4.20000 -18.30000 L

H 0 43.35000 4.47000 -17.55000 L

C 0 41.64000 3.58000 -19.64000 L

O 0 41.41000 2.36000 -19.67000 L

N 0 40.67000 4.50000 -19.66000 L

H 0 40.90000 5.47000 -19.68000 L

C 0 39.27000 4.07000 -19.64000 L

H 0 39.25000 3.10000 -19.87000 L

C 0 38.71000 4.36000 -18.21000 L

H 0 39.25000 3.85000 -17.55000 L

H 0 37.76000 4.05000 -18.18000 L

C 0 38.76000 5.88000 -17.83000 L

H 0 38.26000 6.40000 -18.53000 L

H 0 39.72000 6.17000 -17.81000 L

C 0 38.11000 6.16000 -16.41000 L

H 0 38.21000 7.13000 -16.17000 L

H 0 38.54000 5.60000 -15.71000 L

N 0 36.66000 5.86000 -16.39000 L

H 0 36.36000 5.03000 -15.92000 L

C 0 35.75000 6.64000 -16.96000 L

N 0 36.12000 7.82000 -17.58000 L

H 0 37.08000 8.09000 -17.59000 L

H 0 35.42000 8.40000 -18.00000 L

N 0 34.46000 6.33000 -16.82000 L

H 0 34.19000 5.53000 -16.29000 L

H 0 33.76000 6.91000 -17.24000 L

C 0 38.45000 4.77000 -20.71000 L

O 0 37.23000 4.95000 -20.61000 L

N 0 39.15000 5.27000 -21.75000 L

H 0 40.12000 5.05000 -21.84000 L

C 0 38.51000 6.14000 -22.76000 L

H 0 38.12000 6.91000 -22.25000 L

C 0 39.57000 6.68000 -23.80000 L

H 0 39.98000 5.90000 -24.27000 L

H 0 40.28000 7.18000 -23.31000 L

C 0 39.01000 7.62000 -24.88000 L

H 0 38.49000 8.35000 -24.43000 L

H 0 38.39000 7.10000 -25.47000 L

C 0 40.12000 8.25000 -25.74000 L

O 0 41.25000 7.77000 -25.75000 L

N 0 39.82000 9.38000 -26.39000 L

H 0 38.90000 9.78000 -26.30000 L

H 0 40.50000 9.83000 -26.96000 L

C 0 37.35000 5.40000 -23.44000 L

O 0 36.28000 5.98000 -23.73000 L

N 0 37.57000 4.12000 -23.80000 L

H 0 38.42000 3.65000 -23.56000 L

C 0 36.51000 3.45000 -24.56000 L

H 0 36.15000 4.09000 -25.24000 L

C 0 37.07000 2.17000 -25.31000 L

H 0 36.31000 1.68000 -25.75000 L

H 0 37.52000 1.57000 -24.66000 L

C 0 38.10000 2.63000 -26.39000 L

H 0 38.39000 1.83000 -26.92000 L

H 0 38.89000 3.04000 -25.94000 L

C 0 37.56000 3.66000 -27.38000 L

O 0 36.44000 3.55000 -27.84000 L

N 0 38.31000 4.72000 -27.60000 L

H 0 39.19000 4.81000 -27.13000 L

H 0 38.01000 5.42000 -28.24000 L

C 0 35.41000 3.09000 -23.59000 L

O 0 34.23000 3.08000 -23.92000 L

N 0 35.75000 2.81000 -22.37000 L

H 0 36.71000 2.73000 -22.10000 L

C 0 34.68000 2.61000 -21.41000 L

H 0 34.11000 1.88000 -21.80000 L

C 0 35.29000 2.21000 -20.06000 L

H 0 35.96000 2.91000 -19.81000 L

H 0 35.75000 1.33000 -20.18000 L

C 0 34.25000 2.08000 -18.91000 L

H 0 33.70000 1.25000 -19.03000 L

H 0 33.65000 2.88000 -18.89000 L

C 0 35.05000 2.00000 -17.61000 L

H 0 34.64000 2.56000 -16.90000 L

H 0 36.00000 2.29000 -17.76000 L

C 0 35.06000 0.60000 -17.17000 L

H 0 35.69000 0.07000 -17.74000 L

H 0 34.14000 0.22000 -17.26000 L

N 0 35.49000 0.55000 -15.75000 L

H 0 35.50000 -0.40000 -15.44000 L

H 0 36.41000 0.93000 -15.67000 L

H 0 34.86000 1.08000 -15.19000 L

C 0 33.82000 3.86000 -21.24000 L

O 0 32.56000 3.77000 -21.07000 L

N 0 34.48000 5.05000 -21.23000 L

H 0 35.48000 5.06000 -21.32000 L

C 0 33.73000 6.33000 -21.11000 L

H 0 33.14000 6.25000 -20.30000 L

C 0 34.70000 7.52000 -20.94000 L

H 0 35.26000 7.61000 -21.77000 L

H 0 35.30000 7.35000 -20.15000 L

C 0 33.90000 8.84000 -20.72000 L

H 0 33.31000 9.00000 -21.51000 L

H 0 34.54000 9.60000 -20.61000 L

C 0 32.99000 8.81000 -19.46000 L

O 0 33.48000 8.46000 -18.39000 L

N 0 31.65000 9.05000 -19.63000 L

H 0 31.29000 9.23000 -20.55000 L

H 0 31.04000 9.03000 -18.84000 L

C 0 32.86000 6.53000 -22.34000 L

O 0 31.68000 6.93000 -22.26000 L

N 0 33.38000 6.16000 -23.52000 L

H 0 34.32000 5.81000 -23.60000 L

C 0 32.53000 6.30000 -24.72000 L

H 0 32.24000 7.25000 -24.61000 L

C 0 33.32000 5.94000 -26.14000 L

H 0 32.70000 6.05000 -26.91000 L

H 0 34.10000 6.56000 -26.25000 L

H 0 33.65000 5.00000 -26.11000 L

C 0 31.25000 5.54000 -24.62000 L

O 0 30.17000 6.06000 -25.06000 L

N 0 31.29000 4.33000 -24.05000 L

H 0 32.16000 3.95000 -23.74000 L

C 0 30.06000 3.56000 -23.90000 L

H 0 29.63000 3.63000 -24.80000 L

C 0 30.38000 2.11000 -23.48000 L

H 0 29.54000 1.66000 -23.17000 L

H 0 31.05000 2.12000 -22.73000 L

C 0 30.98000 1.35000 -24.76000 L

H 0 31.84000 1.77000 -25.03000 L

H 0 30.33000 1.40000 -25.52000 L

C 0 31.24000 -0.14000 -24.42000 L

H 0 30.36000 -0.58000 -24.20000 L

H 0 31.84000 -0.19000 -23.62000 L

C 0 31.90000 -0.90000 -25.60000 L

H 0 31.48000 -0.61000 -26.46000 L

H 0 31.77000 -1.89000 -25.48000 L

N 0 33.42000 -0.60000 -25.65000 L

H 0 33.84000 -1.09000 -26.42000 L

H 0 33.56000 0.38000 -25.77000 L

H 0 33.85000 -0.89000 -24.80000 L

C 0 29.17000 4.14000 -22.77000 L

O 0 27.95000 4.17000 -22.91000 L

N 0 29.77000 4.57000 -21.67000 L

H 0 30.76000 4.43000 -21.55000 L

C 0 28.97000 5.28000 -20.60000 L

H 0 28.28000 4.64000 -20.25000 L

C 0 29.86000 5.70000 -19.46000 L

H 0 29.35000 6.33000 -18.87000 L

H 0 30.66000 6.17000 -19.84000 L

C 0 30.35000 4.55000 -18.61000 L

H 0 30.69000 3.85000 -19.24000 L

C 0 31.54000 4.96000 -17.75000 L

H 0 31.85000 4.19000 -17.20000 L

H 0 32.29000 5.27000 -18.35000 L

H 0 31.27000 5.72000 -17.15000 L

C 0 29.15000 3.99000 -17.71000 L

H 0 29.47000 3.23000 -17.15000 L

H 0 28.81000 4.72000 -17.12000 L

H 0 28.40000 3.68000 -18.31000 L

C 0 28.28000 6.52000 -21.18000 L

O 0 27.04000 6.71000 -21.02000 L

N 0 29.03000 7.34000 -21.93000 L

H 0 30.00000 7.14000 -22.08000 L

C 0 28.43000 8.49000 -22.51000 L

H 0 28.13000 8.98000 -21.69000 L

C 0 29.48000 9.32000 -23.37000 L

H 0 29.97000 8.73000 -24.00000 L

C 0 28.76000 10.52000 -24.15000 L

H 0 29.44000 11.03000 -24.68000 L

H 0 28.07000 10.15000 -24.77000 L

H 0 28.33000 11.13000 -23.49000 L

O 0 30.50000 9.86000 -22.48000 L

H 0 31.16000 10.39000 -23.01000 L

C 0 27.23000 8.09000 -23.43000 L

O 0 26.10000 8.68000 -23.38000 L

N 0 27.46000 7.16000 -24.34000 L

H 0 28.36000 6.74000 -24.41000 L

C 0 26.36000 6.75000 -25.24000 L

H 0 26.05000 7.58000 -25.72000 L

C 0 26.88000 5.65000 -26.23000 L

H 0 27.37000 4.95000 -25.71000 L

H 0 27.51000 6.08000 -26.88000 L

C 0 25.74000 4.97000 -27.03000 L

H 0 25.09000 4.57000 -26.37000 L

H 0 26.13000 4.24000 -27.59000 L

C 0 24.95000 5.95000 -27.97000 L

O 0 23.72000 5.71000 -28.25000 L

O 0 25.55000 6.97000 -28.44000 L

C 0 25.19000 6.23000 -24.43000 L

O 0 24.07000 6.31000 -24.86000 L

N 0 25.44000 5.66000 -23.26000 L

H 0 26.38000 5.51000 -22.98000 L

C 0 24.38000 5.26000 -22.42000 L

H 0 23.74000 4.88000 -23.08000 L

C 0 24.97000 4.41000 -21.36000 L

H 0 24.66000 4.72000 -20.46000 L

H 0 25.97000 4.47000 -21.40000 L

C 0 24.61000 3.03000 -21.50000 L

O 0 23.50000 2.72000 -20.96000 L

O 0 25.40000 2.28000 -22.18000 L

C 0 23.75000 6.34000 -21.56000 L

O 0 23.03000 5.96000 -20.61000 L

N 0 24.12000 7.61000 -21.77000 L

H 0 24.83000 7.84000 -22.44000 L

C 0 23.46000 8.67000 -20.99000 L

H 0 22.57000 8.32000 -20.71000 L

H 0 23.34000 9.46000 -21.59000 L

C 0 24.22000 9.13000 -19.74000 L

O 0 23.59000 9.78000 -18.85000 L

N 0 25.53000 8.79000 -19.67000 L

H 0 25.94000 8.26000 -20.41000 L

C 0 26.38000 9.17000 -18.53000 L

H 0 25.84000 9.78000 -17.95000 L

C 0 26.77000 7.90000 -17.78000 L

H 0 27.41000 8.16000 -17.06000 L

H 0 27.22000 7.29000 -18.43000 L

C 0 25.59000 7.12000 -17.13000 L

H 0 25.00000 6.90000 -17.91000 L

C 0 26.00000 5.72000 -16.50000 L

H 0 25.20000 5.28000 -16.10000 L

H 0 26.39000 5.13000 -17.21000 L

H 0 26.69000 5.87000 -15.78000 L

C 0 24.85000 8.06000 -15.98000 L

H 0 24.10000 7.55000 -15.57000 L

H 0 25.52000 8.30000 -15.27000 L

H 0 24.50000 8.89000 -16.40000 L

C 0 27.63000 9.95000 -18.96000 L

O 0 28.75000 9.40000 -19.06000 L

N 0 27.47000 11.23000 -19.26000 L

C 0 26.18000 11.93000 -19.43000 L

H 0 25.95000 12.46000 -18.61000 L

H 0 25.44000 11.29000 -19.62000 L

C 0 26.42000 12.83000 -20.59000 L

H 0 25.88000 13.66000 -20.50000 L

H 0 26.19000 12.37000 -21.44000 L

C 0 27.87000 13.16000 -20.55000 L

H 0 28.02000 14.00000 -20.03000 L

H 0 28.21000 13.28000 -21.48000 L

C 0 28.56000 11.95000 -19.86000 L

H 0 29.04000 11.32000 -20.47000 L

C 0 29.64000 12.43000 -18.86000 L

O 0 29.44000 12.38000 -17.63000 L

N 0 30.74000 12.89000 -19.41000 L

H 0 30.79000 12.97000 -20.41000 L

C 0 31.92000 13.29000 -18.60000 L

H 0 31.66000 13.22000 -17.63000 L

C 0 33.09000 12.36000 -18.93000 L

H 0 33.22000 12.38000 -19.92000 L

H 0 32.83000 11.44000 -18.65000 L

C 0 34.43000 12.68000 -18.27000 L

H 0 34.66000 13.62000 -18.51000 L

C 0 34.23000 12.59000 -16.73000 L

H 0 35.09000 12.80000 -16.27000 L

H 0 33.53000 13.25000 -16.44000 L

H 0 33.93000 11.67000 -16.48000 L

C 0 35.62000 11.71000 -18.79000 L

H 0 36.47000 11.96000 -18.34000 L

H 0 35.39000 10.76000 -18.58000 L

H 0 35.73000 11.82000 -19.78000 L

C 0 32.32000 14.72000 -18.95000 L

O 0 32.51000 15.06000 -20.14000 L

N 0 32.45000 15.57000 -17.94000 L

H 0 32.19000 15.28000 -17.02000 L

C 0 32.96000 16.94000 -18.14000 L

H 0 32.35000 17.59000 -17.70000 L

H 0 33.00000 17.14000 -19.12000 L

C 0 34.36000 16.98000 -17.53000 L

O 0 34.71000 16.32000 -16.51000 L

N 0 35.17000 17.84000 -18.14000 L

H 0 34.86000 18.28000 -18.98000 L

C 0 36.47000 18.13000 -17.64000 L

H 0 36.51000 17.64000 -16.77000 L

C 0 37.55000 17.67000 -18.66000 L

H 0 37.37000 18.09000 -19.55000 L

C 0 38.96000 18.12000 -18.09000 L

H 0 39.68000 17.84000 -18.72000 L

H 0 38.98000 19.11000 -17.98000 L

H 0 39.11000 17.69000 -17.20000 L

C 0 37.51000 16.06000 -18.74000 L

H 0 38.20000 15.74000 -19.39000 L

H 0 37.71000 15.67000 -17.84000 L

H 0 36.60000 15.76000 -19.04000 L

C 0 36.64000 19.61000 -17.39000 L

O 0 36.44000 20.43000 -18.32000 L

N 0 36.95000 19.98000 -16.14000 L

H 0 37.12000 19.27000 -15.45000 L

C 0 37.05000 21.37000 -15.78000 L

H 0 36.58000 21.90000 -16.48000 L

C 0 36.38000 21.49000 -14.35000 L

H 0 36.88000 20.93000 -13.70000 L

H 0 35.43000 21.18000 -14.41000 L

C 0 36.35000 22.90000 -13.81000 L

O 0 37.37000 23.64000 -13.84000 L

N 0 35.16000 23.29000 -13.26000 L

H 0 34.38000 22.65000 -13.24000 L

H 0 35.06000 24.21000 -12.88000 L

C 0 38.55000 21.84000 -15.77000 L

O 0 39.39000 21.16000 -15.17000 L

N 0 38.85000 22.92000 -16.50000 L

H 0 38.09000 23.44000 -16.89000 L

C 0 40.22000 23.39000 -16.76000 L

H 0 40.80000 22.61000 -16.55000 L

C 0 40.40000 23.77000 -18.21000 L

H 0 41.35000 24.07000 -18.32000 L

H 0 39.78000 24.54000 -18.39000 L

C 0 40.12000 22.68000 -19.26000 L

H 0 39.16000 22.43000 -19.14000 L

C 0 40.27000 23.22000 -20.74000 L

H 0 40.08000 22.48000 -21.39000 L

H 0 39.62000 23.96000 -20.89000 L

H 0 41.20000 23.55000 -20.89000 L

C 0 41.07000 21.42000 -19.01000 L

H 0 40.88000 20.72000 -19.70000 L

H 0 42.03000 21.70000 -19.08000 L

H 0 40.90000 21.04000 -18.10000 L

C 0 40.54000 24.64000 -15.89000 L

O 0 39.73000 25.52000 -15.80000 L

N 0 41.70000 24.67000 -15.25000 L

H 0 42.28000 23.86000 -15.29000 L

C 0 42.17000 25.81000 -14.50000 L

H 0 42.25000 25.55000 -13.54000 L

H 0 41.50000 26.55000 -14.59000 L

C 0 43.53000 26.24000 -15.07000 L

O 0 43.93000 25.83000 -16.18000 L

N 0 44.21000 27.10000 -14.35000 L

H 0 43.86000 27.40000 -13.46000 L

C 0 45.45000 27.62000 -14.84000 L

H 0 45.76000 27.02000 -15.58000 L

C 0 45.22000 29.00000 -15.40000 L

H 0 44.49000 28.97000 -16.08000 L

H 0 46.06000 29.32000 -15.84000 L

C 0 44.85000 29.97000 -14.41000 L

H 0 45.12000 29.63000 -13.51000 L

H 0 43.86000 30.10000 -14.44000 L

C 0 45.66000 31.28000 -14.84000 L

H 0 45.02000 31.99000 -15.14000 L

H 0 46.29000 31.07000 -15.59000 L

C 0 46.48000 31.83000 -13.63000 L

H 0 46.80000 31.05000 -13.09000 L

H 0 45.88000 32.41000 -13.08000 L

N 0 47.70000 32.65000 -13.94000 L

H 0 48.14000 32.94000 -13.08000 L

H 0 48.35000 32.11000 -14.47000 L

H 0 47.44000 33.46000 -14.46000 L

C 0 46.46000 27.61000 -13.73000 L

O 0 46.11000 27.64000 -12.56000 L

N 0 47.72000 27.51000 -14.09000 L

H 0 47.98000 27.53000 -15.06000 L

C 0 48.73000 27.37000 -13.08000 L

H 0 48.38000 26.64000 -12.49000 L

C 0 50.07000 26.98000 -13.73000 L

H 0 50.82000 27.28000 -13.14000 L

H 0 50.14000 27.43000 -14.62000 L

C 0 50.18000 25.48000 -13.94000 L

O 0 50.28000 25.04000 -15.06000 L

N 0 50.10000 24.70000 -12.86000 L

H 0 49.97000 25.11000 -11.95000 L

H 0 50.17000 23.71000 -12.95000 L

C 0 48.94000 28.72000 -12.34000 L

O 0 48.83000 29.80000 -12.91000 L

N 0 49.36000 28.59000 -11.09000 L

H 0 49.52000 27.68000 -10.71000 L

C 0 49.59000 29.78000 -10.27000 L

H 0 48.73000 30.28000 -10.18000 L

C 0 50.07000 29.31000 -8.91000 L

H 0 50.91000 28.78000 -9.04000 L

H 0 49.36000 28.73000 -8.51000 L

C 0 50.36000 30.49000 -7.96000 L

H 0 49.51000 30.99000 -7.77000 L

H 0 51.02000 31.10000 -8.37000 L

C 0 50.91000 29.97000 -6.65000 L

H 0 51.81000 29.56000 -6.82000 L

H 0 50.29000 29.28000 -6.28000 L

C 0 51.04000 31.13000 -5.67000 L

H 0 50.19000 31.65000 -5.67000 L

H 0 51.79000 31.72000 -5.97000 L

N 0 51.33000 30.64000 -4.26000 L

H 0 51.40000 31.43000 -3.65000 L

H 0 50.58000 30.05000 -3.96000 L

H 0 52.18000 30.12000 -4.26000 L

C 0 50.63000 30.71000 -10.92000 L

O 0 50.44000 31.90000 -11.03000 L

N 0 51.75000 30.19000 -11.37000 L

H 0 51.91000 29.21000 -11.35000 L

C 0 52.77000 31.13000 -11.90000 L

H 0 52.52000 32.01000 -11.49000 L

C 0 54.14000 30.70000 -11.48000 L

H 0 54.83000 31.32000 -11.87000 L

C 0 54.20000 30.63000 -9.93000 L

H 0 55.12000 30.35000 -9.64000 L

H 0 54.00000 31.53000 -9.55000 L

H 0 53.53000 29.97000 -9.60000 L

O 0 54.37000 29.38000 -12.00000 L

H 0 55.29000 29.08000 -11.73000 L

C 0 52.76000 31.26000 -13.45000 L

O 0 53.73000 31.78000 -14.05000 L

N 0 51.66000 30.87000 -14.10000 L

H 0 50.88000 30.48000 -13.61000 L

C 0 51.62000 31.03000 -15.58000 L

H 0 52.49000 30.67000 -15.91000 L

C 0 50.51000 30.22000 -16.22000 L

H 0 50.43000 29.31000 -15.80000 L

H 0 50.65000 30.13000 -17.20000 L

O 0 49.29000 30.87000 -16.03000 L

H 0 48.56000 30.34000 -16.45000 L

C 0 51.57000 32.49000 -15.98000 L

O 0 51.00000 33.32000 -15.29000 L

N 0 52.19000 32.80000 -17.11000 L

H 0 52.64000 32.09000 -17.64000 L

C 0 52.21000 34.19000 -17.56000 L

H 0 51.89000 34.67000 -16.75000 L

C 0 53.59000 34.59000 -18.03000 L

H 0 53.96000 33.90000 -18.65000 L

C 0 53.47000 35.89000 -18.75000 L

H 0 54.37000 36.18000 -19.07000 L

H 0 52.86000 35.78000 -19.53000 L

H 0 53.10000 36.58000 -18.13000 L

C 0 54.51000 34.77000 -16.81000 L

H 0 55.43000 35.04000 -17.11000 L

H 0 54.14000 35.49000 -16.21000 L

H 0 54.57000 33.91000 -16.30000 L

C 0 51.30000 34.45000 -18.75000 L

O 0 51.42000 35.50000 -19.41000 L

N 0 50.44000 33.50000 -19.07000 L

H 0 50.40000 32.67000 -18.51000 L

C 0 49.58000 33.65000 -20.20000 L

H 0 49.26000 34.59000 -20.32000 L

C 0 50.40000 33.24000 -21.41000 L

H 0 50.67000 32.29000 -21.30000 L

H 0 51.22000 33.82000 -21.46000 L

C 0 49.66000 33.38000 -22.68000 L

O 0 48.42000 33.60000 -22.70000 L

O 0 50.36000 33.19000 -23.70000 L

C 0 48.41000 32.73000 -19.92000 L

O 0 48.43000 31.54000 -20.24000 L

N 0 47.40000 33.28000 -19.26000 L

H 0 47.43000 34.26000 -19.06000 L

C 0 46.27000 32.49000 -18.83000 L

H 0 46.62000 31.82000 -18.17000 L

C 0 45.21000 33.42000 -18.22000 L

H 0 44.42000 32.88000 -17.92000 L

H 0 45.60000 33.90000 -17.44000 L

H 0 44.92000 34.08000 -18.91000 L

C 0 45.64000 31.78000 -20.00000 L

O 0 45.18000 30.65000 -19.92000 L

N 0 45.42000 32.53000 -21.06000 L

H 0 45.72000 33.49000 -21.09000 L

C 0 44.73000 31.94000 -22.19000 L

H 0 43.88000 31.62000 -21.77000 L

C 0 44.50000 32.99000 -23.35000 L

H 0 44.02000 32.54000 -24.11000 L

H 0 43.94000 33.74000 -23.01000 L

H 0 45.38000 33.34000 -23.67000 L

C 0 45.47000 30.72000 -22.75000 L

O 0 44.81000 29.82000 -23.25000 L

N 0 46.79000 30.73000 -22.80000 L

H 0 47.30000 31.54000 -22.49000 L

C 0 47.52000 29.57000 -23.32000 L

H 0 47.10000 29.36000 -24.20000 L

C 0 49.03000 29.91000 -23.53000 L

H 0 49.43000 30.12000 -22.64000 L

H 0 49.09000 30.71000 -24.12000 L

C 0 49.82000 28.75000 -24.17000 L

H 0 49.80000 27.97000 -23.55000 L

H 0 50.76000 29.05000 -24.30000 L

C 0 49.24000 28.31000 -25.56000 L

O 0 49.51000 27.18000 -25.96000 L

O 0 48.49000 29.06000 -26.24000 L

C 0 47.38000 28.38000 -22.31000 L

O 0 47.29000 27.24000 -22.75000 L

N 0 47.34000 28.63000 -20.98000 L

H 0 47.54000 29.54000 -20.62000 L

C 0 47.00000 27.50000 -20.07000 L

H 0 47.78000 26.89000 -20.17000 L

C 0 46.85000 27.93000 -18.60000 L

H 0 46.36000 27.21000 -18.10000 L

H 0 46.34000 28.78000 -18.55000 L

C 0 48.18000 28.14000 -17.93000 L

O 0 49.22000 28.24000 -18.61000 L

O 0 48.26000 28.13000 -16.72000 L

C 0 45.65000 26.86000 -20.47000 L

O 0 45.55000 25.63000 -20.55000 L

N 0 44.59000 27.64000 -20.69000 L

H 0 44.65000 28.64000 -20.62000 L

C 0 43.31000 26.99000 -21.03000 L

H 0 43.14000 26.32000 -20.31000 L

C 0 42.14000 28.00000 -21.08000 L

H 0 41.33000 27.55000 -21.43000 L

H 0 42.39000 28.77000 -21.68000 L

C 0 41.88000 28.52000 -19.64000 L

C 0 42.31000 29.78000 -19.22000 L

H 0 42.78000 30.38000 -19.86000 L

C 0 42.09000 30.22000 -17.83000 L

H 0 42.41000 31.12000 -17.52000 L

C 0 41.42000 29.39000 -16.95000 L

O 0 41.15000 29.81000 -15.65000 L

H 0 40.66000 29.09000 -15.15000 L

C 0 40.95000 28.16000 -17.37000 L

H 0 40.45000 27.57000 -16.74000 L

C 0 41.20000 27.73000 -18.74000 L

H 0 40.86000 26.83000 -19.03000 L

C 0 43.45000 26.31000 -22.39000 L

O 0 42.78000 25.30000 -22.65000 L

N 0 44.13000 26.99000 -23.33000 L

H 0 44.56000 27.87000 -23.13000 L

C 0 44.22000 26.39000 -24.66000 L

H 0 43.27000 26.29000 -24.97000 L

C 0 45.06000 27.33000 -25.61000 L

H 0 45.12000 26.92000 -26.52000 L

H 0 44.62000 28.22000 -25.67000 L

H 0 45.98000 27.44000 -25.23000 L

C 0 44.87000 25.02000 -24.59000 L

O 0 44.51000 24.08000 -25.35000 L

N 0 45.93000 24.92000 -23.81000 L

H 0 46.28000 25.74000 -23.36000 L

C 0 46.61000 23.64000 -23.57000 L

H 0 46.90000 23.38000 -24.49000 L

C 0 47.80000 23.81000 -22.59000 L

H 0 48.03000 22.91000 -22.20000 L

H 0 47.53000 24.43000 -21.85000 L

C 0 49.06000 24.39000 -23.27000 L

H 0 48.79000 25.19000 -23.81000 L

H 0 49.44000 23.69000 -23.88000 L

C 0 50.17000 24.82000 -22.29000 L

O 0 51.20000 25.41000 -22.71000 L

O 0 50.03000 24.58000 -21.09000 L

C 0 45.66000 22.56000 -22.95000 L

O 0 45.65000 21.39000 -23.40000 L

N 0 44.84000 22.98000 -21.99000 L

H 0 44.93000 23.91000 -21.64000 L

C 0 43.81000 22.11000 -21.45000 L

H 0 43.28000 22.63000 -20.78000 L

H 0 44.26000 21.34000 -20.99000 L

C 0 42.88000 21.58000 -22.55000 L

O 0 42.47000 20.39000 -22.53000 L

N 0 42.47000 22.49000 -23.43000 L

H 0 42.77000 23.44000 -23.34000 L

C 0 41.57000 22.10000 -24.53000 L

H 0 40.75000 21.72000 -24.09000 L

C 0 41.15000 23.32000 -25.40000 L

H 0 41.99000 23.73000 -25.75000 L

C 0 40.30000 22.87000 -26.59000 L

H 0 40.04000 23.66000 -27.14000 L

H 0 40.82000 22.23000 -27.15000 L

H 0 39.47000 22.41000 -26.25000 L

C 0 40.31000 24.35000 -24.57000 L

H 0 40.05000 25.12000 -25.16000 L

H 0 39.48000 23.91000 -24.23000 L

H 0 40.85000 24.69000 -23.80000 L

C 0 42.26000 21.04000 -25.39000 L

O 0 41.58000 20.05000 -25.76000 L

N 0 43.54000 21.30000 -25.76000 L

H 0 43.99000 22.12000 -25.42000 L

C 0 44.28000 20.39000 -26.68000 L

H 0 43.70000 20.29000 -27.49000 L

C 0 45.66000 21.01000 -27.09000 L

H 0 46.27000 20.32000 -27.48000 L

H 0 46.11000 21.46000 -26.32000 L

C 0 45.24000 22.05000 -28.16000 L

H 0 44.78000 22.82000 -27.73000 L

H 0 44.62000 21.62000 -28.82000 L

C 0 46.40000 22.57000 -28.91000 L

H 0 46.14000 23.35000 -29.47000 L

H 0 46.81000 21.87000 -29.48000 L

N 0 47.34000 22.99000 -27.96000 L

H 0 47.83000 22.32000 -27.40000 L

C 0 47.59000 24.28000 -27.79000 L

N 0 46.87000 25.08000 -28.54000 L

H 0 46.20000 24.70000 -29.17000 L

H 0 47.00000 26.07000 -28.47000 L

N 0 48.49000 24.73000 -26.89000 L

H 0 48.99000 24.08000 -26.32000 L

H 0 48.65000 25.71000 -26.79000 L

C 0 44.45000 18.99000 -26.06000 L

O 0 44.22000 17.98000 -26.72000 L

N 0 44.71000 18.96000 -24.76000 L

H 0 44.60000 19.78000 -24.19000 L

C 0 45.16000 17.66000 -24.16000 L

H 0 45.61000 17.10000 -24.86000 L

C 0 46.15000 17.97000 -23.02000 L

H 0 45.67000 18.55000 -22.37000 L

C 0 46.46000 16.69000 -22.20000 L

H 0 47.11000 16.91000 -21.47000 L

H 0 45.62000 16.33000 -21.80000 L

H 0 46.87000 16.00000 -22.80000 L

C 0 47.50000 18.73000 -23.59000 L

H 0 48.13000 18.92000 -22.84000 L

H 0 47.96000 18.15000 -24.26000 L

H 0 47.24000 19.59000 -24.03000 L

C 0 43.95000 16.90000 -23.63000 L

O 0 43.83000 15.70000 -23.84000 L

N 0 43.00000 17.59000 -22.92000 L

H 0 43.10000 18.58000 -22.77000 L

C 0 41.85000 16.87000 -22.38000 L

H 0 42.12000 15.92000 -22.51000 L

C 0 41.61000 17.27000 -20.88000 L

H 0 40.80000 16.81000 -20.53000 L

H 0 41.48000 18.26000 -20.82000 L

C 0 42.80000 16.89000 -19.97000 L

H 0 43.59000 17.41000 -20.30000 L

C 0 42.53000 17.29000 -18.44000 L

H 0 43.31000 17.03000 -17.88000 L

H 0 42.38000 18.27000 -18.37000 L

H 0 41.71000 16.81000 -18.11000 L

C 0 43.11000 15.36000 -20.12000 L

H 0 43.88000 15.12000 -19.53000 L

H 0 42.31000 14.83000 -19.85000 L

H 0 43.34000 15.16000 -21.07000 L

C 0 40.56000 17.02000 -23.17000 L

O 0 39.63000 16.20000 -23.03000 L

N 0 40.50000 18.02000 -24.06000 L

H 0 41.25000 18.67000 -24.16000 L

C 0 39.30000 18.12000 -24.87000 L

H 0 39.45000 18.87000 -25.52000 L

H 0 38.55000 18.36000 -24.25000 L

C 0 38.93000 16.88000 -25.62000 L

O 0 37.73000 16.56000 -25.74000 L

N 0 39.95000 16.14000 -26.10000 L

C 0 41.34000 16.63000 -26.37000 L

H 0 41.92000 16.48000 -25.57000 L

H 0 41.32000 17.60000 -26.59000 L

C 0 41.79000 15.75000 -27.60000 L

H 0 42.75000 15.51000 -27.51000 L

H 0 41.64000 16.25000 -28.45000 L

C 0 40.90000 14.45000 -27.57000 L

H 0 41.35000 13.73000 -27.05000 L

H 0 40.71000 14.14000 -28.50000 L

C 0 39.57000 14.91000 -26.84000 L

H 0 38.79000 15.12000 -27.43000 L

C 0 39.01000 13.82000 -25.99000 L

O 0 38.47000 12.84000 -26.50000 L

N 0 39.11000 13.97000 -24.68000 L

H 0 39.54000 14.79000 -24.30000 L

C 0 38.59000 12.94000 -23.80000 L

H 0 38.39000 12.20000 -24.44000 L

C 0 39.67000 12.62000 -22.69000 L

H 0 39.28000 12.02000 -22.00000 L

H 0 39.98000 13.47000 -22.26000 L

C 0 40.91000 11.91000 -23.37000 L

H 0 40.88000 12.12000 -24.34000 L

C 0 42.26000 12.37000 -22.74000 L

H 0 43.02000 11.89000 -23.20000 L

H 0 42.37000 13.35000 -22.87000 L

H 0 42.27000 12.15000 -21.77000 L

C 0 40.82000 10.42000 -23.27000 L

H 0 41.62000 10.02000 -23.71000 L

H 0 40.80000 10.16000 -22.30000 L

H 0 39.99000 10.10000 -23.72000 L

C 0 37.31000 13.32000 -23.08000 L

O 0 36.84000 12.55000 -22.21000 L

N 0 36.69000 14.45000 -23.44000 L

H 0 36.97000 14.93000 -24.27000 L

C 0 35.59000 15.00000 -22.60000 L

H 0 35.41000 14.34000 -21.87000 L

C 0 36.02000 16.45000 -22.08000 L

H 0 35.28000 16.83000 -21.52000 L

H 0 36.85000 16.37000 -21.54000 L

H 0 36.18000 17.05000 -22.87000 L

C 0 34.36000 15.18000 -23.47000 L

O 0 34.47000 15.55000 -24.64000 L

N 0 33.17000 15.08000 -22.86000 L

H 0 33.12000 14.73000 -21.92000 L

C 0 31.97000 15.47000 -23.56000 L

H 0 32.02000 15.24000 -24.53000 L

C 0 30.82000 14.72000 -22.89000 L

H 0 29.96000 15.07000 -23.25000 L

H 0 30.87000 14.88000 -21.90000 L

C 0 30.94000 13.19000 -23.18000 L

O 0 31.18000 12.89000 -24.35000 L

O 0 30.88000 12.34000 -22.26000 L

C 0 31.84000 16.96000 -23.45000 L

O 0 31.31000 17.58000 -24.37000 L

N 0 32.25000 17.56000 -22.31000 L

H 0 32.54000 17.03000 -21.52000 L

C 0 32.27000 19.04000 -22.30000 L

H 0 32.39000 19.39000 -23.23000 L

C 0 30.92000 19.58000 -21.76000 L

H 0 30.19000 19.12000 -22.26000 L

H 0 30.89000 20.56000 -21.96000 L

C 0 30.66000 19.39000 -20.23000 L

C 0 29.90000 18.32000 -19.79000 L

H 0 29.55000 17.66000 -20.46000 L

C 0 29.59000 18.14000 -18.38000 L

H 0 28.98000 17.41000 -18.07000 L

C 0 30.18000 19.00000 -17.48000 L

O 0 29.91000 18.79000 -16.13000 L

H 0 30.40000 19.48000 -15.59000 L

C 0 31.03000 20.03000 -17.89000 L

H 0 31.48000 20.63000 -17.22000 L

C 0 31.25000 20.22000 -19.29000 L

H 0 31.83000 20.97000 -19.60000 L

C 0 33.45000 19.48000 -21.48000 L

O 0 33.93000 18.72000 -20.63000 L

N 0 33.95000 20.68000 -21.79000 L

H 0 33.56000 21.17000 -22.57000 L

C 0 35.03000 21.29000 -21.08000 L

H 0 35.42000 20.57000 -20.50000 L

C 0 36.06000 21.88000 -22.03000 L

H 0 36.70000 22.42000 -21.49000 L

H 0 35.58000 22.47000 -22.68000 L

C 0 36.84000 20.80000 -22.81000 L

H 0 36.24000 20.17000 -23.29000 L

C 0 37.75000 21.56000 -23.83000 L

H 0 38.28000 20.89000 -24.36000 L

H 0 37.18000 22.10000 -24.44000 L

H 0 38.38000 22.16000 -23.33000 L

C 0 37.79000 20.09000 -21.73000 L

H 0 38.33000 19.38000 -22.18000 L

H 0 38.40000 20.77000 -21.33000 L

H 0 37.23000 19.68000 -21.02000 L

C 0 34.45000 22.48000 -20.33000 L

O 0 33.47000 23.13000 -20.82000 L

N 0 35.09000 22.83000 -19.22000 L

H 0 35.88000 22.30000 -18.91000 L

C 0 34.62000 24.02000 -18.45000 L

H 0 33.82000 24.40000 -18.92000 L

C 0 34.28000 23.68000 -17.00000 L

H 0 35.13000 23.42000 -16.55000 L

C 0 33.66000 24.97000 -16.28000 L

H 0 33.44000 24.74000 -15.33000 L

H 0 34.33000 25.71000 -16.30000 L

H 0 32.83000 25.25000 -16.75000 L

C 0 33.23000 22.48000 -16.99000 L

H 0 33.00000 22.25000 -16.04000 L

H 0 32.40000 22.75000 -17.48000 L

H 0 33.63000 21.68000 -17.44000 L

C 0 35.80000 24.91000 -18.36000 L

O 0 36.85000 24.51000 -17.88000 L

N 0 35.63000 26.14000 -18.79000 L

H 0 34.79000 26.37000 -19.29000 L

C 0 36.63000 27.19000 -18.56000 L

H 0 37.54000 26.76000 -18.53000 L

C 0 36.59000 28.24000 -19.69000 L

H 0 35.70000 28.68000 -19.67000 L

C 0 37.63000 29.38000 -19.42000 L

H 0 37.59000 30.05000 -20.16000 L

H 0 37.41000 29.83000 -18.55000 L

H 0 38.55000 28.99000 -19.37000 L

C 0 36.83000 27.52000 -21.11000 L

H 0 36.81000 28.21000 -21.84000 L

H 0 37.72000 27.07000 -21.11000 L

H 0 36.11000 26.84000 -21.27000 L

C 0 36.26000 27.84000 -17.23000 L

O 0 35.31000 28.67000 -17.12000 L

N 0 37.03000 27.43000 -16.24000 L

H 0 37.78000 26.81000 -16.44000 L

C 0 36.81000 27.86000 -14.90000 L

H 0 35.82000 27.94000 -14.80000 L

C 0 37.44000 26.84000 -13.93000 L

H 0 38.43000 26.81000 -14.09000 L

H 0 37.05000 25.94000 -14.11000 L

C 0 37.16000 27.26000 -12.45000 L

O 0 36.40000 28.15000 -12.27000 L

N 0 37.81000 26.68000 -11.49000 L

H 0 38.48000 25.96000 -11.70000 L

H 0 37.64000 26.94000 -10.54000 L

C 0 37.53000 29.16000 -14.67000 L

O 0 38.78000 29.16000 -14.51000 L

N 0 36.80000 30.27000 -14.77000 L

H 0 35.84000 30.21000 -15.02000 L

C 0 37.41000 31.56000 -14.50000 L

H 0 38.39000 31.37000 -14.39000 L

C 0 37.21000 32.50000 -15.74000 L

H 0 37.73000 32.15000 -16.52000 L

C 0 35.71000 32.57000 -16.11000 L

H 0 35.59000 33.17000 -16.90000 L

H 0 35.38000 31.65000 -16.34000 L

H 0 35.19000 32.92000 -15.33000 L

C 0 37.77000 33.90000 -15.43000 L

H 0 37.63000 34.49000 -16.23000 L

H 0 37.28000 34.29000 -14.65000 L

H 0 38.74000 33.84000 -15.23000 L

C 0 36.83000 32.16000 -13.16000 L

O 0 36.77000 33.41000 -12.96000 L

N 0 36.43000 31.28000 -12.24000 L

H 0 36.63000 30.31000 -12.35000 L

C 0 35.70000 31.75000 -11.07000 L

H 0 35.92000 32.72000 -11.07000 L

C 0 34.15000 31.48000 -11.22000 L

H 0 33.76000 32.05000 -11.96000 L

H 0 33.67000 31.67000 -10.37000 L

O 0 33.88000 30.13000 -11.56000 L

H 0 32.89000 30.01000 -11.65000 L

C 0 36.21000 31.20000 -9.74000 L

O 0 35.56000 31.46000 -8.69000 L

N 0 37.36000 30.48000 -9.73000 L

H 0 37.81000 30.20000 -10.58000 L

C 0 37.90000 30.12000 -8.40000 L

H 0 37.22000 29.54000 -7.96000 L

C 0 39.24000 29.40000 -8.57000 L

H 0 39.91000 29.99000 -9.03000 L

H 0 39.14000 28.56000 -9.09000 L

O 0 39.76000 29.07000 -7.26000 L

H 0 40.64000 28.60000 -7.36000 L

C 0 38.20000 31.42000 -7.63000 L

O 0 38.87000 32.28000 -8.17000 L

N 0 37.80000 31.51000 -6.35000 L

C 0 36.94000 30.59000 -5.57000 L

H 0 37.42000 29.73000 -5.41000 L

H 0 36.09000 30.41000 -6.08000 L

C 0 36.67000 31.36000 -4.24000 L

H 0 37.09000 30.90000 -3.47000 L

H 0 35.69000 31.45000 -4.08000 L

C 0 37.28000 32.74000 -4.39000 L

H 0 37.76000 32.99000 -3.55000 L

H 0 36.56000 33.41000 -4.58000 L

C 0 38.26000 32.68000 -5.58000 L

H 0 38.27000 33.48000 -6.17000 L

C 0 39.68000 32.50000 -5.03000 L

O 0 40.15000 33.37000 -4.37000 L

N 0 40.32000 31.38000 -5.30000 L

H 0 39.95000 30.76000 -6.00000 L

C 0 41.57000 30.99000 -4.62000 L

H 0 41.67000 31.70000 -3.92000 L

C 0 41.46000 29.59000 -4.04000 L

H 0 42.29000 29.37000 -3.53000 L

H 0 41.33000 28.93000 -4.78000 L

C 0 40.29000 29.51000 -3.10000 L

O 0 40.08000 30.47000 -2.31000 L

N 0 39.47000 28.46000 -3.21000 L

H 0 39.64000 27.76000 -3.90000 L

H 0 38.68000 28.38000 -2.60000 L

C 0 42.78000 31.04000 -5.50000 L

O 0 43.84000 30.51000 -5.10000 L

N 0 42.64000 31.71000 -6.66000 L

H 0 41.74000 32.05000 -6.93000 L

C 0 43.81000 31.96000 -7.52000 L

H 0 44.62000 31.71000 -6.98000 L

C 0 43.76000 31.07000 -8.79000 L

H 0 42.86000 31.09000 -9.23000 L

C 0 44.84000 31.51000 -9.79000 L

H 0 44.80000 30.94000 -10.60000 L

H 0 44.69000 32.47000 -10.04000 L

H 0 45.74000 31.42000 -9.36000 L

O 0 43.99000 29.70000 -8.40000 L

H 0 43.96000 29.11000 -9.21000 L

C 0 43.85000 33.44000 -7.87000 L

O 0 42.86000 33.97000 -8.46000 L

N 0 44.96000 34.11000 -7.52000 L

H 0 45.74000 33.58000 -7.17000 L

C 0 45.10000 35.60000 -7.64000 L

H 0 44.40000 35.99000 -7.05000 L

C 0 46.55000 36.06000 -7.21000 L

H 0 46.63000 37.06000 -7.30000 L

H 0 46.72000 35.80000 -6.26000 L

H 0 47.23000 35.63000 -7.81000 L

C 0 44.84000 36.07000 -9.08000 L

O 0 45.46000 35.56000 -10.00000 L

N 0 43.95000 37.04000 -9.28000 L

H 0 43.46000 37.41000 -8.49000 L

C 0 43.67000 37.57000 -10.62000 L

H 0 44.55000 37.66000 -11.09000 L

H 0 43.27000 38.48000 -10.50000 L

C 0 42.74000 36.75000 -11.51000 L

O 0 42.32000 37.22000 -12.60000 L

N 0 42.45000 35.52000 -11.11000 L

H 0 42.75000 35.19000 -10.21000 L

C 0 41.69000 34.63000 -12.01000 L

H 0 42.25000 34.59000 -12.84000 L

C 0 41.52000 33.23000 -11.36000 L

H 0 40.93000 33.32000 -10.56000 L

H 0 42.42000 32.90000 -11.07000 L

C 0 40.89000 32.22000 -12.35000 L

H 0 40.16000 32.69000 -12.85000 L

C 0 41.96000 31.77000 -13.30000 L

H 0 41.58000 31.12000 -13.95000 L

H 0 42.31000 32.57000 -13.80000 L

H 0 42.70000 31.34000 -12.79000 L

C 0 40.23000 30.97000 -11.68000 L

H 0 39.85000 30.37000 -12.38000 L

H 0 40.91000 30.47000 -11.15000 L

H 0 39.49000 31.27000 -11.07000 L

C 0 40.31000 35.20000 -12.36000 L

O 0 39.84000 35.13000 -13.50000 L

N 0 39.63000 35.72000 -11.35000 L

H 0 40.06000 35.84000 -10.46000 L

C 0 38.24000 36.11000 -11.54000 L

H 0 37.82000 35.34000 -12.01000 L

C 0 37.55000 36.38000 -10.17000 L

H 0 36.72000 36.92000 -10.32000 L

H 0 38.18000 36.89000 -9.58000 L

C 0 37.16000 35.05000 -9.47000 L

H 0 37.97000 34.47000 -9.38000 L

H 0 36.47000 34.57000 -10.02000 L

C 0 36.59000 35.35000 -8.06000 L

H 0 36.28000 34.51000 -7.62000 L

H 0 35.82000 35.99000 -8.12000 L

N 0 37.67000 35.96000 -7.26000 L

H 0 38.62000 35.77000 -7.51000 L

C 0 37.45000 36.75000 -6.22000 L

N 0 36.19000 37.04000 -5.90000 L

H 0 35.44000 36.65000 -6.45000 L

H 0 35.99000 37.63000 -5.13000 L

N 0 38.46000 37.29000 -5.53000 L

H 0 39.41000 37.09000 -5.80000 L

H 0 38.28000 37.88000 -4.75000 L

C 0 38.13000 37.35000 -12.42000 L

O 0 37.07000 37.60000 -13.00000 L

N 0 39.23000 38.11000 -12.57000 L

H 0 40.05000 37.91000 -12.04000 L

C 0 39.22000 39.24000 -13.52000 L

H 0 38.37000 39.73000 -13.32000 L

C 0 40.35000 40.21000 -13.24000 L

H 0 40.50000 40.34000 -12.26000 L

H 0 40.18000 41.10000 -13.67000 L

O 0 41.53000 39.63000 -13.82000 L

H 0 42.30000 40.24000 -13.66000 L

C 0 39.16000 38.75000 -14.99000 L

O 0 38.80000 39.51000 -15.87000 L

N 0 39.39000 37.46000 -15.25000 L

H 0 39.73000 36.86000 -14.52000 L

C 0 39.14000 36.89000 -16.62000 L

H 0 39.63000 37.52000 -17.24000 L

C 0 39.69000 35.45000 -16.73000 L

H 0 39.44000 35.07000 -17.62000 L

H 0 39.29000 34.89000 -16.00000 L

C 0 41.21000 35.41000 -16.59000 L

H 0 41.42000 35.85000 -15.72000 L

C 0 41.73000 33.97000 -16.40000 L

H 0 42.73000 33.99000 -16.31000 L

H 0 41.33000 33.58000 -15.57000 L

H 0 41.47000 33.42000 -17.19000 L

C 0 41.94000 36.24000 -17.77000 L

H 0 42.93000 36.19000 -17.65000 L

H 0 41.69000 35.84000 -18.65000 L

H 0 41.64000 37.19000 -17.74000 L

C 0 37.66000 36.87000 -17.00000 L

O 0 37.31000 36.57000 -18.13000 L

N 0 36.78000 37.08000 -16.02000 L

H 0 37.12000 37.16000 -15.08000 L

C 0 35.33000 37.18000 -16.27000 L

H 0 35.12000 36.44000 -16.90000 L

C 0 34.56000 37.03000 -14.92000 L

H 0 33.61000 37.30000 -15.05000 L

H 0 34.98000 37.61000 -14.22000 L

C 0 34.61000 35.55000 -14.45000 L

H 0 35.56000 35.23000 -14.45000 L

H 0 34.08000 34.98000 -15.08000 L

C 0 34.03000 35.39000 -13.04000 L

O 0 33.00000 34.72000 -12.87000 L

N 0 34.62000 36.10000 -12.05000 L

H 0 35.40000 36.69000 -12.26000 L

H 0 34.28000 36.03000 -11.11000 L

C 0 34.98000 38.59000 -16.87000 L

O 0 33.89000 38.78000 -17.42000 L

N 0 35.89000 39.54000 -16.79000 L

H 0 36.74000 39.37000 -16.29000 L

C 0 35.64000 40.86000 -17.42000 L

H 0 36.45000 41.44000 -17.27000 L

H 0 34.85000 41.28000 -16.98000 L

C 0 35.38000 40.72000 -18.93000 L

O 0 35.96000 39.83000 -19.62000 L

N 0 34.58000 41.65000 -19.49000 L

H 0 34.25000 42.42000 -18.95000 L

C 0 34.19000 41.52000 -20.88000 L

H 0 33.62000 40.70000 -20.80000 L

C 0 33.40000 42.73000 -21.35000 L

H 0 34.02000 43.50000 -21.51000 L

H 0 32.73000 42.98000 -20.66000 L

C 0 32.69000 42.40000 -22.64000 L

H 0 32.25000 41.51000 -22.54000 L

H 0 33.37000 42.36000 -23.37000 L

C 0 31.61000 43.47000 -23.01000 L

H 0 32.03000 44.36000 -23.18000 L

H 0 30.93000 43.55000 -22.28000 L

C 0 30.95000 42.96000 -24.27000 L

H 0 30.74000 41.99000 -24.16000 L

H 0 31.58000 43.08000 -25.04000 L

N 0 29.69000 43.70000 -24.56000 L

H 0 29.28000 43.34000 -25.40000 L

H 0 29.05000 43.58000 -23.80000 L

H 0 29.89000 44.67000 -24.68000 L

C 0 35.33000 41.25000 -21.92000 L

O 0 35.25000 40.36000 -22.76000 L

N 0 36.33000 42.14000 -21.94000 L

H 0 36.41000 42.83000 -21.22000 L

C 0 37.30000 42.10000 -23.04000 L

H 0 36.77000 42.00000 -23.88000 L

C 0 38.08000 43.40000 -23.09000 L

H 0 38.74000 43.36000 -23.84000 L

H 0 37.45000 44.16000 -23.25000 L

H 0 38.56000 43.54000 -22.23000 L

C 0 38.29000 40.90000 -22.81000 L

O 0 38.64000 40.27000 -23.73000 L

N 0 38.74000 40.66000 -21.58000 L

H 0 38.52000 41.32000 -20.86000 L

C 0 39.55000 39.47000 -21.21000 L

H 0 40.38000 39.56000 -21.77000 L

C 0 39.93000 39.41000 -19.69000 L

H 0 40.34000 38.52000 -19.52000 L

H 0 39.08000 39.49000 -19.16000 L

C 0 40.93000 40.51000 -19.18000 L

H 0 40.45000 41.39000 -19.19000 L

H 0 41.70000 40.55000 -19.81000 L

C 0 41.48000 40.26000 -17.74000 L

O 0 41.92000 41.25000 -17.08000 L

O 0 41.50000 39.09000 -17.25000 L

C 0 38.79000 38.22000 -21.55000 L

O 0 39.38000 37.29000 -22.13000 L

N 0 37.52000 38.16000 -21.16000 L

H 0 37.11000 38.95000 -20.68000 L

C 0 36.73000 37.01000 -21.41000 L

H 0 37.25000 36.30000 -20.94000 L

C 0 35.30000 37.10000 -20.82000 L

H 0 34.80000 37.79000 -21.34000 L

H 0 35.38000 37.40000 -19.86000 L

C 0 34.46000 35.81000 -20.83000 L

H 0 34.45000 35.55000 -21.80000 L

C 0 35.13000 34.68000 -19.98000 L

H 0 34.56000 33.86000 -20.02000 L

H 0 36.03000 34.48000 -20.35000 L

H 0 35.22000 34.99000 -19.04000 L

C 0 32.97000 36.01000 -20.36000 L

H 0 32.49000 35.14000 -20.40000 L

H 0 32.96000 36.36000 -19.43000 L

H 0 32.51000 36.67000 -20.97000 L

C 0 36.62000 36.78000 -22.92000 L

O 0 36.77000 35.67000 -23.33000 L

N 0 36.32000 37.82000 -23.72000 L

H 0 36.29000 38.75000 -23.36000 L

C 0 36.04000 37.54000 -25.11000 L

H 0 35.29000 36.89000 -25.12000 L

C 0 35.60000 38.82000 -25.83000 L

H 0 36.33000 39.49000 -25.75000 L

H 0 34.77000 39.16000 -25.40000 L

C 0 35.32000 38.60000 -27.33000 L

H 0 34.48000 38.06000 -27.41000 L

H 0 36.08000 38.09000 -27.72000 L

C 0 35.15000 39.97000 -28.08000 L

H 0 35.06000 39.82000 -29.07000 L

H 0 35.93000 40.57000 -27.90000 L

N 0 33.94000 40.69000 -27.65000 L

H 0 33.05000 40.28000 -27.87000 L

C 0 33.93000 41.84000 -26.98000 L

N 0 35.07000 42.41000 -26.63000 L

H 0 35.94000 41.99000 -26.86000 L

H 0 35.06000 43.28000 -26.12000 L

N 0 32.78000 42.38000 -26.62000 L

H 0 31.92000 41.93000 -26.86000 L

H 0 32.77000 43.25000 -26.12000 L

C 0 37.34000 36.99000 -25.78000 L

O 0 37.31000 36.07000 -26.57000 L

N 0 38.46000 37.61000 -25.45000 L

H 0 38.42000 38.37000 -24.80000 L

C 0 39.74000 37.20000 -26.01000 L

H 0 39.65000 37.24000 -27.01000 L

C 0 40.82000 38.16000 -25.54000 L

H 0 40.50000 38.57000 -24.69000 L

H 0 40.91000 38.87000 -26.24000 L

C 0 42.20000 37.50000 -25.30000 L

H 0 42.39000 36.83000 -26.02000 L

H 0 42.20000 37.03000 -24.41000 L

C 0 43.31000 38.59000 -25.30000 L

H 0 44.21000 38.19000 -25.46000 L

H 0 43.31000 39.10000 -24.44000 L

N 0 43.00000 39.51000 -26.40000 L

H 0 43.39000 39.30000 -27.30000 L

C 0 42.25000 40.61000 -26.31000 L

N 0 41.76000 41.01000 -25.14000 L

H 0 41.95000 40.49000 -24.31000 L

H 0 41.20000 41.84000 -25.09000 L

N 0 42.03000 41.34000 -27.41000 L

H 0 42.43000 41.05000 -28.28000 L

H 0 41.47000 42.16000 -27.36000 L

C 0 40.10000 35.77000 -25.58000 L

O 0 40.50000 34.94000 -26.42000 L

N 0 39.95000 35.48000 -24.30000 L

H 0 39.66000 36.20000 -23.66000 L

C 0 40.21000 34.14000 -23.78000 L

H 0 41.16000 33.94000 -24.01000 L

C 0 40.00000 34.09000 -22.25000 L

H 0 39.17000 34.60000 -22.03000 L

H 0 40.79000 34.52000 -21.81000 L

C 0 39.85000 32.65000 -21.71000 L

H 0 39.27000 32.08000 -22.30000 L

C 0 41.15000 32.09000 -21.73000 L

H 0 41.11000 31.15000 -21.39000 L

H 0 41.50000 32.09000 -22.67000 L

H 0 41.75000 32.63000 -21.15000 L

C 0 39.32000 32.66000 -20.24000 L

H 0 39.23000 31.72000 -19.91000 L

H 0 39.96000 33.16000 -19.66000 L

H 0 38.42000 33.11000 -20.21000 L

C 0 39.32000 33.12000 -24.45000 L

O 0 39.79000 32.02000 -24.88000 L

N 0 38.02000 33.41000 -24.54000 L

H 0 37.67000 34.30000 -24.28000 L

C 0 37.13000 32.37000 -25.04000 L

H 0 37.45000 31.56000 -24.54000 L

C 0 35.68000 32.65000 -24.73000 L

H 0 35.11000 31.95000 -25.17000 L

H 0 35.44000 33.55000 -25.09000 L

C 0 35.42000 32.62000 -23.21000 L

H 0 36.03000 33.29000 -22.78000 L

C 0 33.95000 33.01000 -22.95000 L

H 0 33.78000 33.00000 -21.96000 L

H 0 33.78000 33.93000 -23.30000 L

H 0 33.35000 32.36000 -23.40000 L

C 0 35.72000 31.21000 -22.61000 L

H 0 35.54000 31.22000 -21.63000 L

H 0 35.12000 30.53000 -23.05000 L

H 0 36.67000 30.97000 -22.78000 L

C 0 37.25000 32.23000 -26.56000 L

O 0 36.97000 31.16000 -27.11000 L

N 0 37.56000 33.32000 -27.25000 L

H 0 37.63000 34.22000 -26.81000 L

C 0 37.80000 33.14000 -28.69000 L

H 0 36.94000 32.79000 -29.06000 L

C 0 38.22000 34.52000 -29.35000 L

H 0 38.97000 34.97000 -28.88000 L

C 0 38.62000 34.35000 -30.87000 L

H 0 38.88000 35.24000 -31.25000 L

H 0 39.40000 33.72000 -30.93000 L

H 0 37.85000 33.98000 -31.38000 L

O 0 37.07000 35.37000 -29.24000 L

H 0 37.27000 36.26000 -29.64000 L

C 0 38.91000 32.11000 -28.92000 L

O 0 38.79000 31.25000 -29.81000 L

N 0 40.00000 32.26000 -28.19000 L

H 0 40.05000 33.01000 -27.54000 L

C 0 41.11000 31.35000 -28.31000 L

H 0 41.39000 31.39000 -29.27000 L

C 0 42.22000 31.83000 -27.40000 L

H 0 41.85000 31.94000 -26.48000 L

H 0 42.55000 32.72000 -27.73000 L

C 0 43.37000 30.83000 -27.38000 L

H 0 43.51000 30.48000 -28.30000 L

H 0 43.14000 30.08000 -26.76000 L

C 0 44.64000 31.56000 -26.88000 L

H 0 44.47000 32.03000 -26.02000 L

H 0 44.96000 32.22000 -27.56000 L

C 0 45.71000 30.59000 -26.66000 L

H 0 45.53000 29.77000 -27.21000 L

H 0 45.73000 30.34000 -25.69000 L

N 0 47.02000 31.12000 -27.03000 L

H 0 47.73000 30.43000 -26.87000 L

H 0 47.02000 31.37000 -28.00000 L

H 0 47.22000 31.94000 -26.49000 L

C 0 40.71000 29.89000 -27.94000 L

O 0 41.03000 28.86000 -28.66000 L

N 0 40.03000 29.77000 -26.79000 L

H 0 39.86000 30.56000 -26.20000 L

C 0 39.53000 28.42000 -26.42000 L

H 0 40.33000 27.83000 -26.33000 L

C 0 38.74000 28.55000 -25.09000 L

H 0 38.09000 29.30000 -25.17000 L

C 0 37.92000 27.28000 -24.75000 L

H 0 37.43000 27.42000 -23.89000 L

H 0 37.26000 27.10000 -25.48000 L

H 0 38.53000 26.49000 -24.66000 L

C 0 39.75000 28.82000 -23.98000 L

H 0 39.27000 28.91000 -23.11000 L

H 0 40.40000 28.06000 -23.93000 L

H 0 40.24000 29.67000 -24.18000 L

C 0 38.68000 27.79000 -27.51000 L

O 0 38.82000 26.58000 -27.88000 L

N 0 37.70000 28.53000 -28.01000 L

H 0 37.55000 29.48000 -27.72000 L

C 0 36.84000 27.91000 -29.01000 L

H 0 36.57000 27.08000 -28.52000 L

C 0 35.63000 28.81000 -29.38000 L

H 0 35.10000 28.37000 -30.11000 L

H 0 35.96000 29.70000 -29.70000 L

C 0 34.72000 29.01000 -28.13000 L

H 0 35.28000 29.28000 -27.35000 L

C 0 33.73000 30.12000 -28.39000 L

H 0 33.15000 30.24000 -27.59000 L

H 0 34.22000 30.97000 -28.59000 L

H 0 33.16000 29.87000 -29.18000 L

C 0 33.94000 27.68000 -27.83000 L

H 0 33.36000 27.81000 -27.02000 L

H 0 33.37000 27.44000 -28.61000 L

H 0 34.59000 26.95000 -27.65000 L

C 0 37.60000 27.58000 -30.33000 L

O 0 37.22000 26.64000 -30.99000 L

N 0 38.55000 28.42000 -30.74000 L

H 0 38.75000 29.25000 -30.22000 L

C 0 39.30000 28.10000 -31.97000 L

H 0 38.63000 28.07000 -32.71000 L

C 0 40.36000 29.17000 -32.27000 L

H 0 40.95000 29.26000 -31.47000 L

H 0 39.89000 30.04000 -32.42000 L

C 0 41.22000 28.75000 -33.58000 L

H 0 40.62000 28.68000 -34.37000 L

H 0 41.67000 27.87000 -33.41000 L

C 0 42.34000 29.80000 -33.92000 L

O 0 42.01000 30.98000 -34.07000 L

N 0 43.64000 29.37000 -34.09000 L

H 0 43.86000 28.40000 -33.99000 L

H 0 44.36000 30.03000 -34.31000 L

C 0 40.05000 26.76000 -31.75000 L

O 0 40.06000 25.85000 -32.62000 L

N 0 40.62000 26.61000 -30.56000 L

H 0 40.56000 27.35000 -29.89000 L

C 0 41.37000 25.37000 -30.22000 L

H 0 42.06000 25.24000 -30.92000 L

C 0 42.06000 25.50000 -28.85000 L

H 0 42.38000 24.61000 -28.55000 L

H 0 41.41000 25.86000 -28.18000 L

C 0 43.25000 26.44000 -28.92000 L

H 0 43.80000 26.37000 -28.10000 L

H 0 42.93000 27.39000 -29.03000 L

C 0 44.05000 26.01000 -30.14000 L

O 0 43.94000 26.69000 -31.25000 L

O 0 44.66000 24.92000 -30.01000 L

C 0 40.40000 24.20000 -30.21000 L

O 0 40.76000 23.14000 -30.71000 L

N 0 39.18000 24.35000 -29.62000 L

H 0 38.95000 25.23000 -29.23000 L

C 0 38.21000 23.26000 -29.56000 L

H 0 38.65000 22.47000 -29.13000 L

C 0 36.98000 23.75000 -28.75000 L

H 0 36.58000 24.54000 -29.21000 L

H 0 37.27000 24.01000 -27.83000 L

C 0 35.90000 22.74000 -28.59000 L

H 0 35.35000 23.01000 -27.80000 L

H 0 36.35000 21.86000 -28.40000 L

C 0 34.97000 22.54000 -29.76000 L

H 0 34.27000 21.87000 -29.51000 L

H 0 35.50000 22.21000 -30.54000 L

N 0 34.28000 23.76000 -30.18000 L

H 0 34.68000 24.27000 -30.95000 L

C 0 33.16000 24.26000 -29.64000 L

N 0 32.60000 25.34000 -30.19000 L

H 0 33.02000 25.76000 -30.99000 L

H 0 31.77000 25.73000 -29.80000 L

N 0 32.56000 23.67000 -28.57000 L

H 0 32.96000 22.84000 -28.17000 L

H 0 31.73000 24.06000 -28.18000 L

C 0 37.79000 22.92000 -30.98000 L

O 0 37.64000 21.74000 -31.33000 L

N 0 37.53000 23.96000 -31.79000 L

H 0 37.69000 24.90000 -31.49000 L

C 0 37.00000 23.68000 -33.16000 L

H 0 36.21000 23.09000 -32.99000 L

C 0 36.63000 25.00000 -33.91000 L

H 0 36.50000 24.81000 -34.88000 L

H 0 37.37000 25.66000 -33.79000 L

C 0 35.34000 25.63000 -33.36000 L

O 0 34.61000 24.93000 -32.63000 L

O 0 35.14000 26.80000 -33.60000 L

C 0 38.07000 22.98000 -34.05000 L

O 0 37.70000 22.36000 -35.07000 L

N 0 39.33000 23.12000 -33.68000 L

H 0 39.54000 23.72000 -32.91000 L

C 0 40.46000 22.41000 -34.37000 L

H 0 41.31000 22.85000 -34.10000 L

H 0 40.33000 22.51000 -35.36000 L

C 0 40.54000 20.90000 -34.02000 L

O 0 41.36000 20.14000 -34.59000 L

N 0 39.74000 20.42000 -33.08000 L

H 0 39.11000 21.02000 -32.58000 L

C 0 39.80000 18.98000 -32.79000 L

H 0 40.76000 18.73000 -32.94000 L

C 0 39.29000 18.68000 -31.31000 L

H 0 39.32000 17.69000 -31.14000 L

H 0 38.36000 19.01000 -31.20000 L

C 0 40.25000 19.43000 -30.30000 L

H 0 40.35000 20.38000 -30.60000 L

C 0 39.62000 19.21000 -28.87000 L

H 0 40.18000 19.66000 -28.18000 L

H 0 38.70000 19.59000 -28.85000 L

H 0 39.58000 18.23000 -28.67000 L

C 0 41.70000 18.98000 -30.28000 L

H 0 42.21000 19.52000 -29.61000 L

H 0 41.74000 18.01000 -30.03000 L

H 0 42.10000 19.10000 -31.18000 L

C 0 38.94000 18.20000 -33.77000 L

O 0 37.85000 18.70000 -34.18000 L

N 0 39.27000 16.93000 -33.98000 L

H 0 40.09000 16.55000 -33.54000 L

C 0 38.44000 16.07000 -34.86000 L

H 0 38.34000 16.55000 -35.73000 L

C 0 39.12000 14.66000 -35.01000 L

H 0 38.50000 14.03000 -35.46000 L

H 0 39.36000 14.30000 -34.10000 L

C 0 40.43000 14.83000 -35.87000 L

H 0 40.94000 15.63000 -35.55000 L

H 0 40.18000 14.95000 -36.83000 L

C 0 41.33000 13.52000 -35.71000 L

H 0 41.53000 13.38000 -34.74000 L

H 0 42.18000 13.64000 -36.22000 L

N 0 40.74000 12.26000 -36.19000 L

H 0 40.17000 11.73000 -35.57000 L

C 0 40.95000 11.79000 -37.42000 L

N 0 40.54000 10.56000 -37.72000 L

H 0 40.08000 10.01000 -37.03000 L

H 0 40.69000 10.20000 -38.64000 L

N 0 41.55000 12.55000 -38.33000 L

H 0 41.84000 13.48000 -38.10000 L

H 0 41.70000 12.20000 -39.26000 L

C 0 37.06000 15.91000 -34.31000 L

O 0 36.88000 15.92000 -33.03000 L

N 0 36.08000 15.74000 -35.22000 L

H 0 36.31000 15.56000 -36.18000 L

C 0 34.68000 15.82000 -34.80000 L

H 0 34.66000 16.74000 -34.43000 L

C 0 33.70000 15.58000 -36.00000 L

H 0 33.37000 16.46000 -36.34000 L

H 0 32.92000 15.03000 -35.70000 L

C 0 34.38000 14.87000 -37.14000 L

H 0 34.25000 13.89000 -36.97000 L

H 0 35.35000 15.09000 -37.06000 L

C 0 33.85000 15.25000 -38.63000 L

H 0 33.05000 14.69000 -38.87000 L

H 0 34.57000 15.12000 -39.31000 L

N 0 33.42000 16.66000 -38.72000 L

H 0 32.60000 16.94000 -38.21000 L

C 0 34.04000 17.58000 -39.43000 L

N 0 35.08000 17.22000 -40.13000 L

H 0 35.39000 16.27000 -40.11000 L

H 0 35.57000 17.90000 -40.68000 L

N 0 33.62000 18.81000 -39.44000 L

H 0 32.81000 19.07000 -38.90000 L

H 0 34.10000 19.50000 -39.98000 L

C 0 34.30000 14.89000 -33.69000 L

O 0 33.60000 15.34000 -32.76000 L

N 0 34.73000 13.63000 -33.70000 L

H 0 35.30000 13.27000 -34.44000 L

C 0 34.33000 12.80000 -32.57000 L

H 0 33.38000 13.11000 -32.51000 L

C 0 34.40000 11.25000 -32.86000 L

H 0 33.87000 10.75000 -32.17000 L

C 0 33.80000 10.94000 -34.21000 L

H 0 33.86000 9.95000 -34.37000 L

H 0 32.85000 11.23000 -34.22000 L

H 0 34.31000 11.42000 -34.92000 L

C 0 35.79000 10.69000 -32.73000 L

H 0 35.77000 9.71000 -32.92000 L

H 0 36.39000 11.15000 -33.38000 L

H 0 36.12000 10.85000 -31.80000 L

C 0 35.02000 13.13000 -31.24000 L

O 0 34.61000 12.62000 -30.23000 L

N 0 36.03000 13.99000 -31.23000 L

H 0 36.32000 14.38000 -32.11000 L

C 0 36.74000 14.39000 -30.03000 L

H 0 36.37000 13.85000 -29.27000 L

C 0 38.25000 14.14000 -30.26000 L

H 0 38.77000 14.49000 -29.48000 L

H 0 38.54000 14.61000 -31.09000 L

C 0 38.56000 12.69000 -30.40000 L

N 0 38.62000 11.84000 -29.32000 L

H 0 38.51000 12.11000 -28.37000 L

C 0 38.86000 10.59000 -29.76000 L

H 0 38.95000 9.78000 -29.18000 L

N 0 38.94000 10.62000 -31.09000 L

C 0 38.75000 11.91000 -31.53000 L

H 0 38.74000 12.22000 -32.48000 L

C 0 36.51000 15.86000 -29.76000 L

O 0 37.34000 16.51000 -29.12000 L

N 0 35.38000 16.38000 -30.21000 L

H 0 34.67000 15.79000 -30.61000 L

C 0 35.17000 17.81000 -30.12000 L

H 0 36.05000 18.23000 -29.88000 L

C 0 34.72000 18.34000 -31.49000 L

H 0 33.79000 18.02000 -31.68000 L

H 0 35.34000 18.00000 -32.20000 L

C 0 34.71000 19.79000 -31.60000 L

H 0 35.64000 20.10000 -31.39000 L

H 0 34.08000 20.12000 -30.90000 L

C 0 34.25000 20.27000 -33.08000 L

H 0 34.22000 21.27000 -33.14000 L

H 0 33.35000 19.90000 -33.32000 L

N 0 35.17000 19.84000 -34.12000 L

H 0 36.13000 19.75000 -33.88000 L

C 0 34.83000 19.54000 -35.39000 L

N 0 33.56000 19.57000 -35.79000 L

H 0 32.84000 19.81000 -35.15000 L

H 0 33.34000 19.34000 -36.74000 L

N 0 35.75000 19.18000 -36.28000 L

H 0 36.71000 19.12000 -36.01000 L

H 0 35.48000 18.96000 -37.22000 L

C 0 34.12000 18.09000 -29.00000 L

O 0 32.94000 17.86000 -29.19000 L

N 0 34.57000 18.59000 -27.82000 L

C 0 35.95000 18.95000 -27.49000 L

H 0 36.29000 19.62000 -28.16000 L

H 0 36.53000 18.14000 -27.50000 L

C 0 35.80000 19.57000 -26.06000 L

H 0 35.62000 20.55000 -26.10000 L

H 0 36.61000 19.40000 -25.51000 L

C 0 34.61000 18.85000 -25.49000 L

H 0 34.21000 19.36000 -24.73000 L

H 0 34.87000 17.93000 -25.18000 L

C 0 33.64000 18.77000 -26.68000 L

H 0 32.97000 18.03000 -26.64000 L

C 0 32.84000 20.09000 -26.78000 L

O 0 33.32000 21.04000 -27.40000 L

N 0 31.65000 20.13000 -26.16000 L

H 0 31.19000 19.28000 -25.91000 L

C 0 31.01000 21.44000 -25.83000 L

H 0 30.88000 21.95000 -26.68000 L

C 0 29.64000 21.21000 -25.14000 L

H 0 29.22000 22.09000 -24.93000 L

H 0 29.04000 20.70000 -25.76000 L

H 0 29.78000 20.69000 -24.30000 L

C 0 31.94000 22.22000 -24.89000 L

O 0 32.75000 21.62000 -24.17000 L

N 0 31.85000 23.55000 -24.94000 L

H 0 31.25000 23.97000 -25.62000 L

C 0 32.61000 24.43000 -24.02000 L

H 0 33.15000 23.82000 -23.43000 L

C 0 33.56000 25.37000 -24.80000 L

H 0 32.93000 25.84000 -25.43000 L

C 0 34.24000 26.52000 -23.92000 L

H 0 34.82000 27.08000 -24.50000 L

H 0 33.52000 27.09000 -23.51000 L

H 0 34.78000 26.10000 -23.19000 L

C 0 34.76000 24.49000 -25.56000 L

H 0 35.36000 25.11000 -26.06000 L

H 0 35.29000 24.00000 -24.87000 L

H 0 34.34000 23.84000 -26.19000 L

C 0 31.64000 25.26000 -23.18000 L

O 0 30.81000 26.00000 -23.72000 L

N 0 31.79000 25.18000 -21.84000 L

H 0 32.46000 24.53000 -21.46000 L

C 0 31.01000 26.01000 -20.92000 L

H 0 30.37000 26.56000 -21.46000 L

C 0 30.31000 25.09000 -19.95000 L

H 0 29.93000 25.64000 -19.21000 L

H 0 30.99000 24.46000 -19.58000 L

C 0 29.15000 24.26000 -20.62000 L

H 0 28.68000 24.83000 -21.29000 L

C 0 29.72000 22.98000 -21.34000 L

H 0 28.97000 22.47000 -21.76000 L

H 0 30.37000 23.26000 -22.05000 L

H 0 30.19000 22.40000 -20.67000 L

C 0 28.22000 23.78000 -19.46000 L

H 0 27.46000 23.25000 -19.84000 L

H 0 28.74000 23.21000 -18.83000 L

H 0 27.85000 24.58000 -18.98000 L

C 0 31.96000 26.91000 -20.13000 L

O 0 33.15000 26.61000 -20.00000 L

N 0 31.40000 27.98000 -19.58000 L

H 0 30.42000 28.14000 -19.70000 L

C 0 32.20000 28.94000 -18.77000 L

H 0 33.16000 28.69000 -18.80000 L

C 0 32.13000 30.41000 -19.33000 L

H 0 31.18000 30.70000 -19.41000 L

C 0 32.94000 31.34000 -18.37000 L

H 0 32.91000 32.28000 -18.71000 L

H 0 32.54000 31.31000 -17.45000 L

H 0 33.89000 31.04000 -18.33000 L

C 0 32.79000 30.46000 -20.72000 L

H 0 32.75000 31.39000 -21.07000 L

H 0 33.75000 30.17000 -20.64000 L

H 0 32.31000 29.84000 -21.34000 L

C 0 31.58000 28.84000 -17.37000 L

O 0 30.34000 28.79000 -17.23000 L

N 0 32.41000 28.56000 -16.37000 L

H 0 33.38000 28.38000 -16.53000 L

C 0 31.87000 28.54000 -15.02000 L

H 0 30.90000 28.35000 -15.12000 L

C 0 32.62000 27.41000 -14.22000 L

H 0 33.60000 27.62000 -14.18000 L

H 0 32.49000 26.53000 -14.68000 L

C 0 32.10000 27.29000 -12.82000 L

H 0 31.10000 27.35000 -12.83000 L

H 0 32.47000 28.04000 -12.27000 L

C 0 32.52000 25.97000 -12.21000 L

H 0 33.52000 25.94000 -12.17000 L

H 0 32.20000 25.23000 -12.81000 L

C 0 31.87000 25.84000 -10.72000 L

H 0 30.87000 25.85000 -10.75000 L

H 0 32.18000 26.57000 -10.12000 L

N 0 32.35000 24.47000 -10.16000 L

H 0 31.99000 24.34000 -9.25000 L

H 0 32.04000 23.74000 -10.77000 L

H 0 33.35000 24.47000 -10.13000 L

C 0 32.12000 29.93000 -14.32000 L

O 0 33.25000 30.40000 -14.21000 L

N 0 31.06000 30.53000 -13.75000 L

H 0 30.19000 30.03000 -13.69000 L

C 0 31.14000 31.86000 -13.22000 L

H 0 32.09000 32.11000 -13.39000 L

C 0 30.10000 32.76000 -13.93000 L

H 0 30.18000 33.70000 -13.61000 L

C 0 30.40000 32.73000 -15.49000 L

H 0 29.73000 33.30000 -15.97000 L

H 0 31.32000 33.07000 -15.66000 L

H 0 30.32000 31.79000 -15.82000 L

C 0 28.63000 32.30000 -13.56000 L

H 0 28.50000 32.35000 -12.57000 L

H 0 28.48000 31.37000 -13.88000 L

C 0 27.62000 33.28000 -14.29000 L

H 0 26.68000 33.01000 -14.07000 L

H 0 27.78000 34.22000 -13.97000 L

H 0 27.75000 33.24000 -15.28000 L

C 0 30.88000 31.92000 -11.71000 L

O 0 30.27000 31.03000 -11.10000 L

N 0 31.35000 32.99000 -11.14000 L

H 0 31.83000 33.67000 -11.70000 L

C 0 31.18000 33.22000 -9.72000 L

H 0 31.39000 32.35000 -9.29000 L

C 0 32.13000 34.47000 -9.36000 L

H 0 32.05000 34.67000 -8.38000 L

H 0 33.08000 34.25000 -9.57000 L

H 0 31.84000 35.27000 -9.88000 L

C 0 29.75000 33.62000 -9.37000 L

O 0 28.96000 34.09000 -10.21000 L

N 0 29.38000 33.50000 -8.08000 L

C 0 30.11000 32.74000 -7.07000 L

H 0 31.02000 33.14000 -6.93000 L

H 0 30.21000 31.79000 -7.36000 L

C 0 29.23000 32.87000 -5.81000 L

H 0 29.57000 33.60000 -5.22000 L

H 0 29.22000 32.00000 -5.31000 L

C 0 27.79000 33.22000 -6.35000 L

H 0 27.28000 33.77000 -5.69000 L

H 0 27.27000 32.39000 -6.57000 L

C 0 28.10000 34.04000 -7.65000 L

H 0 27.46000 33.97000 -8.41000 L

C 0 28.29000 35.52000 -7.18000 L

O 0 27.34000 36.11000 -6.67000 L

N 0 29.47000 36.07000 -7.29000 L

H 0 30.20000 35.58000 -7.78000 L

C 0 29.75000 37.39000 -6.69000 L

H 0 28.97000 37.59000 -6.09000 L

C 0 31.05000 37.31000 -5.89000 L

H 0 31.22000 38.20000 -5.46000 L

H 0 31.80000 37.08000 -6.51000 L

C 0 30.95000 36.21000 -4.78000 L

O 0 29.88000 36.12000 -4.14000 L

O 0 31.93000 35.46000 -4.62000 L

C 0 29.82000 38.53000 -7.75000 L

O 0 30.46000 39.57000 -7.57000 L

N 0 29.21000 38.35000 -8.89000 L

H 0 28.56000 37.60000 -9.01000 L

C 0 29.49000 39.30000 -10.02000 L

H 0 30.44000 39.59000 -9.95000 L

C 0 29.11000 38.62000 -11.35000 L

H 0 29.27000 39.27000 -12.09000 L

H 0 28.13000 38.40000 -11.31000 L

C 0 29.89000 37.31000 -11.67000 L

H 0 29.82000 36.70000 -10.87000 L

C 0 29.30000 36.46000 -12.85000 L

H 0 29.86000 35.64000 -12.98000 L

H 0 28.36000 36.19000 -12.63000 L

H 0 29.30000 37.01000 -13.68000 L

C 0 31.34000 37.66000 -11.89000 L

H 0 31.86000 36.83000 -12.10000 L

H 0 31.41000 38.30000 -12.66000 L

H 0 31.71000 38.09000 -11.07000 L

C 0 28.55000 40.45000 -9.91000 L

O 0 27.44000 40.29000 -9.46000 L

N 0 28.97000 41.62000 -10.41000 L

H 0 29.92000 41.72000 -10.72000 L

C 0 28.03000 42.74000 -10.49000 L

H 0 27.59000 42.76000 -9.59000 L

C 0 28.76000 44.04000 -10.76000 L

H 0 28.09000 44.78000 -10.84000 L

C 0 29.90000 44.29000 -9.65000 L

H 0 30.37000 45.15000 -9.85000 L

H 0 29.48000 44.34000 -8.74000 L

H 0 30.56000 43.54000 -9.67000 L

O 0 29.39000 43.95000 -12.02000 L

H 0 29.88000 44.81000 -12.21000 L

C 0 26.96000 42.52000 -11.55000 L

O 0 27.09000 41.68000 -12.43000 L

N 0 25.92000 43.34000 -11.55000 L

H 0 25.79000 44.01000 -10.83000 L

C 0 24.96000 43.22000 -12.65000 L

H 0 24.62000 42.28000 -12.63000 L

C 0 23.81000 44.22000 -12.47000 L

H 0 23.11000 44.12000 -13.17000 L

H 0 24.14000 45.17000 -12.46000 L

O 0 23.23000 43.92000 -11.20000 L

H 0 22.47000 44.55000 -11.03000 L

C 0 25.68000 43.51000 -13.97000 L

O 0 25.33000 42.88000 -15.01000 L

N 0 26.61000 44.46000 -13.98000 L

H 0 26.83000 44.95000 -13.14000 L

C 0 27.32000 44.78000 -15.26000 L

H 0 26.62000 45.01000 -15.94000 L

C 0 28.24000 45.96000 -15.00000 L

H 0 28.84000 45.75000 -14.23000 L

H 0 27.69000 46.77000 -14.78000 L

C 0 29.07000 46.28000 -16.15000 L

H 0 29.43000 45.42000 -16.52000 L

H 0 29.83000 46.84000 -15.83000 L

C 0 28.35000 47.00000 -17.21000 L

O 0 28.24000 48.26000 -17.19000 L

N 0 27.96000 46.26000 -18.25000 L

H 0 28.16000 45.28000 -18.26000 L

H 0 27.46000 46.68000 -19.01000 L

C 0 28.15000 43.57000 -15.79000 L

O 0 28.25000 43.28000 -17.02000 L

N 0 28.78000 42.85000 -14.84000 L

H 0 28.67000 43.09000 -13.88000 L

C 0 29.61000 41.71000 -15.19000 L

H 0 30.30000 42.10000 -15.79000 L

C 0 30.21000 41.02000 -13.91000 L

H 0 30.61000 40.15000 -14.18000 L

H 0 29.47000 40.86000 -13.25000 L

C 0 31.29000 41.85000 -13.20000 L

O 0 31.96000 42.65000 -13.85000 L

O 0 31.48000 41.62000 -11.96000 L

C 0 28.72000 40.62000 -15.82000 L

O 0 29.05000 40.00000 -16.88000 L

N 0 27.56000 40.42000 -15.23000 L

H 0 27.30000 40.96000 -14.42000 L

C 0 26.64000 39.40000 -15.75000 L

H 0 27.17000 38.55000 -15.79000 L

C 0 25.44000 39.21000 -14.80000 L

H 0 24.74000 38.68000 -15.27000 L

H 0 25.07000 40.11000 -14.57000 L

C 0 25.92000 38.45000 -13.48000 L

H 0 26.66000 38.96000 -13.04000 L

H 0 26.24000 37.53000 -13.70000 L

C 0 24.76000 38.33000 -12.48000 L

H 0 24.69000 37.39000 -12.16000 L

H 0 23.90000 38.59000 -12.92000 L

C 0 25.08000 39.29000 -11.29000 L

H 0 24.97000 40.24000 -11.58000 L

H 0 26.01000 39.14000 -10.97000 L

N 0 24.14000 39.04000 -10.16000 L

H 0 24.35000 39.66000 -9.41000 L

H 0 23.20000 39.19000 -10.46000 L

H 0 24.24000 38.09000 -9.85000 L

C 0 26.15000 39.77000 -17.16000 L

O 0 26.06000 38.89000 -18.06000 L

N 0 25.76000 41.04000 -17.35000 L

H 0 25.72000 41.66000 -16.57000 L

C 0 25.38000 41.52000 -18.70000 L

H 0 24.54000 41.00000 -18.88000 L

C 0 25.21000 43.07000 -18.75000 L

H 0 25.17000 43.38000 -19.70000 L

H 0 25.97000 43.51000 -18.29000 L

C 0 24.00000 43.54000 -18.11000 L

H 0 24.01000 43.20000 -17.17000 L

H 0 23.23000 43.14000 -18.61000 L

C 0 23.88000 45.09000 -18.11000 L

O 0 23.06000 45.44000 -17.28000 L

O 0 24.60000 45.87000 -18.87000 L

C 0 26.48000 41.29000 -19.72000 L

O 0 26.15000 40.86000 -20.84000 L

N 0 27.73000 41.63000 -19.37000 L

H 0 27.88000 42.02000 -18.46000 L

C 0 28.88000 41.46000 -20.27000 L

H 0 28.63000 41.96000 -21.10000 L

C 0 30.17000 42.05000 -19.67000 L

H 0 30.94000 41.78000 -20.25000 L

H 0 30.29000 41.67000 -18.75000 L

C 0 30.10000 43.59000 -19.59000 L

O 0 29.14000 44.11000 -20.16000 L

O 0 30.94000 44.18000 -18.98000 L

C 0 29.09000 39.99000 -20.58000 L

O 0 29.34000 39.63000 -21.72000 L

N 0 29.02000 39.13000 -19.56000 L

H 0 28.89000 39.45000 -18.63000 L

C 0 29.13000 37.68000 -19.84000 L

H 0 29.99000 37.62000 -20.34000 L

C 0 29.17000 36.84000 -18.50000 L

H 0 28.41000 37.11000 -17.91000 L

C 0 28.99000 35.32000 -18.78000 L

H 0 29.02000 34.82000 -17.91000 L

H 0 28.11000 35.15000 -19.22000 L

H 0 29.73000 35.00000 -19.37000 L

C 0 30.53000 37.12000 -17.81000 L

H 0 30.78000 38.07000 -17.98000 L

H 0 31.22000 36.52000 -18.21000 L

C 0 30.41000 36.86000 -16.19000 L

H 0 31.30000 37.05000 -15.76000 L

H 0 29.72000 37.47000 -15.80000 L

H 0 30.16000 35.91000 -16.02000 L

C 0 28.06000 37.14000 -20.79000 L

O 0 28.35000 36.37000 -21.74000 L

N 0 26.82000 37.53000 -20.57000 L

H 0 26.63000 38.16000 -19.81000 L

C 0 25.76000 37.08000 -21.40000 L

H 0 25.75000 36.09000 -21.31000 L

C 0 24.40000 37.65000 -20.94000 L

H 0 23.68000 37.32000 -21.55000 L

H 0 24.22000 37.36000 -20.00000 L

H 0 24.43000 38.65000 -20.98000 L

C 0 26.04000 37.51000 -22.83000 L

O 0 25.88000 36.72000 -23.72000 L

N 0 26.49000 38.75000 -23.01000 L

H 0 26.68000 39.33000 -22.22000 L

C 0 26.72000 39.27000 -24.34000 L

H 0 25.86000 39.16000 -24.84000 L

C 0 27.10000 40.75000 -24.20000 L

H 0 27.79000 40.86000 -23.49000 L

H 0 26.29000 41.29000 -23.96000 L

O 0 27.64000 41.27000 -25.43000 L

H 0 27.88000 42.23000 -25.31000 L

C 0 27.89000 38.49000 -25.05000 L

O 0 27.80000 38.11000 -26.21000 L

N 0 28.96000 38.28000 -24.35000 L

H 0 29.02000 38.63000 -23.41000 L

C 0 30.10000 37.53000 -24.90000 L

H 0 30.35000 37.99000 -25.76000 L

C 0 31.31000 37.61000 -23.93000 L

H 0 30.97000 37.35000 -23.03000 L

C 0 32.53000 36.65000 -24.48000 L

H 0 33.31000 36.70000 -23.85000 L

H 0 32.21000 35.70000 -24.54000 L

H 0 32.82000 36.96000 -25.39000 L

C 0 31.82000 39.05000 -23.83000 L

H 0 32.60000 39.09000 -23.20000 L

H 0 32.11000 39.37000 -24.73000 L

H 0 31.09000 39.65000 -23.48000 L

C 0 29.68000 36.11000 -25.28000 L

O 0 29.97000 35.60000 -26.38000 L

N 0 28.93000 35.44000 -24.41000 L

H 0 28.62000 35.91000 -23.58000 L

C 0 28.55000 34.08000 -24.62000 L

H 0 29.39000 33.55000 -24.76000 L

C 0 27.76000 33.64000 -23.36000 L

H 0 27.18000 34.36000 -22.98000 L

C 0 26.75000 32.57000 -23.72000 L

H 0 26.25000 32.30000 -22.90000 L

H 0 26.11000 32.93000 -24.40000 L

H 0 27.23000 31.78000 -24.10000 L

C 0 28.80000 33.26000 -22.30000 L

H 0 28.34000 32.96000 -21.46000 L

H 0 29.37000 32.51000 -22.64000 L

H 0 29.38000 34.05000 -22.09000 L

C 0 27.67000 33.98000 -25.86000 L

O 0 27.81000 33.05000 -26.70000 L

N 0 26.79000 34.95000 -26.04000 L

H 0 26.69000 35.69000 -25.37000 L

C 0 25.96000 34.93000 -27.26000 L

H 0 25.70000 33.97000 -27.39000 L

C 0 24.68000 35.80000 -27.08000 L

H 0 24.23000 35.92000 -27.96000 L

H 0 24.94000 36.69000 -26.71000 L

C 0 23.73000 34.99000 -26.05000 L

H 0 24.23000 34.83000 -25.19000 L

H 0 23.47000 34.12000 -26.45000 L

C 0 22.42000 35.76000 -25.69000 L

H 0 21.95000 35.30000 -24.93000 L

H 0 21.82000 35.79000 -26.48000 L

C 0 22.69000 37.18000 -25.26000 L

H 0 23.36000 37.60000 -25.88000 L

H 0 23.05000 37.20000 -24.33000 L

N 0 21.37000 37.92000 -25.34000 L

H 0 21.50000 38.87000 -25.07000 L

H 0 21.02000 37.89000 -26.28000 L

H 0 20.70000 37.49000 -24.73000 L

C 0 26.74000 35.31000 -28.51000 L

O 0 26.57000 34.66000 -29.55000 L

N 0 27.60000 36.31000 -28.43000 L

H 0 27.69000 36.80000 -27.56000 L

C 0 28.43000 36.73000 -29.56000 L

H 0 27.81000 37.07000 -30.27000 L

C 0 29.35000 37.82000 -29.08000 L

H 0 29.85000 37.46000 -28.29000 L

H 0 28.77000 38.58000 -28.78000 L

C 0 30.36000 38.34000 -30.10000 L

H 0 29.88000 38.84000 -30.82000 L

H 0 30.86000 37.57000 -30.50000 L

C 0 31.35000 39.26000 -29.47000 L

O 0 31.13000 39.67000 -28.31000 L

O 0 32.37000 39.58000 -30.10000 L

C 0 29.33000 35.52000 -30.06000 L

O 0 29.53000 35.30000 -31.30000 L

N 0 29.86000 34.72000 -29.10000 L

H 0 29.60000 34.83000 -28.14000 L

C 0 30.83000 33.69000 -29.48000 L

H 0 31.19000 34.00000 -30.36000 L

C 0 31.95000 33.61000 -28.45000 L

H 0 32.54000 32.85000 -28.69000 L

H 0 31.54000 33.45000 -27.55000 L

C 0 32.82000 34.86000 -28.34000 L

H 0 32.15000 35.52000 -28.01000 L

C 0 34.00000 34.68000 -27.27000 L

H 0 34.54000 35.52000 -27.23000 L

H 0 33.61000 34.49000 -26.37000 L

H 0 34.58000 33.92000 -27.55000 L

C 0 33.40000 35.41000 -29.72000 L

H 0 33.95000 36.22000 -29.55000 L

H 0 33.96000 34.70000 -30.15000 L

H 0 32.64000 35.65000 -30.32000 L

C 0 30.18000 32.33000 -29.72000 L

O 0 30.83000 31.43000 -30.25000 L

N 0 28.93000 32.18000 -29.34000 L

H 0 28.42000 32.96000 -28.98000 L

C 0 28.27000 30.89000 -29.45000 L

H 0 28.43000 30.54000 -30.38000 L

H 0 27.29000 31.03000 -29.31000 L

C 0 28.85000 29.91000 -28.38000 L

O 0 28.90000 28.66000 -28.61000 L

N 0 29.29000 30.43000 -27.24000 L

H 0 29.41000 31.41000 -27.15000 L

C 0 29.59000 29.50000 -26.08000 L

H 0 30.39000 28.94000 -26.31000 L

C 0 29.90000 30.34000 -24.78000 L

H 0 29.20000 31.04000 -24.63000 L

C 0 29.96000 29.48000 -23.50000 L

H 0 30.16000 30.07000 -22.71000 L

H 0 29.07000 29.03000 -23.37000 L

H 0 30.67000 28.79000 -23.59000 L

C 0 31.28000 30.95000 -24.99000 L

H 0 31.38000 31.69000 -24.33000 L

H 0 31.30000 31.32000 -25.91000 L

C 0 32.48000 29.79000 -24.78000 L

H 0 33.38000 30.21000 -24.92000 L

H 0 32.43000 29.42000 -23.85000 L

H 0 32.35000 29.05000 -25.44000 L

C 0 28.45000 28.50000 -25.89000 L

O 0 27.27000 28.87000 -25.92000 L

N 0 28.75000 27.24000 -25.61000 L

H 0 29.70000 26.97000 -25.48000 L

C 0 27.70000 26.24000 -25.50000 L

H 0 27.09000 26.52000 -26.25000 L

C 0 28.31000 24.84000 -25.64000 L

H 0 27.57000 24.16000 -25.60000 L

H 0 28.93000 24.69000 -24.87000 L

C 0 29.12000 24.69000 -27.02000 L

O 0 28.44000 24.86000 -28.06000 L

O 0 30.36000 24.56000 -26.98000 L

C 0 26.89000 26.23000 -24.19000 L

O 0 25.79000 25.70000 -24.18000 L

N 0 27.45000 26.75000 -23.09000 L

H 0 28.36000 27.14000 -23.13000 L

C 0 26.71000 26.73000 -21.85000 L

H 0 26.56000 25.77000 -21.59000 L

H 0 25.83000 27.18000 -22.00000 L

C 0 27.47000 27.44000 -20.75000 L

O 0 28.59000 27.85000 -20.94000 L

N 0 26.84000 27.56000 -19.59000 L

H 0 25.91000 27.19000 -19.53000 L

C 0 27.39000 28.21000 -18.42000 L

H 0 28.34000 28.42000 -18.60000 L

C 0 26.50000 29.46000 -18.10000 L

H 0 26.76000 29.81000 -17.20000 L

H 0 25.54000 29.17000 -18.08000 L

C 0 26.66000 30.60000 -19.14000 L

H 0 26.56000 30.21000 -20.06000 L

C 0 25.57000 31.71000 -18.90000 L

H 0 25.68000 32.43000 -19.58000 L

H 0 24.66000 31.31000 -18.99000 L

H 0 25.68000 32.09000 -17.98000 L

C 0 28.04000 31.25000 -19.02000 L

H 0 28.13000 31.98000 -19.70000 L

H 0 28.15000 31.63000 -18.10000 L

H 0 28.75000 30.56000 -19.18000 L

C 0 27.22000 27.26000 -17.24000 L

O 0 26.24000 26.54000 -17.12000 L

N 0 28.18000 27.31000 -16.34000 L

H 0 29.01000 27.83000 -16.52000 L

C 0 27.99000 26.61000 -15.10000 L

H 0 27.18000 26.04000 -15.20000 L

C 0 29.20000 25.71000 -14.80000 L

H 0 30.04000 26.25000 -14.78000 L

C 0 28.96000 25.01000 -13.38000 L

H 0 29.74000 24.41000 -13.16000 L

H 0 28.87000 25.71000 -12.67000 L

H 0 28.12000 24.46000 -13.41000 L

C 0 29.30000 24.64000 -15.95000 L

H 0 29.75000 25.06000 -16.74000 L

H 0 28.37000 24.37000 -16.21000 L

C 0 30.09000 23.35000 -15.58000 L

H 0 30.11000 22.73000 -16.36000 L

H 0 31.03000 23.60000 -15.34000 L

H 0 29.65000 22.90000 -14.80000 L

C 0 27.89000 27.72000 -14.00000 L

O 0 28.74000 28.64000 -13.93000 L

N 0 26.84000 27.61000 -13.17000 L

H 0 26.32000 26.76000 -13.12000 L

C 0 26.46000 28.79000 -12.31000 L

H 0 27.20000 29.45000 -12.18000 L

C 0 25.23000 29.46000 -13.01000 L

H 0 24.48000 28.80000 -12.90000 L

C 0 24.76000 30.76000 -12.29000 L

H 0 23.98000 31.15000 -12.78000 L

H 0 24.50000 30.55000 -11.35000 L

H 0 25.51000 31.43000 -12.29000 L

C 0 25.54000 29.79000 -14.57000 L

H 0 24.74000 30.22000 -14.99000 L

H 0 26.32000 30.42000 -14.63000 L

H 0 25.76000 28.94000 -15.05000 L

C 0 25.99000 28.14000 -10.99000 L

O 0 24.89000 27.58000 -10.94000 L

N 0 26.79000 28.20000 -9.91000 L

H 0 26.55000 27.62000 -9.13000 L

C 0 27.97000 29.05000 -9.80000 L

H 0 28.16000 29.25000 -10.76000 L

C 0 27.74000 30.28000 -8.85000 L

H 0 28.59000 30.81000 -8.79000 L

C 0 26.45000 31.07000 -9.35000 L

H 0 26.29000 31.85000 -8.76000 L

H 0 26.60000 31.38000 -10.29000 L

H 0 25.66000 30.46000 -9.33000 L

O 0 27.42000 29.86000 -7.50000 L

H 0 27.28000 30.67000 -6.92000 L

C 0 29.07000 28.35000 -9.09000 L

O 0 28.86000 27.31000 -8.47000 L

N 0 30.24000 29.00000 -9.11000 L

H 0 30.38000 29.75000 -9.76000 L

C 0 31.33000 28.62000 -8.18000 L

H 0 31.39000 27.62000 -8.21000 L

C 0 32.65000 29.30000 -8.68000 L

H 0 32.80000 30.14000 -8.17000 L

H 0 32.57000 29.50000 -9.65000 L

C 0 33.88000 28.38000 -8.47000 L

O 0 34.10000 27.85000 -7.35000 L

N 0 34.64000 28.15000 -9.56000 L

H 0 34.41000 28.56000 -10.43000 L

H 0 35.45000 27.56000 -9.48000 L

C 0 31.00000 28.99000 -6.70000 L

O 0 29.89000 29.43000 -6.35000 L

N 0 32.00000 28.85000 -5.82000 L

H 0 32.87000 28.47000 -6.10000 L

C 0 31.80000 29.26000 -4.44000 L

H 0 30.88000 28.92000 -4.24000 L

C 0 32.93000 28.64000 -3.54000 L

H 0 32.91000 28.98000 -2.60000 L

C 0 32.81000 27.14000 -3.48000 L

H 0 33.54000 26.77000 -2.90000 L

H 0 31.92000 26.89000 -3.10000 L

H 0 32.90000 26.77000 -4.40000 L

O 0 34.22000 28.98000 -4.11000 L

H 0 34.95000 28.59000 -3.55000 L

C 0 31.85000 30.81000 -4.29000 L

O 0 32.30000 31.55000 -5.22000 L

N 0 31.38000 31.31000 -3.14000 L

H 0 31.14000 30.69000 -2.40000 L

C 0 31.23000 32.75000 -2.94000 L

H 0 31.31000 33.14000 -3.85000 L

C 0 29.83000 33.15000 -2.38000 L

H 0 29.13000 32.94000 -3.06000 L

C 0 29.55000 32.52000 -0.99000 L

H 0 28.64000 32.80000 -0.67000 L

H 0 29.58000 31.52000 -1.06000 L

H 0 30.24000 32.83000 -0.34000 L

O 0 29.85000 34.51000 -2.12000 L

H 0 28.96000 34.79000 -1.76000 L

C 0 32.34000 33.24000 -1.97000 L

O 0 32.69000 32.57000 -0.94000 L

N 0 32.88000 34.41000 -2.26000 L

H 0 32.66000 34.86000 -3.12000 L

C 0 33.79000 35.03000 -1.32000 L

H 0 34.22000 34.24000 -0.87000 L

C 0 34.89000 35.92000 -2.01000 L

H 0 35.50000 36.29000 -1.31000 L

C 0 35.75000 35.03000 -2.88000 L

H 0 36.45000 35.58000 -3.32000 L

H 0 36.18000 34.33000 -2.31000 L

H 0 35.17000 34.59000 -3.57000 L

C 0 34.25000 37.14000 -2.86000 L

H 0 34.99000 37.68000 -3.28000 L

H 0 33.66000 36.78000 -3.58000 L

H 0 33.72000 37.73000 -2.26000 L

C 0 32.97000 35.89000 -0.34000 L

O 0 33.57000 36.49000 0.61000 L

N 0 31.65000 35.92000 -0.47000 L

H 0 31.20000 35.44000 -1.22000 L

C 0 30.84000 36.69000 0.51000 L

H 0 31.33000 37.54000 0.71000 L

C 0 29.41000 36.97000 -0.10000 L

H 0 29.48000 37.30000 -1.05000 L

H 0 28.91000 37.64000 0.44000 L

O 0 28.63000 35.75000 -0.12000 L

H 0 27.73000 35.93000 -0.51000 L

C 0 30.70000 35.86000 1.80000 L

O 0 30.99000 34.66000 1.78000 L

N 0 30.25000 36.45000 2.93000 L

H 0 30.06000 37.43000 2.93000 L

C 0 30.03000 35.67000 4.16000 L

H 0 30.06000 34.69000 3.97000 L

C 0 31.13000 35.93000 5.24000 L

H 0 30.88000 35.49000 6.10000 L

H 0 31.24000 36.92000 5.40000 L

C 0 32.44000 35.39000 4.80000 L

H 0 33.14000 35.74000 5.43000 L

H 0 32.62000 35.74000 3.88000 L

C 0 32.47000 33.82000 4.78000 L

H 0 31.85000 33.47000 4.09000 L

H 0 32.21000 33.46000 5.68000 L

N 0 33.84000 33.32000 4.47000 L

H 0 34.45000 33.18000 5.24000 L

C 0 34.32000 33.06000 3.26000 L

N 0 35.62000 32.61000 3.08000 L

H 0 36.21000 32.48000 3.88000 L

H 0 35.96000 32.41000 2.17000 L

N 0 33.53000 33.25000 2.18000 L

H 0 32.60000 33.59000 2.30000 L

H 0 33.88000 33.05000 1.26000 L

C 0 28.64000 36.21000 4.60000 L

O 0 28.58000 37.21000 5.31000 L

N 0 27.54000 35.57000 4.12000 L

C 0 27.54000 34.44000 3.19000 L

H 0 27.96000 33.65000 3.62000 L

H 0 28.06000 34.69000 2.37000 L

C 0 26.00000 34.24000 2.91000 L

H 0 25.76000 33.27000 2.84000 L

H 0 25.71000 34.72000 2.08000 L

C 0 25.31000 34.82000 4.06000 L

H 0 25.28000 34.17000 4.82000 L

H 0 24.38000 35.08000 3.81000 L

C 0 26.17000 36.05000 4.40000 L

H 0 25.95000 36.89000 3.90000 L

C 0 26.05000 36.30000 5.86000 L

O 0 26.61000 35.56000 6.67000 L

N 0 25.23000 37.29000 6.23000 L

H 0 24.83000 37.89000 5.54000 L

C 0 24.89000 37.52000 7.66000 L

H 0 25.71000 37.73000 8.19000 L

C 0 23.93000 38.72000 7.77000 L

H 0 23.71000 38.88000 8.74000 L

H 0 24.37000 39.54000 7.40000 L

H 0 23.09000 38.53000 7.26000 L

C 0 24.24000 36.23000 8.13000 L

O 0 23.53000 35.60000 7.35000 L

N 0 24.55000 35.79000 9.35000 L

H 0 25.29000 36.26000 9.84000 L

C 0 23.90000 34.69000 10.01000 L

H 0 23.07000 34.49000 9.49000 L

H 0 23.65000 34.99000 10.93000 L

C 0 24.77000 33.45000 10.09000 L

O 0 24.36000 32.47000 10.69000 L

N 0 25.90000 33.42000 9.38000 L

H 0 26.13000 34.15000 8.74000 L

C 0 26.83000 32.24000 9.55000 L

H 0 26.32000 31.43000 9.24000 L

C 0 28.12000 32.38000 8.74000 L

H 0 28.69000 31.58000 8.95000 L

H 0 28.58000 33.21000 9.06000 L

C 0 27.98000 32.48000 7.18000 L

H 0 27.34000 33.22000 7.01000 L

C 0 29.35000 32.75000 6.51000 L

H 0 29.23000 32.81000 5.52000 L

H 0 29.72000 33.62000 6.85000 L

H 0 29.99000 32.01000 6.73000 L

C 0 27.50000 31.11000 6.57000 L

H 0 27.42000 31.19000 5.58000 L

H 0 28.16000 30.39000 6.79000 L

H 0 26.61000 30.86000 6.96000 L

C 0 27.17000 32.05000 10.99000 L

O 0 27.58000 32.99000 11.65000 L

N 0 27.10000 30.80000 11.47000 L

H 0 26.85000 30.04000 10.87000 L

C 0 27.40000 30.56000 12.89000 L

H 0 27.48000 31.48000 13.27000 L

C 0 26.26000 29.62000 13.52000 L

H 0 26.47000 29.42000 14.47000 L

H 0 26.20000 28.77000 13.00000 L

C 0 24.90000 30.28000 13.47000 L

H 0 24.24000 29.70000 13.93000 L

H 0 24.63000 30.41000 12.52000 L

C 0 24.98000 31.64000 14.18000 L

O 0 25.39000 31.65000 15.30000 L

N 0 24.64000 32.75000 13.50000 L

H 0 24.34000 32.68000 12.55000 L

H 0 24.68000 33.64000 13.95000 L

C 0 28.71000 29.78000 13.08000 L

O 0 29.25000 29.80000 14.21000 L

N 0 29.22000 29.11000 12.03000 L

H 0 28.83000 29.20000 11.11000 L

C 0 30.37000 28.23000 12.27000 L

H 0 30.63000 27.79000 11.41000 L

H 0 30.10000 27.53000 12.93000 L

C 0 31.56000 29.05000 12.82000 L

O 0 31.74000 30.22000 12.49000 L

N 0 32.34000 28.43000 13.72000 L

H 0 32.16000 27.48000 13.97000 L

C 0 33.49000 29.17000 14.34000 L

H 0 33.14000 30.00000 14.76000 L

C 0 34.19000 28.20000 15.42000 L

H 0 34.96000 28.68000 15.85000 L

H 0 33.52000 27.96000 16.13000 L

H 0 34.52000 27.37000 14.97000 L

C 0 34.48000 29.60000 13.25000 L

O 0 35.23000 30.62000 13.35000 L

N 0 34.48000 28.85000 12.14000 L

H 0 33.79000 28.14000 12.03000 L

C 0 35.50000 29.07000 11.08000 L

H 0 36.26000 29.54000 11.52000 L

C 0 35.88000 27.66000 10.48000 L

H 0 36.60000 27.76000 9.79000 L

H 0 35.07000 27.24000 10.05000 L

C 0 36.39000 26.76000 11.64000 L

H 0 35.68000 26.61000 12.33000 L

C 0 36.77000 25.33000 11.10000 L

H 0 37.09000 24.76000 11.85000 L

H 0 35.97000 24.90000 10.68000 L

H 0 37.49000 25.42000 10.41000 L

C 0 37.63000 27.53000 12.27000 L

H 0 38.00000 26.99000 13.03000 L

H 0 38.34000 27.65000 11.57000 L

H 0 37.34000 28.42000 12.61000 L

C 0 35.00000 29.97000 9.95000 L

O 0 35.56000 29.99000 8.84000 L

N 0 33.90000 30.68000 10.17000 L

H 0 33.45000 30.65000 11.07000 L

C 0 33.32000 31.51000 9.10000 L

H 0 33.19000 30.85000 8.36000 L

C 0 32.02000 32.12000 9.60000 L

H 0 31.46000 31.39000 9.99000 L

H 0 31.54000 32.52000 8.82000 L

C 0 32.27000 33.24000 10.70000 L

H 0 32.74000 34.02000 10.30000 L

H 0 32.82000 32.87000 11.45000 L

C 0 30.96000 33.73000 11.28000 L

H 0 30.51000 32.99000 11.78000 L

H 0 30.36000 34.05000 10.55000 L

N 0 31.22000 34.87000 12.24000 L

H 0 32.12000 35.30000 12.19000 L

C 0 30.36000 35.34000 13.12000 L

N 0 30.74000 36.37000 13.93000 L

H 0 31.66000 36.75000 13.84000 L

H 0 30.11000 36.74000 14.60000 L

N 0 29.12000 34.82000 13.19000 L

H 0 28.85000 34.08000 12.57000 L

H 0 28.46000 35.18000 13.86000 L

C 0 34.23000 32.61000 8.53000 L

O 0 34.02000 33.02000 7.39000 L

N 0 35.24000 33.09000 9.28000 L

H 0 35.42000 32.70000 10.18000 L

C 0 36.08000 34.15000 8.76000 L

H 0 35.56000 34.58000 8.02000 L

C 0 36.37000 35.16000 9.92000 L

H 0 37.08000 35.81000 9.67000 L

H 0 36.62000 34.69000 10.76000 L

O 0 35.11000 35.87000 10.12000 L

H 0 35.23000 36.54000 10.85000 L

C 0 37.40000 33.60000 8.15000 L

O 0 38.29000 34.37000 7.83000 L

N 0 37.51000 32.29000 7.97000 L

H 0 36.85000 31.67000 8.41000 L

C 0 38.58000 31.72000 7.13000 L

H 0 39.42000 32.03000 7.59000 L

C 0 38.52000 30.16000 7.04000 L

H 0 39.18000 29.85000 6.36000 L

H 0 37.59000 29.89000 6.76000 L

C 0 38.84000 29.45000 8.38000 L

H 0 38.64000 28.47000 8.28000 L

H 0 38.25000 29.83000 9.10000 L

C 0 40.37000 29.65000 8.78000 L

O 0 40.71000 29.50000 9.96000 L

O 0 41.16000 30.07000 7.92000 L

C 0 38.47000 32.26000 5.71000 L

O 0 37.36000 32.38000 5.17000 L

N 0 39.64000 32.59000 5.09000 L

H 0 40.49000 32.59000 5.62000 L

C 0 39.70000 32.93000 3.67000 L

H 0 39.02000 33.65000 3.53000 L

C 0 41.19000 33.38000 3.34000 L

H 0 41.87000 32.78000 3.77000 L

C 0 41.46000 33.45000 1.80000 L

H 0 42.40000 33.74000 1.64000 L

H 0 41.31000 32.55000 1.39000 L

H 0 40.84000 34.12000 1.38000 L

O 0 41.29000 34.73000 3.87000 L

H 0 42.21000 35.08000 3.70000 L

C 0 39.32000 31.72000 2.79000 L

O 0 39.77000 30.62000 3.06000 L

N 0 38.60000 31.96000 1.68000 L

H 0 38.25000 32.88000 1.51000 L

C 0 38.33000 30.88000 0.72000 L

H 0 38.43000 30.00000 1.19000 L

H 0 38.99000 30.94000 -0.03000 L

C 0 36.93000 31.00000 0.17000 L

O 0 36.22000 32.03000 0.43000 L

N 0 36.51000 29.99000 -0.59000 L

H 0 37.10000 29.21000 -0.74000 L

C 0 35.17000 30.03000 -1.20000 L

H 0 35.18000 29.58000 -2.09000 L

H 0 34.87000 30.98000 -1.31000 L

C 0 34.28000 29.30000 -0.24000 L

O 0 34.63000 28.18000 0.22000 L

N 0 33.11000 29.89000 -0.02000 L

H 0 32.93000 30.79000 -0.42000 L

C 0 32.06000 29.25000 0.80000 L

H 0 32.49000 28.57000 1.40000 L

C 0 31.32000 30.42000 1.57000 L

H 0 31.01000 31.10000 0.90000 L

H 0 31.95000 30.84000 2.21000 L

C 0 30.08000 29.93000 2.36000 L

H 0 29.59000 29.21000 1.87000 L

C 0 30.52000 29.42000 3.68000 L

H 0 29.72000 29.10000 4.20000 L

H 0 31.15000 28.66000 3.55000 L

H 0 30.98000 30.15000 4.19000 L

C 0 29.29000 31.25000 2.71000 L

H 0 28.47000 31.02000 3.22000 L

H 0 29.87000 31.85000 3.25000 L

H 0 29.03000 31.71000 1.86000 L

C 0 31.09000 28.52000 -0.08000 L

O 0 30.66000 29.08000 -1.12000 L

N 0 30.75000 27.26000 0.29000 L

H 0 31.15000 26.88000 1.12000 L

C 0 29.85000 26.46000 -0.46000 L

H 0 29.51000 27.06000 -1.19000 L

C 0 30.64000 25.25000 -1.01000 L

H 0 31.43000 25.56000 -1.54000 L

H 0 30.05000 24.68000 -1.58000 L

O 0 31.13000 24.44000 0.09000 L

H 0 31.64000 23.66000 -0.28000 L

C 0 28.70000 25.92000 0.48000 L

O 0 28.74000 26.12000 1.70000 L

N 0 27.74000 25.17000 -0.06000 L

H 0 27.71000 24.97000 -1.04000 L

C 0 26.71000 24.65000 0.89000 L

H 0 27.13000 24.56000 1.79000 L

H 0 26.41000 23.76000 0.57000 L

C 0 25.51000 25.56000 1.00000 L

O 0 25.25000 26.39000 0.15000 L

N 0 24.68000 25.29000 2.00000 L

H 0 24.95000 24.62000 2.70000 L

C 0 23.37000 25.96000 2.09000 L

H 0 22.93000 25.78000 1.21000 L

C 0 22.61000 25.38000 3.37000 L

H 0 23.10000 25.68000 4.19000 L

H 0 22.62000 24.38000 3.31000 L

C 0 21.06000 25.91000 3.43000 L

H 0 20.55000 25.49000 2.68000 L

H 0 21.05000 26.90000 3.32000 L

C 0 20.30000 25.56000 4.79000 L

H 0 19.43000 26.05000 4.78000 L

H 0 20.86000 25.89000 5.55000 L

C 0 20.03000 23.94000 4.98000 L

H 0 19.24000 23.80000 5.58000 L

H 0 20.84000 23.51000 5.38000 L

N 0 19.71000 23.15000 3.70000 L

H 0 19.57000 22.19000 3.93000 L

H 0 18.89000 23.52000 3.27000 L

H 0 20.48000 23.22000 3.06000 L

C 0 23.41000 27.46000 2.05000 L

O 0 22.56000 28.07000 1.39000 L

N 0 24.39000 28.12000 2.72000 L

C 0 25.55000 27.62000 3.52000 L

H 0 26.34000 27.46000 2.93000 L

H 0 25.31000 26.78000 4.00000 L

C 0 25.78000 28.80000 4.49000 L

H 0 26.71000 28.80000 4.86000 L

H 0 25.12000 28.79000 5.25000 L

C 0 25.53000 30.03000 3.49000 L

H 0 26.33000 30.20000 2.91000 L

H 0 25.29000 30.87000 3.98000 L

C 0 24.33000 29.56000 2.62000 L

H 0 23.40000 29.81000 2.91000 L

C 0 24.55000 30.14000 1.21000 L

O 0 24.28000 31.32000 1.01000 L

N 0 25.11000 29.35000 0.29000 L

H 0 25.39000 28.43000 0.56000 L

C 0 25.32000 29.77000 -1.10000 L

H 0 25.53000 30.75000 -1.06000 L

C 0 26.50000 28.91000 -1.70000 L

H 0 26.32000 27.95000 -1.49000 L

H 0 27.35000 29.19000 -1.27000 L

C 0 26.72000 29.00000 -3.21000 L

H 0 25.88000 28.70000 -3.68000 L

C 0 27.10000 30.47000 -3.59000 L

H 0 27.24000 30.53000 -4.58000 L

H 0 26.35000 31.09000 -3.32000 L

H 0 27.93000 30.73000 -3.11000 L

C 0 27.89000 28.10000 -3.59000 L

H 0 28.04000 28.15000 -4.58000 L

H 0 28.71000 28.41000 -3.11000 L

H 0 27.69000 27.16000 -3.34000 L

C 0 24.04000 29.46000 -1.94000 L

O 0 23.84000 29.97000 -3.02000 L

N 0 23.16000 28.64000 -1.44000 L

H 0 23.18000 28.40000 -0.46000 L

C 0 22.16000 28.07000 -2.29000 L

H 0 22.73000 27.53000 -2.92000 L

C 0 21.14000 27.20000 -1.46000 L

H 0 20.70000 27.78000 -0.77000 L

H 0 21.63000 26.46000 -1.01000 L

C 0 20.05000 26.61000 -2.38000 L

H 0 20.49000 26.03000 -3.07000 L

H 0 19.57000 27.35000 -2.84000 L

C 0 19.04000 25.76000 -1.56000 L

H 0 19.51000 25.06000 -1.03000 L

H 0 18.36000 25.33000 -2.16000 L

N 0 18.35000 26.72000 -0.65000 L

H 0 18.26000 27.68000 -0.93000 L

C 0 17.85000 26.36000 0.53000 L

N 0 17.35000 27.30000 1.40000 L

H 0 17.36000 28.27000 1.15000 L

H 0 16.98000 27.02000 2.28000 L

N 0 17.79000 25.02000 0.81000 L

H 0 18.10000 24.35000 0.14000 L

H 0 17.42000 24.71000 1.69000 L

C 0 21.35000 29.17000 -3.08000 L

O 0 21.25000 29.11000 -4.33000 L

N 0 20.70000 30.11000 -2.40000 L

H 0 20.83000 30.21000 -1.41000 L

C 0 19.78000 30.99000 -3.16000 L

H 0 19.36000 30.41000 -3.85000 L

C 0 18.72000 31.63000 -2.19000 L

H 0 18.27000 32.39000 -2.66000 L

H 0 19.19000 31.96000 -1.37000 L

C 0 17.65000 30.56000 -1.77000 L

O 0 17.87000 29.37000 -2.09000 L

O 0 16.63000 30.91000 -1.17000 L

C 0 20.52000 32.07000 -3.94000 L

O 0 20.06000 32.49000 -4.95000 L

N 0 21.68000 32.48000 -3.46000 L

H 0 21.98000 32.19000 -2.55000 L

C 0 22.51000 33.36000 -4.24000 L

H 0 21.98000 34.20000 -4.37000 L

C 0 23.84000 33.57000 -3.47000 L

H 0 24.28000 32.68000 -3.34000 L

H 0 23.62000 33.97000 -2.57000 L

C 0 24.81000 34.54000 -4.24000 L

H 0 24.92000 34.20000 -5.18000 L

C 0 24.24000 35.99000 -4.34000 L

H 0 24.88000 36.57000 -4.84000 L

H 0 23.36000 35.97000 -4.82000 L

H 0 24.11000 36.35000 -3.41000 L

C 0 26.15000 34.62000 -3.32000 L

H 0 26.82000 35.22000 -3.75000 L

H 0 25.91000 34.99000 -2.42000 L

H 0 26.54000 33.71000 -3.21000 L

C 0 22.82000 32.70000 -5.58000 L

O 0 22.78000 33.33000 -6.63000 L

N 0 23.16000 31.41000 -5.57000 L

H 0 23.26000 30.93000 -4.70000 L

C 0 23.39000 30.66000 -6.86000 L

H 0 24.08000 31.17000 -7.38000 L

C 0 23.82000 29.22000 -6.53000 L

H 0 23.22000 28.80000 -5.85000 L

H 0 24.77000 29.18000 -6.21000 L

O 0 23.73000 28.48000 -7.71000 L

H 0 24.01000 27.53000 -7.52000 L

C 0 22.12000 30.56000 -7.68000 L

O 0 22.10000 30.84000 -8.89000 L

N 0 21.02000 30.20000 -7.03000 L

H 0 21.05000 29.99000 -6.06000 L

C 0 19.75000 30.13000 -7.78000 L

H 0 19.94000 29.43000 -8.47000 L

C 0 18.56000 29.74000 -6.88000 L

H 0 18.46000 30.38000 -6.12000 L

C 0 17.21000 29.59000 -7.73000 L

H 0 16.46000 29.33000 -7.13000 L

H 0 17.00000 30.46000 -8.18000 L

H 0 17.33000 28.87000 -8.43000 L

O 0 18.87000 28.48000 -6.32000 L

H 0 18.12000 28.18000 -5.73000 L

C 0 19.40000 31.42000 -8.43000 L

O 0 18.95000 31.45000 -9.63000 L

N 0 19.63000 32.54000 -7.72000 L

H 0 20.04000 32.49000 -6.81000 L

C 0 19.24000 33.84000 -8.30000 L

H 0 18.30000 33.70000 -8.61000 L

C 0 19.34000 35.01000 -7.24000 L

H 0 19.30000 35.88000 -7.73000 L

H 0 20.21000 34.94000 -6.76000 L

C 0 18.18000 35.00000 -6.18000 L

H 0 18.33000 35.73000 -5.52000 L

H 0 18.17000 34.12000 -5.71000 L

C 0 16.80000 35.21000 -6.85000 L

O 0 16.20000 34.28000 -7.35000 L

N 0 16.29000 36.45000 -6.80000 L

H 0 16.80000 37.19000 -6.35000 L

H 0 15.40000 36.65000 -7.22000 L

C 0 20.17000 34.15000 -9.48000 L

O 0 19.80000 34.86000 -10.43000 L

N 0 21.46000 33.80000 -9.36000 L

H 0 21.79000 33.41000 -8.50000 L

C 0 22.40000 34.00000 -10.50000 L

H 0 22.31000 34.97000 -10.73000 L

C 0 23.87000 33.64000 -10.08000 L

H 0 23.99000 32.66000 -9.92000 L

C 0 24.87000 34.13000 -11.15000 L

H 0 25.80000 33.90000 -10.87000 L

H 0 24.66000 33.69000 -12.02000 L

H 0 24.78000 35.12000 -11.25000 L

O 0 24.18000 34.34000 -8.86000 L

H 0 25.12000 34.12000 -8.58000 L

C 0 21.98000 33.19000 -11.76000 L

O 0 22.05000 33.64000 -12.95000 L

N 0 21.60000 31.98000 -11.53000 L

H 0 21.67000 31.58000 -10.62000 L

C 0 21.03000 31.18000 -12.69000 L

H 0 21.74000 31.14000 -13.39000 L

C 0 20.56000 29.77000 -12.17000 L

H 0 20.00000 29.88000 -11.35000 L

C 0 19.68000 28.97000 -13.27000 L

H 0 19.41000 28.08000 -12.89000 L

H 0 18.86000 29.50000 -13.49000 L

H 0 20.22000 28.83000 -14.09000 L

C 0 21.81000 28.98000 -11.77000 L

H 0 22.46000 29.61000 -11.35000 L

H 0 22.22000 28.60000 -12.60000 L

C 0 21.45000 27.80000 -10.75000 L

H 0 22.28000 27.31000 -10.51000 L

H 0 21.04000 28.19000 -9.93000 L

H 0 20.80000 27.18000 -11.19000 L

C 0 19.85000 31.88000 -13.31000 L

O 0 19.73000 32.00000 -14.57000 L

N 0 18.95000 32.30000 -12.45000 L

H 0 19.09000 32.16000 -11.47000 L

C 0 17.74000 32.99000 -12.91000 L

H 0 17.24000 32.37000 -13.51000 L

C 0 16.90000 33.34000 -11.65000 L

H 0 17.41000 34.00000 -11.10000 L

H 0 16.76000 32.51000 -11.12000 L

C 0 15.51000 33.96000 -12.03000 L

H 0 15.00000 33.30000 -12.59000 L

H 0 15.66000 34.80000 -12.55000 L

C 0 14.66000 34.30000 -10.69000 L

H 0 13.69000 34.43000 -10.92000 L

H 0 15.01000 35.14000 -10.27000 L

N 0 14.69000 33.26000 -9.62000 L

H 0 15.26000 33.44000 -8.82000 L

C 0 14.03000 32.11000 -9.64000 L

N 0 13.27000 31.72000 -10.72000 L

H 0 13.21000 32.32000 -11.52000 L

H 0 12.79000 30.85000 -10.70000 L

N 0 14.05000 31.29000 -8.62000 L

H 0 14.58000 31.53000 -7.81000 L

H 0 13.55000 30.43000 -8.66000 L

C 0 18.07000 34.22000 -13.79000 L

O 0 17.54000 34.41000 -14.89000 L

N 0 18.98000 35.08000 -13.31000 L

H 0 19.36000 34.94000 -12.39000 L

C 0 19.41000 36.22000 -14.10000 L

H 0 18.58000 36.72000 -14.33000 L

C 0 20.42000 37.04000 -13.25000 L

H 0 21.23000 36.48000 -13.08000 L

H 0 19.98000 37.27000 -12.37000 L

C 0 20.86000 38.34000 -13.90000 L

H 0 20.07000 38.75000 -14.36000 L

H 0 21.57000 38.13000 -14.57000 L

C 0 21.44000 39.36000 -12.84000 L

O 0 21.60000 38.96000 -11.60000 L

O 0 21.64000 40.57000 -13.22000 L

C 0 20.10000 35.77000 -15.40000 L

O 0 19.84000 36.38000 -16.46000 L

N 0 21.05000 34.79000 -15.35000 L

H 0 21.25000 34.31000 -14.49000 L

C 0 21.80000 34.45000 -16.61000 L

H 0 22.16000 35.32000 -16.95000 L

C 0 22.98000 33.47000 -16.32000 L

H 0 23.39000 33.20000 -17.19000 L

H 0 22.61000 32.65000 -15.87000 L

C 0 24.10000 34.04000 -15.42000 L

H 0 24.85000 33.38000 -15.50000 L

H 0 23.72000 34.01000 -14.50000 L

S 0 24.62000 35.75000 -15.90000 L

C 0 25.28000 35.29000 -17.56000 L

H 0 25.63000 36.11000 -18.02000 L

H 0 26.02000 34.62000 -17.45000 L

H 0 24.54000 34.89000 -18.11000 L

C 0 20.83000 33.83000 -17.65000 L

O 0 20.99000 34.02000 -18.87000 L

N 0 19.84000 33.07000 -17.17000 L

H 0 19.75000 32.95000 -16.18000 L

C 0 18.90000 32.42000 -18.05000 L

H 0 19.40000 31.78000 -18.63000 L

C 0 17.90000 31.68000 -17.23000 L

H 0 17.39000 32.35000 -16.70000 L

H 0 18.41000 31.08000 -16.61000 L

C 0 16.92000 30.82000 -18.06000 L

C 0 17.30000 29.58000 -18.63000 L

H 0 18.23000 29.23000 -18.51000 L

C 0 16.31000 28.82000 -19.40000 L

H 0 16.55000 27.93000 -19.79000 L

C 0 15.03000 29.36000 -19.59000 L

O 0 14.07000 28.70000 -20.27000 L

H 0 13.24000 29.26000 -20.29000 L

C 0 14.67000 30.56000 -19.09000 L

H 0 13.76000 30.95000 -19.28000 L

C 0 15.62000 31.26000 -18.27000 L

H 0 15.34000 32.12000 -17.84000 L

C 0 18.12000 33.57000 -18.81000 L

O 0 17.92000 33.50000 -20.04000 L

N 0 17.66000 34.56000 -18.06000 L

H 0 17.81000 34.54000 -17.07000 L

C 0 16.91000 35.70000 -18.67000 L

H 0 16.21000 35.26000 -19.23000 L

C 0 16.25000 36.69000 -17.53000 L

H 0 15.76000 37.44000 -17.98000 L

H 0 15.61000 36.16000 -16.97000 L

H 0 16.98000 37.06000 -16.95000 L

C 0 17.83000 36.50000 -19.60000 L

O 0 17.45000 36.82000 -20.73000 L

N 0 19.05000 36.80000 -19.21000 L

H 0 19.39000 36.49000 -18.32000 L

C 0 19.89000 37.59000 -20.08000 L

H 0 19.32000 38.39000 -20.25000 L

C 0 21.22000 37.90000 -19.41000 L

H 0 21.85000 38.27000 -20.09000 L

H 0 21.60000 37.05000 -19.03000 L

C 0 21.08000 38.92000 -18.27000 L

H 0 20.28000 38.65000 -17.73000 L

C 0 22.31000 38.92000 -17.34000 L

H 0 22.18000 39.60000 -16.61000 L

H 0 22.42000 38.02000 -16.93000 L

H 0 23.13000 39.16000 -17.87000 L

C 0 20.96000 40.37000 -18.89000 L

H 0 20.87000 41.05000 -18.15000 L

H 0 21.78000 40.58000 -19.43000 L

H 0 20.15000 40.42000 -19.48000 L

C 0 20.27000 36.81000 -21.36000 L

O 0 20.69000 37.43000 -22.33000 L

N 0 20.22000 35.48000 -21.34000 L

H 0 19.97000 34.99000 -20.50000 L

C 0 20.56000 34.73000 -22.58000 L

H 0 20.98000 35.40000 -23.19000 L

C 0 21.51000 33.49000 -22.21000 L

H 0 21.64000 32.85000 -22.98000 L

C 0 22.87000 33.97000 -21.73000 L

H 0 23.44000 33.17000 -21.50000 L

H 0 23.31000 34.50000 -22.45000 L

H 0 22.76000 34.53000 -20.91000 L

O 0 20.90000 32.68000 -21.22000 L

H 0 21.50000 31.91000 -21.00000 L

C 0 19.29000 34.19000 -23.25000 L

O 0 19.32000 33.29000 -24.12000 L

N 0 18.16000 34.70000 -22.81000 L

H 0 18.17000 35.35000 -22.05000 L

C 0 16.88000 34.33000 -23.42000 L

H 0 16.23000 34.78000 -22.81000 L

C 0 16.78000 34.80000 -24.88000 L

H 0 15.95000 34.41000 -25.28000 L

H 0 17.58000 34.47000 -25.38000 L

C 0 16.72000 36.33000 -25.01000 L

H 0 16.01000 36.70000 -24.41000 L

H 0 16.52000 36.59000 -25.96000 L

C 0 17.96000 36.99000 -24.64000 L

O 0 19.06000 36.64000 -25.14000 L

N 0 17.86000 37.98000 -23.77000 L

H 0 16.96000 38.23000 -23.41000 L

H 0 18.67000 38.47000 -23.47000 L

C 0 16.58000 32.90000 -23.37000 L

O 0 15.81000 32.40000 -24.22000 L

N 0 17.05000 32.18000 -22.34000 L

H 0 17.58000 32.61000 -21.61000 L

C 0 16.77000 30.74000 -22.33000 L

H 0 15.78000 30.62000 -22.42000 L

H 0 17.06000 30.39000 -21.43000 L

C 0 17.46000 29.93000 -23.41000 L

O 0 17.21000 28.75000 -23.52000 L

N 0 18.34000 30.51000 -24.20000 L

H 0 18.62000 31.46000 -24.03000 L

C 0 18.91000 29.76000 -25.31000 L

H 0 18.22000 29.06000 -25.48000 L

C 0 19.12000 30.67000 -26.50000 L

H 0 19.58000 30.16000 -27.22000 L

H 0 19.69000 31.44000 -26.21000 L

C 0 17.73000 31.20000 -27.00000 L

H 0 17.20000 31.55000 -26.23000 L

H 0 17.23000 30.46000 -27.45000 L

C 0 17.97000 32.33000 -28.02000 L

H 0 18.41000 31.97000 -28.84000 L

H 0 18.53000 33.05000 -27.62000 L

N 0 16.66000 32.89000 -28.39000 L

H 0 15.86000 32.29000 -28.31000 L

C 0 16.45000 34.14000 -28.81000 L

N 0 17.47000 34.97000 -28.96000 L

H 0 18.41000 34.67000 -28.75000 L

H 0 17.31000 35.91000 -29.28000 L

N 0 15.22000 34.55000 -29.07000 L

H 0 14.45000 33.92000 -28.94000 L

H 0 15.06000 35.48000 -29.39000 L

C 0 20.28000 29.13000 -24.99000 L

O 0 20.84000 28.50000 -25.85000 L

N 0 20.83000 29.36000 -23.80000 L

H 0 20.32000 29.87000 -23.11000 L

C 0 22.19000 28.85000 -23.49000 L

H 0 22.59000 28.41000 -24.29000 L

C 0 23.09000 30.03000 -23.13000 L

H 0 22.60000 30.54000 -22.42000 L

C 0 24.41000 29.60000 -22.53000 L

H 0 24.96000 30.40000 -22.31000 L

H 0 24.23000 29.08000 -21.69000 L

H 0 24.90000 29.02000 -23.18000 L

C 0 23.39000 30.94000 -24.36000 L

H 0 23.98000 31.70000 -24.09000 L

H 0 23.84000 30.40000 -25.07000 L

H 0 22.53000 31.30000 -24.72000 L

C 0 22.03000 27.86000 -22.29000 L

O 0 21.59000 28.27000 -21.19000 L

N 0 22.32000 26.56000 -22.49000 L

C 0 22.69000 25.91000 -23.79000 L

H 0 23.66000 26.05000 -23.99000 L

H 0 22.14000 26.27000 -24.54000 L

C 0 22.39000 24.46000 -23.53000 L

H 0 22.97000 23.89000 -24.10000 L

H 0 21.43000 24.27000 -23.73000 L

C 0 22.73000 24.28000 -21.93000 L

H 0 23.71000 24.22000 -21.75000 L

H 0 22.27000 23.50000 -21.52000 L

C 0 22.14000 25.63000 -21.38000 L

H 0 21.17000 25.61000 -21.13000 L

C 0 22.90000 26.06000 -20.10000 L

O 0 24.01000 26.51000 -20.17000 L

N 0 22.27000 25.91000 -18.91000 L

H 0 21.33000 25.58000 -18.89000 L

C 0 22.98000 26.22000 -17.68000 L

H 0 23.89000 26.48000 -17.98000 L

C 0 22.19000 27.36000 -16.87000 L

H 0 21.21000 27.16000 -16.84000 L

C 0 22.70000 27.49000 -15.35000 L

H 0 22.19000 28.21000 -14.88000 L

H 0 22.57000 26.62000 -14.87000 L

H 0 23.68000 27.73000 -15.35000 L

C 0 22.39000 28.66000 -17.63000 L

H 0 22.36000 28.46000 -18.61000 L

H 0 23.28000 29.03000 -17.39000 L

C 0 21.29000 29.74000 -17.31000 L

H 0 21.47000 30.57000 -17.83000 L

H 0 20.39000 29.38000 -17.55000 L

H 0 21.31000 29.95000 -16.33000 L

C 0 23.02000 24.98000 -16.79000 L

O 0 22.02000 24.22000 -16.65000 L

N 0 24.19000 24.78000 -16.18000 L

H 0 24.98000 25.34000 -16.40000 L

C 0 24.26000 23.72000 -15.18000 L

H 0 23.49000 23.10000 -15.30000 L

C 0 25.65000 22.95000 -15.29000 L

H 0 26.45000 23.55000 -15.21000 L

C 0 25.75000 21.91000 -14.11000 L

H 0 26.61000 21.41000 -14.17000 L

H 0 25.70000 22.39000 -13.24000 L

H 0 24.99000 21.26000 -14.17000 L

C 0 25.72000 22.29000 -16.72000 L

H 0 25.79000 23.02000 -17.40000 L

H 0 24.87000 21.78000 -16.87000 L

C 0 26.99000 21.29000 -16.87000 L

H 0 27.00000 20.90000 -17.79000 L

H 0 27.83000 21.80000 -16.72000 L

H 0 26.92000 20.56000 -16.19000 L

C 0 24.27000 24.51000 -13.86000 L

O 0 25.15000 25.37000 -13.63000 L

N 0 23.28000 24.18000 -13.01000 L

H 0 22.71000 23.39000 -13.20000 L

C 0 23.05000 24.99000 -11.81000 L

H 0 22.07000 25.04000 -11.63000 L

H 0 23.41000 25.91000 -11.95000 L

C 0 23.76000 24.31000 -10.65000 L

O 0 23.59000 23.08000 -10.45000 L

N 0 24.51000 25.08000 -9.82000 L

H 0 24.56000 26.08000 -9.92000 L

C 0 25.22000 24.36000 -8.76000 L

H 0 24.67000 23.54000 -8.62000 L

C 0 26.75000 24.15000 -9.11000 L

H 0 27.29000 24.99000 -9.20000 L

C 0 27.58000 23.20000 -8.15000 L

H 0 28.53000 23.14000 -8.46000 L

H 0 27.56000 23.56000 -7.22000 L

H 0 27.17000 22.28000 -8.15000 L

C 0 27.23000 24.52000 -10.37000 L

H 0 28.21000 24.32000 -10.43000 L

H 0 26.74000 24.00000 -11.07000 L

H 0 27.07000 25.50000 -10.51000 L

C 0 25.42000 25.36000 -7.62000 L

O 0 25.59000 26.57000 -7.85000 L

N 0 25.24000 24.87000 -6.38000 L

H 0 24.96000 23.93000 -6.25000 L

C 0 25.47000 25.75000 -5.22000 L

H 0 25.42000 26.71000 -5.51000 L

H 0 26.38000 25.56000 -4.84000 L

C 0 24.38000 25.44000 -4.21000 L

O 0 23.23000 25.78000 -4.44000 L

N 0 24.74000 24.67000 -3.16000 L

H 0 25.66000 24.28000 -3.13000 L

C 0 23.82000 24.40000 -2.03000 L

H 0 23.40000 25.27000 -1.75000 L

H 0 24.36000 24.03000 -1.27000 L

C 0 22.72000 23.42000 -2.34000 L

O 0 21.67000 23.49000 -1.67000 L

N 0 22.90000 22.50000 -3.29000 L

H 0 23.77000 22.44000 -3.79000 L

C 0 21.80000 21.59000 -3.58000 L

H 0 20.95000 22.10000 -3.42000 L

C 0 21.84000 21.05000 -5.08000 L

H 0 22.76000 20.67000 -5.25000 L

C 0 20.84000 19.82000 -5.32000 L

H 0 20.91000 19.52000 -6.28000 L

H 0 21.08000 19.07000 -4.72000 L

H 0 19.90000 20.12000 -5.13000 L

C 0 21.54000 22.29000 -6.08000 L

H 0 21.57000 21.97000 -7.02000 L

H 0 20.64000 22.67000 -5.88000 L

H 0 22.24000 23.00000 -5.94000 L

C 0 21.92000 20.44000 -2.63000 L

O 0 22.98000 19.72000 -2.60000 L

N 0 20.87000 20.21000 -1.85000 L

H 0 20.05000 20.78000 -1.90000 L

C 0 20.96000 19.11000 -0.95000 L

H 0 21.70000 18.54000 -1.30000 L

C 0 21.26000 19.66000 0.45000 L

H 0 20.50000 20.19000 0.81000 L

H 0 22.09000 20.23000 0.45000 L

O 0 21.48000 18.57000 1.29000 L

H 0 21.68000 18.90000 2.21000 L

C 0 19.63000 18.30000 -0.93000 L

O 0 19.49000 17.30000 -0.25000 L

N 0 18.68000 18.68000 -1.80000 L

H 0 18.86000 19.42000 -2.44000 L

C 0 17.40000 18.00000 -1.79000 L

H 0 17.62000 17.04000 -1.62000 L

C 0 16.46000 18.64000 -0.69000 L

H 0 16.94000 18.75000 0.18000 L

H 0 15.64000 18.08000 -0.55000 L

O 0 16.04000 19.97000 -1.17000 L

H 0 15.44000 20.39000 -0.49000 L

C 0 16.70000 18.17000 -3.15000 L

O 0 17.16000 19.00000 -4.01000 L

N 0 15.57000 17.47000 -3.31000 L

H 0 15.22000 16.91000 -2.56000 L

C 0 14.86000 17.55000 -4.56000 L

H 0 14.10000 16.91000 -4.52000 L

H 0 15.49000 17.28000 -5.29000 L

C 0 14.36000 18.97000 -4.78000 L

O 0 14.34000 19.48000 -5.92000 L

N 0 13.85000 19.58000 -3.72000 L

H 0 13.83000 19.12000 -2.83000 L

C 0 13.32000 20.95000 -3.88000 L

H 0 12.59000 20.91000 -4.56000 L

C 0 12.83000 21.46000 -2.50000 L

H 0 13.55000 21.32000 -1.81000 L

H 0 12.01000 20.97000 -2.22000 L

C 0 12.52000 22.98000 -2.64000 L

H 0 11.91000 23.11000 -3.42000 L

H 0 13.37000 23.47000 -2.79000 L

C 0 11.83000 23.52000 -1.34000 L

O 0 10.75000 23.03000 -0.92000 L

N 0 12.42000 24.59000 -0.77000 L

H 0 13.24000 24.99000 -1.17000 L

H 0 12.03000 24.99000 0.07000 L

C 0 14.46000 21.86000 -4.28000 L

O 0 14.30000 22.73000 -5.16000 L

N 0 15.65000 21.70000 -3.67000 L

H 0 15.78000 21.01000 -2.96000 L

C 0 16.77000 22.58000 -4.08000 L

H 0 16.45000 23.50000 -3.83000 L

C 0 18.09000 22.21000 -3.32000 L

H 0 18.83000 22.80000 -3.64000 L

H 0 18.33000 21.26000 -3.51000 L

C 0 17.94000 22.40000 -1.79000 L

O 0 17.01000 23.16000 -1.35000 L

O 0 18.78000 21.86000 -1.09000 L

C 0 17.04000 22.44000 -5.60000 L

O 0 17.30000 23.42000 -6.34000 L

N 0 17.07000 21.20000 -6.07000 L

H 0 16.92000 20.43000 -5.44000 L

C 0 17.32000 20.94000 -7.51000 L

H 0 18.15000 21.46000 -7.72000 L

C 0 17.42000 19.36000 -7.77000 L

H 0 17.59000 19.20000 -8.74000 L

H 0 18.17000 18.98000 -7.23000 L

H 0 16.56000 18.93000 -7.50000 L

C 0 16.28000 21.51000 -8.37000 L

O 0 16.59000 22.17000 -9.37000 L

N 0 15.01000 21.32000 -7.99000 L

H 0 14.80000 20.83000 -7.15000 L

C 0 13.92000 21.83000 -8.84000 L

H 0 14.12000 21.54000 -9.77000 L

C 0 12.57000 21.28000 -8.35000 L

H 0 12.43000 21.62000 -7.42000 L

H 0 12.64000 20.29000 -8.34000 L

C 0 11.33000 21.64000 -9.16000 L

H 0 11.23000 22.63000 -9.07000 L

C 0 11.58000 21.19000 -10.69000 L

H 0 10.78000 21.41000 -11.24000 L

H 0 12.38000 21.67000 -11.05000 L

H 0 11.75000 20.21000 -10.72000 L

C 0 9.95000 21.03000 -8.58000 L

H 0 9.19000 21.31000 -9.16000 L

H 0 10.01000 20.03000 -8.56000 L

H 0 9.81000 21.37000 -7.65000 L

C 0 13.87000 23.31000 -8.84000 L

O 0 13.55000 23.93000 -9.86000 L

N 0 14.27000 23.97000 -7.74000 L

H 0 14.57000 23.47000 -6.92000 L

C 0 14.27000 25.42000 -7.80000 L

H 0 13.36000 25.66000 -8.11000 L

C 0 14.55000 26.01000 -6.38000 L

H 0 14.84000 26.96000 -6.48000 L

H 0 15.28000 25.48000 -5.95000 L

C 0 13.27000 25.96000 -5.48000 L

H 0 13.03000 25.00000 -5.29000 L

H 0 12.51000 26.40000 -5.96000 L

C 0 13.49000 26.68000 -4.14000 L

O 0 14.64000 27.11000 -3.85000 L

O 0 12.54000 26.75000 -3.36000 L

C 0 15.23000 25.95000 -8.85000 L

O 0 14.98000 26.95000 -9.55000 L

N 0 16.43000 25.34000 -8.92000 L

H 0 16.64000 24.59000 -8.30000 L

C 0 17.39000 25.78000 -9.90000 L

H 0 17.45000 26.77000 -9.82000 L

C 0 18.73000 25.05000 -9.63000 L

H 0 19.28000 25.07000 -10.47000 L

H 0 18.54000 24.10000 -9.38000 L

C 0 19.51000 25.79000 -8.44000 L

H 0 18.94000 25.82000 -7.61000 L

H 0 19.76000 26.72000 -8.71000 L

C 0 20.76000 25.04000 -8.12000 L

H 0 21.23000 24.79000 -8.97000 L

H 0 20.54000 24.21000 -7.61000 L

C 0 21.65000 25.91000 -7.30000 L

H 0 21.96000 26.66000 -7.88000 L

H 0 22.44000 25.36000 -7.01000 L

N 0 21.07000 26.51000 -6.07000 L

H 0 21.76000 27.07000 -5.62000 L

H 0 20.29000 27.09000 -6.32000 L

H 0 20.77000 25.79000 -5.46000 L

C 0 16.91000 25.39000 -11.27000 L

O 0 17.13000 26.13000 -12.22000 L

N 0 16.39000 24.19000 -11.44000 L

H 0 16.37000 23.52000 -10.69000 L

C 0 15.80000 23.86000 -12.80000 L

H 0 16.53000 23.96000 -13.48000 L

C 0 15.29000 22.42000 -12.76000 L

H 0 14.72000 22.27000 -11.96000 L

C 0 14.44000 22.15000 -14.04000 L

H 0 14.10000 21.21000 -14.02000 L

H 0 13.66000 22.78000 -14.06000 L

H 0 15.00000 22.29000 -14.85000 L

C 0 16.53000 21.47000 -12.72000 L

H 0 17.22000 21.87000 -12.12000 L

H 0 16.90000 21.38000 -13.64000 L

C 0 16.16000 20.04000 -12.18000 L

H 0 16.98000 19.46000 -12.17000 L

H 0 15.79000 20.11000 -11.26000 L

H 0 15.47000 19.63000 -12.78000 L

C 0 14.69000 24.84000 -13.22000 L

O 0 14.73000 25.39000 -14.35000 L

N 0 13.71000 25.12000 -12.33000 L

H 0 13.66000 24.64000 -11.45000 L

C 0 12.69000 26.15000 -12.70000 L

H 0 12.33000 25.83000 -13.58000 L

C 0 11.63000 26.26000 -11.57000 L

H 0 11.03000 27.03000 -11.76000 L

H 0 12.11000 26.41000 -10.70000 L

C 0 10.75000 24.91000 -11.47000 L

H 0 11.35000 24.13000 -11.30000 L

H 0 10.26000 24.77000 -12.33000 L

C 0 9.71000 25.09000 -10.26000 L

H 0 10.15000 25.29000 -9.39000 L

H 0 9.11000 24.29000 -10.15000 L

N 0 8.88000 26.18000 -10.59000 L

H 0 9.32000 27.08000 -10.63000 L

C 0 7.56000 26.12000 -10.84000 L

N 0 6.83000 24.99000 -10.58000 L

H 0 7.28000 24.19000 -10.18000 L

H 0 5.85000 24.97000 -10.78000 L

N 0 6.96000 27.23000 -11.27000 L

H 0 7.48000 28.07000 -11.38000 L

H 0 5.98000 27.22000 -11.47000 L

C 0 13.25000 27.56000 -12.96000 L

O 0 12.71000 28.34000 -13.73000 L

N 0 14.39000 27.86000 -12.32000 L

H 0 14.80000 27.20000 -11.69000 L

C 0 15.02000 29.14000 -12.55000 L

H 0 14.25000 29.78000 -12.59000 L

C 0 16.05000 29.38000 -11.40000 L

H 0 16.50000 30.27000 -11.54000 L

H 0 15.58000 29.38000 -10.52000 L

H 0 16.74000 28.66000 -11.41000 L

C 0 15.81000 29.19000 -13.86000 L

O 0 16.22000 30.31000 -14.25000 L

N 0 16.09000 28.02000 -14.47000 L

H 0 15.88000 27.15000 -14.02000 L

C 0 16.71000 28.04000 -15.82000 L

H 0 17.13000 28.94000 -15.97000 L

H 0 16.00000 27.88000 -16.51000 L

C 0 17.78000 26.97000 -15.95000 L

O 0 18.28000 26.77000 -17.05000 L

N 0 18.01000 26.16000 -14.94000 L

H 0 17.52000 26.24000 -14.07000 L

C 0 19.06000 25.10000 -15.14000 L

H 0 19.72000 25.52000 -15.77000 L

C 0 19.73000 24.69000 -13.79000 L

H 0 20.42000 23.99000 -13.96000 L

H 0 20.16000 25.49000 -13.38000 L

H 0 19.03000 24.33000 -13.17000 L

C 0 18.58000 23.88000 -15.86000 L

O 0 17.50000 23.41000 -15.53000 L

N 0 19.34000 23.39000 -16.87000 L

H 0 20.14000 23.91000 -17.18000 L

C 0 19.05000 22.15000 -17.52000 L

H 0 18.05000 22.17000 -17.48000 L

C 0 19.58000 22.13000 -18.98000 L

H 0 19.37000 21.26000 -19.42000 L

H 0 20.57000 22.28000 -19.00000 L

O 0 18.98000 23.16000 -19.74000 L

H 0 19.33000 23.13000 -20.67000 L

C 0 19.55000 20.92000 -16.73000 L

O 0 19.00000 19.82000 -16.91000 L

N 0 20.54000 21.12000 -15.87000 L

H 0 20.83000 22.07000 -15.72000 L

C 0 21.23000 20.07000 -15.13000 L

H 0 20.59000 19.30000 -15.06000 L

C 0 22.57000 19.62000 -15.83000 L

H 0 23.01000 18.91000 -15.27000 L

H 0 23.18000 20.41000 -15.90000 L

C 0 22.33000 19.06000 -17.24000 L

H 0 21.76000 19.66000 -17.80000 L

C 0 23.78000 18.84000 -17.86000 L

H 0 23.70000 18.47000 -18.79000 L

H 0 24.27000 19.71000 -17.90000 L

H 0 24.29000 18.19000 -17.30000 L

C 0 21.59000 17.73000 -17.10000 L

H 0 21.42000 17.34000 -18.00000 L

H 0 22.15000 17.09000 -16.56000 L

H 0 20.72000 17.88000 -16.63000 L

C 0 21.66000 20.78000 -13.83000 L

O 0 21.77000 22.02000 -13.80000 L

N 0 21.85000 19.98000 -12.79000 L

H 0 21.63000 19.01000 -12.83000 L

C 0 22.41000 20.57000 -11.56000 L

H 0 22.68000 21.49000 -11.83000 L

C 0 21.36000 20.65000 -10.39000 L

H 0 21.80000 21.10000 -9.61000 L

C 0 20.11000 21.48000 -10.84000 L

H 0 19.45000 21.52000 -10.09000 L

H 0 20.40000 22.41000 -11.08000 L

H 0 19.69000 21.04000 -11.63000 L

C 0 20.95000 19.24000 -9.88000 L

H 0 20.28000 19.34000 -9.14000 L

H 0 20.54000 18.73000 -10.63000 L

H 0 21.76000 18.76000 -9.55000 L

C 0 23.59000 19.70000 -11.08000 L

O 0 23.79000 18.57000 -11.59000 L

N 0 24.41000 20.26000 -10.19000 L

H 0 24.31000 21.22000 -9.97000 L

C 0 25.45000 19.47000 -9.54000 L

H 0 25.26000 18.51000 -9.75000 L

C 0 26.86000 19.95000 -10.04000 L

H 0 27.57000 19.42000 -9.58000 L

H 0 26.97000 20.92000 -9.81000 L

C 0 27.01000 19.77000 -11.60000 L

H 0 26.27000 20.25000 -12.07000 L

H 0 26.98000 18.80000 -11.83000 L

C 0 28.37000 20.37000 -12.05000 L

O 0 28.72000 21.50000 -11.66000 L

N 0 29.06000 19.66000 -12.95000 L

H 0 28.70000 18.80000 -13.30000 L

H 0 29.94000 20.00000 -13.27000 L

C 0 25.40000 19.74000 -8.04000 L

O 0 24.85000 20.77000 -7.57000 L

N 0 26.09000 18.87000 -7.28000 L

H 0 26.51000 18.06000 -7.69000 L

C 0 26.24000 19.15000 -5.87000 L

H 0 26.10000 20.13000 -5.73000 L

C 0 25.15000 18.46000 -5.05000 L

H 0 24.28000 18.88000 -5.31000 L

H 0 25.35000 18.67000 -4.09000 L

C 0 24.99000 16.91000 -5.18000 L

H 0 25.21000 16.64000 -6.11000 L

C 0 25.99000 16.18000 -4.26000 L

H 0 25.86000 15.19000 -4.36000 L

H 0 26.92000 16.43000 -4.51000 L

H 0 25.82000 16.44000 -3.31000 L

C 0 23.51000 16.50000 -4.88000 L

H 0 23.42000 15.51000 -4.96000 L

H 0 23.27000 16.78000 -3.94000 L

H 0 22.90000 16.95000 -5.52000 L

C 0 27.67000 18.61000 -5.53000 L H 301

O 0 28.22000 17.72000 -6.21000 L

N 0 29.04000 19.86000 -1.76000 L H 320

H 0 28.93000 20.66000 -2.35000 L

C 0 29.08000 20.03000 -0.27000 L

H 0 29.97000 19.68000 0.01000 L

C 0 28.89000 21.56000 0.07000 L

H 0 28.06000 21.92000 -0.34000 L

C 0 28.90000 21.81000 1.62000 L

H 0 28.78000 22.78000 1.80000 L

H 0 28.15000 21.29000 2.05000 L

H 0 29.77000 21.50000 2.00000 L

O 0 29.99000 22.27000 -0.47000 L

H 0 29.90000 23.24000 -0.27000 L

C 0 28.06000 19.14000 0.49000 L

O 0 28.39000 18.57000 1.54000 L

N 0 26.85000 18.97000 -0.06000 L

H 0 26.62000 19.44000 -0.91000 L

C 0 25.87000 18.09000 0.59000 L

H 0 25.67000 18.53000 1.46000 L

C 0 24.63000 17.96000 -0.33000 L

H 0 23.95000 17.37000 0.10000 L

H 0 24.23000 18.87000 -0.49000 L

H 0 24.91000 17.57000 -1.21000 L

C 0 26.41000 16.69000 0.83000 L H 322

O 0 26.08000 16.03000 1.84000 L

N 0 28.18000 16.24000 4.05000 L H 353

H 0 27.74000 16.54000 3.20000 L

C 0 27.42000 16.17000 5.29000 L

H 0 28.12000 16.11000 5.99000 L

C 0 26.48000 17.42000 5.41000 L

H 0 25.81000 17.27000 6.13000 L

H 0 26.01000 17.57000 4.54000 L

C 0 27.27000 18.67000 5.75000 L

C 0 27.55000 19.63000 4.80000 L

H 0 27.18000 19.53000 3.87000 L

C 0 28.36000 20.78000 5.13000 L

H 0 28.52000 21.49000 4.45000 L

C 0 28.93000 20.90000 6.41000 L

H 0 29.52000 21.68000 6.63000 L

C 0 28.66000 19.92000 7.38000 L

H 0 29.02000 20.01000 8.31000 L

C 0 27.88000 18.78000 7.02000 L

H 0 27.75000 18.04000 7.68000 L

C 0 26.54000 14.92000 5.34000 L

O 0 26.36000 14.38000 6.44000 L

N 0 25.96000 14.49000 4.20000 L

H 0 26.23000 14.88000 3.33000 L

C 0 24.92000 13.44000 4.24000 L

H 0 24.69000 13.31000 5.20000 L

C 0 23.67000 13.92000 3.45000 L

H 0 22.93000 13.26000 3.57000 L

H 0 23.90000 13.98000 2.47000 L

C 0 23.22000 15.30000 3.95000 L

C 0 22.99000 16.42000 3.20000 L

H 0 23.07000 16.46000 2.21000 L

N 0 22.64000 17.48000 4.01000 L

H 0 22.36000 18.39000 3.70000 L

C 0 22.72000 17.09000 5.32000 L

C 0 22.46000 17.83000 6.52000 L

H 0 22.19000 18.79000 6.48000 L

C 0 22.60000 17.18000 7.73000 L

H 0 22.43000 17.68000 8.58000 L

C 0 22.99000 15.82000 7.78000 L

H 0 23.09000 15.37000 8.66000 L

C 0 23.24000 15.07000 6.57000 L

H 0 23.52000 14.11000 6.61000 L

C 0 23.08000 15.72000 5.33000 L

C 0 25.46000 12.11000 3.68000 L H 355

O 0 24.81000 11.06000 3.82000 L

N 0 24.10000 8.57000 0.29000 L H 374

C 0 24.53000 8.20000 1.66000 L

H 0 24.72000 9.02000 2.20000 L

H 0 25.34000 7.62000 1.62000 L

C 0 23.29000 7.44000 2.20000 L

H 0 23.22000 7.51000 3.19000 L

H 0 23.31000 6.47000 1.93000 L

C 0 22.17000 8.18000 1.50000 L

H 0 21.98000 9.05000 1.96000 L

H 0 21.34000 7.63000 1.48000 L

C 0 22.67000 8.43000 0.09000 L

H 0 22.50000 7.71000 -0.58000 L

C 0 22.04000 9.75000 -0.37000 L

O 0 20.89000 9.77000 -0.79000 L

N 0 22.71000 10.88000 -0.22000 L

H 0 23.63000 10.89000 0.16000 L

C 0 22.03000 12.12000 -0.66000 L

H 0 21.15000 12.09000 -0.18000 L

C 0 22.86000 13.38000 -0.27000 L

H 0 23.26000 13.21000 0.63000 L

C 0 24.04000 13.63000 -1.18000 L

H 0 24.53000 14.46000 -0.88000 L

H 0 24.66000 12.85000 -1.14000 L

H 0 23.72000 13.76000 -2.12000 L

C 0 21.91000 14.66000 -0.26000 L

H 0 22.45000 15.47000 -0.02000 L

H 0 21.52000 14.78000 -1.18000 L

H 0 21.18000 14.53000 0.40000 L

C 0 21.76000 12.12000 -2.15000 L

O 0 20.86000 12.81000 -2.64000 L

N 0 22.52000 11.35000 -2.94000 L

H 0 23.23000 10.76000 -2.54000 L

C 0 22.30000 11.40000 -4.39000 L

H 0 22.36000 12.37000 -4.63000 L

C 0 23.40000 10.66000 -5.17000 L

H 0 23.39000 9.68000 -4.95000 L

C 0 23.15000 10.80000 -6.67000 L

H 0 23.87000 10.32000 -7.18000 L

H 0 22.26000 10.40000 -6.90000 L

H 0 23.15000 11.77000 -6.92000 L

C 0 24.77000 11.26000 -4.79000 L

H 0 25.50000 10.79000 -5.30000 L

H 0 24.78000 12.24000 -5.03000 L

H 0 24.92000 11.15000 -3.81000 L

C 0 20.90000 10.83000 -4.75000 L

O 0 20.11000 11.45000 -5.50000 L

N 0 20.60000 9.64000 -4.26000 L

H 0 21.26000 9.18000 -3.66000 L

C 0 19.28000 8.97000 -4.59000 L

H 0 19.26000 8.04000 -4.20000 L

H 0 19.16000 8.92000 -5.58000 L

C 0 18.20000 9.84000 -3.95000 L

O 0 17.07000 9.93000 -4.48000 L

N 0 18.52000 10.45000 -2.79000 L

H 0 19.43000 10.35000 -2.40000 L

C 0 17.50000 11.27000 -2.11000 L

H 0 16.73000 10.66000 -1.96000 L

C 0 18.02000 11.82000 -0.76000 L

H 0 18.81000 12.41000 -0.94000 L

H 0 18.29000 11.05000 -0.19000 L

C 0 16.91000 12.65000 -0.02000 L

H 0 16.23000 12.02000 0.36000 L

H 0 16.47000 13.26000 -0.67000 L

C 0 17.50000 13.49000 1.13000 L

H 0 18.28000 13.03000 1.54000 L

H 0 16.80000 13.65000 1.83000 L

C 0 17.92000 14.79000 0.54000 L

H 0 17.30000 15.05000 -0.20000 L

H 0 18.85000 14.72000 0.18000 L

N 0 17.88000 15.80000 1.59000 L

H 0 18.16000 16.68000 1.21000 L

H 0 16.95000 15.87000 1.94000 L

H 0 18.50000 15.54000 2.33000 L

C 0 17.08000 12.43000 -2.99000 L

O 0 15.90000 12.67000 -3.23000 L

N 0 18.08000 13.14000 -3.54000 L

H 0 19.03000 12.88000 -3.38000 L

C 0 17.77000 14.28000 -4.37000 L

H 0 17.21000 14.90000 -3.81000 L

C 0 19.08000 15.02000 -4.80000 L

H 0 19.74000 14.38000 -5.20000 L

C 0 18.82000 16.05000 -5.92000 L

H 0 19.68000 16.50000 -6.17000 L

H 0 18.44000 15.59000 -6.72000 L

H 0 18.17000 16.74000 -5.60000 L

C 0 19.71000 15.64000 -3.54000 L

H 0 20.55000 16.12000 -3.79000 L

H 0 19.06000 16.29000 -3.14000 L

H 0 19.92000 14.92000 -2.88000 L

C 0 16.96000 13.78000 -5.56000 L

O 0 15.97000 14.43000 -5.96000 L

N 0 17.38000 12.68000 -6.19000 L

H 0 18.17000 12.17000 -5.86000 L

C 0 16.64000 12.24000 -7.41000 L

H 0 16.61000 13.07000 -7.96000 L

C 0 17.38000 11.04000 -8.11000 L

H 0 16.84000 10.74000 -8.89000 L

H 0 17.46000 10.28000 -7.46000 L

C 0 18.85000 11.52000 -8.59000 L

H 0 19.45000 11.66000 -7.80000 L

H 0 18.78000 12.37000 -9.11000 L

C 0 19.51000 10.44000 -9.51000 L

H 0 20.36000 10.80000 -9.91000 L

H 0 18.88000 10.19000 -10.25000 L

C 0 19.84000 9.20000 -8.69000 L

H 0 18.98000 8.82000 -8.34000 L

H 0 20.41000 9.48000 -7.92000 L

N 0 20.58000 8.12000 -9.54000 L

H 0 20.77000 7.33000 -8.97000 L

H 0 20.00000 7.85000 -10.31000 L

H 0 21.43000 8.51000 -9.89000 L

C 0 15.20000 11.79000 -7.11000 L

O 0 14.26000 12.07000 -7.89000 L

N 0 15.03000 11.00000 -6.05000 L

H 0 15.82000 10.72000 -5.50000 L

C 0 13.65000 10.56000 -5.68000 L

H 0 13.28000 10.05000 -6.46000 L

C 0 13.72000 9.70000 -4.42000 L

H 0 14.21000 10.23000 -3.72000 L

H 0 14.23000 8.87000 -4.63000 L

C 0 12.39000 9.28000 -3.81000 L

H 0 11.90000 8.75000 -4.50000 L

H 0 11.87000 10.11000 -3.60000 L

C 0 12.63000 8.41000 -2.49000 L

H 0 13.41000 7.80000 -2.61000 L

H 0 11.81000 7.88000 -2.27000 L

N 0 12.92000 9.24000 -1.30000 L

H 0 12.20000 9.85000 -0.96000 L

C 0 14.08000 9.24000 -0.64000 L

N 0 15.10000 8.45000 -1.04000 L

H 0 14.99000 7.85000 -1.84000 L

H 0 15.96000 8.45000 -0.54000 L

N 0 14.23000 10.03000 0.42000 L

H 0 13.48000 10.62000 0.72000 L

H 0 15.10000 10.03000 0.92000 L

C 0 12.76000 11.78000 -5.38000 L

O 0 11.58000 11.82000 -5.75000 L

N 0 13.26000 12.70000 -4.58000 L

H 0 14.19000 12.60000 -4.22000 L

C 0 12.44000 13.90000 -4.20000 L

H 0 11.57000 13.56000 -3.85000 L

C 0 13.22000 14.77000 -3.16000 L

H 0 12.81000 15.68000 -3.11000 L

H 0 14.18000 14.86000 -3.44000 L

C 0 13.21000 14.18000 -1.76000 L

H 0 13.50000 13.22000 -1.82000 L

H 0 12.27000 14.23000 -1.41000 L

C 0 14.11000 14.88000 -0.78000 L

O 0 14.89000 15.72000 -1.19000 L

O 0 14.09000 14.55000 0.42000 L

C 0 12.19000 14.74000 -5.42000 L

O 0 11.09000 15.29000 -5.60000 L

N 0 13.18000 14.83000 -6.32000 L

H 0 14.05000 14.36000 -6.18000 L

C 0 12.96000 15.63000 -7.52000 L

H 0 12.68000 16.54000 -7.19000 L

C 0 14.24000 15.64000 -8.34000 L

H 0 14.54000 14.69000 -8.45000 L

H 0 14.94000 16.14000 -7.83000 L

C 0 14.16000 16.25000 -9.72000 L

H 0 13.53000 15.59000 -10.12000 L

C 0 13.59000 17.72000 -9.79000 L

H 0 13.58000 18.03000 -10.74000 L

H 0 12.66000 17.73000 -9.43000 L

H 0 14.16000 18.33000 -9.25000 L

C 0 15.54000 16.33000 -10.46000 L

H 0 15.41000 16.74000 -11.36000 L

H 0 16.18000 16.88000 -9.93000 L

H 0 15.91000 15.40000 -10.57000 L

C 0 11.87000 15.01000 -8.39000 L

O 0 10.99000 15.71000 -8.92000 L

N 0 11.97000 13.70000 -8.64000 L

H 0 12.78000 13.19000 -8.34000 L

C 0 10.91000 13.04000 -9.35000 L

H 0 10.92000 13.50000 -10.24000 L

C 0 11.25000 11.52000 -9.47000 L

H 0 11.36000 11.13000 -8.55000 L

H 0 12.10000 11.41000 -9.98000 L

C 0 10.12000 10.78000 -10.19000 L

H 0 10.03000 11.16000 -11.12000 L

H 0 9.27000 10.91000 -9.69000 L

C 0 10.44000 9.27000 -10.28000 L

O 0 11.60000 8.91000 -9.93000 L

O 0 9.56000 8.46000 -10.72000 L

C 0 9.51000 13.23000 -8.70000 L

O 0 8.55000 13.55000 -9.40000 L

N 0 9.38000 13.05000 -7.39000 L

H 0 10.17000 12.77000 -6.85000 L

C 0 8.09000 13.25000 -6.71000 L

H 0 7.46000 12.59000 -7.13000 L

C 0 8.23000 13.03000 -5.19000 L

H 0 7.34000 13.17000 -4.75000 L

H 0 8.54000 12.10000 -5.02000 L

H 0 8.89000 13.68000 -4.82000 L

C 0 7.60000 14.67000 -6.94000 L

O 0 6.39000 14.91000 -7.16000 L

N 0 8.51000 15.67000 -6.86000 L

H 0 9.48000 15.47000 -6.71000 L

C 0 8.04000 17.07000 -7.00000 L

H 0 7.24000 17.12000 -6.40000 L

C 0 9.19000 18.08000 -6.61000 L

H 0 8.94000 19.01000 -6.88000 L

H 0 10.05000 17.82000 -7.04000 L

C 0 9.38000 18.06000 -5.12000 L

H 0 9.38000 17.13000 -4.78000 L

C 0 10.77000 18.71000 -4.85000 L

H 0 10.94000 18.72000 -3.87000 L

H 0 11.48000 18.17000 -5.31000 L

H 0 10.77000 19.64000 -5.20000 L

C 0 8.20000 18.85000 -4.45000 L

H 0 8.32000 18.85000 -3.46000 L

H 0 8.19000 19.80000 -4.78000 L

H 0 7.33000 18.42000 -4.68000 L

C 0 7.62000 17.37000 -8.44000 L

O 0 6.70000 18.19000 -8.66000 L

N 0 8.35000 16.81000 -9.42000 L

H 0 9.12000 16.21000 -9.21000 L

C 0 7.97000 17.10000 -10.83000 L

H 0 8.08000 18.09000 -10.97000 L

C 0 8.88000 16.35000 -11.84000 L

H 0 8.46000 16.40000 -12.74000 L

H 0 8.95000 15.40000 -11.55000 L

C 0 10.29000 16.93000 -11.94000 L

H 0 10.71000 17.12000 -11.05000 L

C 0 11.22000 15.92000 -12.57000 L

H 0 12.14000 16.32000 -12.63000 L

H 0 11.25000 15.09000 -12.02000 L

H 0 10.90000 15.70000 -13.49000 L

C 0 10.14000 18.23000 -12.72000 L

H 0 11.04000 18.66000 -12.82000 L

H 0 9.76000 18.04000 -13.62000 L

H 0 9.53000 18.85000 -12.23000 L

C 0 6.52000 16.58000 -11.02000 L

O 0 5.72000 17.26000 -11.65000 L

N 0 6.24000 15.41000 -10.49000 L

H 0 6.94000 14.90000 -9.99000 L

C 0 4.84000 14.86000 -10.64000 L

H 0 4.64000 14.82000 -11.62000 L

C 0 4.77000 13.50000 -10.00000 L

H 0 3.81000 13.21000 -9.98000 L

H 0 5.11000 13.57000 -9.06000 L

C 0 5.57000 12.44000 -10.73000 L

H 0 6.53000 12.71000 -10.78000 L

H 0 5.21000 12.31000 -11.66000 L

C 0 5.44000 11.15000 -9.94000 L

H 0 4.48000 10.90000 -9.89000 L

H 0 5.79000 11.30000 -9.02000 L

C 0 6.21000 10.03000 -10.59000 L

H 0 6.03000 9.17000 -10.11000 L

H 0 7.19000 10.23000 -10.57000 L

N 0 5.76000 9.92000 -12.00000 L

H 0 6.26000 9.18000 -12.45000 L

H 0 4.78000 9.73000 -12.02000 L

H 0 5.94000 10.78000 -12.47000 L

C 0 3.85000 15.74000 -9.90000 L

O 0 2.81000 16.16000 -10.43000 L

N 0 4.21000 16.09000 -8.68000 L

H 0 5.11000 15.83000 -8.32000 L

C 0 3.28000 16.86000 -7.90000 L

H 0 2.40000 16.39000 -7.85000 L

C 0 3.92000 17.00000 -6.51000 L

H 0 4.91000 17.10000 -6.61000 L

H 0 3.72000 16.18000 -5.97000 L

C 0 3.44000 18.14000 -5.76000 L

H 0 2.45000 18.20000 -5.86000 L

H 0 3.86000 18.97000 -6.13000 L

C 0 3.80000 17.99000 -4.28000 L

O 0 3.04000 18.51000 -3.40000 L

O 0 4.81000 17.28000 -4.04000 L

C 0 3.00000 18.19000 -8.58000 L

O 0 1.89000 18.72000 -8.51000 L

N 0 3.97000 18.73000 -9.32000 L

H 0 4.81000 18.23000 -9.49000 L

C 0 3.76000 20.07000 -9.86000 L

H 0 3.06000 20.48000 -9.28000 L

C 0 5.03000 20.92000 -9.76000 L

H 0 4.93000 21.71000 -10.37000 L

H 0 5.81000 20.37000 -10.06000 L

C 0 5.25000 21.41000 -8.28000 L

H 0 5.24000 20.62000 -7.66000 L

H 0 4.53000 22.05000 -8.02000 L

C 0 6.56000 22.12000 -8.06000 L

O 0 7.13000 22.66000 -8.99000 L

N 0 7.11000 22.02000 -6.83000 L

H 0 6.65000 21.50000 -6.11000 L

H 0 7.98000 22.48000 -6.63000 L

C 0 3.24000 19.99000 -11.30000 L

O 0 3.16000 21.01000 -11.96000 L

N 0 2.93000 18.78000 -11.78000 L

H 0 3.05000 17.96000 -11.22000 L

C 0 2.41000 18.68000 -13.15000 L

H 0 1.84000 19.49000 -13.28000 L

H 0 1.84000 17.86000 -13.16000 L

C 0 3.41000 18.60000 -14.30000 L

O 0 3.01000 18.62000 -15.47000 L

N 0 4.70000 18.43000 -14.05000 L

H 0 5.03000 18.30000 -13.11000 L

C 0 5.62000 18.44000 -15.20000 L

H 0 5.24000 19.04000 -15.90000 L

C 0 7.01000 18.93000 -14.72000 L

H 0 7.67000 18.83000 -15.46000 L

H 0 7.30000 18.38000 -13.93000 L

C 0 7.01000 20.37000 -14.29000 L

C 0 7.13000 20.70000 -12.96000 L

H 0 7.17000 19.97000 -12.27000 L

C 0 7.18000 22.04000 -12.54000 L

H 0 7.32000 22.26000 -11.58000 L

C 0 7.03000 23.05000 -13.47000 L

H 0 7.05000 24.00000 -13.18000 L

C 0 6.86000 22.73000 -14.81000 L

H 0 6.70000 23.46000 -15.47000 L

C 0 6.89000 21.40000 -15.24000 L

H 0 6.84000 21.19000 -16.21000 L

C 0 5.74000 17.03000 -15.78000 L

O 0 5.81000 16.06000 -15.01000 L

N 0 5.82000 16.89000 -17.11000 L

H 0 5.76000 17.67000 -17.72000 L

C 0 6.00000 15.54000 -17.63000 L

H 0 5.81000 15.57000 -18.61000 L

H 0 5.34000 14.95000 -17.18000 L

C 0 7.41000 15.01000 -17.40000 L

O 0 7.62000 13.81000 -17.32000 L

N 0 8.38000 15.89000 -17.26000 L

H 0 8.23000 16.86000 -17.45000 L

C 0 9.69000 15.41000 -16.81000 L

H 0 10.10000 14.83000 -17.52000 L

H 0 9.59000 14.88000 -15.97000 L

C 0 10.54000 16.65000 -16.58000 L

O 0 10.04000 17.78000 -16.70000 L

N 0 11.83000 16.45000 -16.30000 L

H 0 12.20000 15.52000 -16.29000 L

C 0 12.71000 17.56000 -16.01000 L

H 0 12.34000 17.99000 -15.19000 L

C 0 14.21000 16.99000 -15.93000 L

H 0 14.44000 16.35000 -16.66000 L

C 0 15.24000 18.19000 -16.05000 L

H 0 16.17000 17.83000 -16.00000 L

H 0 15.11000 18.65000 -16.93000 L

H 0 15.09000 18.84000 -15.31000 L

C 0 14.34000 16.20000 -14.64000 L

H 0 15.27000 15.83000 -14.56000 L

H 0 14.15000 16.79000 -13.86000 L

H 0 13.69000 15.44000 -14.65000 L

C 0 12.68000 18.56000 -17.13000 L

O 0 12.70000 19.75000 -16.96000 L

N 0 12.72000 18.07000 -18.38000 L

H 0 12.78000 17.08000 -18.54000 L

C 0 12.68000 19.00000 -19.49000 L

H 0 13.48000 19.55000 -19.27000 L

C 0 12.69000 18.16000 -20.88000 L

H 0 11.86000 17.62000 -21.06000 L

C 0 12.93000 19.11000 -22.10000 L

H 0 12.93000 18.58000 -22.95000 L

H 0 12.20000 19.79000 -22.14000 L

H 0 13.81000 19.57000 -22.00000 L

O 0 13.78000 17.22000 -20.77000 L

H 0 13.83000 16.66000 -21.60000 L

C 0 11.62000 20.09000 -19.49000 L

O 0 11.88000 21.28000 -19.80000 L

N 0 10.42000 19.71000 -19.10000 L

H 0 10.29000 18.77000 -18.79000 L

C 0 9.31000 20.59000 -19.11000 L

H 0 9.31000 21.02000 -20.01000 L

C 0 8.03000 19.74000 -18.73000 L

H 0 7.22000 20.32000 -18.71000 L

H 0 8.16000 19.31000 -17.83000 L

C 0 7.77000 18.63000 -19.72000 L

O 0 7.57000 17.47000 -19.29000 L

O 0 7.75000 18.96000 -20.94000 L

C 0 9.47000 21.63000 -18.03000 L

O 0 8.91000 22.72000 -18.14000 L

N 0 10.25000 21.35000 -17.00000 L

H 0 10.77000 20.50000 -16.99000 L

C 0 10.35000 22.28000 -15.85000 L

H 0 9.48000 22.77000 -15.82000 L

C 0 10.60000 21.50000 -14.49000 L

H 0 10.67000 22.16000 -13.74000 L

H 0 9.84000 20.87000 -14.32000 L

H 0 11.46000 20.98000 -14.55000 L

C 0 11.43000 23.29000 -16.09000 L

O 0 11.55000 24.29000 -15.36000 L

N 0 12.34000 23.01000 -17.07000 L

H 0 12.22000 22.22000 -17.67000 L

C 0 13.50000 23.89000 -17.22000 L

H 0 13.95000 23.85000 -16.33000 L

C 0 14.47000 23.41000 -18.34000 L

H 0 14.03000 23.33000 -19.23000 L

C 0 15.56000 24.47000 -18.54000 L

H 0 16.19000 24.18000 -19.26000 L

H 0 15.13000 25.34000 -18.81000 L

H 0 16.06000 24.60000 -17.69000 L

C 0 15.02000 21.98000 -17.96000 L

H 0 14.25000 21.38000 -17.73000 L

H 0 15.63000 22.05000 -17.18000 L

C 0 15.80000 21.36000 -19.18000 L

H 0 16.14000 20.45000 -18.93000 L

H 0 15.18000 21.28000 -19.97000 L

H 0 16.57000 21.95000 -19.42000 L

C 0 13.07000 25.31000 -17.50000 L

O 0 12.35000 25.58000 -18.49000 L

N 0 13.44000 26.26000 -16.65000 L

H 0 13.97000 26.04000 -15.83000 L

C 0 13.04000 27.67000 -16.95000 L

H 0 13.24000 27.84000 -17.92000 L

H 0 13.61000 28.27000 -16.39000 L

C 0 11.55000 27.98000 -16.68000 L

O 0 11.06000 29.09000 -16.98000 L

N 0 10.82000 27.07000 -16.06000 L

H 0 11.25000 26.24000 -15.71000 L

C 0 9.34000 27.28000 -15.89000 L

H 0 9.02000 27.39000 -16.83000 L

C 0 8.66000 26.06000 -15.18000 L

H 0 7.68000 26.24000 -15.08000 L

H 0 8.80000 25.24000 -15.72000 L

H 0 9.07000 25.93000 -14.27000 L

C 0 9.00000 28.55000 -15.12000 L

O 0 7.92000 29.17000 -15.41000 L

N 0 9.87000 29.00000 -14.18000 L

H 0 10.74000 28.53000 -14.02000 L

C 0 9.48000 30.21000 -13.39000 L

H 0 8.63000 29.95000 -12.93000 L

C 0 10.48000 30.61000 -12.35000 L

H 0 10.22000 31.51000 -12.00000 L

H 0 11.37000 30.68000 -12.80000 L

C 0 10.58000 29.64000 -11.19000 L

O 0 9.64000 28.84000 -10.96000 L

O 0 11.63000 29.70000 -10.50000 L

C 0 9.37000 31.42000 -14.33000 L

O 0 8.60000 32.37000 -14.04000 L

N 0 10.11000 31.39000 -15.44000 L

H 0 10.63000 30.57000 -15.67000 L

C 0 10.17000 32.58000 -16.32000 L

H 0 10.23000 33.36000 -15.70000 L

C 0 11.39000 32.59000 -17.22000 L

H 0 11.36000 33.41000 -17.80000 L

H 0 11.37000 31.78000 -17.80000 L

C 0 12.70000 32.60000 -16.44000 L

N 0 13.51000 33.72000 -16.33000 L

C 0 14.60000 33.44000 -15.61000 L

H 0 15.33000 34.08000 -15.37000 L

N 0 14.54000 32.15000 -15.25000 L

H 0 15.22000 31.65000 -14.72000 L

C 0 13.34000 31.62000 -15.75000 L

H 0 13.01000 30.68000 -15.62000 L

C 0 8.90000 32.65000 -17.19000 L

O 0 8.63000 33.65000 -17.78000 L

N 0 8.12000 31.60000 -17.21000 L

H 0 8.33000 30.80000 -16.64000 L

C 0 6.93000 31.59000 -18.08000 L

H 0 7.05000 32.37000 -18.70000 L

C 0 6.85000 30.27000 -18.85000 L

H 0 6.11000 30.33000 -19.52000 L

H 0 6.66000 29.53000 -18.21000 L

C 0 8.18000 29.99000 -19.59000 L

H 0 8.88000 29.79000 -18.91000 L

H 0 8.44000 30.81000 -20.10000 L

C 0 8.06000 28.79000 -20.58000 L

H 0 8.84000 28.78000 -21.20000 L

H 0 7.22000 28.87000 -21.11000 L

N 0 8.03000 27.49000 -19.90000 L

H 0 7.13000 27.10000 -19.68000 L

C 0 9.12000 26.78000 -19.57000 L

N 0 9.00000 25.59000 -18.98000 L

H 0 8.09000 25.21000 -18.79000 L

H 0 9.81000 25.06000 -18.73000 L

N 0 10.33000 27.27000 -19.81000 L

H 0 10.42000 28.16000 -20.25000 L

H 0 11.14000 26.74000 -19.56000 L

C 0 5.66000 31.79000 -17.27000 L

O 0 4.59000 31.78000 -17.84000 L

N 0 5.72000 32.00000 -15.96000 L

H 0 6.59000 31.94000 -15.47000 L

C 0 4.48000 32.33000 -15.24000 L

H 0 3.81000 31.81000 -15.78000 L

C 0 4.51000 31.86000 -13.79000 L

H 0 4.44000 30.87000 -13.75000 L

H 0 3.74000 32.27000 -13.29000 L

C 0 5.70000 32.22000 -13.09000 L

H 0 5.47000 32.92000 -12.41000 L

H 0 6.35000 32.59000 -13.75000 L

C 0 6.27000 30.96000 -12.40000 L

H 0 7.06000 31.22000 -11.85000 L

H 0 6.54000 30.30000 -13.09000 L

N 0 5.34000 30.28000 -11.50000 L

H 0 5.18000 29.30000 -11.66000 L

C 0 4.69000 30.86000 -10.49000 L

N 0 3.86000 30.13000 -9.74000 L

H 0 3.72000 29.16000 -9.94000 L

H 0 3.37000 30.56000 -8.98000 L

N 0 4.85000 32.15000 -10.22000 L

H 0 5.46000 32.71000 -10.78000 L

H 0 4.35000 32.57000 -9.46000 L

C 0 4.08000 33.81000 -15.25000 L

O 0 4.82000 34.68000 -15.71000 L

O 0 3.02000 34.15000 -14.69000 L

Figure S16. Structure of the model 2 subjected for ONIOM calculations (PM6:UFF).


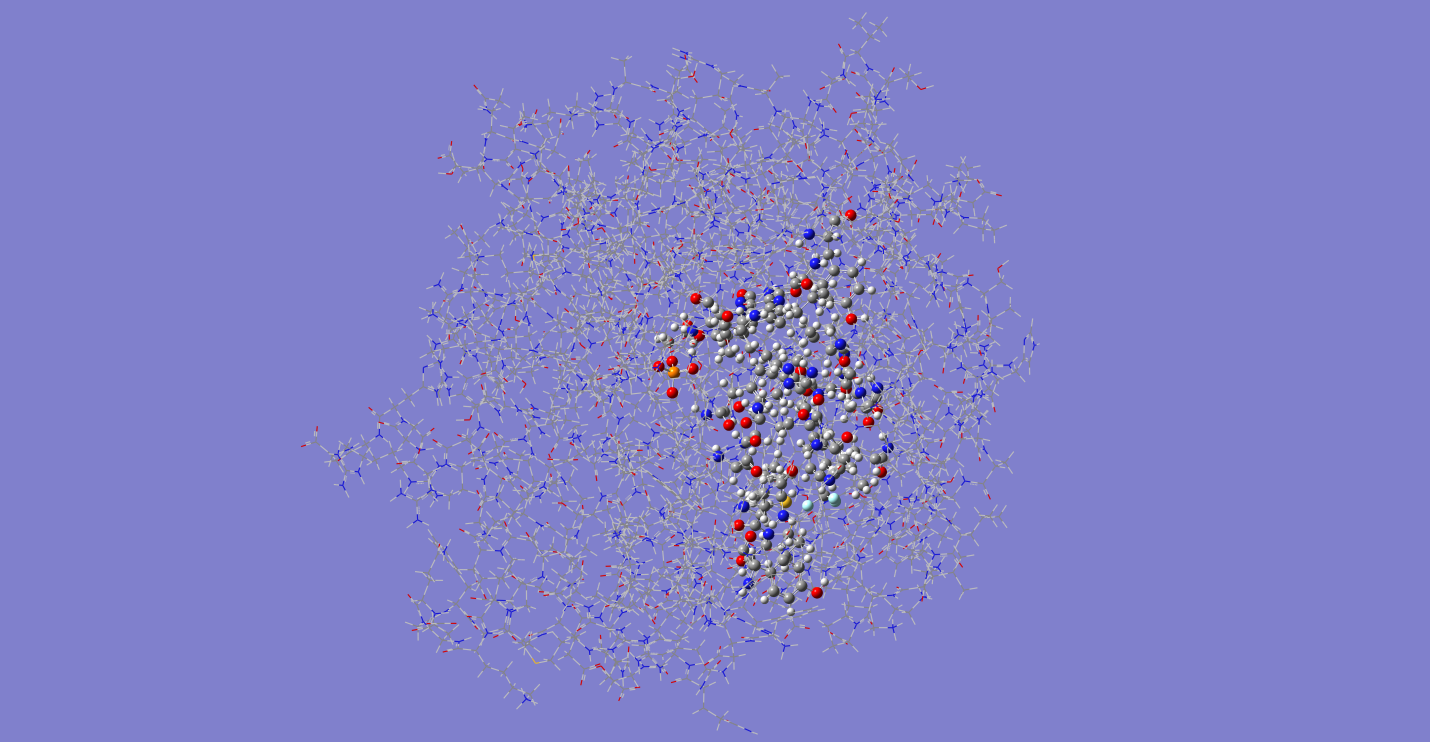


**Input file**:

%nproc=8

%mem=9000mb

# ONIOM(PM6:UFF)=EmbedCharge Opt=QuadMac

test teriflunomide-3G0U

2 1 -1 1

C 0 31.79600 9.48600 2.34800 H

C 0 32.36700 10.81100 2.21900 H

C 0 32.10100 11.65200 1.10200 H

C 0 32.72800 12.87200 1.09500 H

C 0 33.18700 11.28900 3.27300 H

C 0 33.81600 12.53800 3.26200 H

C 0 33.59300 13.34800 2.12700 H

N 0 34.17900 14.57900 1.99300 H

C 0 34.31700 15.39800 3.01800 H

O 0 33.93500 15.11700 4.14600 H

C 0 34.83700 16.75800 2.78600 H

C 0 34.88700 17.65300 3.90100 H

N 0 34.95700 18.44100 4.75600 H

C 0 35.22700 17.20600 1.38500 H

C 0 35.81400 18.51000 1.14400 H

O 0 35.14300 16.38200 0.33800 H

H 0 35.42700 16.83600 -0.44600 H

H 0 34.50900 14.85300 1.08100 H

H 0 36.01500 18.62600 0.08000 H

H 0 35.12200 19.28500 1.46900 H

H 0 36.74500 18.59800 1.70100 H

F 0 32.23500 8.90300 3.54200 H

F 0 30.39900 9.57900 2.36000 H

F 0 32.20200 8.69600 1.26600 H

H 0 31.43100 11.31400 0.31300 H

H 0 32.58100 13.55500 0.26000 H

H 0 33.35600 10.67200 4.15400 H

H 0 34.44000 12.84100 4.10200 H

N 0 34.90800 24.32700 -8.65800 H

C 0 35.71800 24.29200 -9.75100 H

O 0 35.32100 24.72000 -10.80300 H

N 0 36.98900 23.72700 -9.67300 H

C 0 37.49500 23.22500 -8.53800 H

O 0 38.61000 22.68600 -8.55800 H

C 0 36.72200 23.30400 -7.38000 H

N 0 37.21900 22.81800 -6.20200 H

C 0 36.41100 22.77700 -5.05000 H

C 0 36.89900 22.21700 -3.83500 H

C 0 36.04500 22.16300 -2.75000 H

C 0 36.38300 21.50300 -1.50400 H

C 0 34.71600 22.63100 -2.86900 H

C 0 33.78900 22.41500 -1.77500 H

C 0 34.22700 23.19300 -4.05700 H

C 0 35.07700 23.23300 -5.16800 H

N 0 34.59100 23.80000 -6.37200 H

C 0 35.38600 23.80100 -7.47100 H

C 0 33.24800 24.46200 -6.38300 H

C 0 32.11100 23.40400 -6.56300 H

O 0 32.38200 22.83300 -7.85500 H

C 0 30.74800 24.14800 -6.64300 H

O 0 30.81300 25.15700 -7.72600 H

C 0 30.50700 24.87800 -5.30400 H

O 0 31.08700 24.11200 -4.21500 H

C 0 29.00600 25.02700 -5.00000 H

O 0 28.40800 23.72000 -4.97500 H

P 0 28.05500 22.98100 -3.56400 H

O 0 29.32500 22.41900 -2.96700 H

O 0 27.12300 21.86800 -3.84400 H

O 0 27.41400 23.96700 -2.66000 H

H 0 37.53400 23.70600 -10.52100 H

H 0 37.91600 21.83700 -3.74700 H

H 0 33.21100 23.58300 -4.09700 H

H 0 35.55200 21.59600 -0.80700 H

H 0 36.58100 20.44900 -1.69400 H

H 0 37.27000 21.96700 -1.07600 H

H 0 32.81800 22.83600 -2.03400 H

H 0 33.68400 21.34600 -1.59400 H

H 0 34.16700 22.90000 -0.87700 H

H 0 33.09400 24.98700 -5.44100 H

H 0 33.20000 25.17300 -7.20700 H

H 0 32.11200 22.72200 -5.71400 H

H 0 31.73100 22.17000 -8.05100 H

H 0 29.94500 23.43300 -6.81700 H

H 0 29.98300 25.61600 -7.78100 H

H 0 30.96500 25.86400 -5.36400 H

H 0 30.40900 23.89100 -3.58700 H

H 0 28.53000 25.62900 -5.77300 H

H 0 28.87800 25.51100 -4.03300 H

N 0 23.69000 7.45000 7.79000 H

H 0 22.95000 7.18000 7.17000 H

C 0 24.81000 8.26000 7.27000 H

H 0 25.66000 7.80000 7.54000 H

C 0 24.74000 8.36000 5.74000 H

H 0 25.18000 9.21000 5.45000 H

H 0 23.78000 8.37000 5.46000 H

C 0 25.43000 7.21000 5.05000 H

C 0 24.83000 5.96000 4.96000 H

H 0 23.93000 5.83000 5.38000 H

C 0 25.44000 4.92000 4.32000 H

H 0 24.99000 4.03000 4.25000 H

C 0 26.69000 5.11000 3.77000 H

O 0 27.32000 4.07000 3.17000 H

H 0 28.21000 4.38000 2.83000 H

C 0 27.31000 6.32000 3.83000 H

H 0 28.20000 6.45000 3.40000 H

C 0 26.69000 7.37000 4.49000 H

H 0 27.15000 8.25000 4.56000 H

C 0 24.76000 9.64000 7.91000 H

O 0 25.76000 10.15000 8.40000 H

N 0 28.54000 10.00000 9.23000 H

H 0 27.55000 9.92000 9.11000 H

C 0 29.23000 11.23000 8.78000 H

H 0 30.00000 10.94000 8.21000 H

C 0 28.33000 12.13000 7.87000 H

H 0 28.86000 12.93000 7.59000 H

H 0 27.54000 12.42000 8.40000 H

C 0 27.84000 11.35000 6.57000 H

H 0 27.29000 12.00000 6.05000 H

H 0 27.26000 10.60000 6.90000 H

S 0 29.25000 10.72000 5.59000 H

C 0 29.98000 12.26000 4.94000 H

H 0 30.78000 12.04000 4.37000 H

H 0 29.31000 12.74000 4.38000 H

H 0 30.27000 12.85000 5.70000 H

C 0 29.75000 11.99000 10.02000 H

O 0 30.93000 12.27000 10.10000 H

N 0 32.74000 10.18000 11.32000 H

H 0 31.85000 9.91000 10.97000 H

C 0 33.83000 10.42000 10.39000 H

H 0 34.52000 9.73000 10.58000 H

C 0 33.30000 10.35000 8.97000 H

H 0 32.92000 11.23000 8.67000 H

H 0 32.61000 9.64000 8.87000 H

C 0 34.53000 10.01000 8.17000 H

H 0 35.39000 10.29000 8.61000 H

C 0 34.50000 8.48000 8.01000 H

H 0 35.30000 8.19000 7.48000 H

H 0 34.52000 8.05000 8.91000 H

H 0 33.67000 8.21000 7.53000 H

C 0 34.48000 10.78000 6.86000 H

H 0 35.29000 10.56000 6.32000 H

H 0 33.66000 10.51000 6.36000 H

H 0 34.46000 11.76000 7.05000 H

C 0 34.36000 11.80000 10.62000 H

O 0 35.56000 11.97000 10.69000 H

N 0 33.47000 12.78000 10.73000 H

H 0 32.50000 12.55000 10.72000 H

C 0 33.87000 14.22000 10.86000 H

H 0 34.49000 14.39000 10.09000 H

C 0 32.65000 15.19000 10.68000 H

H 0 32.95000 16.10000 10.98000 H

H 0 31.92000 14.86000 11.27000 H

C 0 32.06000 15.34000 9.18000 H

H 0 31.48000 14.54000 9.01000 H

H 0 32.84000 15.33000 8.55000 H

C 0 31.18000 16.67000 8.86000 H

O 0 30.22000 17.02000 9.63000 H

N 0 31.51000 17.40000 7.71000 H

H 0 32.27000 17.09000 7.13000 H

H 0 31.00000 18.22000 7.47000 H

C 0 34.64000 14.45000 12.20000 H

O 0 35.44000 15.37000 12.32000 H

N 0 39.04000 17.72000 7.56000 H

C 0 39.01000 19.00000 8.31000 H

H 0 39.84000 19.53000 8.12000 H

H 0 38.94000 18.83000 9.29000 H

C 0 37.78000 19.68000 7.78000 H

H 0 37.86000 20.67000 7.89000 H

H 0 36.97000 19.35000 8.26000 H

C 0 37.77000 19.26000 6.27000 H

H 0 38.41000 19.81000 5.74000 H

H 0 36.85000 19.33000 5.88000 H

C 0 38.24000 17.77000 6.33000 H

H 0 37.48000 17.12000 6.37000 H

C 0 39.05000 17.35000 5.09000 H

O 0 38.57000 16.55000 4.29000 H

N 0 39.41000 13.61000 5.21000 H

H 0 39.43000 14.46000 5.74000 H

C 0 38.16000 12.92000 4.98000 H

H 0 38.26000 11.98000 5.32000 H

C 0 37.01000 13.64000 5.79000 H

H 0 36.15000 13.17000 5.63000 H

H 0 37.23000 13.63000 6.76000 H

H 0 36.94000 14.59000 5.48000 H

C 0 37.90000 12.92000 3.40000 H

O 0 37.44000 11.96000 2.81000 H

N 0 38.21000 14.04000 2.75000 H

H 0 38.59000 14.82000 3.24000 H

C 0 37.99000 14.13000 1.29000 H

H 0 37.03000 13.93000 1.08000 H

C 0 38.37000 15.54000 0.89000 H

H 0 39.30000 15.73000 1.21000 H

H 0 37.73000 16.18000 1.34000 H

C 0 38.32000 15.81000 -0.58000 H

N 0 38.85000 16.99000 -1.12000 H

C 0 38.62000 17.00000 -2.43000 H

H 0 38.88000 17.74000 -3.06000 H

N 0 38.00000 15.87000 -2.77000 H

H 0 37.76000 15.59000 -3.70000 H

C 0 37.74000 15.14000 -1.61000 H

H 0 37.22000 14.29000 -1.55000 H

C 0 38.92000 13.13000 0.56000 H

O 0 38.50000 12.35000 -0.29000 H

N 0 37.54000 9.67000 1.03000 H

H 0 37.69000 10.53000 1.53000 H

C 0 36.32000 9.52000 0.23000 H

H 0 35.87000 8.72000 0.62000 H

C 0 35.38000 10.82000 0.31000 H

H 0 34.57000 10.67000 -0.26000 H

H 0 35.11000 10.97000 1.26000 H

H 0 35.89000 11.61000 -0.03000 H

C 0 36.74000 9.30000 -1.27000 H

O 0 36.12000 8.51000 -1.97000 H

N 0 35.52000 17.68000 -6.25000 H

H 0 34.77000 17.88000 -6.89000 H

C 0 35.58000 16.44000 -5.44000 H

H 0 35.81000 16.71000 -4.51000 H

C 0 34.23000 15.74000 -5.44000 H

H 0 33.91000 15.65000 -6.38000 H

H 0 33.58000 16.30000 -4.92000 H

C 0 34.29000 14.38000 -4.82000 H

C 0 34.44000 14.23000 -3.42000 H

H 0 34.44000 15.05000 -2.83000 H

C 0 34.61000 12.93000 -2.81000 H

H 0 34.71000 12.84000 -1.82000 H

C 0 34.64000 11.82000 -3.61000 H

H 0 34.75000 10.92000 -3.19000 H

C 0 34.51000 11.91000 -5.03000 H

H 0 34.53000 11.09000 -5.60000 H

C 0 34.34000 13.24000 -5.62000 H

H 0 34.26000 13.33000 -6.61000 H

C 0 36.64000 15.49000 -6.02000 H

O 0 37.52000 14.87000 -5.33000 H

N 0 40.91000 23.52000 4.05000 H

H 0 41.44000 23.17000 4.83000 H

C 0 39.52000 23.10000 3.90000 H

H 0 39.13000 23.73000 3.23000 H

C 0 39.35000 21.61000 3.39000 H

H 0 39.72000 20.94000 4.04000 H

C 0 37.80000 21.40000 3.08000 H

H 0 37.65000 20.47000 2.75000 H

H 0 37.27000 21.55000 3.91000 H

H 0 37.51000 22.06000 2.37000 H

C 0 40.15000 21.42000 2.13000 H

H 0 40.05000 20.48000 1.81000 H

H 0 39.82000 22.05000 1.43000 H

H 0 41.12000 21.60000 2.32000 H

C 0 38.92000 23.16000 5.32000 H

O 0 39.56000 22.67000 6.30000 H

N 0 35.20000 22.97000 7.78000 H

H 0 35.79000 22.65000 8.52000 H

C 0 33.78000 22.67000 7.78000 H

H 0 33.49000 22.75000 6.83000 H

C 0 33.58000 21.27000 8.34000 H

H 0 32.60000 21.10000 8.39000 H

H 0 33.97000 21.25000 9.26000 H

C 0 34.20000 20.11000 7.57000 H

H 0 35.19000 20.24000 7.49000 H

H 0 33.80000 20.05000 6.65000 H

C 0 33.82000 18.83000 8.47000 H

H 0 32.83000 18.69000 8.46000 H

H 0 34.12000 18.98000 9.41000 H

N 0 34.38000 17.55000 8.09000 H

H 0 33.95000 17.12000 7.31000 H

C 0 35.41000 16.85000 8.63000 H

N 0 35.66000 15.70000 8.05000 H

H 0 35.11000 15.41000 7.26000 H

H 0 36.40000 15.12000 8.38000 H

N 0 36.20000 17.23000 9.66000 H

H 0 36.06000 18.12000 10.10000 H

H 0 36.94000 16.63000 9.98000 H

C 0 33.02000 23.67000 8.71000 H

O 0 33.55000 24.07000 9.77000 H

N 0 30.89000 24.67000 3.76000 H

H 0 30.49000 25.08000 2.94000 H

C 0 32.32000 24.42000 3.79000 H

H 0 32.57000 24.28000 4.75000 H

C 0 32.71000 23.11000 2.94000 H

H 0 32.24000 23.11000 2.06000 H

C 0 34.26000 23.06000 2.57000 H

H 0 34.45000 22.23000 2.04000 H

H 0 34.50000 23.86000 2.02000 H

H 0 34.81000 23.05000 3.40000 H

C 0 32.23000 21.86000 3.71000 H

H 0 32.46000 21.04000 3.19000 H

H 0 32.67000 21.83000 4.60000 H

H 0 31.24000 21.90000 3.83000 H

C 0 33.00000 25.70000 3.30000 H

O 0 32.43000 26.42000 2.50000 H

N 0 41.78000 25.51000 -1.51000 H

H 0 40.81000 25.36000 -1.66000 H

C 0 42.80000 24.57000 -2.07000 H

H 0 43.29000 24.22000 -1.28000 H

C 0 42.07000 23.51000 -2.89000 H

H 0 42.65000 23.22000 -3.65000 H

H 0 41.22000 23.89000 -3.24000 H

C 0 41.73000 22.32000 -2.12000 H

C 0 40.44000 21.96000 -1.91000 H

H 0 39.70000 22.56000 -2.23000 H

C 0 40.12000 20.75000 -1.24000 H

H 0 39.16000 20.50000 -1.12000 H

C 0 41.11000 19.91000 -0.76000 H

O 0 40.73000 18.74000 -0.14000 H

H 0 41.55000 18.24000 0.15000 H

C 0 42.41000 20.20000 -0.93000 H

H 0 43.12000 19.59000 -0.58000 H

C 0 42.76000 21.44000 -1.65000 H

H 0 43.72000 21.67000 -1.81000 H

C 0 43.83000 25.22000 -2.96000 H

O 0 45.01000 24.89000 -2.89000 H

N 0 28.22000 19.15000 -4.44000 H

H 0 27.81000 19.98000 -4.06000 H

C 0 29.38000 18.59000 -3.80000 H

H 0 29.46000 17.63000 -4.08000 H

C 0 30.66000 19.31000 -4.29000 H

H 0 30.55000 20.29000 -4.10000 H

H 0 30.73000 19.18000 -5.28000 H

C 0 32.00000 18.85000 -3.66000 H

C 0 33.22000 19.53000 -3.99000 H

H 0 33.21000 20.28000 -4.65000 H

C 0 34.41000 19.16000 -3.40000 H

H 0 35.26000 19.63000 -3.63000 H

C 0 34.40000 18.13000 -2.47000 H

O 0 35.58000 17.80000 -1.93000 H

H 0 35.86000 17.65000 -2.98000 H

C 0 33.24000 17.42000 -2.14000 H

H 0 33.27000 16.65000 -1.51000 H

C 0 32.02000 17.83000 -2.73000 H

H 0 31.17000 17.37000 -2.48000 H

C 0 29.15000 18.66000 -2.29000 H

O 0 29.11000 17.61000 -1.61000 H

N 0 27.19000 16.20000 -0.13000 H

H 0 27.37000 16.74000 -0.95000 H

C 0 27.79000 14.81000 0.02000 H

H 0 27.06000 14.14000 0.15000 H

C 0 28.60000 14.50000 -1.26000 H

H 0 29.41000 15.09000 -1.27000 H

H 0 28.02000 14.72000 -2.05000 H

C 0 29.09000 13.09000 -1.47000 H

H 0 29.56000 12.68000 -0.70000 H

C 0 27.81000 12.28000 -1.79000 H

H 0 28.05000 11.32000 -1.94000 H

H 0 27.18000 12.34000 -1.01000 H

H 0 27.37000 12.65000 -2.61000 H

C 0 30.05000 13.22000 -2.73000 H

H 0 30.43000 12.32000 -2.95000 H

H 0 29.53000 13.56000 -3.51000 H

H 0 30.79000 13.86000 -2.52000 H

C 0 28.70000 14.77000 1.25000 H

O 0 28.80000 13.73000 1.91000 H

N 0 29.30000 15.90000 1.65000 H

H 0 29.11000 16.76000 1.17000 H

C 0 30.22000 15.89000 2.78000 H

H 0 30.72000 15.03000 2.66000 H

C 0 31.20000 17.09000 2.79000 H

H 0 31.96000 16.99000 3.43000 H

C 0 31.78000 17.26000 1.38000 H

H 0 32.41000 18.03000 1.37000 H

H 0 32.27000 16.42000 1.12000 H

H 0 31.04000 17.43000 0.73000 H

O 0 30.47000 18.26000 3.28000 H

H 0 31.08000 19.05000 3.29000 H

C 0 29.46000 15.88000 4.07000 H

O 0 30.01000 15.56000 5.12000 H

N 0 26.59000 12.19000 2.97000 H

H 0 27.00000 13.09000 2.83000 H

C 0 27.27000 11.01000 2.35000 H

H 0 27.30000 10.26000 3.01000 H

H 0 28.20000 11.26000 2.09000 H

C 0 26.49000 10.61000 1.14000 H

O 0 25.53000 11.30000 0.77000 H

N 0 26.91000 9.53000 0.48000 H

C 0 28.06000 8.72000 0.96000 H

H 0 27.79000 8.19000 1.77000 H

H 0 28.83000 9.31000 1.18000 H

C 0 28.36000 7.79000 -0.27000 H

H 0 28.75000 6.92000 0.03000 H

H 0 28.98000 8.23000 -0.91000 H

C 0 26.98000 7.57000 -0.92000 H

H 0 26.46000 6.87000 -0.42000 H

H 0 27.07000 7.29000 -1.87000 H

C 0 26.32000 9.00000 -0.77000 H

H 0 26.51000 9.68000 -1.48000 H

C 0 24.78000 8.90000 -0.83000 H

O 0 24.22000 9.08000 -1.91000 H

N -1 38.74300 26.11700 -7.20600 L

C -1 37.42200 26.52300 -7.53800 L

O -1 37.06600 26.75300 -8.72900 L

N -1 36.50000 26.75900 -6.62700 L

C -1 36.75200 26.57900 -5.30600 L

O -1 35.85200 26.73200 -4.43000 L

C -1 37.99700 26.16600 -4.92300 L

C -1 38.96900 25.91600 -5.85200 L

C -1 40.31900 25.56200 -5.30300 L

O -1 40.74000 26.22400 -4.31100 L

O -1 41.05100 24.78100 -5.91600 L

H -1 38.22500 26.03200 -3.86700 L

H -1 39.43100 25.99700 -7.93300 L

H -1 35.58600 27.08000 -6.90600 L

N -1 23.19000 2.27000 3.31000 L

H -1 24.04000 1.80000 3.09000 L

H -1 23.24000 2.65000 4.24000 L

H -1 22.43000 1.63000 3.25000 L

C -1 22.98000 3.38000 2.34000 L

H -1 22.93000 3.02000 1.41000 L

H -1 23.74000 4.03000 2.40000 L

C -1 21.70000 4.07000 2.70000 L

O -1 21.00000 4.60000 1.82000 L

N -1 21.39000 4.02000 3.99000 L

H -1 22.02000 3.52000 4.59000 L

C -1 20.21000 4.64000 4.61000 L

H -1 19.49000 4.68000 3.93000 L

C -1 19.75000 3.76000 5.75000 L

H -1 20.55000 3.42000 6.24000 L

H -1 19.23000 3.00000 5.38000 L

C -1 18.89000 4.47000 6.72000 L

O -1 19.41000 4.95000 7.76000 L

O -1 17.66000 4.49000 6.48000 L

C -1 20.55000 6.07000 5.09000 L

O -1 21.59000 6.33000 5.75000 L

N -1 19.70000 7.01000 4.65000 L

H -1 18.84000 6.74000 4.22000 L

C -1 20.06000 8.41000 4.79000 L

H -1 20.91000 8.52000 4.27000 L

C -1 19.00000 9.35000 4.21000 L

H -1 19.35000 10.29000 4.25000 L

H -1 18.17000 9.28000 4.78000 L

C -1 18.59000 9.09000 2.81000 L

H -1 18.56000 8.10000 2.67000 L

H -1 19.27000 9.49000 2.20000 L

C -1 17.22000 9.68000 2.50000 L

O -1 16.74000 10.59000 3.25000 L

O -1 16.63000 9.24000 1.49000 L

C -1 20.28000 8.71000 6.25000 L

O -1 21.24000 9.40000 6.60000 L

N -1 19.42000 8.15000 7.11000 L

H -1 18.74000 7.49000 6.79000 L

C -1 19.47000 8.50000 8.53000 L

H -1 19.47000 9.50000 8.52000 L

C -1 18.25000 7.96000 9.30000 L

H -1 18.51000 7.10000 9.74000 L

H -1 17.52000 7.80000 8.65000 L

C -1 17.73000 8.95000 10.41000 L

H -1 17.62000 9.86000 10.01000 L

H -1 18.40000 8.99000 11.15000 L

C -1 16.37000 8.50000 11.00000 L

H -1 16.04000 9.14000 11.69000 L

H -1 16.44000 7.58000 11.40000 L

N -1 15.42000 8.45000 9.92000 L

H -1 15.22000 9.32000 9.45000 L

C -1 14.78000 7.37000 9.48000 L

N -1 14.95000 6.19000 10.07000 L

H -1 15.56000 6.11000 10.86000 L

H -1 14.46000 5.39000 9.73000 L

N -1 13.95000 7.48000 8.45000 L

H -1 13.81000 8.37000 8.01000 L

H -1 13.47000 6.67000 8.11000 L

C -1 20.76000 8.08000 9.20000 L

O -1 21.41000 8.89000 9.92000 L

N -1 21.16000 6.84000 8.94000 L

H -1 20.61000 6.26000 8.33000 L

C -1 22.39000 6.30000 9.52000 L

H -1 22.24000 6.40000 10.50000 L

C -1 22.56000 4.84000 9.11000 L

H -1 22.54000 4.78000 8.11000 L

H -1 21.81000 4.31000 9.49000 L

C -1 23.86000 4.25000 9.59000 L

C -1 24.05000 3.98000 10.93000 L

H -1 23.31000 4.17000 11.58000 L

C -1 25.23000 3.45000 11.38000 L

H -1 25.36000 3.27000 12.35000 L

C -1 26.26000 3.18000 10.50000 L

H -1 27.12000 2.78000 10.83000 L

C -1 26.10000 3.44000 9.16000 L

H -1 26.84000 3.25000 8.51000 L

C -1 24.88000 3.98000 8.71000 L

H -1 24.76000 4.17000 7.73000 L

C 0 23.65000 7.08000 9.08000 L H 79

O -1 24.59000 7.27000 9.89000 L

N 0 23.58000 10.24000 7.88000 L H 98

H -1 22.80000 9.74000 7.51000 L

C -1 23.38000 11.61000 8.37000 L

H -1 24.05000 12.17000 7.90000 L

C -1 21.96000 12.06000 8.04000 L

H -1 21.81000 12.99000 8.37000 L

H -1 21.83000 12.03000 7.05000 L

H -1 21.30000 11.44000 8.48000 L

C -1 23.62000 11.74000 9.88000 L

O -1 24.39000 12.60000 10.34000 L

N -1 23.00000 10.86000 10.66000 L

H -1 22.47000 10.13000 10.24000 L

C -1 23.06000 10.94000 12.13000 L

H -1 23.14000 11.92000 12.33000 L

C -1 21.78000 10.33000 12.76000 L

H -1 21.83000 10.42000 13.75000 L

H -1 21.74000 9.36000 12.52000 L

C -1 20.51000 11.02000 12.25000 L

H -1 20.56000 11.09000 11.26000 L

H -1 20.47000 11.94000 12.65000 L

C -1 19.22000 10.28000 12.61000 L

O -1 19.12000 9.78000 13.76000 L

O -1 18.32000 10.22000 11.74000 L

C -1 24.30000 10.25000 12.71000 L

O -1 24.80000 10.66000 13.74000 L

N -1 24.81000 9.20000 12.07000 L

H -1 24.47000 8.93000 11.17000 L

C -1 25.88000 8.47000 12.74000 L

H -1 26.04000 9.02000 13.55000 L

C -1 25.38000 7.05000 13.11000 L

H -1 26.11000 6.55000 13.59000 L

H -1 25.13000 6.55000 12.28000 L

C -1 24.19000 7.08000 14.01000 L

N -1 22.91000 6.79000 13.57000 L

C -1 22.05000 6.99000 14.55000 L

H -1 21.06000 6.82000 14.50000 L

N -1 22.71000 7.44000 15.60000 L

H -1 22.31000 7.68000 16.48000 L

C -1 24.05000 7.51000 15.29000 L

H -1 24.79000 7.82000 15.89000 L

C -1 27.22000 8.45000 12.01000 L

O -1 28.28000 8.87000 12.56000 L

N -1 27.18000 7.94000 10.79000 L

H -1 26.31000 7.72000 10.37000 L

C -1 28.42000 7.69000 10.09000 L

H -1 28.95000 7.13000 10.72000 L

C -1 28.11000 6.94000 8.80000 L

H -1 27.73000 7.60000 8.15000 L

H -1 27.43000 6.24000 9.01000 L

C -1 29.26000 6.26000 8.11000 L

H -1 29.83000 5.73000 8.74000 L

C -1 28.65000 5.35000 7.05000 L

H -1 29.38000 4.87000 6.56000 L

H -1 28.05000 4.68000 7.49000 L

H -1 28.13000 5.90000 6.40000 L

C -1 30.09000 7.31000 7.48000 L

H -1 30.87000 6.89000 7.01000 L

H -1 29.54000 7.82000 6.82000 L

H -1 30.43000 7.94000 8.18000 L

C 0 29.22000 8.99000 9.80000 L H 100

O -1 30.43000 9.04000 10.07000 L

N 0 28.86000 12.23000 11.02000 L H 115

C -1 27.46000 11.78000 11.08000 L

H -1 27.41000 10.80000 11.26000 L

H -1 26.98000 11.99000 10.23000 L

C -1 26.90000 12.58000 12.25000 L

H -1 26.20000 12.05000 12.74000 L

H -1 26.49000 13.44000 11.93000 L

C -1 28.10000 12.87000 13.17000 L

H -1 28.20000 12.15000 13.85000 L

H -1 27.99000 13.75000 13.63000 L

C -1 29.33000 12.91000 12.24000 L

H -1 29.64000 13.83000 12.02000 L

C -1 30.53000 12.20000 12.87000 L

O -1 31.45000 12.85000 13.38000 L

N -1 30.53000 10.87000 12.85000 L

H -1 29.78000 10.37000 12.43000 L

C -1 31.66000 10.15000 13.45000 L

H -1 31.84000 10.59000 14.33000 L

C -1 31.25000 8.67000 13.62000 L

H -1 30.91000 8.26000 12.78000 L

C -1 32.43000 7.82000 14.11000 L

H -1 32.14000 6.87000 14.22000 L

H -1 33.18000 7.87000 13.45000 L

H -1 32.75000 8.17000 14.99000 L

O -1 30.18000 8.65000 14.58000 L

H -1 29.88000 7.71000 14.73000 L

C 0 32.93000 10.30000 12.63000 L H 117

O -1 34.05000 10.56000 13.13000 L

N 0 34.41000 13.57000 13.19000 L H 151

H -1 33.79000 12.80000 13.03000 L

C -1 35.07000 13.70000 14.49000 L

H -1 34.48000 13.30000 15.19000 L

H -1 35.21000 14.67000 14.69000 L

C -1 36.40000 12.99000 14.48000 L

O -1 37.23000 13.22000 15.33000 L

N -1 36.64000 12.12000 13.49000 L

H -1 35.97000 11.98000 12.77000 L

C -1 37.87000 11.40000 13.50000 L

H -1 38.13000 11.47000 14.46000 L

C -1 37.69000 9.96000 13.02000 L

H -1 38.60000 9.62000 12.82000 L

H -1 37.16000 10.03000 12.17000 L

C -1 37.00000 8.87000 13.84000 L

H -1 36.03000 9.06000 13.99000 L

C -1 37.16000 7.52000 13.06000 L

H -1 36.72000 6.79000 13.57000 L

H -1 36.73000 7.60000 12.16000 L

H -1 38.13000 7.32000 12.94000 L

C -1 37.67000 8.82000 15.20000 L

H -1 37.25000 8.12000 15.76000 L

H -1 38.65000 8.62000 15.08000 L

H -1 37.57000 9.71000 15.65000 L

C -1 38.93000 12.02000 12.60000 L

O -1 40.10000 11.82000 12.88000 L

N -1 38.55000 12.67000 11.50000 L

H -1 37.57000 12.85000 11.35000 L

C -1 39.52000 13.14000 10.51000 L

H -1 40.41000 12.92000 10.90000 L

C -1 39.22000 12.46000 9.18000 L

H -1 39.82000 12.85000 8.48000 L

H -1 38.26000 12.64000 8.94000 L

C -1 39.43000 10.94000 9.20000 L

H -1 39.11000 10.50000 10.04000 L

C -1 38.63000 10.38000 8.07000 L

H -1 38.74000 9.38000 8.05000 L

H -1 37.67000 10.61000 8.20000 L

H -1 38.96000 10.77000 7.21000 L

C -1 40.91000 10.64000 9.09000 L

H -1 41.06000 9.65000 9.10000 L

H -1 41.26000 11.02000 8.23000 L

H -1 41.40000 11.07000 9.85000 L

C -1 39.43000 14.64000 10.26000 L

O -1 38.35000 15.22000 10.34000 L

N -1 40.50000 15.28000 9.86000 L

H -1 41.41000 14.86000 9.87000 L

C -1 40.30000 16.66000 9.42000 L

H -1 39.75000 17.14000 10.10000 L

C -1 41.61000 17.38000 9.33000 L

H -1 42.07000 17.35000 10.22000 L

H -1 41.46000 18.33000 9.06000 L

C -1 42.51000 16.74000 8.31000 L

O -1 42.84000 15.55000 8.57000 L

O -1 42.80000 17.36000 7.24000 L

C 0 39.61000 16.59000 8.00000 L H 153

O -1 39.58000 15.50000 7.34000 L

N 0 40.29000 17.79000 4.94000 L H 165

H -1 40.71000 18.41000 5.59000 L

C -1 41.02000 17.31000 3.74000 L

H -1 40.41000 17.51000 2.96000 L

C -1 42.32000 18.08000 3.59000 L

H -1 42.93000 17.86000 4.35000 L

H -1 42.13000 19.06000 3.57000 L

C -1 42.96000 17.66000 2.28000 L

H -1 42.26000 17.67000 1.57000 L

H -1 43.31000 16.73000 2.39000 L

C -1 44.12000 18.57000 1.84000 L

O -1 44.93000 18.13000 1.00000 L

O -1 44.24000 19.72000 2.29000 L

C -1 41.27000 15.80000 3.74000 L

O -1 41.16000 15.09000 2.70000 L

N -1 41.67000 15.26000 4.92000 L

H -1 41.84000 15.84000 5.72000 L

C -1 41.83000 13.83000 5.02000 L

H -1 42.51000 13.64000 4.32000 L

C -1 42.27000 13.41000 6.45000 L

H -1 42.40000 12.42000 6.52000 L

H -1 41.61000 13.70000 7.14000 L

O -1 43.52000 14.03000 6.73000 L

H -1 43.83000 13.78000 7.65000 L

C 0 40.54000 13.11000 4.71000 L H 167

O -1 40.57000 12.01000 4.11000 L

N 0 40.20000 13.11000 0.93000 L H 192

H -1 40.53000 13.74000 1.63000 L

C -1 41.10000 12.16000 0.31000 L

H -1 41.05000 12.38000 -0.66000 L

C -1 42.53000 12.33000 0.87000 L

H -1 43.12000 11.61000 0.50000 L

H -1 42.51000 12.26000 1.87000 L

C -1 43.13000 13.68000 0.50000 L

H -1 42.50000 14.39000 0.79000 L

H -1 43.24000 13.71000 -0.49000 L

C -1 44.51000 13.90000 1.18000 L

H -1 44.42000 13.89000 2.17000 L

H -1 44.91000 14.77000 0.89000 L

N -1 45.45000 12.83000 0.80000 L

H -1 45.17000 12.21000 0.06000 L

C -1 46.65000 12.62000 1.37000 L

N -1 47.10000 13.42000 2.34000 L

H -1 46.55000 14.20000 2.65000 L

H -1 47.99000 13.25000 2.76000 L

N -1 47.41000 11.59000 0.96000 L

H -1 47.08000 10.99000 0.23000 L

H -1 48.30000 11.43000 1.38000 L

C -1 40.64000 10.73000 0.48000 L

O -1 40.77000 9.94000 -0.45000 L

N -1 40.20000 10.35000 1.69000 L

H -1 40.19000 11.01000 2.44000 L

C -1 39.71000 8.96000 1.92000 L

H -1 40.47000 8.37000 1.64000 L

C -1 39.36000 8.75000 3.38000 L

H -1 38.62000 9.39000 3.59000 L

H -1 40.17000 8.99000 3.91000 L

C -1 38.91000 7.37000 3.86000 L

H -1 38.10000 7.04000 3.35000 L

C -1 40.16000 6.41000 3.64000 L

H -1 39.92000 5.49000 3.93000 L

H -1 40.40000 6.40000 2.67000 L

H -1 40.93000 6.75000 4.18000 L

C -1 38.47000 7.45000 5.34000 L

H -1 38.17000 6.54000 5.65000 L

H -1 39.24000 7.76000 5.90000 L

H -1 37.71000 8.10000 5.43000 L

C 0 38.44000 8.68000 1.12000 L H 194

O -1 38.27000 7.57000 0.60000 L

N 0 37.73000 9.99000 -1.77000 L H 202

H -1 38.21000 10.66000 -1.22000 L

C -1 38.12000 9.74000 -3.20000 L

H -1 37.29000 9.82000 -3.76000 L

C -1 39.14000 10.82000 -3.67000 L

H -1 39.90000 10.82000 -3.02000 L

C -1 39.81000 10.49000 -5.02000 L

H -1 40.45000 11.21000 -5.27000 L

H -1 40.30000 9.62000 -4.94000 L

H -1 39.11000 10.40000 -5.73000 L

C -1 38.47000 12.26000 -3.66000 L

H -1 39.14000 12.94000 -3.96000 L

H -1 37.69000 12.26000 -4.28000 L

H -1 38.17000 12.48000 -2.73000 L

C -1 38.65000 8.30000 -3.35000 L

O -1 38.27000 7.54000 -4.27000 L

N -1 39.49000 7.90000 -2.40000 L

H -1 39.71000 8.53000 -1.65000 L

C -1 40.08000 6.57000 -2.43000 L

H -1 40.59000 6.53000 -3.29000 L

C -1 40.97000 6.34000 -1.20000 L

H -1 41.21000 5.37000 -1.14000 L

H -1 40.47000 6.61000 -0.38000 L

C -1 42.23000 7.10000 -1.22000 L

H -1 42.01000 8.06000 -1.04000 L

H -1 42.62000 7.02000 -2.14000 L

C -1 43.22000 6.50000 -0.11000 L

H -1 43.84000 5.83000 -0.53000 L

H -1 42.70000 6.05000 0.61000 L

N -1 44.08000 7.52000 0.55000 L

H -1 44.95000 7.74000 0.10000 L

C -1 43.82000 8.17000 1.70000 L

N -1 42.71000 7.94000 2.37000 L

H -1 42.05000 7.27000 2.03000 L

H -1 42.53000 8.44000 3.22000 L

N -1 44.69000 9.05000 2.20000 L

H -1 45.55000 9.23000 1.73000 L

H -1 44.48000 9.53000 3.06000 L

C -1 39.03000 5.52000 -2.38000 L

O -1 39.01000 4.55000 -3.18000 L

N -1 38.14000 5.65000 -1.42000 L

H -1 38.18000 6.44000 -0.80000 L

C -1 37.13000 4.68000 -1.27000 L

H -1 37.59000 3.82000 -1.06000 L

C -1 36.22000 5.16000 -0.17000 L

H -1 35.74000 5.98000 -0.48000 L

H -1 36.76000 5.38000 0.64000 L

C -1 35.22000 4.21000 0.23000 L

C -1 35.51000 3.29000 1.22000 L

H -1 36.42000 3.27000 1.63000 L

C -1 34.53000 2.38000 1.67000 L

H -1 34.76000 1.71000 2.37000 L

C -1 33.22000 2.42000 1.13000 L

H -1 32.53000 1.78000 1.45000 L

C -1 32.91000 3.36000 0.14000 L

H -1 31.99000 3.38000 -0.26000 L

C -1 33.91000 4.28000 -0.29000 L

H -1 33.69000 4.98000 -0.96000 L

C -1 36.28000 4.58000 -2.52000 L

O -1 35.86000 3.46000 -2.93000 L

N -1 35.92000 5.74000 -3.06000 L

H -1 36.27000 6.60000 -2.69000 L

C -1 35.02000 5.75000 -4.21000 L

H -1 34.20000 5.24000 -3.96000 L

C -1 34.69000 7.21000 -4.57000 L

H -1 35.52000 7.75000 -4.72000 L

C -1 33.84000 7.31000 -5.85000 L

H -1 33.65000 8.27000 -6.05000 L

H -1 34.34000 6.90000 -6.61000 L

H -1 32.98000 6.82000 -5.71000 L

O -1 34.02000 7.81000 -3.46000 L

H -1 33.79000 8.76000 -3.68000 L

C -1 35.74000 5.11000 -5.38000 L

O -1 35.13000 4.39000 -6.18000 L

N -1 37.02000 5.41000 -5.54000 L

H -1 37.50000 5.97000 -4.86000 L

C -1 37.71000 4.92000 -6.71000 L

H -1 37.11000 5.16000 -7.46000 L

C -1 39.03000 5.57000 -6.86000 L

H -1 38.97000 6.56000 -6.74000 L

H -1 39.45000 5.36000 -7.75000 L

O -1 39.81000 5.03000 -5.85000 L

H -1 40.73000 5.43000 -5.89000 L

C -1 37.97000 3.42000 -6.61000 L

O -1 38.14000 2.74000 -7.63000 L

N -1 38.05000 2.90000 -5.40000 L

H -1 37.98000 3.49000 -4.60000 L

C -1 38.23000 1.46000 -5.24000 L

H -1 38.90000 1.22000 -5.95000 L

C -1 38.78000 1.08000 -3.87000 L

H -1 38.78000 0.09000 -3.77000 L

H -1 38.20000 1.49000 -3.16000 L

C -1 40.19000 1.60000 -3.69000 L

H -1 40.14000 2.58000 -3.82000 L

C -1 40.68000 1.41000 -2.29000 L

H -1 41.61000 1.76000 -2.21000 L

H -1 40.08000 1.91000 -1.66000 L

H -1 40.66000 0.44000 -2.05000 L

C -1 41.20000 0.98000 -4.72000 L

H -1 42.11000 1.35000 -4.56000 L

H -1 41.21000 -0.01000 -4.62000 L

H -1 40.90000 1.22000 -5.65000 L

C -1 36.94000 0.73000 -5.48000 L

O -1 36.95000 -0.49000 -5.44000 L

N -1 35.83000 1.43000 -5.68000 L

H -1 35.85000 2.43000 -5.65000 L

C -1 34.61000 0.75000 -5.94000 L

H -1 34.79000 0.07000 -6.65000 L

H -1 33.96000 1.42000 -6.29000 L

C -1 34.02000 0.06000 -4.73000 L

O -1 33.13000 -0.80000 -4.86000 L

N -1 34.44000 0.49000 -3.54000 L

H -1 35.18000 1.16000 -3.50000 L

C -1 33.85000 -0.01000 -2.29000 L

H -1 33.69000 -0.99000 -2.42000 L

C -1 34.83000 0.21000 -1.13000 L

H -1 34.46000 -0.21000 -0.30000 L

H -1 34.94000 1.20000 -0.98000 L

C -1 36.19000 -0.39000 -1.42000 L

H -1 36.46000 -0.19000 -2.36000 L

C -1 37.27000 0.18000 -0.52000 L

H -1 38.15000 -0.24000 -0.74000 L

H -1 37.33000 1.17000 -0.66000 L

H -1 37.04000 -0.01000 0.44000 L

C -1 36.08000 -1.88000 -1.23000 L

H -1 36.97000 -2.31000 -1.42000 L

H -1 35.81000 -2.08000 -0.29000 L

H -1 35.40000 -2.25000 -1.86000 L

C -1 32.51000 0.66000 -2.00000 L

O -1 32.24000 1.10000 -0.90000 L

N -1 31.67000 0.78000 -2.99000 L

H -1 31.86000 0.37000 -3.88000 L

C -1 30.45000 1.53000 -2.77000 L

H -1 30.51000 1.91000 -1.84000 L

C -1 30.29000 2.61000 -3.85000 L

H -1 29.33000 2.90000 -3.79000 L

H -1 30.44000 2.15000 -4.72000 L

C -1 31.07000 3.91000 -3.97000 L

H -1 32.05000 3.75000 -3.81000 L

C -1 30.89000 4.44000 -5.41000 L

H -1 31.40000 5.29000 -5.51000 L

H -1 31.24000 3.76000 -6.06000 L

H -1 29.92000 4.61000 -5.58000 L

C -1 30.60000 4.91000 -2.89000 L

H -1 31.12000 5.76000 -2.97000 L

H -1 29.63000 5.11000 -3.02000 L

H -1 30.74000 4.52000 -1.98000 L

C -1 29.30000 0.59000 -2.96000 L

O -1 29.25000 -0.08000 -3.99000 L

N -1 28.34000 0.58000 -2.02000 L

C -1 28.07000 1.51000 -0.91000 L

H -1 28.53000 2.38000 -1.07000 L

H -1 28.39000 1.12000 -0.04000 L

C -1 26.55000 1.66000 -0.93000 L

H -1 26.27000 2.39000 -1.55000 L

H -1 26.20000 1.86000 -0.01000 L

C -1 26.07000 0.28000 -1.43000 L

H -1 25.17000 0.35000 -1.86000 L

H -1 26.03000 -0.38000 -0.68000 L

C -1 27.14000 -0.13000 -2.47000 L

H -1 27.28000 -1.12000 -2.53000 L

C -1 26.77000 0.43000 -3.84000 L

O -1 26.37000 1.34000 -3.91000 L

O -1 26.97000 -0.23000 -4.86000 L

N -1 18.49000 3.33000 -6.29000 L

H -1 18.32000 2.37000 -6.51000 L

H -1 19.45000 3.54000 -6.47000 L

H -1 18.30000 3.50000 -5.32000 L

C -1 17.63000 4.19000 -7.12000 L

H -1 16.71000 3.93000 -6.84000 L

C -1 17.87000 5.70000 -6.83000 L

H -1 18.77000 5.97000 -7.16000 L

H -1 17.82000 5.86000 -5.84000 L

C -1 16.85000 6.59000 -7.49000 L

C -1 15.64000 6.87000 -6.87000 L

H -1 15.46000 6.52000 -5.95000 L

C -1 14.67000 7.63000 -7.52000 L

H -1 13.80000 7.81000 -7.07000 L

C -1 14.92000 8.16000 -8.77000 L

H -1 14.23000 8.72000 -9.23000 L

C -1 16.14000 7.89000 -9.40000 L

H -1 16.33000 8.28000 -10.30000 L

C -1 17.07000 7.08000 -8.77000 L

H -1 17.91000 6.85000 -9.26000 L

C -1 17.78000 3.89000 -8.62000 L

O -1 18.83000 4.10000 -9.20000 L

N -1 16.73000 3.37000 -9.25000 L

H -1 15.94000 3.08000 -8.71000 L

C -1 16.71000 3.19000 -10.71000 L

H -1 17.64000 3.03000 -11.03000 L

C -1 15.84000 2.01000 -11.07000 L

H -1 15.72000 2.00000 -12.06000 L

H -1 14.95000 2.13000 -10.63000 L

C -1 16.41000 0.66000 -10.64000 L

H -1 15.70000 0.13000 -10.18000 L

H -1 17.18000 0.81000 -10.02000 L

C -1 16.91000 -0.10000 -11.87000 L

O -1 16.68000 0.35000 -13.01000 L

N -1 17.60000 -1.25000 -11.66000 L

H -1 17.77000 -1.56000 -10.73000 L

H -1 17.94000 -1.78000 -12.44000 L

C -1 16.08000 4.45000 -11.28000 L

O -1 14.98000 4.86000 -10.85000 L

N -1 16.79000 5.10000 -12.19000 L

H -1 17.64000 4.69000 -12.53000 L

C -1 16.36000 6.40000 -12.71000 L

H -1 16.15000 6.94000 -11.89000 L

C -1 17.47000 7.09000 -13.54000 L

H -1 17.12000 7.94000 -13.91000 L

H -1 17.73000 6.47000 -14.28000 L

C -1 18.74000 7.41000 -12.71000 L

O -1 19.82000 7.47000 -13.30000 L

O -1 18.63000 7.58000 -11.48000 L

C -1 15.12000 6.22000 -13.60000 L

O -1 15.14000 5.49000 -14.58000 L

N -1 14.05000 6.93000 -13.31000 L

H -1 14.00000 7.47000 -12.47000 L

C -1 12.94000 6.91000 -14.26000 L

H -1 12.87000 5.97000 -14.57000 L

C -1 11.67000 7.36000 -13.54000 L

H -1 11.69000 7.10000 -12.58000 L

H -1 10.86000 6.96000 -13.97000 L

O -1 11.56000 8.75000 -13.61000 L

H -1 10.72000 9.04000 -13.14000 L

C -1 13.21000 7.79000 -15.48000 L

O -1 14.00000 8.74000 -15.43000 L

N -1 12.55000 7.46000 -16.58000 L

H -1 12.01000 6.62000 -16.60000 L

C -1 12.62000 8.28000 -17.74000 L

H -1 13.57000 8.15000 -18.00000 L

C -1 11.56000 7.80000 -18.75000 L

H -1 11.47000 8.47000 -19.49000 L

H -1 10.68000 7.70000 -18.29000 L

C -1 11.92000 6.45000 -19.37000 L

O -1 11.14000 5.96000 -20.21000 L

O -1 12.98000 5.88000 -19.00000 L

C -1 12.34000 9.79000 -17.52000 L

O -1 12.81000 10.61000 -18.32000 L

N -1 11.51000 10.15000 -16.53000 L

H -1 11.13000 9.45000 -15.92000 L

C -1 11.16000 11.59000 -16.33000 L

H -1 10.72000 11.87000 -17.18000 L

C -1 10.23000 11.79000 -15.10000 L

H -1 10.12000 12.77000 -14.95000 L

H -1 10.67000 11.38000 -14.30000 L

C -1 8.82000 11.18000 -15.21000 L

H -1 8.97000 10.20000 -15.30000 L

H -1 8.45000 11.55000 -16.06000 L

S -1 7.81000 11.63000 -13.71000 L

C -1 7.84000 13.43000 -13.89000 L

H -1 7.33000 13.85000 -13.14000 L

H -1 8.79000 13.75000 -13.86000 L

H -1 7.43000 13.69000 -14.76000 L

C -1 12.43000 12.37000 -16.01000 L

O -1 12.41000 13.59000 -16.01000 L

N -1 13.50000 11.66000 -15.62000 L

H -1 13.48000 10.67000 -15.63000 L

C -1 14.72000 12.38000 -15.16000 L

H -1 14.40000 13.27000 -14.83000 L

C -1 15.39000 11.65000 -14.01000 L

H -1 16.29000 12.07000 -13.89000 L

H -1 15.50000 10.70000 -14.30000 L

C -1 14.69000 11.65000 -12.63000 L

H -1 13.91000 11.03000 -12.67000 L

C -1 15.64000 11.08000 -11.57000 L

H -1 15.19000 11.08000 -10.68000 L

H -1 15.90000 10.15000 -11.82000 L

H -1 16.47000 11.65000 -11.53000 L

C -1 14.17000 13.09000 -12.25000 L

H -1 13.72000 13.06000 -11.36000 L

H -1 14.94000 13.73000 -12.22000 L

H -1 13.51000 13.39000 -12.94000 L

C -1 15.69000 12.64000 -16.32000 L

O -1 16.67000 13.42000 -16.19000 L

N -1 15.39000 12.07000 -17.48000 L

H -1 14.52000 11.59000 -17.60000 L

C -1 16.35000 12.14000 -18.57000 L

H -1 17.25000 12.03000 -18.16000 L

C -1 16.06000 11.02000 -19.59000 L

H -1 15.10000 11.04000 -19.85000 L

H -1 16.28000 10.13000 -19.18000 L

C -1 16.92000 11.21000 -20.85000 L

H -1 17.88000 11.09000 -20.61000 L

H -1 16.78000 12.14000 -21.20000 L

C -1 16.51000 10.19000 -21.93000 L

O -1 16.16000 10.62000 -23.06000 L

O -1 16.48000 8.97000 -21.61000 L

C -1 16.31000 13.49000 -19.23000 L

O -1 15.25000 14.08000 -19.40000 L

N -1 17.44000 14.01000 -19.59000 L

H -1 18.29000 13.50000 -19.44000 L

C -1 17.48000 15.29000 -20.18000 L

H -1 16.54000 15.48000 -20.43000 L

C -1 18.17000 16.32000 -19.19000 L

H -1 19.05000 15.99000 -18.84000 L

C -1 18.44000 17.69000 -19.91000 L

H -1 18.87000 18.32000 -19.27000 L

H -1 19.04000 17.54000 -20.69000 L

H -1 17.57000 18.07000 -20.22000 L

C -1 17.37000 16.43000 -17.97000 L

H -1 17.80000 17.08000 -17.34000 L

H -1 16.45000 16.76000 -18.21000 L

H -1 17.30000 15.54000 -17.53000 L

C -1 18.47000 15.21000 -21.36000 L

O -1 19.52000 14.55000 -21.26000 L

N -1 18.13000 15.86000 -22.45000 L

H -1 17.25000 16.32000 -22.49000 L

C -1 19.01000 15.92000 -23.62000 L

H -1 19.76000 15.29000 -23.43000 L

C -1 18.19000 15.54000 -24.89000 L

H -1 17.29000 15.98000 -24.83000 L

H -1 18.07000 14.55000 -24.92000 L

C -1 18.87000 15.99000 -26.18000 L

H -1 19.84000 16.06000 -25.98000 L

H -1 18.50000 16.90000 -26.39000 L

C -1 18.68000 15.06000 -27.48000 L

H -1 18.76000 15.61000 -28.31000 L

H -1 17.79000 14.61000 -27.45000 L

N -1 19.72000 13.99000 -27.57000 L

H -1 19.43000 13.05000 -27.41000 L

C -1 21.01000 14.19000 -27.86000 L

N -1 21.47000 15.42000 -28.10000 L

H -1 20.84000 16.20000 -28.06000 L

H -1 22.43000 15.57000 -28.31000 L

N -1 21.86000 13.16000 -27.89000 L

H -1 21.52000 12.24000 -27.70000 L

H -1 22.82000 13.31000 -28.10000 L

C -1 19.56000 17.32000 -23.81000 L

O -1 18.75000 18.23000 -24.08000 L

N -1 20.87000 17.56000 -23.63000 L

H -1 21.47000 16.81000 -23.35000 L

C -1 21.44000 18.92000 -23.84000 L

H -1 20.78000 19.30000 -24.49000 L

C -1 21.72000 19.76000 -22.53000 L

H -1 22.71000 19.92000 -22.46000 L

C -1 21.09000 21.17000 -22.58000 L

H -1 21.29000 21.66000 -21.73000 L

H -1 21.46000 21.68000 -23.36000 L

H -1 20.09000 21.09000 -22.69000 L

C -1 21.61000 19.02000 -21.23000 L

H -1 21.81000 19.65000 -20.48000 L

H -1 20.69000 18.66000 -21.13000 L

H -1 22.27000 18.27000 -21.22000 L

C -1 22.88000 18.79000 -24.23000 L

O -1 23.42000 17.74000 -24.05000 L

N -1 23.54000 19.87000 -24.65000 L

H -1 23.03000 20.71000 -24.84000 L

C -1 25.00000 19.85000 -24.85000 L

H -1 25.18000 20.73000 -25.29000 L

C -1 25.80000 19.71000 -23.47000 L

H -1 26.78000 19.67000 -23.68000 L

H -1 25.52000 18.86000 -23.02000 L

C -1 25.56000 20.91000 -22.47000 L

H -1 24.57000 20.99000 -22.36000 L

C -1 26.15000 20.68000 -21.06000 L

H -1 25.95000 21.48000 -20.48000 L

H -1 25.73000 19.87000 -20.65000 L

H -1 27.14000 20.55000 -21.12000 L

C -1 26.01000 22.23000 -23.02000 L

H -1 25.83000 22.94000 -22.35000 L

H -1 26.99000 22.19000 -23.22000 L

H -1 25.51000 22.42000 -23.86000 L

C -1 25.41000 18.73000 -25.80000 L

O -1 26.59000 18.38000 -25.85000 L

N -1 24.45000 18.21000 -26.58000 L

H -1 23.51000 18.50000 -26.45000 L

C -1 24.78000 17.21000 -27.63000 L

H -1 25.66000 17.45000 -28.04000 L

H -1 24.07000 17.23000 -28.33000 L

C -1 24.86000 15.84000 -27.04000 L

O -1 25.45000 14.89000 -27.64000 L

N -1 24.31000 15.72000 -25.83000 L

H -1 23.96000 16.55000 -25.39000 L

C -1 24.19000 14.46000 -25.12000 L

H -1 24.21000 13.82000 -25.89000 L

C -1 25.42000 14.33000 -24.14000 L

H -1 25.35000 13.47000 -23.63000 L

H -1 25.42000 15.09000 -23.50000 L

C -1 26.73000 14.33000 -24.87000 L

N -1 27.28000 13.17000 -25.40000 L

C -1 28.41000 13.47000 -26.03000 L

H -1 29.02000 12.82000 -26.49000 L

N -1 28.59000 14.79000 -25.96000 L

H -1 29.35000 15.29000 -26.37000 L

C -1 27.57000 15.34000 -25.23000 L

H -1 27.46000 16.31000 -25.00000 L

C -1 22.81000 14.15000 -24.39000 L

O -1 22.00000 15.01000 -23.99000 L

N -1 22.61000 12.88000 -24.13000 L

H -1 23.23000 12.21000 -24.54000 L

C -1 21.55000 12.41000 -23.28000 L

H -1 20.77000 13.03000 -23.36000 L

C -1 21.18000 11.00000 -23.69000 L

H -1 22.02000 10.46000 -23.77000 L

H -1 20.72000 11.03000 -24.57000 L

C -1 20.27000 10.33000 -22.70000 L

H -1 19.56000 10.97000 -22.42000 L

H -1 20.80000 10.05000 -21.90000 L

C -1 19.63000 9.09000 -23.35000 L

H -1 19.31000 9.33000 -24.26000 L

H -1 18.85000 8.80000 -22.79000 L

C -1 20.59000 7.91000 -23.48000 L

H -1 20.96000 7.68000 -22.58000 L

H -1 21.34000 8.15000 -24.10000 L

N -1 19.79000 6.72000 -24.04000 L

H -1 20.39000 5.93000 -24.14000 L

H -1 19.04000 6.50000 -23.42000 L

H -1 19.42000 6.97000 -24.94000 L

C -1 22.15000 12.31000 -21.88000 L

O -1 23.28000 11.84000 -21.75000 L

N -1 21.38000 12.66000 -20.83000 L

H -1 20.45000 12.97000 -20.98000 L

C -1 21.92000 12.57000 -19.44000 L

H -1 22.85000 12.19000 -19.40000 L

C -1 21.97000 13.96000 -18.76000 L

H -1 22.08000 13.85000 -17.78000 L

H -1 21.11000 14.44000 -18.95000 L

C -1 23.10000 14.82000 -19.27000 L

C -1 23.05000 15.39000 -20.52000 L

H -1 22.25000 15.21000 -21.10000 L

C -1 24.09000 16.22000 -21.01000 L

H -1 24.01000 16.65000 -21.90000 L

C -1 25.26000 16.42000 -20.21000 L

H -1 26.03000 16.95000 -20.56000 L

C -1 25.30000 15.85000 -18.94000 L

H -1 26.07000 16.04000 -18.33000 L

C -1 24.25000 15.01000 -18.50000 L

H -1 24.34000 14.54000 -17.62000 L

C -1 20.88000 11.71000 -18.77000 L

O -1 19.71000 12.11000 -18.75000 L

N -1 21.22000 10.51000 -18.32000 L

H -1 22.15000 10.17000 -18.37000 L

C -1 20.16000 9.72000 -17.73000 L

H -1 19.53000 9.71000 -18.51000 L

C -1 20.64000 8.30000 -17.39000 L

H -1 21.35000 8.02000 -18.04000 L

H -1 19.87000 7.67000 -17.45000 L

C -1 21.22000 8.05000 -16.11000 L

H -1 20.69000 8.57000 -15.44000 L

H -1 22.16000 8.39000 -16.13000 L

C -1 21.15000 6.48000 -15.84000 L

H -1 20.20000 6.19000 -15.70000 L

H -1 21.70000 6.24000 -15.04000 L

N -1 21.68000 5.71000 -16.99000 L

H -1 21.52000 6.08000 -17.91000 L

C -1 22.34000 4.55000 -16.90000 L

N -1 22.56000 4.00000 -15.69000 L

H -1 22.23000 4.46000 -14.87000 L

H -1 23.06000 3.14000 -15.62000 L

N -1 22.77000 3.92000 -18.00000 L

H -1 22.60000 4.31000 -18.90000 L

H -1 23.27000 3.06000 -17.92000 L

C -1 19.43000 10.36000 -16.52000 L

O -1 18.21000 10.08000 -16.29000 L

N -1 20.13000 11.21000 -15.76000 L

H -1 21.12000 11.33000 -15.89000 L

C -1 19.40000 11.98000 -14.72000 L

H -1 18.45000 12.11000 -14.99000 L

C -1 19.35000 11.20000 -13.36000 L

H -1 19.04000 10.26000 -13.52000 L

H -1 18.70000 11.65000 -12.74000 L

C -1 20.68000 11.13000 -12.67000 L

O -1 21.26000 12.17000 -12.30000 L

N -1 21.23000 9.94000 -12.54000 L

H -1 20.75000 9.13000 -12.90000 L

H -1 22.11000 9.84000 -12.09000 L

C -1 20.19000 13.33000 -14.65000 L

O -1 21.35000 13.40000 -15.16000 L

N -1 19.56000 14.40000 -14.11000 L

C -1 18.24000 14.49000 -13.41000 L

H -1 18.27000 13.99000 -12.55000 L

H -1 17.52000 14.13000 -13.99000 L

C -1 18.08000 16.03000 -13.17000 L

H -1 18.37000 16.29000 -12.26000 L

H -1 17.13000 16.31000 -13.31000 L

C -1 18.98000 16.65000 -14.23000 L

H -1 19.24000 17.58000 -13.97000 L

H -1 18.54000 16.66000 -15.13000 L

C -1 20.20000 15.70000 -14.21000 L

H -1 20.81000 15.69000 -15.01000 L

C -1 21.11000 16.06000 -13.04000 L

O -1 21.47000 17.25000 -12.89000 L

N -1 21.49000 15.08000 -12.21000 L

H -1 21.25000 14.13000 -12.43000 L

C -1 22.25000 15.36000 -10.96000 L

H -1 22.19000 16.36000 -10.86000 L

C -1 21.63000 14.65000 -9.73000 L

H -1 21.70000 13.66000 -9.87000 L

C -1 22.40000 14.93000 -8.45000 L

H -1 21.96000 14.45000 -7.69000 L

H -1 23.34000 14.62000 -8.55000 L

H -1 22.39000 15.92000 -8.27000 L

C -1 20.14000 15.02000 -9.60000 L

H -1 19.75000 14.56000 -8.80000 L

H -1 20.05000 16.01000 -9.48000 L

H -1 19.65000 14.75000 -10.42000 L

C -1 23.70000 14.96000 -11.09000 L

O -1 24.01000 13.75000 -11.19000 L

N -1 24.58000 15.97000 -11.21000 L

H -1 24.28000 16.92000 -11.19000 L

C -1 26.02000 15.62000 -11.37000 L

H -1 26.38000 16.16000 -12.13000 L

H -1 26.07000 14.64000 -11.59000 L

C -1 26.85000 15.88000 -10.14000 L

O -1 26.42000 16.58000 -9.22000 L

N -1 28.09000 15.37000 -10.13000 L

H -1 28.38000 14.77000 -10.88000 L

C -1 29.03000 15.69000 -9.06000 L

H -1 28.51000 15.87000 -8.22000 L

C -1 29.97000 14.50000 -8.73000 L

H -1 29.36000 13.80000 -8.34000 L

C -1 30.72000 13.91000 -9.99000 L

H -1 31.30000 13.15000 -9.71000 L

H -1 30.05000 13.58000 -10.66000 L

H -1 31.28000 14.62000 -10.41000 L

C -1 31.00000 14.86000 -7.68000 L

H -1 31.46000 15.69000 -8.00000 L

H -1 31.66000 14.11000 -7.65000 L

C -1 30.37000 15.11000 -6.19000 L

H -1 31.10000 15.34000 -5.55000 L

H -1 29.71000 15.86000 -6.23000 L

H -1 29.91000 14.28000 -5.88000 L

C -1 29.78000 16.94000 -9.54000 L

O -1 30.31000 16.96000 -10.65000 L

N -1 29.76000 17.99000 -8.73000 L

H -1 29.27000 17.95000 -7.86000 L

C -1 30.47000 19.25000 -9.11000 L

H -1 30.14000 19.48000 -10.03000 L

C -1 30.13000 20.35000 -8.11000 L

H -1 30.61000 21.19000 -8.37000 L

H -1 29.15000 20.51000 -8.11000 L

H -1 30.42000 20.07000 -7.20000 L

C -1 32.01000 19.04000 -9.11000 L

O -1 32.56000 18.16000 -8.39000 L

N -1 32.69000 19.90000 -9.85000 L

H -1 32.20000 20.53000 -10.45000 L

C -1 34.15000 19.94000 -9.79000 L

H -1 34.44000 19.03000 -10.09000 L

C -1 34.68000 21.09000 -10.71000 L

H -1 35.68000 21.12000 -10.68000 L

H -1 34.39000 20.93000 -11.66000 L

H -1 34.32000 21.97000 -10.40000 L

C -1 34.64000 20.14000 -8.39000 L

O -1 33.96000 20.79000 -7.56000 L

N -1 35.86000 19.63000 -8.13000 L

H -1 36.32000 19.08000 -8.83000 L

C -1 36.52000 19.86000 -6.81000 L

H -1 36.00000 20.54000 -6.29000 L

H -1 37.45000 20.19000 -6.96000 L

C 0 36.54000 18.52000 -6.07000 L H 204

O -1 37.44000 18.27000 -5.24000 L

N 0 36.54000 15.32000 -7.33000 L H 222

H -1 35.80000 15.76000 -7.83000 L

C -1 37.52000 14.45000 -8.03000 L

H -1 38.07000 13.98000 -7.35000 L

C -1 36.77000 13.47000 -8.89000 L

H -1 36.35000 13.99000 -9.63000 L

H -1 36.05000 13.07000 -8.32000 L

C -1 37.71000 12.32000 -9.49000 L

O -1 38.89000 12.37000 -9.21000 L

O -1 37.20000 11.41000 -10.19000 L

C -1 38.38000 15.33000 -8.96000 L

O -1 38.11000 15.41000 -10.16000 L

N -1 39.36000 16.02000 -8.39000 L

H -1 39.56000 15.86000 -7.42000 L

C -1 40.17000 17.00000 -9.13000 L

H -1 39.52000 17.60000 -9.60000 L

C -1 41.10000 17.76000 -8.14000 L

H -1 41.88000 18.11000 -8.66000 L

H -1 41.42000 17.10000 -7.46000 L

C -1 40.32000 18.97000 -7.41000 L

H -1 39.40000 18.69000 -7.14000 L

H -1 40.26000 19.77000 -8.01000 L

C -1 41.07000 19.40000 -6.15000 L

H -1 42.00000 19.66000 -6.40000 L

H -1 41.09000 18.63000 -5.52000 L

C -1 40.33000 20.62000 -5.47000 L

H -1 40.74000 20.84000 -4.59000 L

H -1 39.36000 20.43000 -5.35000 L

N -1 40.51000 21.81000 -6.45000 L

H -1 40.06000 22.62000 -6.07000 L

H -1 41.49000 21.99000 -6.57000 L

H -1 40.10000 21.58000 -7.33000 L

C -1 41.03000 16.32000 -10.16000 L

O -1 41.34000 16.96000 -11.17000 L

N -1 41.44000 15.06000 -9.92000 L

H -1 41.04000 14.53000 -9.17000 L

C -1 42.51000 14.49000 -10.79000 L

H -1 42.77000 15.22000 -11.42000 L

C -1 43.78000 14.05000 -9.96000 L

H -1 44.58000 14.07000 -10.55000 L

H -1 43.64000 13.11000 -9.62000 L

C -1 44.08000 14.91000 -8.75000 L

N -1 44.38000 16.27000 -8.85000 L

C -1 44.58000 16.76000 -7.63000 L

H -1 44.88000 17.68000 -7.41000 L

N -1 44.30000 15.79000 -6.74000 L

H -1 44.31000 15.90000 -5.75000 L

C -1 44.01000 14.64000 -7.42000 L

H -1 43.79000 13.75000 -7.00000 L

C -1 41.93000 13.29000 -11.56000 L

O -1 42.67000 12.52000 -12.14000 L

N -1 40.61000 13.17000 -11.69000 L

H -1 39.99000 13.81000 -11.21000 L

C -1 40.06000 12.11000 -12.53000 L

H -1 40.49000 12.19000 -13.43000 L

H -1 39.08000 12.26000 -12.62000 L

C -1 40.31000 10.71000 -11.98000 L

O -1 40.44000 9.75000 -12.76000 L

N -1 40.28000 10.55000 -10.66000 L

H -1 40.12000 11.34000 -10.07000 L

C -1 40.50000 9.20000 -10.02000 L

H -1 40.94000 8.71000 -10.76000 L

C -1 41.31000 9.37000 -8.69000 L

H -1 41.54000 8.48000 -8.30000 L

H -1 40.80000 9.91000 -8.02000 L

C -1 42.56000 10.06000 -8.96000 L

H -1 42.34000 10.87000 -9.51000 L

H -1 43.14000 9.45000 -9.49000 L

C -1 43.26000 10.48000 -7.69000 L

O -1 42.86000 11.44000 -6.99000 L

O -1 44.25000 9.82000 -7.38000 L

C -1 39.30000 8.35000 -9.71000 L

O -1 39.44000 7.17000 -9.47000 L

N -1 38.09000 8.88000 -9.73000 L

H -1 37.92000 9.77000 -10.17000 L

C -1 36.99000 8.14000 -9.09000 L

H -1 37.31000 7.20000 -8.99000 L

C -1 36.63000 8.79000 -7.67000 L

H -1 35.88000 8.27000 -7.25000 L

H -1 37.43000 8.76000 -7.08000 L

H -1 36.34000 9.74000 -7.81000 L

C -1 35.79000 8.17000 -9.99000 L

O -1 34.66000 8.02000 -9.54000 L

N -1 36.02000 8.39000 -11.30000 L

H -1 36.95000 8.36000 -11.65000 L

C -1 34.92000 8.69000 -12.21000 L

H -1 34.51000 9.53000 -11.85000 L

C -1 35.41000 8.90000 -13.67000 L

H -1 35.94000 8.10000 -13.96000 L

C -1 34.19000 9.06000 -14.64000 L

H -1 34.53000 9.19000 -15.58000 L

H -1 33.62000 8.24000 -14.61000 L

H -1 33.65000 9.85000 -14.37000 L

C -1 36.35000 10.09000 -13.74000 L

H -1 36.65000 10.21000 -14.69000 L

H -1 35.87000 10.91000 -13.44000 L

H -1 37.14000 9.92000 -13.15000 L

C -1 33.92000 7.55000 -12.19000 L

O -1 32.72000 7.74000 -12.04000 L

N -1 34.41000 6.31000 -12.40000 L

H -1 35.39000 6.16000 -12.53000 L

C -1 33.48000 5.20000 -12.42000 L

H -1 32.82000 5.49000 -13.12000 L

C -1 34.24000 3.88000 -12.79000 L

H -1 33.63000 3.09000 -12.67000 L

H -1 35.04000 3.77000 -12.20000 L

C -1 34.70000 3.92000 -14.25000 L

O -1 35.89000 4.01000 -14.46000 L

O -1 33.88000 4.06000 -15.15000 L

C -1 32.72000 4.99000 -11.09000 L

O -1 31.55000 4.67000 -11.12000 L

N -1 33.39000 5.20000 -9.95000 L

H -1 34.34000 5.49000 -9.98000 L

C -1 32.70000 5.01000 -8.65000 L

H -1 33.38000 5.02000 -7.92000 L

H -1 32.24000 4.12000 -8.66000 L

C -1 31.70000 6.11000 -8.46000 L

O -1 30.69000 5.88000 -7.82000 L

N -1 31.92000 7.30000 -9.04000 L

H -1 32.80000 7.48000 -9.49000 L

C -1 30.86000 8.39000 -9.01000 L

H -1 30.51000 8.47000 -8.08000 L

C -1 31.48000 9.76000 -9.45000 L

H -1 30.76000 10.45000 -9.53000 L

H -1 31.95000 9.66000 -10.32000 L

C -1 32.49000 10.19000 -8.37000 L

H -1 32.99000 9.39000 -8.03000 L

C -1 33.50000 11.23000 -8.95000 L

H -1 34.15000 11.50000 -8.24000 L

H -1 33.99000 10.82000 -9.72000 L

H -1 33.00000 12.04000 -9.27000 L

C -1 31.67000 10.85000 -7.24000 L

H -1 32.29000 11.15000 -6.51000 L

H -1 31.18000 11.65000 -7.60000 L

H -1 31.01000 10.20000 -6.87000 L

C -1 29.66000 8.02000 -9.86000 L

O -1 28.55000 8.20000 -9.42000 L

N -1 29.83000 7.42000 -11.04000 L

H -1 30.74000 7.28000 -11.44000 L

C -1 28.59000 6.96000 -11.71000 L

H -1 28.06000 7.80000 -11.83000 L

C -1 28.87000 6.27000 -13.06000 L

H -1 28.03000 5.84000 -13.39000 L

H -1 29.58000 5.58000 -12.94000 L

C -1 29.34000 7.30000 -14.09000 L

C -1 30.57000 7.17000 -14.69000 L

H -1 31.14000 6.37000 -14.48000 L

C -1 31.05000 8.13000 -15.60000 L

H -1 31.96000 8.02000 -16.02000 L

C -1 30.27000 9.24000 -15.91000 L

O -1 30.77000 10.19000 -16.79000 L

H -1 30.09000 10.92000 -16.91000 L

C -1 29.03000 9.43000 -15.31000 L

H -1 28.48000 10.24000 -15.52000 L

C -1 28.56000 8.43000 -14.37000 L

H -1 27.67000 8.55000 -13.92000 L

C -1 27.91000 5.89000 -10.84000 L

O -1 26.65000 5.83000 -10.79000 L

N -1 28.72000 4.97000 -10.27000 L

H -1 29.71000 4.98000 -10.41000 L

C -1 28.08000 3.96000 -9.43000 L

H -1 27.44000 3.49000 -10.03000 L

C -1 29.19000 3.04000 -8.89000 L

H -1 29.85000 3.59000 -8.38000 L

H -1 29.64000 2.60000 -9.66000 L

C -1 28.66000 1.97000 -7.97000 L

H -1 27.94000 1.46000 -8.44000 L

H -1 28.29000 2.39000 -7.15000 L

C -1 29.78000 1.01000 -7.58000 L

H -1 30.30000 1.40000 -6.82000 L

H -1 30.39000 0.87000 -8.37000 L

C -1 29.21000 -0.34000 -7.15000 L

H -1 28.88000 -0.84000 -7.96000 L

H -1 28.45000 -0.20000 -6.51000 L

N -1 30.33000 -1.14000 -6.47000 L

H -1 29.98000 -2.03000 -6.18000 L

H -1 31.09000 -1.27000 -7.12000 L

H -1 30.67000 -0.63000 -5.68000 L

C -1 27.29000 4.58000 -8.22000 L

O -1 26.29000 3.98000 -7.75000 L

N -1 27.73000 5.75000 -7.70000 L

H -1 28.60000 6.12000 -8.03000 L

C -1 26.97000 6.49000 -6.66000 L

H -1 26.82000 5.85000 -5.91000 L

C -1 27.79000 7.71000 -6.25000 L

H -1 27.53000 8.49000 -6.82000 L

H -1 28.76000 7.52000 -6.40000 L

C -1 27.61000 8.11000 -4.82000 L

H -1 27.63000 7.24000 -4.32000 L

H -1 26.69000 8.48000 -4.79000 L

S -1 28.83000 9.26000 -4.20000 L

C -1 30.27000 8.37000 -4.25000 L

H -1 31.02000 8.93000 -3.92000 L

H -1 30.18000 7.56000 -3.67000 L

H -1 30.45000 8.08000 -5.19000 L

C -1 25.61000 7.00000 -7.18000 L

O -1 24.72000 7.37000 -6.39000 L

N -1 25.43000 7.06000 -8.49000 L

H -1 26.17000 6.86000 -9.12000 L

C -1 24.06000 7.43000 -8.98000 L

H -1 23.45000 7.49000 -8.19000 L

H -1 23.74000 6.71000 -9.59000 L

C -1 24.08000 8.78000 -9.72000 L

O -1 23.04000 9.23000 -10.16000 L

N -1 25.26000 9.43000 -9.86000 L

H -1 26.09000 9.03000 -9.46000 L

C -1 25.35000 10.71000 -10.58000 L

H -1 24.64000 11.30000 -10.19000 L

C -1 26.72000 11.40000 -10.42000 L

H -1 26.78000 12.16000 -11.07000 L

H -1 27.44000 10.73000 -10.62000 L

C -1 26.92000 11.93000 -9.02000 L

C -1 27.81000 11.30000 -8.13000 L

H -1 28.30000 10.48000 -8.43000 L

C -1 28.01000 11.82000 -6.80000 L

H -1 28.66000 11.39000 -6.17000 L

C -1 27.27000 12.96000 -6.40000 L

H -1 27.42000 13.35000 -5.49000 L

C -1 26.32000 13.57000 -7.25000 L

H -1 25.77000 14.35000 -6.95000 L

C -1 26.15000 13.02000 -8.59000 L

H -1 25.48000 13.43000 -9.21000 L

C -1 25.07000 10.47000 -12.02000 L

O -1 25.54000 9.48000 -12.60000 L

N -1 24.31000 11.36000 -12.62000 L

H -1 23.96000 12.13000 -12.09000 L

C -1 23.95000 11.27000 -14.04000 L

H -1 23.13000 11.82000 -14.19000 L

H -1 23.76000 10.32000 -14.26000 L

C -1 25.07000 11.77000 -14.91000 L

O -1 25.07000 11.53000 -16.14000 L

N -1 26.03000 12.49000 -14.31000 L

H -1 25.97000 12.71000 -13.34000 L

C -1 27.18000 12.94000 -15.12000 L

H -1 27.42000 12.15000 -15.69000 L

C -1 26.92000 14.10000 -16.10000 L

H -1 26.22000 13.81000 -16.74000 L

H -1 27.77000 14.29000 -16.59000 L

C -1 26.46000 15.43000 -15.46000 L

C -1 27.28000 16.55000 -15.46000 L

H -1 28.21000 16.47000 -15.83000 L

C -1 26.85000 17.78000 -14.95000 L

H -1 27.48000 18.56000 -14.94000 L

C -1 25.56000 17.91000 -14.46000 L

H -1 25.24000 18.81000 -14.14000 L

C -1 24.71000 16.82000 -14.40000 L

H -1 23.81000 16.90000 -13.97000 L

C -1 25.12000 15.56000 -14.97000 L

H -1 24.48000 14.79000 -15.01000 L

C -1 28.26000 13.32000 -14.13000 L

O -1 27.95000 13.58000 -12.97000 L

N -1 29.51000 13.19000 -14.57000 L

H -1 29.68000 12.91000 -15.52000 L

C -1 30.63000 13.46000 -13.67000 L

H -1 30.26000 13.75000 -12.79000 L

C -1 31.49000 12.18000 -13.54000 L

H -1 31.70000 11.80000 -14.44000 L

C -1 32.87000 12.52000 -12.78000 L

H -1 33.42000 11.68000 -12.69000 L

H -1 33.38000 13.20000 -13.30000 L

H -1 32.67000 12.88000 -11.87000 L

C -1 30.66000 11.15000 -12.75000 L

H -1 31.19000 10.31000 -12.64000 L

H -1 30.44000 11.52000 -11.85000 L

H -1 29.82000 10.95000 -13.25000 L

C -1 31.46000 14.57000 -14.22000 L

O -1 31.78000 14.54000 -15.41000 L

N -1 31.81000 15.56000 -13.38000 L

H -1 31.42000 15.63000 -12.46000 L

C -1 32.79000 16.55000 -13.86000 L

H -1 32.95000 16.39000 -14.84000 L

C -1 32.23000 17.95000 -13.62000 L

H -1 32.12000 18.06000 -12.63000 L

H -1 31.33000 17.99000 -14.06000 L

C -1 33.09000 19.13000 -14.17000 L

H -1 33.34000 18.95000 -15.12000 L

H -1 33.93000 19.21000 -13.62000 L

C -1 32.36000 20.46000 -14.12000 L

O -1 32.96000 21.40000 -13.55000 L

O -1 31.18000 20.52000 -14.62000 L

C -1 34.06000 16.38000 -13.02000 L

O -1 33.98000 16.36000 -11.78000 L

N -1 35.23000 16.26000 -13.66000 L

H -1 35.25000 16.19000 -14.65000 L

C -1 36.48000 16.23000 -12.91000 L

H -1 36.15000 16.07000 -11.98000 L

C -1 37.43000 15.12000 -13.39000 L

H -1 38.25000 15.11000 -12.82000 L

C -1 36.66000 13.74000 -13.32000 L

H -1 37.26000 13.01000 -13.63000 L

H -1 36.37000 13.56000 -12.38000 L

H -1 35.85000 13.78000 -13.92000 L

C -1 37.80000 15.38000 -14.87000 L

H -1 37.87000 16.37000 -15.01000 L

H -1 37.08000 15.01000 -15.46000 L

C -1 39.11000 14.75000 -15.28000 L

H -1 39.30000 14.96000 -16.24000 L

H -1 39.85000 15.12000 -14.71000 L

H -1 39.06000 13.76000 -15.15000 L

C -1 37.21000 17.53000 -12.99000 L

O -1 36.89000 18.35000 -13.84000 L

N -1 38.24000 17.72000 -12.16000 L

H -1 38.49000 17.00000 -11.51000 L

C -1 39.00000 18.99000 -12.19000 L

H -1 38.76000 19.48000 -13.02000 L

H -1 39.98000 18.77000 -12.20000 L

C -1 38.67000 19.85000 -10.95000 L

O -1 37.92000 19.41000 -10.05000 L

N -1 39.21000 21.09000 -10.90000 L

H -1 39.12000 21.62000 -10.06000 L

C -1 39.94000 21.68000 -12.05000 L

H -1 39.37000 21.46000 -12.84000 L

C -1 40.12000 23.20000 -11.79000 L

H -1 40.70000 23.61000 -12.50000 L

H -1 40.52000 23.37000 -10.89000 L

O -1 38.85000 23.88000 -11.84000 L

H -1 38.99000 24.86000 -11.67000 L

C -1 41.34000 21.08000 -12.23000 L

O -1 42.00000 20.71000 -11.26000 L

N -1 41.75000 20.93000 -13.48000 L

H -1 41.12000 21.17000 -14.23000 L

C -1 43.06000 20.44000 -13.81000 L

H -1 43.40000 20.08000 -12.94000 L

C -1 42.98000 19.35000 -14.90000 L

H -1 42.72000 19.80000 -15.76000 L

C -1 44.42000 18.65000 -15.09000 L

H -1 44.36000 17.94000 -15.80000 L

H -1 45.09000 19.33000 -15.36000 L

H -1 44.70000 18.22000 -14.23000 L

C -1 41.83000 18.31000 -14.56000 L

H -1 41.79000 17.61000 -15.27000 L

H -1 42.03000 17.87000 -13.68000 L

H -1 40.95000 18.79000 -14.50000 L

C -1 43.93000 21.58000 -14.38000 L

O -1 43.47000 22.35000 -15.24000 L

N -1 45.18000 21.71000 -13.91000 L

H -1 45.53000 21.06000 -13.24000 L

C -1 46.02000 22.80000 -14.38000 L

H -1 45.39000 23.45000 -14.80000 L

C -1 46.80000 23.48000 -13.22000 L

H -1 47.44000 24.16000 -13.58000 L

C -1 45.84000 24.11000 -12.20000 L

H -1 46.37000 24.54000 -11.47000 L

H -1 45.28000 24.80000 -12.66000 L

H -1 45.25000 23.40000 -11.82000 L

O -1 47.55000 22.49000 -12.49000 L

H -1 48.05000 22.93000 -11.75000 L

C -1 47.06000 22.17000 -15.37000 L

O -1 47.27000 20.94000 -15.38000 L

N -1 47.68000 23.00000 -16.20000 L

C -1 47.33000 24.41000 -16.54000 L

H -1 47.81000 25.04000 -15.93000 L

H -1 46.34000 24.55000 -16.45000 L

C -1 47.82000 24.54000 -18.06000 L

H -1 48.06000 25.48000 -18.29000 L

H -1 47.11000 24.21000 -18.70000 L

C -1 49.11000 23.59000 -18.08000 L

H -1 49.90000 24.02000 -17.66000 L

H -1 49.34000 23.29000 -19.01000 L

C -1 48.62000 22.39000 -17.22000 L

H -1 48.14000 21.70000 -17.77000 L

C -1 49.77000 21.59000 -16.59000 L

O -1 49.89000 20.41000 -16.87000 L

N -1 50.53000 22.20000 -15.67000 L

H -1 50.35000 23.16000 -15.46000 L

C -1 51.59000 21.52000 -14.97000 L

H -1 51.84000 20.74000 -15.54000 L

C -1 52.83000 22.46000 -14.76000 L

H -1 53.57000 21.92000 -14.38000 L

H -1 52.57000 23.17000 -14.10000 L

C -1 53.34000 23.17000 -16.05000 L

H -1 54.24000 23.58000 -15.88000 L

H -1 52.69000 23.89000 -16.32000 L

C -1 53.47000 22.14000 -17.22000 L

H -1 52.60000 21.66000 -17.29000 L

H -1 54.19000 21.49000 -16.96000 L

C -1 53.84000 22.79000 -18.67000 L

H -1 54.71000 22.41000 -19.00000 L

H -1 53.92000 23.78000 -18.59000 L

N -1 52.75000 22.48000 -19.75000 L

H -1 53.02000 22.90000 -20.62000 L

H -1 52.66000 21.49000 -19.86000 L

H -1 51.87000 22.86000 -19.46000 L

C -1 51.03000 21.13000 -13.61000 L

O -1 50.12000 21.80000 -13.09000 L

N -1 51.58000 20.06000 -13.03000 L

C -1 52.66000 19.21000 -13.63000 L

H -1 53.51000 19.73000 -13.71000 L

H -1 52.38000 18.89000 -14.54000 L

C -1 52.82000 18.05000 -12.67000 L

H -1 53.79000 17.82000 -12.58000 L

H -1 52.32000 17.26000 -13.01000 L

C -1 52.22000 18.58000 -11.28000 L

H -1 52.95000 18.92000 -10.68000 L

H -1 51.72000 17.85000 -10.81000 L

C -1 51.25000 19.74000 -11.64000 L

H -1 50.28000 19.53000 -11.58000 L

C -1 51.55000 20.92000 -10.69000 L

O -1 52.50000 21.68000 -10.88000 L

N -1 50.77000 21.05000 -9.65000 L

H -1 49.94000 20.50000 -9.56000 L

C -1 51.14000 22.02000 -8.62000 L

H -1 52.13000 21.94000 -8.49000 L

C -1 50.82000 23.45000 -9.06000 L

H -1 51.30000 23.63000 -9.93000 L

H -1 51.17000 24.08000 -8.36000 L

C -1 49.26000 23.67000 -9.26000 L

H -1 48.79000 23.39000 -8.42000 L

H -1 48.95000 23.12000 -10.03000 L

C -1 48.88000 25.14000 -9.54000 L

O -1 49.57000 25.83000 -10.25000 L

N -1 47.73000 25.60000 -9.01000 L

H -1 47.17000 24.99000 -8.45000 L

H -1 47.44000 26.54000 -9.17000 L

C -1 50.42000 21.67000 -7.32000 L

O -1 49.46000 20.91000 -7.31000 L

N -1 51.01000 22.08000 -6.22000 L

H -1 51.75000 22.75000 -6.26000 L

C -1 50.56000 21.54000 -4.95000 L

H -1 50.33000 20.61000 -5.21000 L

C -1 51.68000 21.66000 -3.88000 L

H -1 51.29000 21.58000 -2.97000 L

H -1 52.14000 22.55000 -3.97000 L

C -1 52.73000 20.56000 -4.06000 L

H -1 53.09000 20.61000 -4.99000 L

H -1 52.28000 19.67000 -3.93000 L

C -1 53.88000 20.70000 -3.05000 L

O -1 53.94000 21.74000 -2.31000 L

O -1 54.71000 19.76000 -3.00000 L

C -1 49.33000 22.26000 -4.35000 L

O -1 48.69000 21.67000 -3.47000 L

N -1 49.08000 23.47000 -4.77000 L

H -1 49.64000 23.85000 -5.51000 L

C -1 47.97000 24.32000 -4.19000 L

H -1 47.17000 23.74000 -4.01000 L

H -1 47.72000 25.03000 -4.85000 L

C -1 48.45000 24.97000 -2.88000 L

O -1 49.70000 25.03000 -2.61000 L

N -1 47.53000 25.53000 -2.07000 L

H -1 46.56000 25.46000 -2.31000 L

C -1 47.94000 26.27000 -0.82000 L

H -1 48.75000 26.81000 -1.07000 L

C -1 46.77000 27.15000 -0.31000 L

H -1 46.98000 27.51000 0.60000 L

H -1 45.93000 26.61000 -0.27000 L

C -1 46.53000 28.28000 -1.20000 L

O -1 47.50000 28.90000 -1.59000 L

N -1 45.27000 28.55000 -1.64000 L

H -1 44.51000 27.96000 -1.35000 L

H -1 45.10000 29.32000 -2.25000 L

C -1 48.39000 25.37000 0.27000 L

O -1 48.06000 24.21000 0.30000 L

N -1 49.12000 25.92000 1.26000 L

C -1 49.58000 27.30000 1.31000 L

H -1 48.80000 27.92000 1.25000 L

H -1 50.21000 27.49000 0.56000 L

C -1 50.27000 27.39000 2.69000 L

H -1 49.76000 28.00000 3.30000 L

H -1 51.21000 27.73000 2.59000 L

C -1 50.33000 26.03000 3.32000 L

H -1 49.97000 26.08000 4.25000 L

H -1 51.28000 25.72000 3.34000 L

C -1 49.47000 25.08000 2.46000 L

H -1 49.90000 24.22000 2.17000 L

C -1 48.27000 24.66000 3.26000 L

O -1 47.28000 25.40000 3.24000 L

N -1 48.37000 23.58000 4.04000 L

H -1 49.25000 23.10000 4.06000 L

C -1 47.30000 23.04000 4.84000 L

H -1 46.46000 23.31000 4.37000 L

C -1 47.44000 21.56000 4.93000 L

H -1 46.74000 21.19000 5.54000 L

H -1 48.34000 21.33000 5.30000 L

C -1 47.30000 20.93000 3.66000 L

H -1 48.07000 21.18000 3.07000 L

H -1 46.44000 21.20000 3.23000 L

C -1 47.31000 19.48000 3.95000 L

H -1 46.44000 19.20000 4.34000 L

H -1 48.05000 19.26000 4.58000 L

N -1 47.51000 18.77000 2.73000 L

H -1 47.92000 19.26000 1.96000 L

C -1 47.20000 17.48000 2.55000 L

N -1 46.62000 16.75000 3.55000 L

H -1 46.43000 17.17000 4.44000 L

H -1 46.39000 15.79000 3.39000 L

N -1 47.45000 16.94000 1.35000 L

H -1 47.86000 17.50000 0.63000 L

H -1 47.22000 15.98000 1.18000 L

C -1 47.36000 23.58000 6.26000 L

O -1 48.45000 23.87000 6.75000 L

N -1 46.22000 23.76000 6.92000 L

C -1 46.27000 24.13000 8.36000 L

H -1 46.86000 24.92000 8.52000 L

H -1 46.60000 23.36000 8.92000 L

C -1 44.81000 24.44000 8.63000 L

H -1 44.55000 25.34000 8.30000 L

H -1 44.58000 24.36000 9.61000 L

C -1 44.14000 23.38000 7.86000 L

H -1 43.16000 23.57000 7.77000 L

H -1 44.26000 22.49000 8.29000 L

C -1 44.87000 23.48000 6.50000 L

H -1 44.87000 22.63000 5.97000 L

C -1 44.23000 24.47000 5.49000 L

O -1 44.56000 25.66000 5.42000 L

N -1 43.44000 23.92000 4.55000 L

H -1 43.21000 22.95000 4.59000 L

C -1 42.93000 24.77000 3.48000 L

H -1 42.91000 25.72000 3.79000 L

C -1 43.89000 24.61000 2.22000 L

H -1 44.79000 24.97000 2.44000 L

H -1 43.50000 25.11000 1.45000 L

C -1 44.04000 23.10000 1.81000 L

H -1 43.20000 22.80000 1.35000 L

H -1 44.18000 22.55000 2.63000 L

C -1 45.30000 22.96000 0.82000 L

H -1 46.14000 23.30000 1.25000 L

H -1 45.15000 23.44000 -0.04000 L

N -1 45.51000 21.53000 0.52000 L

H -1 45.05000 20.84000 1.07000 L

C -1 46.29000 21.15000 -0.49000 L

N -1 46.37000 19.88000 -0.82000 L

H -1 45.85000 19.20000 -0.32000 L

H -1 46.96000 19.60000 -1.58000 L

N -1 46.97000 22.07000 -1.14000 L

H -1 46.89000 23.03000 -0.87000 L

H -1 47.57000 21.82000 -1.90000 L

C 0 41.49000 24.36000 3.18000 L H 224

O -1 40.93000 24.76000 2.17000 L

N 0 37.74000 23.77000 5.47000 L H 238

H -1 37.23000 24.06000 4.67000 L

C -1 37.18000 24.00000 6.81000 L

H -1 37.65000 23.38000 7.44000 L

C -1 37.38000 25.48000 7.24000 L

H -1 37.10000 25.60000 8.19000 L

H -1 36.84000 26.08000 6.65000 L

C -1 38.81000 25.90000 7.13000 L

C -1 39.29000 26.50000 5.98000 L

H -1 38.66000 26.72000 5.24000 L

C -1 40.64000 26.79000 5.87000 L

H -1 40.99000 27.19000 5.02000 L

C -1 41.53000 26.52000 6.93000 L

H -1 42.49000 26.76000 6.84000 L

C -1 41.09000 25.94000 8.08000 L

H -1 41.73000 25.74000 8.82000 L

C -1 39.71000 25.63000 8.20000 L

H -1 39.37000 25.22000 9.05000 L

C 0 35.69000 23.69000 6.77000 L H 240

O -1 34.97000 24.12000 5.85000 L

N 0 31.76000 24.01000 8.35000 L H 262

H -1 31.37000 23.63000 7.51000 L

C -1 30.95000 24.92000 9.16000 L

H -1 31.45000 25.16000 9.99000 L

C -1 30.61000 26.26000 8.43000 L

H -1 29.81000 26.66000 8.89000 L

H -1 30.36000 26.03000 7.49000 L

C -1 31.70000 27.38000 8.36000 L

H -1 31.98000 27.60000 9.29000 L

C -1 32.97000 26.85000 7.59000 L

H -1 33.66000 27.58000 7.55000 L

H -1 33.34000 26.06000 8.07000 L

H -1 32.71000 26.59000 6.66000 L

C -1 31.09000 28.68000 7.66000 L

H -1 31.79000 29.40000 7.62000 L

H -1 30.78000 28.46000 6.74000 L

H -1 30.31000 29.01000 8.20000 L

C -1 29.69000 24.10000 9.40000 L

O -1 28.65000 24.35000 8.75000 L

N -1 29.75000 23.13000 10.32000 L

C -1 30.93000 22.69000 11.12000 L

H -1 31.35000 23.47000 11.59000 L

H -1 31.62000 22.25000 10.54000 L

C -1 30.34000 21.70000 12.11000 L

H -1 30.14000 22.18000 12.97000 L

H -1 31.00000 20.97000 12.28000 L

C -1 28.99000 21.14000 11.41000 L

H -1 28.25000 21.01000 12.08000 L

H -1 29.16000 20.27000 10.93000 L

C -1 28.57000 22.22000 10.38000 L

H -1 28.40000 21.86000 9.46000 L

C -1 27.23000 22.96000 10.81000 L

O -1 26.18000 22.63000 10.35000 L

N -1 27.31000 23.98000 11.63000 L

H -1 28.20000 24.28000 11.96000 L

C -1 26.06000 24.71000 12.07000 L

H -1 25.42000 24.06000 12.47000 L

C -1 26.47000 25.79000 13.06000 L

H -1 25.67000 26.37000 13.22000 L

H -1 27.19000 26.33000 12.64000 L

C -1 26.97000 25.22000 14.44000 L

H -1 26.49000 24.36000 14.61000 L

H -1 26.74000 25.88000 15.15000 L

C -1 28.53000 24.95000 14.50000 L

O -1 28.90000 24.58000 15.63000 L

O -1 29.34000 25.04000 13.45000 L

C -1 25.43000 25.40000 10.87000 L

O -1 24.30000 25.89000 10.99000 L

N -1 26.19000 25.55000 9.74000 L

H -1 27.11000 25.18000 9.71000 L

C -1 25.64000 26.25000 8.59000 L

H -1 24.77000 26.61000 8.92000 L

C -1 26.57000 27.37000 8.14000 L

H -1 26.10000 27.92000 7.44000 L

H -1 27.40000 26.97000 7.74000 L

C -1 26.97000 28.29000 9.32000 L

O -1 26.06000 28.93000 9.86000 L

O -1 28.21000 28.44000 9.63000 L

C -1 25.43000 25.31000 7.40000 L

O -1 25.02000 25.75000 6.32000 L

N -1 25.79000 24.05000 7.58000 L

H -1 26.04000 23.76000 8.51000 L

C -1 25.87000 23.04000 6.50000 L

H -1 26.33000 22.23000 6.87000 L

C -1 24.43000 22.64000 6.02000 L

H -1 24.50000 22.03000 5.23000 L

H -1 23.93000 23.47000 5.76000 L

C -1 23.69000 21.90000 7.24000 L

H -1 23.73000 22.49000 8.05000 L

H -1 24.16000 21.04000 7.43000 L

C -1 22.20000 21.59000 6.93000 L

O -1 21.86000 21.10000 5.84000 L

N -1 21.32000 21.84000 7.91000 L

H -1 21.63000 22.21000 8.79000 L

H -1 20.34000 21.66000 7.77000 L

C -1 26.67000 23.65000 5.35000 L

O -1 26.25000 23.63000 4.19000 L

N -1 27.84000 24.17000 5.67000 L

H -1 28.19000 24.06000 6.60000 L

C -1 28.64000 24.89000 4.69000 L

H -1 28.27000 24.72000 3.78000 L

C -1 28.57000 26.40000 5.02000 L

H -1 29.11000 26.91000 4.35000 L

H -1 27.62000 26.71000 4.98000 L

H -1 28.93000 26.56000 5.94000 L

C 0 30.08000 24.37000 4.81000 L H 264

O -1 30.48000 23.78000 5.88000 L

N 0 34.24000 25.97000 3.77000 L H 278

H -1 34.61000 25.47000 4.56000 L

C -1 35.02000 27.01000 3.13000 L

H -1 34.47000 27.39000 2.39000 L

C -1 35.44000 28.11000 4.17000 L

H -1 35.80000 27.68000 5.00000 L

C -1 36.57000 29.09000 3.55000 L

H -1 36.82000 29.78000 4.23000 L

H -1 37.38000 28.55000 3.31000 L

H -1 36.22000 29.54000 2.73000 L

C -1 34.16000 28.91000 4.51000 L

H -1 33.46000 28.27000 4.81000 L

H -1 33.85000 29.38000 3.68000 L

C -1 34.43000 30.00000 5.66000 L

H -1 33.58000 30.49000 5.85000 L

H -1 34.73000 29.53000 6.49000 L

H -1 35.13000 30.64000 5.35000 L

C -1 36.32000 26.30000 2.68000 L

O -1 36.88000 25.49000 3.45000 L

N -1 36.71000 26.48000 1.42000 L

H -1 36.15000 27.00000 0.77000 L

C -1 37.98000 25.89000 1.01000 L

H -1 38.43000 25.60000 1.85000 L

C -1 37.73000 24.70000 0.06000 L

H -1 36.91000 24.20000 0.37000 L

H -1 38.52000 24.09000 0.08000 L

C -1 37.50000 25.18000 -1.38000 L

O -1 38.46000 25.36000 -2.16000 L

N -1 36.24000 25.44000 -1.73000 L

H -1 35.50000 25.32000 -1.07000 L

H -1 36.04000 25.76000 -2.66000 L

C -1 38.85000 26.90000 0.31000 L

O -1 38.34000 27.88000 -0.36000 L

N -1 40.18000 26.68000 0.43000 L

H -1 40.52000 25.95000 1.02000 L

C -1 41.11000 27.51000 -0.32000 L

H -1 40.59000 27.94000 -1.06000 L

C -1 41.69000 28.67000 0.52000 L

H -1 40.97000 29.34000 0.67000 L

H -1 42.43000 29.08000 -0.01000 L

C -1 42.27000 28.26000 1.92000 L

H -1 42.83000 27.44000 1.82000 L

H -1 41.52000 28.08000 2.55000 L

C -1 43.15000 29.43000 2.49000 L

H -1 42.61000 30.27000 2.56000 L

H -1 43.94000 29.59000 1.89000 L

N -1 43.66000 29.08000 3.84000 L

H -1 44.52000 28.58000 3.89000 L

C -1 43.05000 29.40000 4.99000 L

N -1 41.83000 29.96000 5.00000 L

H -1 41.36000 30.14000 4.13000 L

H -1 41.39000 30.18000 5.86000 L

N -1 43.62000 29.12000 6.16000 L

H -1 44.52000 28.68000 6.18000 L

H -1 43.16000 29.36000 7.01000 L

C 0 42.21000 26.56000 -0.80000 L H 280

O -1 43.42000 26.78000 -0.61000 L

N 0 43.39000 26.14000 -3.82000 L H 299

H -1 42.42000 26.36000 -3.83000 L

C -1 44.27000 26.85000 -4.75000 L

H -1 45.08000 27.14000 -4.23000 L

H -1 43.78000 27.65000 -5.08000 L

C -1 44.70000 25.95000 -5.94000 L

O -1 45.88000 25.96000 -6.36000 L

N -1 43.79000 25.13000 -6.46000 L

H -1 42.91000 25.04000 -6.00000 L

C -1 44.06000 24.36000 -7.69000 L

H -1 43.20000 23.87000 -7.87000 L

C -1 44.44000 25.28000 -8.88000 L

H -1 44.91000 24.72000 -9.56000 L

H -1 45.07000 25.98000 -8.53000 L

C -1 43.25000 26.02000 -9.58000 L

C -1 43.52000 27.13000 -10.42000 L

H -1 44.44000 27.51000 -10.47000 L

C -1 42.45000 27.73000 -11.21000 L

H -1 42.63000 28.46000 -11.88000 L

C -1 41.12000 27.22000 -10.99000 L

H -1 40.34000 27.68000 -11.43000 L

C -1 40.88000 26.13000 -10.20000 L

H -1 39.95000 25.78000 -10.11000 L

C -1 41.94000 25.50000 -9.51000 L

H -1 41.77000 24.67000 -8.98000 L

C -1 45.26000 23.41000 -7.45000 L

O -1 46.21000 23.38000 -8.26000 L

N -1 45.23000 22.66000 -6.37000 L

H -1 44.61000 22.89000 -5.62000 L

C -1 46.10000 21.50000 -6.27000 L

H -1 47.04000 21.83000 -6.32000 L

C -1 45.94000 20.81000 -4.93000 L

H -1 46.22000 21.43000 -4.20000 L

H -1 46.51000 19.99000 -4.91000 L

C -1 44.51000 20.40000 -4.67000 L

O -1 43.61000 21.21000 -4.76000 L

N -1 44.29000 19.14000 -4.35000 L

H -1 45.06000 18.50000 -4.30000 L

H -1 43.36000 18.82000 -4.18000 L

C -1 45.72000 20.55000 -7.43000 L

O -1 44.55000 20.19000 -7.62000 L

N -1 46.69000 20.17000 -8.24000 L

H -1 47.65000 20.40000 -8.04000 L

C -1 46.35000 19.39000 -9.45000 L

H -1 45.55000 18.84000 -9.21000 L

C -1 46.03000 20.36000 -10.61000 L

H -1 46.78000 21.01000 -10.76000 L

H -1 45.18000 20.87000 -10.44000 L

O -1 45.85000 19.56000 -11.81000 L

H -1 45.65000 20.16000 -12.58000 L

C -1 47.55000 18.47000 -9.85000 L

O -1 48.73000 18.91000 -9.82000 L

N -1 47.26000 17.21000 -10.17000 L

H -1 46.34000 16.87000 -9.98000 L

C -1 48.24000 16.32000 -10.79000 L

H -1 49.03000 16.30000 -10.17000 L

C -1 47.61000 14.95000 -10.91000 L

H -1 48.21000 14.37000 -11.46000 L

H -1 46.73000 15.04000 -11.37000 L

C -1 47.41000 14.31000 -9.57000 L

N -1 46.83000 13.06000 -9.41000 L

C -1 46.79000 12.78000 -8.12000 L

H -1 46.46000 11.92000 -7.73000 L

N -1 47.25000 13.82000 -7.44000 L

H -1 47.30000 13.88000 -6.44000 L

C -1 47.65000 14.79000 -8.32000 L

H -1 48.05000 15.68000 -8.09000 L

C -1 48.76000 16.82000 -12.16000 L

O -1 49.75000 16.27000 -12.69000 L

N -1 48.03000 17.77000 -12.76000 L

H -1 47.21000 18.13000 -12.32000 L

C -1 48.43000 18.30000 -14.06000 L

H -1 49.43000 18.23000 -14.12000 L

H -1 48.16000 19.26000 -14.09000 L

C -1 47.80000 17.56000 -15.23000 L

O -1 47.33000 16.42000 -15.12000 L

N -1 47.85000 18.19000 -16.41000 L

H -1 48.32000 19.07000 -16.49000 L

C -1 47.21000 17.60000 -17.58000 L

H -1 46.28000 17.47000 -17.24000 L

C -1 47.42000 18.55000 -18.82000 L

H -1 47.10000 18.08000 -19.64000 L

H -1 48.39000 18.76000 -18.91000 L

C -1 46.67000 19.90000 -18.75000 L

H -1 46.89000 20.36000 -17.89000 L

C -1 47.06000 20.85000 -19.92000 L

H -1 46.55000 21.71000 -19.84000 L

H -1 48.04000 21.03000 -19.89000 L

H -1 46.83000 20.41000 -20.79000 L

C -1 45.19000 19.62000 -18.77000 L

H -1 44.68000 20.48000 -18.72000 L

H -1 44.95000 19.14000 -19.62000 L

H -1 44.95000 19.05000 -17.99000 L

C -1 47.75000 16.22000 -17.99000 L

O -1 46.98000 15.35000 -18.35000 L

N -1 49.08000 16.05000 -17.98000 L

H -1 49.70000 16.77000 -17.66000 L

C -1 49.60000 14.73000 -18.50000 L

H -1 49.24000 14.64000 -19.43000 L

C -1 51.11000 14.71000 -18.57000 L

H -1 51.47000 13.82000 -18.83000 L

H -1 51.53000 15.00000 -17.71000 L

O -1 51.45000 15.64000 -19.55000 L

H -1 52.44000 15.67000 -19.65000 L

C -1 49.10000 13.54000 -17.68000 L

O -1 48.61000 12.50000 -18.21000 L

N -1 49.19000 13.68000 -16.37000 L

H -1 49.58000 14.51000 -15.97000 L

C -1 48.71000 12.63000 -15.52000 L

H -1 49.25000 11.86000 -15.86000 L

C -1 48.99000 12.92000 -14.00000 L

H -1 48.74000 13.85000 -13.77000 L

C -1 48.14000 11.94000 -13.12000 L

H -1 48.31000 12.12000 -12.16000 L

H -1 47.17000 12.09000 -13.32000 L

H -1 48.38000 11.00000 -13.34000 L

C -1 50.51000 12.78000 -13.71000 L

H -1 50.68000 12.96000 -12.74000 L

H -1 50.81000 11.85000 -13.93000 L

H -1 51.01000 13.44000 -14.26000 L

C -1 47.23000 12.35000 -15.73000 L

O -1 46.86000 11.20000 -15.88000 L

N -1 46.38000 13.39000 -15.72000 L

H -1 46.73000 14.32000 -15.62000 L

C -1 44.95000 13.17000 -15.86000 L

H -1 44.68000 12.51000 -15.16000 L

C -1 44.18000 14.53000 -15.67000 L

H -1 44.48000 15.25000 -16.30000 L

C -1 42.68000 14.31000 -16.01000 L

H -1 42.19000 15.18000 -15.89000 L

H -1 42.59000 14.01000 -16.96000 L

H -1 42.29000 13.62000 -15.40000 L

C -1 44.44000 14.97000 -14.18000 L

H -1 43.97000 15.83000 -14.00000 L

H -1 44.09000 14.27000 -13.56000 L

H -1 45.42000 15.09000 -14.03000 L

C -1 44.68000 12.64000 -17.26000 L

O -1 43.86000 11.75000 -17.43000 L

N -1 45.48000 13.10000 -18.25000 L

H -1 46.18000 13.78000 -18.06000 L

C -1 45.29000 12.57000 -19.62000 L

H -1 44.34000 12.80000 -19.84000 L

C -1 46.29000 13.25000 -20.59000 L

H -1 47.21000 13.10000 -20.22000 L

H -1 46.08000 14.22000 -20.60000 L

C -1 46.22000 12.67000 -22.12000 L

H -1 45.57000 13.20000 -22.66000 L

H -1 45.94000 11.71000 -22.11000 L

C -1 47.67000 12.79000 -22.75000 L

O -1 48.36000 13.72000 -22.39000 L

O -1 48.19000 11.91000 -23.43000 L

C -1 45.49000 11.03000 -19.68000 L

O -1 44.64000 10.29000 -20.26000 L

N -1 46.61000 10.56000 -19.09000 L

H -1 47.26000 11.17000 -18.63000 L

C -1 46.81000 9.11000 -19.14000 L

H -1 46.71000 8.83000 -20.09000 L

C -1 48.24000 8.81000 -18.65000 L

H -1 48.34000 7.82000 -18.50000 L

H -1 48.42000 9.30000 -17.80000 L

C -1 49.23000 9.24000 -19.70000 L

N -1 50.06000 10.35000 -19.56000 L

C -1 50.75000 10.53000 -20.68000 L

H -1 51.43000 11.25000 -20.84000 L

N -1 50.40000 9.59000 -21.54000 L

H -1 50.79000 9.47000 -22.46000 L

C -1 49.41000 8.80000 -20.98000 L

H -1 48.91000 8.06000 -21.42000 L

C -1 45.75000 8.35000 -18.36000 L

O -1 45.32000 7.29000 -18.72000 L

N -1 45.33000 8.91000 -17.24000 L

H -1 45.72000 9.78000 -16.94000 L

C -1 44.31000 8.28000 -16.43000 L

H -1 44.61000 7.34000 -16.27000 L

C -1 44.17000 9.14000 -15.10000 L

H -1 43.64000 9.97000 -15.29000 L

H -1 45.08000 9.39000 -14.78000 L

C -1 43.49000 8.48000 -13.93000 L

H -1 43.86000 7.55000 -13.85000 L

H -1 42.51000 8.44000 -14.13000 L

C -1 43.75000 9.30000 -12.60000 L

H -1 43.08000 9.04000 -11.90000 L

H -1 43.68000 10.28000 -12.78000 L

N -1 45.04000 9.12000 -11.98000 L

H -1 45.47000 8.21000 -12.04000 L

C -1 45.69000 10.07000 -11.35000 L

N -1 45.20000 11.35000 -11.33000 L

H -1 44.34000 11.56000 -11.79000 L

H -1 45.70000 12.07000 -10.85000 L

N -1 46.82000 9.80000 -10.75000 L

H -1 47.18000 8.87000 -10.77000 L

H -1 47.32000 10.52000 -10.27000 L

C -1 42.99000 8.16000 -17.21000 L

O -1 42.27000 7.14000 -17.16000 L

N -1 42.59000 9.20000 -17.92000 L

H -1 43.12000 10.05000 -17.92000 L

C -1 41.39000 9.08000 -18.70000 L

H -1 40.78000 8.64000 -18.04000 L

C -1 40.89000 10.47000 -19.19000 L

H -1 40.11000 10.32000 -19.80000 L

H -1 41.63000 10.91000 -19.70000 L

C -1 40.44000 11.41000 -17.99000 L

H -1 41.22000 11.64000 -17.41000 L

C -1 39.86000 12.74000 -18.59000 L

H -1 39.57000 13.34000 -17.85000 L

H -1 40.56000 13.19000 -19.14000 L

H -1 39.07000 12.52000 -19.17000 L

C -1 39.37000 10.67000 -17.08000 L

H -1 39.10000 11.27000 -16.33000 L

H -1 38.57000 10.44000 -17.63000 L

H -1 39.78000 9.83000 -16.71000 L

C -1 41.57000 8.19000 -19.93000 L

O -1 40.62000 7.48000 -20.34000 L

N -1 42.75000 8.23000 -20.54000 L

H -1 43.48000 8.85000 -20.23000 L

C -1 42.95000 7.32000 -21.71000 L

H -1 42.22000 7.55000 -22.35000 L

C -1 44.33000 7.55000 -22.33000 L

H -1 44.55000 6.73000 -22.86000 L

H -1 44.98000 7.66000 -21.58000 L

C -1 44.42000 8.83000 -23.28000 L

H -1 44.17000 9.65000 -22.77000 L

H -1 43.80000 8.72000 -24.06000 L

C -1 45.91000 8.96000 -23.80000 L

H -1 46.23000 8.10000 -24.20000 L

H -1 46.53000 9.24000 -23.06000 L

N -1 45.94000 9.98000 -24.84000 L

H -1 45.12000 10.53000 -25.00000 L

C -1 47.04000 10.22000 -25.57000 L

N -1 48.09000 9.49000 -25.29000 L

H -1 48.05000 8.81000 -24.56000 L

H -1 48.94000 9.62000 -25.80000 L

N -1 47.11000 11.23000 -26.45000 L

H -1 46.32000 11.84000 -26.58000 L

H -1 47.94000 11.37000 -26.98000 L

C -1 42.83000 5.86000 -21.32000 L

O -1 42.39000 5.04000 -22.15000 L

N -1 43.14000 5.53000 -20.07000 L

H -1 43.40000 6.23000 -19.42000 L

C -1 43.10000 4.10000 -19.65000 L

H -1 43.60000 3.60000 -20.36000 L

C -1 43.82000 3.91000 -18.24000 L

H -1 43.79000 2.95000 -17.97000 L

H -1 44.78000 4.20000 -18.30000 L

H -1 43.35000 4.47000 -17.55000 L

C -1 41.64000 3.58000 -19.64000 L

O -1 41.41000 2.36000 -19.67000 L

N -1 40.67000 4.50000 -19.66000 L

H -1 40.90000 5.47000 -19.68000 L

C -1 39.27000 4.07000 -19.64000 L

H -1 39.25000 3.10000 -19.87000 L

C -1 38.71000 4.36000 -18.21000 L

H -1 39.25000 3.85000 -17.55000 L

H -1 37.76000 4.05000 -18.18000 L

C -1 38.76000 5.88000 -17.83000 L

H -1 38.26000 6.40000 -18.53000 L

H -1 39.72000 6.17000 -17.81000 L

C -1 38.11000 6.16000 -16.41000 L

H -1 38.21000 7.13000 -16.17000 L

H -1 38.54000 5.60000 -15.71000 L

N -1 36.66000 5.86000 -16.39000 L

H -1 36.36000 5.03000 -15.92000 L

C -1 35.75000 6.64000 -16.96000 L

N -1 36.12000 7.82000 -17.58000 L

H -1 37.08000 8.09000 -17.59000 L

H -1 35.42000 8.40000 -18.00000 L

N -1 34.46000 6.33000 -16.82000 L

H -1 34.19000 5.53000 -16.29000 L

H -1 33.76000 6.91000 -17.24000 L

C -1 38.45000 4.77000 -20.71000 L

O -1 37.23000 4.95000 -20.61000 L

N -1 39.15000 5.27000 -21.75000 L

H -1 40.12000 5.05000 -21.84000 L

C -1 38.51000 6.14000 -22.76000 L

H -1 38.12000 6.91000 -22.25000 L

C -1 39.57000 6.68000 -23.80000 L

H -1 39.98000 5.90000 -24.27000 L

H -1 40.28000 7.18000 -23.31000 L

C -1 39.01000 7.62000 -24.88000 L

H -1 38.49000 8.35000 -24.43000 L

H -1 38.39000 7.10000 -25.47000 L

C -1 40.12000 8.25000 -25.74000 L

O -1 41.25000 7.77000 -25.75000 L

N -1 39.82000 9.38000 -26.39000 L

H -1 38.90000 9.78000 -26.30000 L

H -1 40.50000 9.83000 -26.96000 L

C -1 37.35000 5.40000 -23.44000 L

O -1 36.28000 5.98000 -23.73000 L

N -1 37.57000 4.12000 -23.80000 L

H -1 38.42000 3.65000 -23.56000 L

C -1 36.51000 3.45000 -24.56000 L

H -1 36.15000 4.09000 -25.24000 L

C -1 37.07000 2.17000 -25.31000 L

H -1 36.31000 1.68000 -25.75000 L

H -1 37.52000 1.57000 -24.66000 L

C -1 38.10000 2.63000 -26.39000 L

H -1 38.39000 1.83000 -26.92000 L

H -1 38.89000 3.04000 -25.94000 L

C -1 37.56000 3.66000 -27.38000 L

O -1 36.44000 3.55000 -27.84000 L

N -1 38.31000 4.72000 -27.60000 L

H -1 39.19000 4.81000 -27.13000 L

H -1 38.01000 5.42000 -28.24000 L

C -1 35.41000 3.09000 -23.59000 L

O -1 34.23000 3.08000 -23.92000 L

N -1 35.75000 2.81000 -22.37000 L

H -1 36.71000 2.73000 -22.10000 L

C -1 34.68000 2.61000 -21.41000 L

H -1 34.11000 1.88000 -21.80000 L

C -1 35.29000 2.21000 -20.06000 L

H -1 35.96000 2.91000 -19.81000 L

H -1 35.75000 1.33000 -20.18000 L

C -1 34.25000 2.08000 -18.91000 L

H -1 33.70000 1.25000 -19.03000 L

H -1 33.65000 2.88000 -18.89000 L

C -1 35.05000 2.00000 -17.61000 L

H -1 34.64000 2.56000 -16.90000 L

H -1 36.00000 2.29000 -17.76000 L

C -1 35.06000 0.60000 -17.17000 L

H -1 35.69000 0.07000 -17.74000 L

H -1 34.14000 0.22000 -17.26000 L

N -1 35.49000 0.55000 -15.75000 L

H -1 35.50000 -0.40000 -15.44000 L

H -1 36.41000 0.93000 -15.67000 L

H -1 34.86000 1.08000 -15.19000 L

C -1 33.82000 3.86000 -21.24000 L

O -1 32.56000 3.77000 -21.07000 L

N -1 34.48000 5.05000 -21.23000 L

H -1 35.48000 5.06000 -21.32000 L

C -1 33.73000 6.33000 -21.11000 L

H -1 33.14000 6.25000 -20.30000 L

C -1 34.70000 7.52000 -20.94000 L

H -1 35.26000 7.61000 -21.77000 L

H -1 35.30000 7.35000 -20.15000 L

C -1 33.90000 8.84000 -20.72000 L

H -1 33.31000 9.00000 -21.51000 L

H -1 34.54000 9.60000 -20.61000 L

C -1 32.99000 8.81000 -19.46000 L

O -1 33.48000 8.46000 -18.39000 L

N -1 31.65000 9.05000 -19.63000 L

H -1 31.29000 9.23000 -20.55000 L

H -1 31.04000 9.03000 -18.84000 L

C -1 32.86000 6.53000 -22.34000 L

O -1 31.68000 6.93000 -22.26000 L

N -1 33.38000 6.16000 -23.52000 L

H -1 34.32000 5.81000 -23.60000 L

C -1 32.53000 6.30000 -24.72000 L

H -1 32.24000 7.25000 -24.61000 L

C -1 33.32000 5.94000 -26.14000 L

H -1 32.70000 6.05000 -26.91000 L

H -1 34.10000 6.56000 -26.25000 L

H -1 33.65000 5.00000 -26.11000 L

C -1 31.25000 5.54000 -24.62000 L

O -1 30.17000 6.06000 -25.06000 L

N -1 31.29000 4.33000 -24.05000 L

H -1 32.16000 3.95000 -23.74000 L

C -1 30.06000 3.56000 -23.90000 L

H -1 29.63000 3.63000 -24.80000 L

C -1 30.38000 2.11000 -23.48000 L

H -1 29.54000 1.66000 -23.17000 L

H -1 31.05000 2.12000 -22.73000 L

C -1 30.98000 1.35000 -24.76000 L

H -1 31.84000 1.77000 -25.03000 L

H -1 30.33000 1.40000 -25.52000 L

C -1 31.24000 -0.14000 -24.42000 L

H -1 30.36000 -0.58000 -24.20000 L

H -1 31.84000 -0.19000 -23.62000 L

C -1 31.90000 -0.90000 -25.60000 L

H -1 31.48000 -0.61000 -26.46000 L

H -1 31.77000 -1.89000 -25.48000 L

N -1 33.42000 -0.60000 -25.65000 L

H -1 33.84000 -1.09000 -26.42000 L

H -1 33.56000 0.38000 -25.77000 L

H -1 33.85000 -0.89000 -24.80000 L

C -1 29.17000 4.14000 -22.77000 L

O -1 27.95000 4.17000 -22.91000 L

N -1 29.77000 4.57000 -21.67000 L

H -1 30.76000 4.43000 -21.55000 L

C -1 28.97000 5.28000 -20.60000 L

H -1 28.28000 4.64000 -20.25000 L

C -1 29.86000 5.70000 -19.46000 L

H -1 29.35000 6.33000 -18.87000 L

H -1 30.66000 6.17000 -19.84000 L

C -1 30.35000 4.55000 -18.61000 L

H -1 30.69000 3.85000 -19.24000 L

C -1 31.54000 4.96000 -17.75000 L

H -1 31.85000 4.19000 -17.20000 L

H -1 32.29000 5.27000 -18.35000 L

H -1 31.27000 5.72000 -17.15000 L

C -1 29.15000 3.99000 -17.71000 L

H -1 29.47000 3.23000 -17.15000 L

H -1 28.81000 4.72000 -17.12000 L

H -1 28.40000 3.68000 -18.31000 L

C -1 28.28000 6.52000 -21.18000 L

O -1 27.04000 6.71000 -21.02000 L

N -1 29.03000 7.34000 -21.93000 L

H -1 30.00000 7.14000 -22.08000 L

C -1 28.43000 8.49000 -22.51000 L

H -1 28.13000 8.98000 -21.69000 L

C -1 29.48000 9.32000 -23.37000 L

H -1 29.97000 8.73000 -24.00000 L

C -1 28.76000 10.52000 -24.15000 L

H -1 29.44000 11.03000 -24.68000 L

H -1 28.07000 10.15000 -24.77000 L

H -1 28.33000 11.13000 -23.49000 L

O -1 30.50000 9.86000 -22.48000 L

H -1 31.16000 10.39000 -23.01000 L

C -1 27.23000 8.09000 -23.43000 L

O -1 26.10000 8.68000 -23.38000 L

N -1 27.46000 7.16000 -24.34000 L

H -1 28.36000 6.74000 -24.41000 L

C -1 26.36000 6.75000 -25.24000 L

H -1 26.05000 7.58000 -25.72000 L

C -1 26.88000 5.65000 -26.23000 L

H -1 27.37000 4.95000 -25.71000 L

H -1 27.51000 6.08000 -26.88000 L

C -1 25.74000 4.97000 -27.03000 L

H -1 25.09000 4.57000 -26.37000 L

H -1 26.13000 4.24000 -27.59000 L

C -1 24.95000 5.95000 -27.97000 L

O -1 23.72000 5.71000 -28.25000 L

O -1 25.55000 6.97000 -28.44000 L

C -1 25.19000 6.23000 -24.43000 L

O -1 24.07000 6.31000 -24.86000 L

N -1 25.44000 5.66000 -23.26000 L

H -1 26.38000 5.51000 -22.98000 L

C -1 24.38000 5.26000 -22.42000 L

H -1 23.74000 4.88000 -23.08000 L

C -1 24.97000 4.41000 -21.36000 L

H -1 24.66000 4.72000 -20.46000 L

H -1 25.97000 4.47000 -21.40000 L

C -1 24.61000 3.03000 -21.50000 L

O -1 23.50000 2.72000 -20.96000 L

O -1 25.40000 2.28000 -22.18000 L

C -1 23.75000 6.34000 -21.56000 L

O -1 23.03000 5.96000 -20.61000 L

N -1 24.12000 7.61000 -21.77000 L

H -1 24.83000 7.84000 -22.44000 L

C -1 23.46000 8.67000 -20.99000 L

H -1 22.57000 8.32000 -20.71000 L

H -1 23.34000 9.46000 -21.59000 L

C -1 24.22000 9.13000 -19.74000 L

O -1 23.59000 9.78000 -18.85000 L

N -1 25.53000 8.79000 -19.67000 L

H -1 25.94000 8.26000 -20.41000 L

C -1 26.38000 9.17000 -18.53000 L

H -1 25.84000 9.78000 -17.95000 L

C -1 26.77000 7.90000 -17.78000 L

H -1 27.41000 8.16000 -17.06000 L

H -1 27.22000 7.29000 -18.43000 L

C -1 25.59000 7.12000 -17.13000 L

H -1 25.00000 6.90000 -17.91000 L

C -1 26.00000 5.72000 -16.50000 L

H -1 25.20000 5.28000 -16.10000 L

H -1 26.39000 5.13000 -17.21000 L

H -1 26.69000 5.87000 -15.78000 L

C -1 24.85000 8.06000 -15.98000 L

H -1 24.10000 7.55000 -15.57000 L

H -1 25.52000 8.30000 -15.27000 L

H -1 24.50000 8.89000 -16.40000 L

C -1 27.63000 9.95000 -18.96000 L

O -1 28.75000 9.40000 -19.06000 L

N -1 27.47000 11.23000 -19.26000 L

C -1 26.18000 11.93000 -19.43000 L

H -1 25.95000 12.46000 -18.61000 L

H -1 25.44000 11.29000 -19.62000 L

C -1 26.42000 12.83000 -20.59000 L

H -1 25.88000 13.66000 -20.50000 L

H -1 26.19000 12.37000 -21.44000 L

C -1 27.87000 13.16000 -20.55000 L

H -1 28.02000 14.00000 -20.03000 L

H -1 28.21000 13.28000 -21.48000 L

C -1 28.56000 11.95000 -19.86000 L

H -1 29.04000 11.32000 -20.47000 L

C -1 29.64000 12.43000 -18.86000 L

O -1 29.44000 12.38000 -17.63000 L

N -1 30.74000 12.89000 -19.41000 L

H -1 30.79000 12.97000 -20.41000 L

C -1 31.92000 13.29000 -18.60000 L

H -1 31.66000 13.22000 -17.63000 L

C -1 33.09000 12.36000 -18.93000 L

H -1 33.22000 12.38000 -19.92000 L

H -1 32.83000 11.44000 -18.65000 L

C -1 34.43000 12.68000 -18.27000 L

H -1 34.66000 13.62000 -18.51000 L

C -1 34.23000 12.59000 -16.73000 L

H -1 35.09000 12.80000 -16.27000 L

H -1 33.53000 13.25000 -16.44000 L

H -1 33.93000 11.67000 -16.48000 L

C -1 35.62000 11.71000 -18.79000 L

H -1 36.47000 11.96000 -18.34000 L

H -1 35.39000 10.76000 -18.58000 L

H -1 35.73000 11.82000 -19.78000 L

C -1 32.32000 14.72000 -18.95000 L

O -1 32.51000 15.06000 -20.14000 L

N -1 32.45000 15.57000 -17.94000 L

H -1 32.19000 15.28000 -17.02000 L

C -1 32.96000 16.94000 -18.14000 L

H -1 32.35000 17.59000 -17.70000 L

H -1 33.00000 17.14000 -19.12000 L

C -1 34.36000 16.98000 -17.53000 L

O -1 34.71000 16.32000 -16.51000 L

N -1 35.17000 17.84000 -18.14000 L

H -1 34.86000 18.28000 -18.98000 L

C -1 36.47000 18.13000 -17.64000 L

H -1 36.51000 17.64000 -16.77000 L

C -1 37.55000 17.67000 -18.66000 L

H -1 37.37000 18.09000 -19.55000 L

C -1 38.96000 18.12000 -18.09000 L

H -1 39.68000 17.84000 -18.72000 L

H -1 38.98000 19.11000 -17.98000 L

H -1 39.11000 17.69000 -17.20000 L

C -1 37.51000 16.06000 -18.74000 L

H -1 38.20000 15.74000 -19.39000 L

H -1 37.71000 15.67000 -17.84000 L

H -1 36.60000 15.76000 -19.04000 L

C -1 36.64000 19.61000 -17.39000 L

O -1 36.44000 20.43000 -18.32000 L

N -1 36.95000 19.98000 -16.14000 L

H -1 37.12000 19.27000 -15.45000 L

C -1 37.05000 21.37000 -15.78000 L

H -1 36.58000 21.90000 -16.48000 L

C -1 36.38000 21.49000 -14.35000 L

H -1 36.88000 20.93000 -13.70000 L

H -1 35.43000 21.18000 -14.41000 L

C -1 36.35000 22.90000 -13.81000 L

O -1 37.37000 23.64000 -13.84000 L

N -1 35.16000 23.29000 -13.26000 L

H -1 34.38000 22.65000 -13.24000 L

H -1 35.06000 24.21000 -12.88000 L

C -1 38.55000 21.84000 -15.77000 L

O -1 39.39000 21.16000 -15.17000 L

N -1 38.85000 22.92000 -16.50000 L

H -1 38.09000 23.44000 -16.89000 L

C -1 40.22000 23.39000 -16.76000 L

H -1 40.80000 22.61000 -16.55000 L

C -1 40.40000 23.77000 -18.21000 L

H -1 41.35000 24.07000 -18.32000 L

H -1 39.78000 24.54000 -18.39000 L

C -1 40.12000 22.68000 -19.26000 L

H -1 39.16000 22.43000 -19.14000 L

C -1 40.27000 23.22000 -20.74000 L

H -1 40.08000 22.48000 -21.39000 L

H -1 39.62000 23.96000 -20.89000 L

H -1 41.20000 23.55000 -20.89000 L

C -1 41.07000 21.42000 -19.01000 L

H -1 40.88000 20.72000 -19.70000 L

H -1 42.03000 21.70000 -19.08000 L

H -1 40.90000 21.04000 -18.10000 L

C -1 40.54000 24.64000 -15.89000 L

O -1 39.73000 25.52000 -15.80000 L

N -1 41.70000 24.67000 -15.25000 L

H -1 42.28000 23.86000 -15.29000 L

C -1 42.17000 25.81000 -14.50000 L

H -1 42.25000 25.55000 -13.54000 L

H -1 41.50000 26.55000 -14.59000 L

C -1 43.53000 26.24000 -15.07000 L

O -1 43.93000 25.83000 -16.18000 L

N -1 44.21000 27.10000 -14.35000 L

H -1 43.86000 27.40000 -13.46000 L

C -1 45.45000 27.62000 -14.84000 L

H -1 45.76000 27.02000 -15.58000 L

C -1 45.22000 29.00000 -15.40000 L

H -1 44.49000 28.97000 -16.08000 L

H -1 46.06000 29.32000 -15.84000 L

C -1 44.85000 29.97000 -14.41000 L

H -1 45.12000 29.63000 -13.51000 L

H -1 43.86000 30.10000 -14.44000 L

C -1 45.66000 31.28000 -14.84000 L

H -1 45.02000 31.99000 -15.14000 L

H -1 46.29000 31.07000 -15.59000 L

C -1 46.48000 31.83000 -13.63000 L

H -1 46.80000 31.05000 -13.09000 L

H -1 45.88000 32.41000 -13.08000 L

N -1 47.70000 32.65000 -13.94000 L

H -1 48.14000 32.94000 -13.08000 L

H -1 48.35000 32.11000 -14.47000 L

H -1 47.44000 33.46000 -14.46000 L

C -1 46.46000 27.61000 -13.73000 L

O -1 46.11000 27.64000 -12.56000 L

N -1 47.72000 27.51000 -14.09000 L

H -1 47.98000 27.53000 -15.06000 L

C -1 48.73000 27.37000 -13.08000 L

H -1 48.38000 26.64000 -12.49000 L

C -1 50.07000 26.98000 -13.73000 L

H -1 50.82000 27.28000 -13.14000 L

H -1 50.14000 27.43000 -14.62000 L

C -1 50.18000 25.48000 -13.94000 L

O -1 50.28000 25.04000 -15.06000 L

N -1 50.10000 24.70000 -12.86000 L

H -1 49.97000 25.11000 -11.95000 L

H -1 50.17000 23.71000 -12.95000 L

C -1 48.94000 28.72000 -12.34000 L

O -1 48.83000 29.80000 -12.91000 L

N -1 49.36000 28.59000 -11.09000 L

H -1 49.52000 27.68000 -10.71000 L

C -1 49.59000 29.78000 -10.27000 L

H -1 48.73000 30.28000 -10.18000 L

C -1 50.07000 29.31000 -8.91000 L

H -1 50.91000 28.78000 -9.04000 L

H -1 49.36000 28.73000 -8.51000 L

C -1 50.36000 30.49000 -7.96000 L

H -1 49.51000 30.99000 -7.77000 L

H -1 51.02000 31.10000 -8.37000 L

C -1 50.91000 29.97000 -6.65000 L

H -1 51.81000 29.56000 -6.82000 L

H -1 50.29000 29.28000 -6.28000 L

C -1 51.04000 31.13000 -5.67000 L

H -1 50.19000 31.65000 -5.67000 L

H -1 51.79000 31.72000 -5.97000 L

N -1 51.33000 30.64000 -4.26000 L

H -1 51.40000 31.43000 -3.65000 L

H -1 50.58000 30.05000 -3.96000 L

H -1 52.18000 30.12000 -4.26000 L

C -1 50.63000 30.71000 -10.92000 L

O -1 50.44000 31.90000 -11.03000 L

N -1 51.75000 30.19000 -11.37000 L

H -1 51.91000 29.21000 -11.35000 L

C -1 52.77000 31.13000 -11.90000 L

H -1 52.52000 32.01000 -11.49000 L

C -1 54.14000 30.70000 -11.48000 L

H -1 54.83000 31.32000 -11.87000 L

C -1 54.20000 30.63000 -9.93000 L

H -1 55.12000 30.35000 -9.64000 L

H -1 54.00000 31.53000 -9.55000 L

H -1 53.53000 29.97000 -9.60000 L

O -1 54.37000 29.38000 -12.00000 L

H -1 55.29000 29.08000 -11.73000 L

C -1 52.76000 31.26000 -13.45000 L

O -1 53.73000 31.78000 -14.05000 L

N -1 51.66000 30.87000 -14.10000 L

H -1 50.88000 30.48000 -13.61000 L

C -1 51.62000 31.03000 -15.58000 L

H -1 52.49000 30.67000 -15.91000 L

C -1 50.51000 30.22000 -16.22000 L

H -1 50.43000 29.31000 -15.80000 L

H -1 50.65000 30.13000 -17.20000 L

O -1 49.29000 30.87000 -16.03000 L

H -1 48.56000 30.34000 -16.45000 L

C -1 51.57000 32.49000 -15.98000 L

O -1 51.00000 33.32000 -15.29000 L

N -1 52.19000 32.80000 -17.11000 L

H -1 52.64000 32.09000 -17.64000 L

C -1 52.21000 34.19000 -17.56000 L

H -1 51.89000 34.67000 -16.75000 L

C -1 53.59000 34.59000 -18.03000 L

H -1 53.96000 33.90000 -18.65000 L

C -1 53.47000 35.89000 -18.75000 L

H -1 54.37000 36.18000 -19.07000 L

H -1 52.86000 35.78000 -19.53000 L

H -1 53.10000 36.58000 -18.13000 L

C -1 54.51000 34.77000 -16.81000 L

H -1 55.43000 35.04000 -17.11000 L

H -1 54.14000 35.49000 -16.21000 L

H -1 54.57000 33.91000 -16.30000 L

C -1 51.30000 34.45000 -18.75000 L

O -1 51.42000 35.50000 -19.41000 L

N -1 50.44000 33.50000 -19.07000 L

H -1 50.40000 32.67000 -18.51000 L

C -1 49.58000 33.65000 -20.20000 L

H -1 49.26000 34.59000 -20.32000 L

C -1 50.40000 33.24000 -21.41000 L

H -1 50.67000 32.29000 -21.30000 L

H -1 51.22000 33.82000 -21.46000 L

C -1 49.66000 33.38000 -22.68000 L

O -1 48.42000 33.60000 -22.70000 L

O -1 50.36000 33.19000 -23.70000 L

C -1 48.41000 32.73000 -19.92000 L

O -1 48.43000 31.54000 -20.24000 L

N -1 47.40000 33.28000 -19.26000 L

H -1 47.43000 34.26000 -19.06000 L

C -1 46.27000 32.49000 -18.83000 L

H -1 46.62000 31.82000 -18.17000 L

C -1 45.21000 33.42000 -18.22000 L

H -1 44.42000 32.88000 -17.92000 L

H -1 45.60000 33.90000 -17.44000 L

H -1 44.92000 34.08000 -18.91000 L

C -1 45.64000 31.78000 -20.00000 L

O -1 45.18000 30.65000 -19.92000 L

N -1 45.42000 32.53000 -21.06000 L

H -1 45.72000 33.49000 -21.09000 L

C -1 44.73000 31.94000 -22.19000 L

H -1 43.88000 31.62000 -21.77000 L

C -1 44.50000 32.99000 -23.35000 L

H -1 44.02000 32.54000 -24.11000 L

H -1 43.94000 33.74000 -23.01000 L

H -1 45.38000 33.34000 -23.67000 L

C -1 45.47000 30.72000 -22.75000 L

O -1 44.81000 29.82000 -23.25000 L

N -1 46.79000 30.73000 -22.80000 L

H -1 47.30000 31.54000 -22.49000 L

C -1 47.52000 29.57000 -23.32000 L

H -1 47.10000 29.36000 -24.20000 L

C -1 49.03000 29.91000 -23.53000 L

H -1 49.43000 30.12000 -22.64000 L

H -1 49.09000 30.71000 -24.12000 L

C -1 49.82000 28.75000 -24.17000 L

H -1 49.80000 27.97000 -23.55000 L

H -1 50.76000 29.05000 -24.30000 L

C -1 49.24000 28.31000 -25.56000 L

O -1 49.51000 27.18000 -25.96000 L

O -1 48.49000 29.06000 -26.24000 L

C -1 47.38000 28.38000 -22.31000 L

O -1 47.29000 27.24000 -22.75000 L

N -1 47.34000 28.63000 -20.98000 L

H -1 47.54000 29.54000 -20.62000 L

C -1 47.00000 27.50000 -20.07000 L

H -1 47.78000 26.89000 -20.17000 L

C -1 46.85000 27.93000 -18.60000 L

H -1 46.36000 27.21000 -18.10000 L

H -1 46.34000 28.78000 -18.55000 L

C -1 48.18000 28.14000 -17.93000 L

O -1 49.22000 28.24000 -18.61000 L

O -1 48.26000 28.13000 -16.72000 L

C -1 45.65000 26.86000 -20.47000 L

O -1 45.55000 25.63000 -20.55000 L

N -1 44.59000 27.64000 -20.69000 L

H -1 44.65000 28.64000 -20.62000 L

C -1 43.31000 26.99000 -21.03000 L

H -1 43.14000 26.32000 -20.31000 L

C -1 42.14000 28.00000 -21.08000 L

H -1 41.33000 27.55000 -21.43000 L

H -1 42.39000 28.77000 -21.68000 L

C -1 41.88000 28.52000 -19.64000 L

C -1 42.31000 29.78000 -19.22000 L

H -1 42.78000 30.38000 -19.86000 L

C -1 42.09000 30.22000 -17.83000 L

H -1 42.41000 31.12000 -17.52000 L

C -1 41.42000 29.39000 -16.95000 L

O -1 41.15000 29.81000 -15.65000 L

H -1 40.66000 29.09000 -15.15000 L

C -1 40.95000 28.16000 -17.37000 L

H -1 40.45000 27.57000 -16.74000 L

C -1 41.20000 27.73000 -18.74000 L

H -1 40.86000 26.83000 -19.03000 L

C -1 43.45000 26.31000 -22.39000 L

O -1 42.78000 25.30000 -22.65000 L

N -1 44.13000 26.99000 -23.33000 L

H -1 44.56000 27.87000 -23.13000 L

C -1 44.22000 26.39000 -24.66000 L

H -1 43.27000 26.29000 -24.97000 L

C -1 45.06000 27.33000 -25.61000 L

H -1 45.12000 26.92000 -26.52000 L

H -1 44.62000 28.22000 -25.67000 L

H -1 45.98000 27.44000 -25.23000 L

C -1 44.87000 25.02000 -24.59000 L

O -1 44.51000 24.08000 -25.35000 L

N -1 45.93000 24.92000 -23.81000 L

H -1 46.28000 25.74000 -23.36000 L

C -1 46.61000 23.64000 -23.57000 L

H -1 46.90000 23.38000 -24.49000 L

C -1 47.80000 23.81000 -22.59000 L

H -1 48.03000 22.91000 -22.20000 L

H -1 47.53000 24.43000 -21.85000 L

C -1 49.06000 24.39000 -23.27000 L

H -1 48.79000 25.19000 -23.81000 L

H -1 49.44000 23.69000 -23.88000 L

C -1 50.17000 24.82000 -22.29000 L

O -1 51.20000 25.41000 -22.71000 L

O -1 50.03000 24.58000 -21.09000 L

C -1 45.66000 22.56000 -22.95000 L

O -1 45.65000 21.39000 -23.40000 L

N -1 44.84000 22.98000 -21.99000 L

H -1 44.93000 23.91000 -21.64000 L

C -1 43.81000 22.11000 -21.45000 L

H -1 43.28000 22.63000 -20.78000 L

H -1 44.26000 21.34000 -20.99000 L

C -1 42.88000 21.58000 -22.55000 L

O -1 42.47000 20.39000 -22.53000 L

N -1 42.47000 22.49000 -23.43000 L

H -1 42.77000 23.44000 -23.34000 L

C -1 41.57000 22.10000 -24.53000 L

H -1 40.75000 21.72000 -24.09000 L

C -1 41.15000 23.32000 -25.40000 L

H -1 41.99000 23.73000 -25.75000 L

C -1 40.30000 22.87000 -26.59000 L

H -1 40.04000 23.66000 -27.14000 L

H -1 40.82000 22.23000 -27.15000 L

H -1 39.47000 22.41000 -26.25000 L

C -1 40.31000 24.35000 -24.57000 L

H -1 40.05000 25.12000 -25.16000 L

H -1 39.48000 23.91000 -24.23000 L

H -1 40.85000 24.69000 -23.80000 L

C -1 42.26000 21.04000 -25.39000 L

O -1 41.58000 20.05000 -25.76000 L

N -1 43.54000 21.30000 -25.76000 L

H -1 43.99000 22.12000 -25.42000 L

C -1 44.28000 20.39000 -26.68000 L

H -1 43.70000 20.29000 -27.49000 L

C -1 45.66000 21.01000 -27.09000 L

H -1 46.27000 20.32000 -27.48000 L

H -1 46.11000 21.46000 -26.32000 L

C -1 45.24000 22.05000 -28.16000 L

H -1 44.78000 22.82000 -27.73000 L

H -1 44.62000 21.62000 -28.82000 L

C -1 46.40000 22.57000 -28.91000 L

H -1 46.14000 23.35000 -29.47000 L

H -1 46.81000 21.87000 -29.48000 L

N -1 47.34000 22.99000 -27.96000 L

H -1 47.83000 22.32000 -27.40000 L

C -1 47.59000 24.28000 -27.79000 L

N -1 46.87000 25.08000 -28.54000 L

H -1 46.20000 24.70000 -29.17000 L

H -1 47.00000 26.07000 -28.47000 L

N -1 48.49000 24.73000 -26.89000 L

H -1 48.99000 24.08000 -26.32000 L

H -1 48.65000 25.71000 -26.79000 L

C -1 44.45000 18.99000 -26.06000 L

O -1 44.22000 17.98000 -26.72000 L

N -1 44.71000 18.96000 -24.76000 L

H -1 44.60000 19.78000 -24.19000 L

C -1 45.16000 17.66000 -24.16000 L

H -1 45.61000 17.10000 -24.86000 L

C -1 46.15000 17.97000 -23.02000 L

H -1 45.67000 18.55000 -22.37000 L

C -1 46.46000 16.69000 -22.20000 L

H -1 47.11000 16.91000 -21.47000 L

H -1 45.62000 16.33000 -21.80000 L

H -1 46.87000 16.00000 -22.80000 L

C -1 47.50000 18.73000 -23.59000 L

H -1 48.13000 18.92000 -22.84000 L

H -1 47.96000 18.15000 -24.26000 L

H -1 47.24000 19.59000 -24.03000 L

C -1 43.95000 16.90000 -23.63000 L

O -1 43.83000 15.70000 -23.84000 L

N -1 43.00000 17.59000 -22.92000 L

H -1 43.10000 18.58000 -22.77000 L

C -1 41.85000 16.87000 -22.38000 L

H -1 42.12000 15.92000 -22.51000 L

C -1 41.61000 17.27000 -20.88000 L

H -1 40.80000 16.81000 -20.53000 L

H -1 41.48000 18.26000 -20.82000 L

C -1 42.80000 16.89000 -19.97000 L

H -1 43.59000 17.41000 -20.30000 L

C -1 42.53000 17.29000 -18.44000 L

H -1 43.31000 17.03000 -17.88000 L

H -1 42.38000 18.27000 -18.37000 L

H -1 41.71000 16.81000 -18.11000 L

C -1 43.11000 15.36000 -20.12000 L

H -1 43.88000 15.12000 -19.53000 L

H -1 42.31000 14.83000 -19.85000 L

H -1 43.34000 15.16000 -21.07000 L

C -1 40.56000 17.02000 -23.17000 L

O -1 39.63000 16.20000 -23.03000 L

N -1 40.50000 18.02000 -24.06000 L

H -1 41.25000 18.67000 -24.16000 L

C -1 39.30000 18.12000 -24.87000 L

H -1 39.45000 18.87000 -25.52000 L

H -1 38.55000 18.36000 -24.25000 L

C -1 38.93000 16.88000 -25.62000 L

O -1 37.73000 16.56000 -25.74000 L

N -1 39.95000 16.14000 -26.10000 L

C -1 41.34000 16.63000 -26.37000 L

H -1 41.92000 16.48000 -25.57000 L

H -1 41.32000 17.60000 -26.59000 L

C -1 41.79000 15.75000 -27.60000 L

H -1 42.75000 15.51000 -27.51000 L

H -1 41.64000 16.25000 -28.45000 L

C -1 40.90000 14.45000 -27.57000 L

H -1 41.35000 13.73000 -27.05000 L

H -1 40.71000 14.14000 -28.50000 L

C -1 39.57000 14.91000 -26.84000 L

H -1 38.79000 15.12000 -27.43000 L

C -1 39.01000 13.82000 -25.99000 L

O -1 38.47000 12.84000 -26.50000 L

N -1 39.11000 13.97000 -24.68000 L

H -1 39.54000 14.79000 -24.30000 L

C -1 38.59000 12.94000 -23.80000 L

H -1 38.39000 12.20000 -24.44000 L

C -1 39.67000 12.62000 -22.69000 L

H -1 39.28000 12.02000 -22.00000 L

H -1 39.98000 13.47000 -22.26000 L

C -1 40.91000 11.91000 -23.37000 L

H -1 40.88000 12.12000 -24.34000 L

C -1 42.26000 12.37000 -22.74000 L

H -1 43.02000 11.89000 -23.20000 L

H -1 42.37000 13.35000 -22.87000 L

H -1 42.27000 12.15000 -21.77000 L

C -1 40.82000 10.42000 -23.27000 L

H -1 41.62000 10.02000 -23.71000 L

H -1 40.80000 10.16000 -22.30000 L

H -1 39.99000 10.10000 -23.72000 L

C -1 37.31000 13.32000 -23.08000 L

O -1 36.84000 12.55000 -22.21000 L

N -1 36.69000 14.45000 -23.44000 L

H -1 36.97000 14.93000 -24.27000 L

C -1 35.59000 15.00000 -22.60000 L

H -1 35.41000 14.34000 -21.87000 L

C -1 36.02000 16.45000 -22.08000 L

H -1 35.28000 16.83000 -21.52000 L

H -1 36.85000 16.37000 -21.54000 L

H -1 36.18000 17.05000 -22.87000 L

C -1 34.36000 15.18000 -23.47000 L

O -1 34.47000 15.55000 -24.64000 L

N -1 33.17000 15.08000 -22.86000 L

H -1 33.12000 14.73000 -21.92000 L

C -1 31.97000 15.47000 -23.56000 L

H -1 32.02000 15.24000 -24.53000 L

C -1 30.82000 14.72000 -22.89000 L

H -1 29.96000 15.07000 -23.25000 L

H -1 30.87000 14.88000 -21.90000 L

C -1 30.94000 13.19000 -23.18000 L

O -1 31.18000 12.89000 -24.35000 L

O -1 30.88000 12.34000 -22.26000 L

C -1 31.84000 16.96000 -23.45000 L

O -1 31.31000 17.58000 -24.37000 L

N -1 32.25000 17.56000 -22.31000 L

H -1 32.54000 17.03000 -21.52000 L

C -1 32.27000 19.04000 -22.30000 L

H -1 32.39000 19.39000 -23.23000 L

C -1 30.92000 19.58000 -21.76000 L

H -1 30.19000 19.12000 -22.26000 L

H -1 30.89000 20.56000 -21.96000 L

C -1 30.66000 19.39000 -20.23000 L

C -1 29.90000 18.32000 -19.79000 L

H -1 29.55000 17.66000 -20.46000 L

C -1 29.59000 18.14000 -18.38000 L

H -1 28.98000 17.41000 -18.07000 L

C -1 30.18000 19.00000 -17.48000 L

O -1 29.91000 18.79000 -16.13000 L

H -1 30.40000 19.48000 -15.59000 L

C -1 31.03000 20.03000 -17.89000 L

H -1 31.48000 20.63000 -17.22000 L

C -1 31.25000 20.22000 -19.29000 L

H -1 31.83000 20.97000 -19.60000 L

C -1 33.45000 19.48000 -21.48000 L

O -1 33.93000 18.72000 -20.63000 L

N -1 33.95000 20.68000 -21.79000 L

H -1 33.56000 21.17000 -22.57000 L

C -1 35.03000 21.29000 -21.08000 L

H -1 35.42000 20.57000 -20.50000 L

C -1 36.06000 21.88000 -22.03000 L

H -1 36.70000 22.42000 -21.49000 L

H -1 35.58000 22.47000 -22.68000 L

C -1 36.84000 20.80000 -22.81000 L

H -1 36.24000 20.17000 -23.29000 L

C -1 37.75000 21.56000 -23.83000 L

H -1 38.28000 20.89000 -24.36000 L

H -1 37.18000 22.10000 -24.44000 L

H -1 38.38000 22.16000 -23.33000 L

C -1 37.79000 20.09000 -21.73000 L

H -1 38.33000 19.38000 -22.18000 L

H -1 38.40000 20.77000 -21.33000 L

H -1 37.23000 19.68000 -21.02000 L

C -1 34.45000 22.48000 -20.33000 L

O -1 33.47000 23.13000 -20.82000 L

N -1 35.09000 22.83000 -19.22000 L

H -1 35.88000 22.30000 -18.91000 L

C -1 34.62000 24.02000 -18.45000 L

H -1 33.82000 24.40000 -18.92000 L

C -1 34.28000 23.68000 -17.00000 L

H -1 35.13000 23.42000 -16.55000 L

C -1 33.66000 24.97000 -16.28000 L

H -1 33.44000 24.74000 -15.33000 L

H -1 34.33000 25.71000 -16.30000 L

H -1 32.83000 25.25000 -16.75000 L

C -1 33.23000 22.48000 -16.99000 L

H -1 33.00000 22.25000 -16.04000 L

H -1 32.40000 22.75000 -17.48000 L

H -1 33.63000 21.68000 -17.44000 L

C -1 35.80000 24.91000 -18.36000 L

O -1 36.85000 24.51000 -17.88000 L

N -1 35.63000 26.14000 -18.79000 L

H -1 34.79000 26.37000 -19.29000 L

C -1 36.63000 27.19000 -18.56000 L

H -1 37.54000 26.76000 -18.53000 L

C -1 36.59000 28.24000 -19.69000 L

H -1 35.70000 28.68000 -19.67000 L

C -1 37.63000 29.38000 -19.42000 L

H -1 37.59000 30.05000 -20.16000 L

H -1 37.41000 29.83000 -18.55000 L

H -1 38.55000 28.99000 -19.37000 L

C -1 36.83000 27.52000 -21.11000 L

H -1 36.81000 28.21000 -21.84000 L

H -1 37.72000 27.07000 -21.11000 L

H -1 36.11000 26.84000 -21.27000 L

C -1 36.26000 27.84000 -17.23000 L

O -1 35.31000 28.67000 -17.12000 L

N -1 37.03000 27.43000 -16.24000 L

H -1 37.78000 26.81000 -16.44000 L

C -1 36.81000 27.86000 -14.90000 L

H -1 35.82000 27.94000 -14.80000 L

C -1 37.44000 26.84000 -13.93000 L

H -1 38.43000 26.81000 -14.09000 L

H -1 37.05000 25.94000 -14.11000 L

C -1 37.16000 27.26000 -12.45000 L

O -1 36.40000 28.15000 -12.27000 L

N -1 37.81000 26.68000 -11.49000 L

H -1 38.48000 25.96000 -11.70000 L

H -1 37.64000 26.94000 -10.54000 L

C -1 37.53000 29.16000 -14.67000 L

O -1 38.78000 29.16000 -14.51000 L

N -1 36.80000 30.27000 -14.77000 L

H -1 35.84000 30.21000 -15.02000 L

C -1 37.41000 31.56000 -14.50000 L

H -1 38.39000 31.37000 -14.39000 L

C -1 37.21000 32.50000 -15.74000 L

H -1 37.73000 32.15000 -16.52000 L

C -1 35.71000 32.57000 -16.11000 L

H -1 35.59000 33.17000 -16.90000 L

H -1 35.38000 31.65000 -16.34000 L

H -1 35.19000 32.92000 -15.33000 L

C -1 37.77000 33.90000 -15.43000 L

H -1 37.63000 34.49000 -16.23000 L

H -1 37.28000 34.29000 -14.65000 L

H -1 38.74000 33.84000 -15.23000 L

C -1 36.83000 32.16000 -13.16000 L

O -1 36.77000 33.41000 -12.96000 L

N -1 36.43000 31.28000 -12.24000 L

H -1 36.63000 30.31000 -12.35000 L

C -1 35.70000 31.75000 -11.07000 L

H -1 35.92000 32.72000 -11.07000 L

C -1 34.15000 31.48000 -11.22000 L

H -1 33.76000 32.05000 -11.96000 L

H -1 33.67000 31.67000 -10.37000 L

O -1 33.88000 30.13000 -11.56000 L

H -1 32.89000 30.01000 -11.65000 L

C -1 36.21000 31.20000 -9.74000 L

O -1 35.56000 31.46000 -8.69000 L

N -1 37.36000 30.48000 -9.73000 L

H -1 37.81000 30.20000 -10.58000 L

C -1 37.90000 30.12000 -8.40000 L

H -1 37.22000 29.54000 -7.96000 L

C -1 39.24000 29.40000 -8.57000 L

H -1 39.91000 29.99000 -9.03000 L

H -1 39.14000 28.56000 -9.09000 L

O -1 39.76000 29.07000 -7.26000 L

H -1 40.64000 28.60000 -7.36000 L

C -1 38.20000 31.42000 -7.63000 L

O -1 38.87000 32.28000 -8.17000 L

N -1 37.80000 31.51000 -6.35000 L

C -1 36.94000 30.59000 -5.57000 L

H -1 37.42000 29.73000 -5.41000 L

H -1 36.09000 30.41000 -6.08000 L

C -1 36.67000 31.36000 -4.24000 L

H -1 37.09000 30.90000 -3.47000 L

H -1 35.69000 31.45000 -4.08000 L

C -1 37.28000 32.74000 -4.39000 L

H -1 37.76000 32.99000 -3.55000 L

H -1 36.56000 33.41000 -4.58000 L

C -1 38.26000 32.68000 -5.58000 L

H -1 38.27000 33.48000 -6.17000 L

C -1 39.68000 32.50000 -5.03000 L

O -1 40.15000 33.37000 -4.37000 L

N -1 40.32000 31.38000 -5.30000 L

H -1 39.95000 30.76000 -6.00000 L

C -1 41.57000 30.99000 -4.62000 L

H -1 41.67000 31.70000 -3.92000 L

C -1 41.46000 29.59000 -4.04000 L

H -1 42.29000 29.37000 -3.53000 L

H -1 41.33000 28.93000 -4.78000 L

C -1 40.29000 29.51000 -3.10000 L

O -1 40.08000 30.47000 -2.31000 L

N -1 39.47000 28.46000 -3.21000 L

H -1 39.64000 27.76000 -3.90000 L

H -1 38.68000 28.38000 -2.60000 L

C -1 42.78000 31.04000 -5.50000 L

O -1 43.84000 30.51000 -5.10000 L

N -1 42.64000 31.71000 -6.66000 L

H -1 41.74000 32.05000 -6.93000 L

C -1 43.81000 31.96000 -7.52000 L

H -1 44.62000 31.71000 -6.98000 L

C -1 43.76000 31.07000 -8.79000 L

H -1 42.86000 31.09000 -9.23000 L

C -1 44.84000 31.51000 -9.79000 L

H -1 44.80000 30.94000 -10.60000 L

H -1 44.69000 32.47000 -10.04000 L

H -1 45.74000 31.42000 -9.36000 L

O -1 43.99000 29.70000 -8.40000 L

H -1 43.96000 29.11000 -9.21000 L

C -1 43.85000 33.44000 -7.87000 L

O -1 42.86000 33.97000 -8.46000 L

N -1 44.96000 34.11000 -7.52000 L

H -1 45.74000 33.58000 -7.17000 L

C -1 45.10000 35.60000 -7.64000 L

H -1 44.40000 35.99000 -7.05000 L

C -1 46.55000 36.06000 -7.21000 L

H -1 46.63000 37.06000 -7.30000 L

H -1 46.72000 35.80000 -6.26000 L

H -1 47.23000 35.63000 -7.81000 L

C -1 44.84000 36.07000 -9.08000 L

O -1 45.46000 35.56000 -10.00000 L

N -1 43.95000 37.04000 -9.28000 L

H -1 43.46000 37.41000 -8.49000 L

C -1 43.67000 37.57000 -10.62000 L

H -1 44.55000 37.66000 -11.09000 L

H -1 43.27000 38.48000 -10.50000 L

C -1 42.74000 36.75000 -11.51000 L

O -1 42.32000 37.22000 -12.60000 L

N -1 42.45000 35.52000 -11.11000 L

H -1 42.75000 35.19000 -10.21000 L

C -1 41.69000 34.63000 -12.01000 L

H -1 42.25000 34.59000 -12.84000 L

C -1 41.52000 33.23000 -11.36000 L

H -1 40.93000 33.32000 -10.56000 L

H -1 42.42000 32.90000 -11.07000 L

C -1 40.89000 32.22000 -12.35000 L

H -1 40.16000 32.69000 -12.85000 L

C -1 41.96000 31.77000 -13.30000 L

H -1 41.58000 31.12000 -13.95000 L

H -1 42.31000 32.57000 -13.80000 L

H -1 42.70000 31.34000 -12.79000 L

C -1 40.23000 30.97000 -11.68000 L

H -1 39.85000 30.37000 -12.38000 L

H -1 40.91000 30.47000 -11.15000 L

H -1 39.49000 31.27000 -11.07000 L

C -1 40.31000 35.20000 -12.36000 L

O -1 39.84000 35.13000 -13.50000 L

N -1 39.63000 35.72000 -11.35000 L

H -1 40.06000 35.84000 -10.46000 L

C -1 38.24000 36.11000 -11.54000 L

H -1 37.82000 35.34000 -12.01000 L

C -1 37.55000 36.38000 -10.17000 L

H -1 36.72000 36.92000 -10.32000 L

H -1 38.18000 36.89000 -9.58000 L

C -1 37.16000 35.05000 -9.47000 L

H -1 37.97000 34.47000 -9.38000 L

H -1 36.47000 34.57000 -10.02000 L

C -1 36.59000 35.35000 -8.06000 L

H -1 36.28000 34.51000 -7.62000 L

H -1 35.82000 35.99000 -8.12000 L

N -1 37.67000 35.96000 -7.26000 L

H -1 38.62000 35.77000 -7.51000 L

C -1 37.45000 36.75000 -6.22000 L

N -1 36.19000 37.04000 -5.90000 L

H -1 35.44000 36.65000 -6.45000 L

H -1 35.99000 37.63000 -5.13000 L

N -1 38.46000 37.29000 -5.53000 L

H -1 39.41000 37.09000 -5.80000 L

H -1 38.28000 37.88000 -4.75000 L

C -1 38.13000 37.35000 -12.42000 L

O -1 37.07000 37.60000 -13.00000 L

N -1 39.23000 38.11000 -12.57000 L

H -1 40.05000 37.91000 -12.04000 L

C -1 39.22000 39.24000 -13.52000 L

H -1 38.37000 39.73000 -13.32000 L

C -1 40.35000 40.21000 -13.24000 L

H -1 40.50000 40.34000 -12.26000 L

H -1 40.18000 41.10000 -13.67000 L

O -1 41.53000 39.63000 -13.82000 L

H -1 42.30000 40.24000 -13.66000 L

C -1 39.16000 38.75000 -14.99000 L

O -1 38.80000 39.51000 -15.87000 L

N -1 39.39000 37.46000 -15.25000 L

H -1 39.73000 36.86000 -14.52000 L

C -1 39.14000 36.89000 -16.62000 L

H -1 39.63000 37.52000 -17.24000 L

C -1 39.69000 35.45000 -16.73000 L

H -1 39.44000 35.07000 -17.62000 L

H -1 39.29000 34.89000 -16.00000 L

C -1 41.21000 35.41000 -16.59000 L

H -1 41.42000 35.85000 -15.72000 L

C -1 41.73000 33.97000 -16.40000 L

H -1 42.73000 33.99000 -16.31000 L

H -1 41.33000 33.58000 -15.57000 L

H -1 41.47000 33.42000 -17.19000 L

C -1 41.94000 36.24000 -17.77000 L

H -1 42.93000 36.19000 -17.65000 L

H -1 41.69000 35.84000 -18.65000 L

H -1 41.64000 37.19000 -17.74000 L

C -1 37.66000 36.87000 -17.00000 L

O -1 37.31000 36.57000 -18.13000 L

N -1 36.78000 37.08000 -16.02000 L

H -1 37.12000 37.16000 -15.08000 L

C -1 35.33000 37.18000 -16.27000 L

H -1 35.12000 36.44000 -16.90000 L

C -1 34.56000 37.03000 -14.92000 L

H -1 33.61000 37.30000 -15.05000 L

H -1 34.98000 37.61000 -14.22000 L

C -1 34.61000 35.55000 -14.45000 L

H -1 35.56000 35.23000 -14.45000 L

H -1 34.08000 34.98000 -15.08000 L

C -1 34.03000 35.39000 -13.04000 L

O -1 33.00000 34.72000 -12.87000 L

N -1 34.62000 36.10000 -12.05000 L

H -1 35.40000 36.69000 -12.26000 L

H -1 34.28000 36.03000 -11.11000 L

C -1 34.98000 38.59000 -16.87000 L

O -1 33.89000 38.78000 -17.42000 L

N -1 35.89000 39.54000 -16.79000 L

H -1 36.74000 39.37000 -16.29000 L

C -1 35.64000 40.86000 -17.42000 L

H -1 36.45000 41.44000 -17.27000 L

H -1 34.85000 41.28000 -16.98000 L

C -1 35.38000 40.72000 -18.93000 L

O -1 35.96000 39.83000 -19.62000 L

N -1 34.58000 41.65000 -19.49000 L

H -1 34.25000 42.42000 -18.95000 L

C -1 34.19000 41.52000 -20.88000 L

H -1 33.62000 40.70000 -20.80000 L

C -1 33.40000 42.73000 -21.35000 L

H -1 34.02000 43.50000 -21.51000 L

H -1 32.73000 42.98000 -20.66000 L

C -1 32.69000 42.40000 -22.64000 L

H -1 32.25000 41.51000 -22.54000 L

H -1 33.37000 42.36000 -23.37000 L

C -1 31.61000 43.47000 -23.01000 L

H -1 32.03000 44.36000 -23.18000 L

H -1 30.93000 43.55000 -22.28000 L

C -1 30.95000 42.96000 -24.27000 L

H -1 30.74000 41.99000 -24.16000 L

H -1 31.58000 43.08000 -25.04000 L

N -1 29.69000 43.70000 -24.56000 L

H -1 29.28000 43.34000 -25.40000 L

H -1 29.05000 43.58000 -23.80000 L

H -1 29.89000 44.67000 -24.68000 L

C -1 35.33000 41.25000 -21.92000 L

O -1 35.25000 40.36000 -22.76000 L

N -1 36.33000 42.14000 -21.94000 L

H -1 36.41000 42.83000 -21.22000 L

C -1 37.30000 42.10000 -23.04000 L

H -1 36.77000 42.00000 -23.88000 L

C -1 38.08000 43.40000 -23.09000 L

H -1 38.74000 43.36000 -23.84000 L

H -1 37.45000 44.16000 -23.25000 L

H -1 38.56000 43.54000 -22.23000 L

C -1 38.29000 40.90000 -22.81000 L

O -1 38.64000 40.27000 -23.73000 L

N -1 38.74000 40.66000 -21.58000 L

H -1 38.52000 41.32000 -20.86000 L

C -1 39.55000 39.47000 -21.21000 L

H -1 40.38000 39.56000 -21.77000 L

C -1 39.93000 39.41000 -19.69000 L

H -1 40.34000 38.52000 -19.52000 L

H -1 39.08000 39.49000 -19.16000 L

C -1 40.93000 40.51000 -19.18000 L

H -1 40.45000 41.39000 -19.19000 L

H -1 41.70000 40.55000 -19.81000 L

C -1 41.48000 40.26000 -17.74000 L

O -1 41.92000 41.25000 -17.08000 L

O -1 41.50000 39.09000 -17.25000 L

C -1 38.79000 38.22000 -21.55000 L

O -1 39.38000 37.29000 -22.13000 L

N -1 37.52000 38.16000 -21.16000 L

H -1 37.11000 38.95000 -20.68000 L

C -1 36.73000 37.01000 -21.41000 L

H -1 37.25000 36.30000 -20.94000 L

C -1 35.30000 37.10000 -20.82000 L

H -1 34.80000 37.79000 -21.34000 L

H -1 35.38000 37.40000 -19.86000 L

C -1 34.46000 35.81000 -20.83000 L

H -1 34.45000 35.55000 -21.80000 L

C -1 35.13000 34.68000 -19.98000 L

H -1 34.56000 33.86000 -20.02000 L

H -1 36.03000 34.48000 -20.35000 L

H -1 35.22000 34.99000 -19.04000 L

C -1 32.97000 36.01000 -20.36000 L

H -1 32.49000 35.14000 -20.40000 L

H -1 32.96000 36.36000 -19.43000 L

H -1 32.51000 36.67000 -20.97000 L

C -1 36.62000 36.78000 -22.92000 L

O -1 36.77000 35.67000 -23.33000 L

N -1 36.32000 37.82000 -23.72000 L

H -1 36.29000 38.75000 -23.36000 L

C -1 36.04000 37.54000 -25.11000 L

H -1 35.29000 36.89000 -25.12000 L

C -1 35.60000 38.82000 -25.83000 L

H -1 36.33000 39.49000 -25.75000 L

H -1 34.77000 39.16000 -25.40000 L

C -1 35.32000 38.60000 -27.33000 L

H -1 34.48000 38.06000 -27.41000 L

H -1 36.08000 38.09000 -27.72000 L

C -1 35.15000 39.97000 -28.08000 L

H -1 35.06000 39.82000 -29.07000 L

H -1 35.93000 40.57000 -27.90000 L

N -1 33.94000 40.69000 -27.65000 L

H -1 33.05000 40.28000 -27.87000 L

C -1 33.93000 41.84000 -26.98000 L

N -1 35.07000 42.41000 -26.63000 L

H -1 35.94000 41.99000 -26.86000 L

H -1 35.06000 43.28000 -26.12000 L

N -1 32.78000 42.38000 -26.62000 L

H -1 31.92000 41.93000 -26.86000 L

H -1 32.77000 43.25000 -26.12000 L

C -1 37.34000 36.99000 -25.78000 L

O -1 37.31000 36.07000 -26.57000 L

N -1 38.46000 37.61000 -25.45000 L

H -1 38.42000 38.37000 -24.80000 L

C -1 39.74000 37.20000 -26.01000 L

H -1 39.65000 37.24000 -27.01000 L

C -1 40.82000 38.16000 -25.54000 L

H -1 40.50000 38.57000 -24.69000 L

H -1 40.91000 38.87000 -26.24000 L

C -1 42.20000 37.50000 -25.30000 L

H -1 42.39000 36.83000 -26.02000 L

H -1 42.20000 37.03000 -24.41000 L

C -1 43.31000 38.59000 -25.30000 L

H -1 44.21000 38.19000 -25.46000 L

H -1 43.31000 39.10000 -24.44000 L

N -1 43.00000 39.51000 -26.40000 L

H -1 43.39000 39.30000 -27.30000 L

C -1 42.25000 40.61000 -26.31000 L

N -1 41.76000 41.01000 -25.14000 L

H -1 41.95000 40.49000 -24.31000 L

H -1 41.20000 41.84000 -25.09000 L

N -1 42.03000 41.34000 -27.41000 L

H -1 42.43000 41.05000 -28.28000 L

H -1 41.47000 42.16000 -27.36000 L

C -1 40.10000 35.77000 -25.58000 L

O -1 40.50000 34.94000 -26.42000 L

N -1 39.95000 35.48000 -24.30000 L

H -1 39.66000 36.20000 -23.66000 L

C -1 40.21000 34.14000 -23.78000 L

H -1 41.16000 33.94000 -24.01000 L

C -1 40.00000 34.09000 -22.25000 L

H -1 39.17000 34.60000 -22.03000 L

H -1 40.79000 34.52000 -21.81000 L

C -1 39.85000 32.65000 -21.71000 L

H -1 39.27000 32.08000 -22.30000 L

C -1 41.15000 32.09000 -21.73000 L

H -1 41.11000 31.15000 -21.39000 L

H -1 41.50000 32.09000 -22.67000 L

H -1 41.75000 32.63000 -21.15000 L

C -1 39.32000 32.66000 -20.24000 L

H -1 39.23000 31.72000 -19.91000 L

H -1 39.96000 33.16000 -19.66000 L

H -1 38.42000 33.11000 -20.21000 L

C -1 39.32000 33.12000 -24.45000 L

O -1 39.79000 32.02000 -24.88000 L

N -1 38.02000 33.41000 -24.54000 L

H -1 37.67000 34.30000 -24.28000 L

C -1 37.13000 32.37000 -25.04000 L

H -1 37.45000 31.56000 -24.54000 L

C -1 35.68000 32.65000 -24.73000 L

H -1 35.11000 31.95000 -25.17000 L

H -1 35.44000 33.55000 -25.09000 L

C -1 35.42000 32.62000 -23.21000 L

H -1 36.03000 33.29000 -22.78000 L

C -1 33.95000 33.01000 -22.95000 L

H -1 33.78000 33.00000 -21.96000 L

H -1 33.78000 33.93000 -23.30000 L

H -1 33.35000 32.36000 -23.40000 L

C -1 35.72000 31.21000 -22.61000 L

H -1 35.54000 31.22000 -21.63000 L

H -1 35.12000 30.53000 -23.05000 L

H -1 36.67000 30.97000 -22.78000 L

C -1 37.25000 32.23000 -26.56000 L

O -1 36.97000 31.16000 -27.11000 L

N -1 37.56000 33.32000 -27.25000 L

H -1 37.63000 34.22000 -26.81000 L

C -1 37.80000 33.14000 -28.69000 L

H -1 36.94000 32.79000 -29.06000 L

C -1 38.22000 34.52000 -29.35000 L

H -1 38.97000 34.97000 -28.88000 L

C -1 38.62000 34.35000 -30.87000 L

H -1 38.88000 35.24000 -31.25000 L

H -1 39.40000 33.72000 -30.93000 L

H -1 37.85000 33.98000 -31.38000 L

O -1 37.07000 35.37000 -29.24000 L

H -1 37.27000 36.26000 -29.64000 L

C -1 38.91000 32.11000 -28.92000 L

O -1 38.79000 31.25000 -29.81000 L

N -1 40.00000 32.26000 -28.19000 L

H -1 40.05000 33.01000 -27.54000 L

C -1 41.11000 31.35000 -28.31000 L

H -1 41.39000 31.39000 -29.27000 L

C -1 42.22000 31.83000 -27.40000 L

H -1 41.85000 31.94000 -26.48000 L

H -1 42.55000 32.72000 -27.73000 L

C -1 43.37000 30.83000 -27.38000 L

H -1 43.51000 30.48000 -28.30000 L

H -1 43.14000 30.08000 -26.76000 L

C -1 44.64000 31.56000 -26.88000 L

H -1 44.47000 32.03000 -26.02000 L

H -1 44.96000 32.22000 -27.56000 L

C -1 45.71000 30.59000 -26.66000 L

H -1 45.53000 29.77000 -27.21000 L

H -1 45.73000 30.34000 -25.69000 L

N -1 47.02000 31.12000 -27.03000 L

H -1 47.73000 30.43000 -26.87000 L

H -1 47.02000 31.37000 -28.00000 L

H -1 47.22000 31.94000 -26.49000 L

C -1 40.71000 29.89000 -27.94000 L

O -1 41.03000 28.86000 -28.66000 L

N -1 40.03000 29.77000 -26.79000 L

H -1 39.86000 30.56000 -26.20000 L

C -1 39.53000 28.42000 -26.42000 L

H -1 40.33000 27.83000 -26.33000 L

C -1 38.74000 28.55000 -25.09000 L

H -1 38.09000 29.30000 -25.17000 L

C -1 37.92000 27.28000 -24.75000 L

H -1 37.43000 27.42000 -23.89000 L

H -1 37.26000 27.10000 -25.48000 L

H -1 38.53000 26.49000 -24.66000 L

C -1 39.75000 28.82000 -23.98000 L

H -1 39.27000 28.91000 -23.11000 L

H -1 40.40000 28.06000 -23.93000 L

H -1 40.24000 29.67000 -24.18000 L

C -1 38.68000 27.79000 -27.51000 L

O -1 38.82000 26.58000 -27.88000 L

N -1 37.70000 28.53000 -28.01000 L

H -1 37.55000 29.48000 -27.72000 L

C -1 36.84000 27.91000 -29.01000 L

H -1 36.57000 27.08000 -28.52000 L

C -1 35.63000 28.81000 -29.38000 L

H -1 35.10000 28.37000 -30.11000 L

H -1 35.96000 29.70000 -29.70000 L

C -1 34.72000 29.01000 -28.13000 L

H -1 35.28000 29.28000 -27.35000 L

C -1 33.73000 30.12000 -28.39000 L

H -1 33.15000 30.24000 -27.59000 L

H -1 34.22000 30.97000 -28.59000 L

H -1 33.16000 29.87000 -29.18000 L

C -1 33.94000 27.68000 -27.83000 L

H -1 33.36000 27.81000 -27.02000 L

H -1 33.37000 27.44000 -28.61000 L

H -1 34.59000 26.95000 -27.65000 L

C -1 37.60000 27.58000 -30.33000 L

O -1 37.22000 26.64000 -30.99000 L

N -1 38.55000 28.42000 -30.74000 L

H -1 38.75000 29.25000 -30.22000 L

C -1 39.30000 28.10000 -31.97000 L

H -1 38.63000 28.07000 -32.71000 L

C -1 40.36000 29.17000 -32.27000 L

H -1 40.95000 29.26000 -31.47000 L

H -1 39.89000 30.04000 -32.42000 L

C -1 41.22000 28.75000 -33.58000 L

H -1 40.62000 28.68000 -34.37000 L

H -1 41.67000 27.87000 -33.41000 L

C -1 42.34000 29.80000 -33.92000 L

O -1 42.01000 30.98000 -34.07000 L

N -1 43.64000 29.37000 -34.09000 L

H -1 43.86000 28.40000 -33.99000 L

H -1 44.36000 30.03000 -34.31000 L

C -1 40.05000 26.76000 -31.75000 L

O -1 40.06000 25.85000 -32.62000 L

N -1 40.62000 26.61000 -30.56000 L

H -1 40.56000 27.35000 -29.89000 L

C -1 41.37000 25.37000 -30.22000 L

H -1 42.06000 25.24000 -30.92000 L

C -1 42.06000 25.50000 -28.85000 L

H -1 42.38000 24.61000 -28.55000 L

H -1 41.41000 25.86000 -28.18000 L

C -1 43.25000 26.44000 -28.92000 L

H -1 43.80000 26.37000 -28.10000 L

H -1 42.93000 27.39000 -29.03000 L

C -1 44.05000 26.01000 -30.14000 L

O -1 43.94000 26.69000 -31.25000 L

O -1 44.66000 24.92000 -30.01000 L

C -1 40.40000 24.20000 -30.21000 L

O -1 40.76000 23.14000 -30.71000 L

N -1 39.18000 24.35000 -29.62000 L

H -1 38.95000 25.23000 -29.23000 L

C -1 38.21000 23.26000 -29.56000 L

H -1 38.65000 22.47000 -29.13000 L

C -1 36.98000 23.75000 -28.75000 L

H -1 36.58000 24.54000 -29.21000 L

H -1 37.27000 24.01000 -27.83000 L

C -1 35.90000 22.74000 -28.59000 L

H -1 35.35000 23.01000 -27.80000 L

H -1 36.35000 21.86000 -28.40000 L

C -1 34.97000 22.54000 -29.76000 L

H -1 34.27000 21.87000 -29.51000 L

H -1 35.50000 22.21000 -30.54000 L

N -1 34.28000 23.76000 -30.18000 L

H -1 34.68000 24.27000 -30.95000 L

C -1 33.16000 24.26000 -29.64000 L

N -1 32.60000 25.34000 -30.19000 L

H -1 33.02000 25.76000 -30.99000 L

H -1 31.77000 25.73000 -29.80000 L

N -1 32.56000 23.67000 -28.57000 L

H -1 32.96000 22.84000 -28.17000 L

H -1 31.73000 24.06000 -28.18000 L

C -1 37.79000 22.92000 -30.98000 L

O -1 37.64000 21.74000 -31.33000 L

N -1 37.53000 23.96000 -31.79000 L

H -1 37.69000 24.90000 -31.49000 L

C -1 37.00000 23.68000 -33.16000 L

H -1 36.21000 23.09000 -32.99000 L

C -1 36.63000 25.00000 -33.91000 L

H -1 36.50000 24.81000 -34.88000 L

H -1 37.37000 25.66000 -33.79000 L

C -1 35.34000 25.63000 -33.36000 L

O -1 34.61000 24.93000 -32.63000 L

O -1 35.14000 26.80000 -33.60000 L

C -1 38.07000 22.98000 -34.05000 L

O -1 37.70000 22.36000 -35.07000 L

N -1 39.33000 23.12000 -33.68000 L

H -1 39.54000 23.72000 -32.91000 L

C -1 40.46000 22.41000 -34.37000 L

H -1 41.31000 22.85000 -34.10000 L

H -1 40.33000 22.51000 -35.36000 L

C -1 40.54000 20.90000 -34.02000 L

O -1 41.36000 20.14000 -34.59000 L

N -1 39.74000 20.42000 -33.08000 L

H -1 39.11000 21.02000 -32.58000 L

C -1 39.80000 18.98000 -32.79000 L

H -1 40.76000 18.73000 -32.94000 L

C -1 39.29000 18.68000 -31.31000 L

H -1 39.32000 17.69000 -31.14000 L

H -1 38.36000 19.01000 -31.20000 L

C -1 40.25000 19.43000 -30.30000 L

H -1 40.35000 20.38000 -30.60000 L

C -1 39.62000 19.21000 -28.87000 L

H -1 40.18000 19.66000 -28.18000 L

H -1 38.70000 19.59000 -28.85000 L

H -1 39.58000 18.23000 -28.67000 L

C -1 41.70000 18.98000 -30.28000 L

H -1 42.21000 19.52000 -29.61000 L

H -1 41.74000 18.01000 -30.03000 L

H -1 42.10000 19.10000 -31.18000 L

C -1 38.94000 18.20000 -33.77000 L

O -1 37.85000 18.70000 -34.18000 L

N -1 39.27000 16.93000 -33.98000 L

H -1 40.09000 16.55000 -33.54000 L

C -1 38.44000 16.07000 -34.86000 L

H -1 38.34000 16.55000 -35.73000 L

C -1 39.12000 14.66000 -35.01000 L

H -1 38.50000 14.03000 -35.46000 L

H -1 39.36000 14.30000 -34.10000 L

C -1 40.43000 14.83000 -35.87000 L

H -1 40.94000 15.63000 -35.55000 L

H -1 40.18000 14.95000 -36.83000 L

C -1 41.33000 13.52000 -35.71000 L

H -1 41.53000 13.38000 -34.74000 L

H -1 42.18000 13.64000 -36.22000 L

N -1 40.74000 12.26000 -36.19000 L

H -1 40.17000 11.73000 -35.57000 L

C -1 40.95000 11.79000 -37.42000 L

N -1 40.54000 10.56000 -37.72000 L

H -1 40.08000 10.01000 -37.03000 L

H -1 40.69000 10.20000 -38.64000 L

N -1 41.55000 12.55000 -38.33000 L

H -1 41.84000 13.48000 -38.10000 L

H -1 41.70000 12.20000 -39.26000 L

C -1 37.06000 15.91000 -34.31000 L

O -1 36.88000 15.92000 -33.03000 L

N -1 36.08000 15.74000 -35.22000 L

H -1 36.31000 15.56000 -36.18000 L

C -1 34.68000 15.82000 -34.80000 L

H -1 34.66000 16.74000 -34.43000 L

C -1 33.70000 15.58000 -36.00000 L

H -1 33.37000 16.46000 -36.34000 L

H -1 32.92000 15.03000 -35.70000 L

C -1 34.38000 14.87000 -37.14000 L

H -1 34.25000 13.89000 -36.97000 L

H -1 35.35000 15.09000 -37.06000 L

C -1 33.85000 15.25000 -38.63000 L

H -1 33.05000 14.69000 -38.87000 L

H -1 34.57000 15.12000 -39.31000 L

N -1 33.42000 16.66000 -38.72000 L

H -1 32.60000 16.94000 -38.21000 L

C -1 34.04000 17.58000 -39.43000 L

N -1 35.08000 17.22000 -40.13000 L

H -1 35.39000 16.27000 -40.11000 L

H -1 35.57000 17.90000 -40.68000 L

N -1 33.62000 18.81000 -39.44000 L

H -1 32.81000 19.07000 -38.90000 L

H -1 34.10000 19.50000 -39.98000 L

C -1 34.30000 14.89000 -33.69000 L

O -1 33.60000 15.34000 -32.76000 L

N -1 34.73000 13.63000 -33.70000 L

H -1 35.30000 13.27000 -34.44000 L

C -1 34.33000 12.80000 -32.57000 L

H -1 33.38000 13.11000 -32.51000 L

C -1 34.40000 11.25000 -32.86000 L

H -1 33.87000 10.75000 -32.17000 L

C -1 33.80000 10.94000 -34.21000 L

H -1 33.86000 9.95000 -34.37000 L

H -1 32.85000 11.23000 -34.22000 L

H -1 34.31000 11.42000 -34.92000 L

C -1 35.79000 10.69000 -32.73000 L

H -1 35.77000 9.71000 -32.92000 L

H -1 36.39000 11.15000 -33.38000 L

H -1 36.12000 10.85000 -31.80000 L

C -1 35.02000 13.13000 -31.24000 L

O -1 34.61000 12.62000 -30.23000 L

N -1 36.03000 13.99000 -31.23000 L

H -1 36.32000 14.38000 -32.11000 L

C -1 36.74000 14.39000 -30.03000 L

H -1 36.37000 13.85000 -29.27000 L

C -1 38.25000 14.14000 -30.26000 L

H -1 38.77000 14.49000 -29.48000 L

H -1 38.54000 14.61000 -31.09000 L

C -1 38.56000 12.69000 -30.40000 L

N -1 38.62000 11.84000 -29.32000 L

H -1 38.51000 12.11000 -28.37000 L

C -1 38.86000 10.59000 -29.76000 L

H -1 38.95000 9.78000 -29.18000 L

N -1 38.94000 10.62000 -31.09000 L

C -1 38.75000 11.91000 -31.53000 L

H -1 38.74000 12.22000 -32.48000 L

C -1 36.51000 15.86000 -29.76000 L

O -1 37.34000 16.51000 -29.12000 L

N -1 35.38000 16.38000 -30.21000 L

H -1 34.67000 15.79000 -30.61000 L

C -1 35.17000 17.81000 -30.12000 L

H -1 36.05000 18.23000 -29.88000 L

C -1 34.72000 18.34000 -31.49000 L

H -1 33.79000 18.02000 -31.68000 L

H -1 35.34000 18.00000 -32.20000 L

C -1 34.71000 19.79000 -31.60000 L

H -1 35.64000 20.10000 -31.39000 L

H -1 34.08000 20.12000 -30.90000 L

C -1 34.25000 20.27000 -33.08000 L

H -1 34.22000 21.27000 -33.14000 L

H -1 33.35000 19.90000 -33.32000 L

N -1 35.17000 19.84000 -34.12000 L

H -1 36.13000 19.75000 -33.88000 L

C -1 34.83000 19.54000 -35.39000 L

N -1 33.56000 19.57000 -35.79000 L

H -1 32.84000 19.81000 -35.15000 L

H -1 33.34000 19.34000 -36.74000 L

N -1 35.75000 19.18000 -36.28000 L

H -1 36.71000 19.12000 -36.01000 L

H -1 35.48000 18.96000 -37.22000 L

C -1 34.12000 18.09000 -29.00000 L

O -1 32.94000 17.86000 -29.19000 L

N -1 34.57000 18.59000 -27.82000 L

C -1 35.95000 18.95000 -27.49000 L

H -1 36.29000 19.62000 -28.16000 L

H -1 36.53000 18.14000 -27.50000 L

C -1 35.80000 19.57000 -26.06000 L

H -1 35.62000 20.55000 -26.10000 L

H -1 36.61000 19.40000 -25.51000 L

C -1 34.61000 18.85000 -25.49000 L

H -1 34.21000 19.36000 -24.73000 L

H -1 34.87000 17.93000 -25.18000 L

C -1 33.64000 18.77000 -26.68000 L

H -1 32.97000 18.03000 -26.64000 L

C -1 32.84000 20.09000 -26.78000 L

O -1 33.32000 21.04000 -27.40000 L

N -1 31.65000 20.13000 -26.16000 L

H -1 31.19000 19.28000 -25.91000 L

C -1 31.01000 21.44000 -25.83000 L

H -1 30.88000 21.95000 -26.68000 L

C -1 29.64000 21.21000 -25.14000 L

H -1 29.22000 22.09000 -24.93000 L

H -1 29.04000 20.70000 -25.76000 L

H -1 29.78000 20.69000 -24.30000 L

C -1 31.94000 22.22000 -24.89000 L

O -1 32.75000 21.62000 -24.17000 L

N -1 31.85000 23.55000 -24.94000 L

H -1 31.25000 23.97000 -25.62000 L

C -1 32.61000 24.43000 -24.02000 L

H -1 33.15000 23.82000 -23.43000 L

C -1 33.56000 25.37000 -24.80000 L

H -1 32.93000 25.84000 -25.43000 L

C -1 34.24000 26.52000 -23.92000 L

H -1 34.82000 27.08000 -24.50000 L

H -1 33.52000 27.09000 -23.51000 L

H -1 34.78000 26.10000 -23.19000 L

C -1 34.76000 24.49000 -25.56000 L

H -1 35.36000 25.11000 -26.06000 L

H -1 35.29000 24.00000 -24.87000 L

H -1 34.34000 23.84000 -26.19000 L

C -1 31.64000 25.26000 -23.18000 L

O -1 30.81000 26.00000 -23.72000 L

N -1 31.79000 25.18000 -21.84000 L

H -1 32.46000 24.53000 -21.46000 L

C -1 31.01000 26.01000 -20.92000 L

H -1 30.37000 26.56000 -21.46000 L

C -1 30.31000 25.09000 -19.95000 L

H -1 29.93000 25.64000 -19.21000 L

H -1 30.99000 24.46000 -19.58000 L

C -1 29.15000 24.26000 -20.62000 L

H -1 28.68000 24.83000 -21.29000 L

C -1 29.72000 22.98000 -21.34000 L

H -1 28.97000 22.47000 -21.76000 L

H -1 30.37000 23.26000 -22.05000 L

H -1 30.19000 22.40000 -20.67000 L

C -1 28.22000 23.78000 -19.46000 L

H -1 27.46000 23.25000 -19.84000 L

H -1 28.74000 23.21000 -18.83000 L

H -1 27.85000 24.58000 -18.98000 L

C -1 31.96000 26.91000 -20.13000 L

O -1 33.15000 26.61000 -20.00000 L

N -1 31.40000 27.98000 -19.58000 L

H -1 30.42000 28.14000 -19.70000 L

C -1 32.20000 28.94000 -18.77000 L

H -1 33.16000 28.69000 -18.80000 L

C -1 32.13000 30.41000 -19.33000 L

H -1 31.18000 30.70000 -19.41000 L

C -1 32.94000 31.34000 -18.37000 L

H -1 32.91000 32.28000 -18.71000 L

H -1 32.54000 31.31000 -17.45000 L

H -1 33.89000 31.04000 -18.33000 L

C -1 32.79000 30.46000 -20.72000 L

H -1 32.75000 31.39000 -21.07000 L

H -1 33.75000 30.17000 -20.64000 L

H -1 32.31000 29.84000 -21.34000 L

C -1 31.58000 28.84000 -17.37000 L

O -1 30.34000 28.79000 -17.23000 L

N -1 32.41000 28.56000 -16.37000 L

H -1 33.38000 28.38000 -16.53000 L

C -1 31.87000 28.54000 -15.02000 L

H -1 30.90000 28.35000 -15.12000 L

C -1 32.62000 27.41000 -14.22000 L

H -1 33.60000 27.62000 -14.18000 L

H -1 32.49000 26.53000 -14.68000 L

C -1 32.10000 27.29000 -12.82000 L

H -1 31.10000 27.35000 -12.83000 L

H -1 32.47000 28.04000 -12.27000 L

C -1 32.52000 25.97000 -12.21000 L

H -1 33.52000 25.94000 -12.17000 L

H -1 32.20000 25.23000 -12.81000 L

C -1 31.87000 25.84000 -10.72000 L

H -1 30.87000 25.85000 -10.75000 L

H -1 32.18000 26.57000 -10.12000 L

N -1 32.35000 24.47000 -10.16000 L

H -1 31.99000 24.34000 -9.25000 L

H -1 32.04000 23.74000 -10.77000 L

H -1 33.35000 24.47000 -10.13000 L

C -1 32.12000 29.93000 -14.32000 L

O -1 33.25000 30.40000 -14.21000 L

N -1 31.06000 30.53000 -13.75000 L

H -1 30.19000 30.03000 -13.69000 L

C -1 31.14000 31.86000 -13.22000 L

H -1 32.09000 32.11000 -13.39000 L

C -1 30.10000 32.76000 -13.93000 L

H -1 30.18000 33.70000 -13.61000 L

C -1 30.40000 32.73000 -15.49000 L

H -1 29.73000 33.30000 -15.97000 L

H -1 31.32000 33.07000 -15.66000 L

H -1 30.32000 31.79000 -15.82000 L

C -1 28.63000 32.30000 -13.56000 L

H -1 28.50000 32.35000 -12.57000 L

H -1 28.48000 31.37000 -13.88000 L

C -1 27.62000 33.28000 -14.29000 L

H -1 26.68000 33.01000 -14.07000 L

H -1 27.78000 34.22000 -13.97000 L

H -1 27.75000 33.24000 -15.28000 L

C -1 30.88000 31.92000 -11.71000 L

O -1 30.27000 31.03000 -11.10000 L

N -1 31.35000 32.99000 -11.14000 L

H -1 31.83000 33.67000 -11.70000 L

C -1 31.18000 33.22000 -9.72000 L

H -1 31.39000 32.35000 -9.29000 L

C -1 32.13000 34.47000 -9.36000 L

H -1 32.05000 34.67000 -8.38000 L

H -1 33.08000 34.25000 -9.57000 L

H -1 31.84000 35.27000 -9.88000 L

C -1 29.75000 33.62000 -9.37000 L

O -1 28.96000 34.09000 -10.21000 L

N -1 29.38000 33.50000 -8.08000 L

C -1 30.11000 32.74000 -7.07000 L

H -1 31.02000 33.14000 -6.93000 L

H -1 30.21000 31.79000 -7.36000 L

C -1 29.23000 32.87000 -5.81000 L

H -1 29.57000 33.60000 -5.22000 L

H -1 29.22000 32.00000 -5.31000 L

C -1 27.79000 33.22000 -6.35000 L

H -1 27.28000 33.77000 -5.69000 L

H -1 27.27000 32.39000 -6.57000 L

C -1 28.10000 34.04000 -7.65000 L

H -1 27.46000 33.97000 -8.41000 L

C -1 28.29000 35.52000 -7.18000 L

O -1 27.34000 36.11000 -6.67000 L

N -1 29.47000 36.07000 -7.29000 L

H -1 30.20000 35.58000 -7.78000 L

C -1 29.75000 37.39000 -6.69000 L

H -1 28.97000 37.59000 -6.09000 L

C -1 31.05000 37.31000 -5.89000 L

H -1 31.22000 38.20000 -5.46000 L

H -1 31.80000 37.08000 -6.51000 L

C -1 30.95000 36.21000 -4.78000 L

O -1 29.88000 36.12000 -4.14000 L

O -1 31.93000 35.46000 -4.62000 L

C -1 29.82000 38.53000 -7.75000 L

O -1 30.46000 39.57000 -7.57000 L

N -1 29.21000 38.35000 -8.89000 L

H -1 28.56000 37.60000 -9.01000 L

C -1 29.49000 39.30000 -10.02000 L

H -1 30.44000 39.59000 -9.95000 L

C -1 29.11000 38.62000 -11.35000 L

H -1 29.27000 39.27000 -12.09000 L

H -1 28.13000 38.40000 -11.31000 L

C -1 29.89000 37.31000 -11.67000 L

H -1 29.82000 36.70000 -10.87000 L

C -1 29.30000 36.46000 -12.85000 L

H -1 29.86000 35.64000 -12.98000 L

H -1 28.36000 36.19000 -12.63000 L

H -1 29.30000 37.01000 -13.68000 L

C -1 31.34000 37.66000 -11.89000 L

H -1 31.86000 36.83000 -12.10000 L

H -1 31.41000 38.30000 -12.66000 L

H -1 31.71000 38.09000 -11.07000 L

C -1 28.55000 40.45000 -9.91000 L

O -1 27.44000 40.29000 -9.46000 L

N -1 28.97000 41.62000 -10.41000 L

H -1 29.92000 41.72000 -10.72000 L

C -1 28.03000 42.74000 -10.49000 L

H -1 27.59000 42.76000 -9.59000 L

C -1 28.76000 44.04000 -10.76000 L

H -1 28.09000 44.78000 -10.84000 L

C -1 29.90000 44.29000 -9.65000 L

H -1 30.37000 45.15000 -9.85000 L

H -1 29.48000 44.34000 -8.74000 L

H -1 30.56000 43.54000 -9.67000 L

O -1 29.39000 43.95000 -12.02000 L

H -1 29.88000 44.81000 -12.21000 L

C -1 26.96000 42.52000 -11.55000 L

O -1 27.09000 41.68000 -12.43000 L

N -1 25.92000 43.34000 -11.55000 L

H -1 25.79000 44.01000 -10.83000 L

C -1 24.96000 43.22000 -12.65000 L

H -1 24.62000 42.28000 -12.63000 L

C -1 23.81000 44.22000 -12.47000 L

H -1 23.11000 44.12000 -13.17000 L

H -1 24.14000 45.17000 -12.46000 L

O -1 23.23000 43.92000 -11.20000 L

H -1 22.47000 44.55000 -11.03000 L

C -1 25.68000 43.51000 -13.97000 L

O -1 25.33000 42.88000 -15.01000 L

N -1 26.61000 44.46000 -13.98000 L

H -1 26.83000 44.95000 -13.14000 L

C -1 27.32000 44.78000 -15.26000 L

H -1 26.62000 45.01000 -15.94000 L

C -1 28.24000 45.96000 -15.00000 L

H -1 28.84000 45.75000 -14.23000 L

H -1 27.69000 46.77000 -14.78000 L

C -1 29.07000 46.28000 -16.15000 L

H -1 29.43000 45.42000 -16.52000 L

H -1 29.83000 46.84000 -15.83000 L

C -1 28.35000 47.00000 -17.21000 L

O -1 28.24000 48.26000 -17.19000 L

N -1 27.96000 46.26000 -18.25000 L

H -1 28.16000 45.28000 -18.26000 L

H -1 27.46000 46.68000 -19.01000 L

C -1 28.15000 43.57000 -15.79000 L

O -1 28.25000 43.28000 -17.02000 L

N -1 28.78000 42.85000 -14.84000 L

H -1 28.67000 43.09000 -13.88000 L

C -1 29.61000 41.71000 -15.19000 L

H -1 30.30000 42.10000 -15.79000 L

C -1 30.21000 41.02000 -13.91000 L

H -1 30.61000 40.15000 -14.18000 L

H -1 29.47000 40.86000 -13.25000 L

C -1 31.29000 41.85000 -13.20000 L

O -1 31.96000 42.65000 -13.85000 L

O -1 31.48000 41.62000 -11.96000 L

C -1 28.72000 40.62000 -15.82000 L

O -1 29.05000 40.00000 -16.88000 L

N -1 27.56000 40.42000 -15.23000 L

H -1 27.30000 40.96000 -14.42000 L

C -1 26.64000 39.40000 -15.75000 L

H -1 27.17000 38.55000 -15.79000 L

C -1 25.44000 39.21000 -14.80000 L

H -1 24.74000 38.68000 -15.27000 L

H -1 25.07000 40.11000 -14.57000 L

C -1 25.92000 38.45000 -13.48000 L

H -1 26.66000 38.96000 -13.04000 L

H -1 26.24000 37.53000 -13.70000 L

C -1 24.76000 38.33000 -12.48000 L

H -1 24.69000 37.39000 -12.16000 L

H -1 23.90000 38.59000 -12.92000 L

C -1 25.08000 39.29000 -11.29000 L

H -1 24.97000 40.24000 -11.58000 L

H -1 26.01000 39.14000 -10.97000 L

N -1 24.14000 39.04000 -10.16000 L

H -1 24.35000 39.66000 -9.41000 L

H -1 23.20000 39.19000 -10.46000 L

H -1 24.24000 38.09000 -9.85000 L

C -1 26.15000 39.77000 -17.16000 L

O -1 26.06000 38.89000 -18.06000 L

N -1 25.76000 41.04000 -17.35000 L

H -1 25.72000 41.66000 -16.57000 L

C -1 25.38000 41.52000 -18.70000 L

H -1 24.54000 41.00000 -18.88000 L

C -1 25.21000 43.07000 -18.75000 L

H -1 25.17000 43.38000 -19.70000 L

H -1 25.97000 43.51000 -18.29000 L

C -1 24.00000 43.54000 -18.11000 L

H -1 24.01000 43.20000 -17.17000 L

H -1 23.23000 43.14000 -18.61000 L

C -1 23.88000 45.09000 -18.11000 L

O -1 23.06000 45.44000 -17.28000 L

O -1 24.60000 45.87000 -18.87000 L

C -1 26.48000 41.29000 -19.72000 L

O -1 26.15000 40.86000 -20.84000 L

N -1 27.73000 41.63000 -19.37000 L

H -1 27.88000 42.02000 -18.46000 L

C -1 28.88000 41.46000 -20.27000 L

H -1 28.63000 41.96000 -21.10000 L

C -1 30.17000 42.05000 -19.67000 L

H -1 30.94000 41.78000 -20.25000 L

H -1 30.29000 41.67000 -18.75000 L

C -1 30.10000 43.59000 -19.59000 L

O -1 29.14000 44.11000 -20.16000 L

O -1 30.94000 44.18000 -18.98000 L

C -1 29.09000 39.99000 -20.58000 L

O -1 29.34000 39.63000 -21.72000 L

N -1 29.02000 39.13000 -19.56000 L

H -1 28.89000 39.45000 -18.63000 L

C -1 29.13000 37.68000 -19.84000 L

H -1 29.99000 37.62000 -20.34000 L

C -1 29.17000 36.84000 -18.50000 L

H -1 28.41000 37.11000 -17.91000 L

C -1 28.99000 35.32000 -18.78000 L

H -1 29.02000 34.82000 -17.91000 L

H -1 28.11000 35.15000 -19.22000 L

H -1 29.73000 35.00000 -19.37000 L

C -1 30.53000 37.12000 -17.81000 L

H -1 30.78000 38.07000 -17.98000 L

H -1 31.22000 36.52000 -18.21000 L

C -1 30.41000 36.86000 -16.19000 L

H -1 31.30000 37.05000 -15.76000 L

H -1 29.72000 37.47000 -15.80000 L

H -1 30.16000 35.91000 -16.02000 L

C -1 28.06000 37.14000 -20.79000 L

O -1 28.35000 36.37000 -21.74000 L

N -1 26.82000 37.53000 -20.57000 L

H -1 26.63000 38.16000 -19.81000 L

C -1 25.76000 37.08000 -21.40000 L

H -1 25.75000 36.09000 -21.31000 L

C -1 24.40000 37.65000 -20.94000 L

H -1 23.68000 37.32000 -21.55000 L

H -1 24.22000 37.36000 -20.00000 L

H -1 24.43000 38.65000 -20.98000 L

C -1 26.04000 37.51000 -22.83000 L

O -1 25.88000 36.72000 -23.72000 L

N -1 26.49000 38.75000 -23.01000 L

H -1 26.68000 39.33000 -22.22000 L

C -1 26.72000 39.27000 -24.34000 L

H -1 25.86000 39.16000 -24.84000 L

C -1 27.10000 40.75000 -24.20000 L

H -1 27.79000 40.86000 -23.49000 L

H -1 26.29000 41.29000 -23.96000 L

O -1 27.64000 41.27000 -25.43000 L

H -1 27.88000 42.23000 -25.31000 L

C -1 27.89000 38.49000 -25.05000 L

O -1 27.80000 38.11000 -26.21000 L

N -1 28.96000 38.28000 -24.35000 L

H -1 29.02000 38.63000 -23.41000 L

C -1 30.10000 37.53000 -24.90000 L

H -1 30.35000 37.99000 -25.76000 L

C -1 31.31000 37.61000 -23.93000 L

H -1 30.97000 37.35000 -23.03000 L

C -1 32.53000 36.65000 -24.48000 L

H -1 33.31000 36.70000 -23.85000 L

H -1 32.21000 35.70000 -24.54000 L

H -1 32.82000 36.96000 -25.39000 L

C -1 31.82000 39.05000 -23.83000 L

H -1 32.60000 39.09000 -23.20000 L

H -1 32.11000 39.37000 -24.73000 L

H -1 31.09000 39.65000 -23.48000 L

C -1 29.68000 36.11000 -25.28000 L

O -1 29.97000 35.60000 -26.38000 L

N -1 28.93000 35.44000 -24.41000 L

H -1 28.62000 35.91000 -23.58000 L

C -1 28.55000 34.08000 -24.62000 L

H -1 29.39000 33.55000 -24.76000 L

C -1 27.76000 33.64000 -23.36000 L

H -1 27.18000 34.36000 -22.98000 L

C -1 26.75000 32.57000 -23.72000 L

H -1 26.25000 32.30000 -22.90000 L

H -1 26.11000 32.93000 -24.40000 L

H -1 27.23000 31.78000 -24.10000 L

C -1 28.80000 33.26000 -22.30000 L

H -1 28.34000 32.96000 -21.46000 L

H -1 29.37000 32.51000 -22.64000 L

H -1 29.38000 34.05000 -22.09000 L

C -1 27.67000 33.98000 -25.86000 L

O -1 27.81000 33.05000 -26.70000 L

N -1 26.79000 34.95000 -26.04000 L

H -1 26.69000 35.69000 -25.37000 L

C -1 25.96000 34.93000 -27.26000 L

H -1 25.70000 33.97000 -27.39000 L

C -1 24.68000 35.80000 -27.08000 L

H -1 24.23000 35.92000 -27.96000 L

H -1 24.94000 36.69000 -26.71000 L

C -1 23.73000 34.99000 -26.05000 L

H -1 24.23000 34.83000 -25.19000 L

H -1 23.47000 34.12000 -26.45000 L

C -1 22.42000 35.76000 -25.69000 L

H -1 21.95000 35.30000 -24.93000 L

H -1 21.82000 35.79000 -26.48000 L

C -1 22.69000 37.18000 -25.26000 L

H -1 23.36000 37.60000 -25.88000 L

H -1 23.05000 37.20000 -24.33000 L

N -1 21.37000 37.92000 -25.34000 L

H -1 21.50000 38.87000 -25.07000 L

H -1 21.02000 37.89000 -26.28000 L

H -1 20.70000 37.49000 -24.73000 L

C -1 26.74000 35.31000 -28.51000 L

O -1 26.57000 34.66000 -29.55000 L

N -1 27.60000 36.31000 -28.43000 L

H -1 27.69000 36.80000 -27.56000 L

C -1 28.43000 36.73000 -29.56000 L

H -1 27.81000 37.07000 -30.27000 L

C -1 29.35000 37.82000 -29.08000 L

H -1 29.85000 37.46000 -28.29000 L

H -1 28.77000 38.58000 -28.78000 L

C -1 30.36000 38.34000 -30.10000 L

H -1 29.88000 38.84000 -30.82000 L

H -1 30.86000 37.57000 -30.50000 L

C -1 31.35000 39.26000 -29.47000 L

O -1 31.13000 39.67000 -28.31000 L

O -1 32.37000 39.58000 -30.10000 L

C -1 29.33000 35.52000 -30.06000 L

O -1 29.53000 35.30000 -31.30000 L

N -1 29.86000 34.72000 -29.10000 L

H -1 29.60000 34.83000 -28.14000 L

C -1 30.83000 33.69000 -29.48000 L

H -1 31.19000 34.00000 -30.36000 L

C -1 31.95000 33.61000 -28.45000 L

H -1 32.54000 32.85000 -28.69000 L

H -1 31.54000 33.45000 -27.55000 L

C -1 32.82000 34.86000 -28.34000 L

H -1 32.15000 35.52000 -28.01000 L

C -1 34.00000 34.68000 -27.27000 L

H -1 34.54000 35.52000 -27.23000 L

H -1 33.61000 34.49000 -26.37000 L

H -1 34.58000 33.92000 -27.55000 L

C -1 33.40000 35.41000 -29.72000 L

H -1 33.95000 36.22000 -29.55000 L

H -1 33.96000 34.70000 -30.15000 L

H -1 32.64000 35.65000 -30.32000 L

C -1 30.18000 32.33000 -29.72000 L

O -1 30.83000 31.43000 -30.25000 L

N -1 28.93000 32.18000 -29.34000 L

H -1 28.42000 32.96000 -28.98000 L

C -1 28.27000 30.89000 -29.45000 L

H -1 28.43000 30.54000 -30.38000 L

H -1 27.29000 31.03000 -29.31000 L

C -1 28.85000 29.91000 -28.38000 L

O -1 28.90000 28.66000 -28.61000 L

N -1 29.29000 30.43000 -27.24000 L

H -1 29.41000 31.41000 -27.15000 L

C -1 29.59000 29.50000 -26.08000 L

H -1 30.39000 28.94000 -26.31000 L

C -1 29.90000 30.34000 -24.78000 L

H -1 29.20000 31.04000 -24.63000 L

C -1 29.96000 29.48000 -23.50000 L

H -1 30.16000 30.07000 -22.71000 L

H -1 29.07000 29.03000 -23.37000 L

H -1 30.67000 28.79000 -23.59000 L

C -1 31.28000 30.95000 -24.99000 L

H -1 31.38000 31.69000 -24.33000 L

H -1 31.30000 31.32000 -25.91000 L

C -1 32.48000 29.79000 -24.78000 L

H -1 33.38000 30.21000 -24.92000 L

H -1 32.43000 29.42000 -23.85000 L

H -1 32.35000 29.05000 -25.44000 L

C -1 28.45000 28.50000 -25.89000 L

O -1 27.27000 28.87000 -25.92000 L

N -1 28.75000 27.24000 -25.61000 L

H -1 29.70000 26.97000 -25.48000 L

C -1 27.70000 26.24000 -25.50000 L

H -1 27.09000 26.52000 -26.25000 L

C -1 28.31000 24.84000 -25.64000 L

H -1 27.57000 24.16000 -25.60000 L

H -1 28.93000 24.69000 -24.87000 L

C -1 29.12000 24.69000 -27.02000 L

O -1 28.44000 24.86000 -28.06000 L

O -1 30.36000 24.56000 -26.98000 L

C -1 26.89000 26.23000 -24.19000 L

O -1 25.79000 25.70000 -24.18000 L

N -1 27.45000 26.75000 -23.09000 L

H -1 28.36000 27.14000 -23.13000 L

C -1 26.71000 26.73000 -21.85000 L

H -1 26.56000 25.77000 -21.59000 L

H -1 25.83000 27.18000 -22.00000 L

C -1 27.47000 27.44000 -20.75000 L

O -1 28.59000 27.85000 -20.94000 L

N -1 26.84000 27.56000 -19.59000 L

H -1 25.91000 27.19000 -19.53000 L

C -1 27.39000 28.21000 -18.42000 L

H -1 28.34000 28.42000 -18.60000 L

C -1 26.50000 29.46000 -18.10000 L

H -1 26.76000 29.81000 -17.20000 L

H -1 25.54000 29.17000 -18.08000 L

C -1 26.66000 30.60000 -19.14000 L

H -1 26.56000 30.21000 -20.06000 L

C -1 25.57000 31.71000 -18.90000 L

H -1 25.68000 32.43000 -19.58000 L

H -1 24.66000 31.31000 -18.99000 L

H -1 25.68000 32.09000 -17.98000 L

C -1 28.04000 31.25000 -19.02000 L

H -1 28.13000 31.98000 -19.70000 L

H -1 28.15000 31.63000 -18.10000 L

H -1 28.75000 30.56000 -19.18000 L

C -1 27.22000 27.26000 -17.24000 L

O -1 26.24000 26.54000 -17.12000 L

N -1 28.18000 27.31000 -16.34000 L

H -1 29.01000 27.83000 -16.52000 L

C -1 27.99000 26.61000 -15.10000 L

H -1 27.18000 26.04000 -15.20000 L

C -1 29.20000 25.71000 -14.80000 L

H -1 30.04000 26.25000 -14.78000 L

C -1 28.96000 25.01000 -13.38000 L

H -1 29.74000 24.41000 -13.16000 L

H -1 28.87000 25.71000 -12.67000 L

H -1 28.12000 24.46000 -13.41000 L

C -1 29.30000 24.64000 -15.95000 L

H -1 29.75000 25.06000 -16.74000 L

H -1 28.37000 24.37000 -16.21000 L

C -1 30.09000 23.35000 -15.58000 L

H -1 30.11000 22.73000 -16.36000 L

H -1 31.03000 23.60000 -15.34000 L

H -1 29.65000 22.90000 -14.80000 L

C -1 27.89000 27.72000 -14.00000 L

O -1 28.74000 28.64000 -13.93000 L

N -1 26.84000 27.61000 -13.17000 L

H -1 26.32000 26.76000 -13.12000 L

C -1 26.46000 28.79000 -12.31000 L

H -1 27.20000 29.45000 -12.18000 L

C -1 25.23000 29.46000 -13.01000 L

H -1 24.48000 28.80000 -12.90000 L

C -1 24.76000 30.76000 -12.29000 L

H -1 23.98000 31.15000 -12.78000 L

H -1 24.50000 30.55000 -11.35000 L

H -1 25.51000 31.43000 -12.29000 L

C -1 25.54000 29.79000 -14.57000 L

H -1 24.74000 30.22000 -14.99000 L

H -1 26.32000 30.42000 -14.63000 L

H -1 25.76000 28.94000 -15.05000 L

C -1 25.99000 28.14000 -10.99000 L

O -1 24.89000 27.58000 -10.94000 L

N -1 26.79000 28.20000 -9.91000 L

H -1 26.55000 27.62000 -9.13000 L

C -1 27.97000 29.05000 -9.80000 L

H -1 28.16000 29.25000 -10.76000 L

C -1 27.74000 30.28000 -8.85000 L

H -1 28.59000 30.81000 -8.79000 L

C -1 26.45000 31.07000 -9.35000 L

H -1 26.29000 31.85000 -8.76000 L

H -1 26.60000 31.38000 -10.29000 L

H -1 25.66000 30.46000 -9.33000 L

O -1 27.42000 29.86000 -7.50000 L

H -1 27.28000 30.67000 -6.92000 L

C -1 29.07000 28.35000 -9.09000 L

O -1 28.86000 27.31000 -8.47000 L

N -1 30.24000 29.00000 -9.11000 L

H -1 30.38000 29.75000 -9.76000 L

C -1 31.33000 28.62000 -8.18000 L

H -1 31.39000 27.62000 -8.21000 L

C -1 32.65000 29.30000 -8.68000 L

H -1 32.80000 30.14000 -8.17000 L

H -1 32.57000 29.50000 -9.65000 L

C -1 33.88000 28.38000 -8.47000 L

O -1 34.10000 27.85000 -7.35000 L

N -1 34.64000 28.15000 -9.56000 L

H -1 34.41000 28.56000 -10.43000 L

H -1 35.45000 27.56000 -9.48000 L

C -1 31.00000 28.99000 -6.70000 L

O -1 29.89000 29.43000 -6.35000 L

N -1 32.00000 28.85000 -5.82000 L

H -1 32.87000 28.47000 -6.10000 L

C -1 31.80000 29.26000 -4.44000 L

H -1 30.88000 28.92000 -4.24000 L

C -1 32.93000 28.64000 -3.54000 L

H -1 32.91000 28.98000 -2.60000 L

C -1 32.81000 27.14000 -3.48000 L

H -1 33.54000 26.77000 -2.90000 L

H -1 31.92000 26.89000 -3.10000 L

H -1 32.90000 26.77000 -4.40000 L

O -1 34.22000 28.98000 -4.11000 L

H -1 34.95000 28.59000 -3.55000 L

C -1 31.85000 30.81000 -4.29000 L

O -1 32.30000 31.55000 -5.22000 L

N -1 31.38000 31.31000 -3.14000 L

H -1 31.14000 30.69000 -2.40000 L

C -1 31.23000 32.75000 -2.94000 L

H -1 31.31000 33.14000 -3.85000 L

C -1 29.83000 33.15000 -2.38000 L

H -1 29.13000 32.94000 -3.06000 L

C -1 29.55000 32.52000 -0.99000 L

H -1 28.64000 32.80000 -0.67000 L

H -1 29.58000 31.52000 -1.06000 L

H -1 30.24000 32.83000 -0.34000 L

O -1 29.85000 34.51000 -2.12000 L

H -1 28.96000 34.79000 -1.76000 L

C -1 32.34000 33.24000 -1.97000 L

O -1 32.69000 32.57000 -0.94000 L

N -1 32.88000 34.41000 -2.26000 L

H -1 32.66000 34.86000 -3.12000 L

C -1 33.79000 35.03000 -1.32000 L

H -1 34.22000 34.24000 -0.87000 L

C -1 34.89000 35.92000 -2.01000 L

H -1 35.50000 36.29000 -1.31000 L

C -1 35.75000 35.03000 -2.88000 L

H -1 36.45000 35.58000 -3.32000 L

H -1 36.18000 34.33000 -2.31000 L

H -1 35.17000 34.59000 -3.57000 L

C -1 34.25000 37.14000 -2.86000 L

H -1 34.99000 37.68000 -3.28000 L

H -1 33.66000 36.78000 -3.58000 L

H -1 33.72000 37.73000 -2.26000 L

C -1 32.97000 35.89000 -0.34000 L

O -1 33.57000 36.49000 0.61000 L

N -1 31.65000 35.92000 -0.47000 L

H -1 31.20000 35.44000 -1.22000 L

C -1 30.84000 36.69000 0.51000 L

H -1 31.33000 37.54000 0.71000 L

C -1 29.41000 36.97000 -0.10000 L

H -1 29.48000 37.30000 -1.05000 L

H -1 28.91000 37.64000 0.44000 L

O -1 28.63000 35.75000 -0.12000 L

H -1 27.73000 35.93000 -0.51000 L

C -1 30.70000 35.86000 1.80000 L

O -1 30.99000 34.66000 1.78000 L

N -1 30.25000 36.45000 2.93000 L

H -1 30.06000 37.43000 2.93000 L

C -1 30.03000 35.67000 4.16000 L

H -1 30.06000 34.69000 3.97000 L

C -1 31.13000 35.93000 5.24000 L

H -1 30.88000 35.49000 6.10000 L

H -1 31.24000 36.92000 5.40000 L

C -1 32.44000 35.39000 4.80000 L

H -1 33.14000 35.74000 5.43000 L

H -1 32.62000 35.74000 3.88000 L

C -1 32.47000 33.82000 4.78000 L

H -1 31.85000 33.47000 4.09000 L

H -1 32.21000 33.46000 5.68000 L

N -1 33.84000 33.32000 4.47000 L

H -1 34.45000 33.18000 5.24000 L

C -1 34.32000 33.06000 3.26000 L

N -1 35.62000 32.61000 3.08000 L

H -1 36.21000 32.48000 3.88000 L

H -1 35.96000 32.41000 2.17000 L

N -1 33.53000 33.25000 2.18000 L

H -1 32.60000 33.59000 2.30000 L

H -1 33.88000 33.05000 1.26000 L

C -1 28.64000 36.21000 4.60000 L

O -1 28.58000 37.21000 5.31000 L

N -1 27.54000 35.57000 4.12000 L

C -1 27.54000 34.44000 3.19000 L

H -1 27.96000 33.65000 3.62000 L

H -1 28.06000 34.69000 2.37000 L

C -1 26.00000 34.24000 2.91000 L

H -1 25.76000 33.27000 2.84000 L

H -1 25.71000 34.72000 2.08000 L

C -1 25.31000 34.82000 4.06000 L

H -1 25.28000 34.17000 4.82000 L

H -1 24.38000 35.08000 3.81000 L

C -1 26.17000 36.05000 4.40000 L

H -1 25.95000 36.89000 3.90000 L

C -1 26.05000 36.30000 5.86000 L

O -1 26.61000 35.56000 6.67000 L

N -1 25.23000 37.29000 6.23000 L

H -1 24.83000 37.89000 5.54000 L

C -1 24.89000 37.52000 7.66000 L

H -1 25.71000 37.73000 8.19000 L

C -1 23.93000 38.72000 7.77000 L

H -1 23.71000 38.88000 8.74000 L

H -1 24.37000 39.54000 7.40000 L

H -1 23.09000 38.53000 7.26000 L

C -1 24.24000 36.23000 8.13000 L

O -1 23.53000 35.60000 7.35000 L

N -1 24.55000 35.79000 9.35000 L

H -1 25.29000 36.26000 9.84000 L

C -1 23.90000 34.69000 10.01000 L

H -1 23.07000 34.49000 9.49000 L

H -1 23.65000 34.99000 10.93000 L

C -1 24.77000 33.45000 10.09000 L

O -1 24.36000 32.47000 10.69000 L

N -1 25.90000 33.42000 9.38000 L

H -1 26.13000 34.15000 8.74000 L

C -1 26.83000 32.24000 9.55000 L

H -1 26.32000 31.43000 9.24000 L

C -1 28.12000 32.38000 8.74000 L

H -1 28.69000 31.58000 8.95000 L

H -1 28.58000 33.21000 9.06000 L

C -1 27.98000 32.48000 7.18000 L

H -1 27.34000 33.22000 7.01000 L

C -1 29.35000 32.75000 6.51000 L

H -1 29.23000 32.81000 5.52000 L

H -1 29.72000 33.62000 6.85000 L

H -1 29.99000 32.01000 6.73000 L

C -1 27.50000 31.11000 6.57000 L

H -1 27.42000 31.19000 5.58000 L

H -1 28.16000 30.39000 6.79000 L

H -1 26.61000 30.86000 6.96000 L

C -1 27.17000 32.05000 10.99000 L

O -1 27.58000 32.99000 11.65000 L

N -1 27.10000 30.80000 11.47000 L

H -1 26.85000 30.04000 10.87000 L

C -1 27.40000 30.56000 12.89000 L

H -1 27.48000 31.48000 13.27000 L

C -1 26.26000 29.62000 13.52000 L

H -1 26.47000 29.42000 14.47000 L

H -1 26.20000 28.77000 13.00000 L

C -1 24.90000 30.28000 13.47000 L

H -1 24.24000 29.70000 13.93000 L

H -1 24.63000 30.41000 12.52000 L

C -1 24.98000 31.64000 14.18000 L

O -1 25.39000 31.65000 15.30000 L

N -1 24.64000 32.75000 13.50000 L

H -1 24.34000 32.68000 12.55000 L

H -1 24.68000 33.64000 13.95000 L

C -1 28.71000 29.78000 13.08000 L

O -1 29.25000 29.80000 14.21000 L

N -1 29.22000 29.11000 12.03000 L

H -1 28.83000 29.20000 11.11000 L

C -1 30.37000 28.23000 12.27000 L

H -1 30.63000 27.79000 11.41000 L

H -1 30.10000 27.53000 12.93000 L

C -1 31.56000 29.05000 12.82000 L

O -1 31.74000 30.22000 12.49000 L

N -1 32.34000 28.43000 13.72000 L

H -1 32.16000 27.48000 13.97000 L

C -1 33.49000 29.17000 14.34000 L

H -1 33.14000 30.00000 14.76000 L

C -1 34.19000 28.20000 15.42000 L

H -1 34.96000 28.68000 15.85000 L

H -1 33.52000 27.96000 16.13000 L

H -1 34.52000 27.37000 14.97000 L

C -1 34.48000 29.60000 13.25000 L

O -1 35.23000 30.62000 13.35000 L

N -1 34.48000 28.85000 12.14000 L

H -1 33.79000 28.14000 12.03000 L

C -1 35.50000 29.07000 11.08000 L

H -1 36.26000 29.54000 11.52000 L

C -1 35.88000 27.66000 10.48000 L

H -1 36.60000 27.76000 9.79000 L

H -1 35.07000 27.24000 10.05000 L

C -1 36.39000 26.76000 11.64000 L

H -1 35.68000 26.61000 12.33000 L

C -1 36.77000 25.33000 11.10000 L

H -1 37.09000 24.76000 11.85000 L

H -1 35.97000 24.90000 10.68000 L

H -1 37.49000 25.42000 10.41000 L

C -1 37.63000 27.53000 12.27000 L

H -1 38.00000 26.99000 13.03000 L

H -1 38.34000 27.65000 11.57000 L

H -1 37.34000 28.42000 12.61000 L

C -1 35.00000 29.97000 9.95000 L

O -1 35.56000 29.99000 8.84000 L

N -1 33.90000 30.68000 10.17000 L

H -1 33.45000 30.65000 11.07000 L

C -1 33.32000 31.51000 9.10000 L

H -1 33.19000 30.85000 8.36000 L

C -1 32.02000 32.12000 9.60000 L

H -1 31.46000 31.39000 9.99000 L

H -1 31.54000 32.52000 8.82000 L

C -1 32.27000 33.24000 10.70000 L

H -1 32.74000 34.02000 10.30000 L

H -1 32.82000 32.87000 11.45000 L

C -1 30.96000 33.73000 11.28000 L

H -1 30.51000 32.99000 11.78000 L

H -1 30.36000 34.05000 10.55000 L

N -1 31.22000 34.87000 12.24000 L

H -1 32.12000 35.30000 12.19000 L

C -1 30.36000 35.34000 13.12000 L

N -1 30.74000 36.37000 13.93000 L

H -1 31.66000 36.75000 13.84000 L

H -1 30.11000 36.74000 14.60000 L

N -1 29.12000 34.82000 13.19000 L

H -1 28.85000 34.08000 12.57000 L

H -1 28.46000 35.18000 13.86000 L

C -1 34.23000 32.61000 8.53000 L

O -1 34.02000 33.02000 7.39000 L

N -1 35.24000 33.09000 9.28000 L

H -1 35.42000 32.70000 10.18000 L

C -1 36.08000 34.15000 8.76000 L

H -1 35.56000 34.58000 8.02000 L

C -1 36.37000 35.16000 9.92000 L

H -1 37.08000 35.81000 9.67000 L

H -1 36.62000 34.69000 10.76000 L

O -1 35.11000 35.87000 10.12000 L

H -1 35.23000 36.54000 10.85000 L

C -1 37.40000 33.60000 8.15000 L

O -1 38.29000 34.37000 7.83000 L

N -1 37.51000 32.29000 7.97000 L

H -1 36.85000 31.67000 8.41000 L

C -1 38.58000 31.72000 7.13000 L

H -1 39.42000 32.03000 7.59000 L

C -1 38.52000 30.16000 7.04000 L

H -1 39.18000 29.85000 6.36000 L

H -1 37.59000 29.89000 6.76000 L

C -1 38.84000 29.45000 8.38000 L

H -1 38.64000 28.47000 8.28000 L

H -1 38.25000 29.83000 9.10000 L

C -1 40.37000 29.65000 8.78000 L

O -1 40.71000 29.50000 9.96000 L

O -1 41.16000 30.07000 7.92000 L

C -1 38.47000 32.26000 5.71000 L

O -1 37.36000 32.38000 5.17000 L

N -1 39.64000 32.59000 5.09000 L

H -1 40.49000 32.59000 5.62000 L

C -1 39.70000 32.93000 3.67000 L

H -1 39.02000 33.65000 3.53000 L

C -1 41.19000 33.38000 3.34000 L

H -1 41.87000 32.78000 3.77000 L

C -1 41.46000 33.45000 1.80000 L

H -1 42.40000 33.74000 1.64000 L

H -1 41.31000 32.55000 1.39000 L

H -1 40.84000 34.12000 1.38000 L

O -1 41.29000 34.73000 3.87000 L

H -1 42.21000 35.08000 3.70000 L

C -1 39.32000 31.72000 2.79000 L

O -1 39.77000 30.62000 3.06000 L

N -1 38.60000 31.96000 1.68000 L

H -1 38.25000 32.88000 1.51000 L

C -1 38.33000 30.88000 0.72000 L

H -1 38.43000 30.00000 1.19000 L

H -1 38.99000 30.94000 -0.03000 L

C -1 36.93000 31.00000 0.17000 L

O -1 36.22000 32.03000 0.43000 L

N -1 36.51000 29.99000 -0.59000 L

H -1 37.10000 29.21000 -0.74000 L

C -1 35.17000 30.03000 -1.20000 L

H -1 35.18000 29.58000 -2.09000 L

H -1 34.87000 30.98000 -1.31000 L

C -1 34.28000 29.30000 -0.24000 L

O -1 34.63000 28.18000 0.22000 L

N -1 33.11000 29.89000 -0.02000 L

H -1 32.93000 30.79000 -0.42000 L

C -1 32.06000 29.25000 0.80000 L

H -1 32.49000 28.57000 1.40000 L

C -1 31.32000 30.42000 1.57000 L

H -1 31.01000 31.10000 0.90000 L

H -1 31.95000 30.84000 2.21000 L

C -1 30.08000 29.93000 2.36000 L

H -1 29.59000 29.21000 1.87000 L

C -1 30.52000 29.42000 3.68000 L

H -1 29.72000 29.10000 4.20000 L

H -1 31.15000 28.66000 3.55000 L

H -1 30.98000 30.15000 4.19000 L

C -1 29.29000 31.25000 2.71000 L

H -1 28.47000 31.02000 3.22000 L

H -1 29.87000 31.85000 3.25000 L

H -1 29.03000 31.71000 1.86000 L

C -1 31.09000 28.52000 -0.08000 L

O -1 30.66000 29.08000 -1.12000 L

N -1 30.75000 27.26000 0.29000 L

H -1 31.15000 26.88000 1.12000 L

C -1 29.85000 26.46000 -0.46000 L

H -1 29.51000 27.06000 -1.19000 L

C -1 30.64000 25.25000 -1.01000 L

H -1 31.43000 25.56000 -1.54000 L

H -1 30.05000 24.68000 -1.58000 L

O -1 31.13000 24.44000 0.09000 L

H -1 31.64000 23.66000 -0.28000 L

C -1 28.70000 25.92000 0.48000 L

O -1 28.74000 26.12000 1.70000 L

N -1 27.74000 25.17000 -0.06000 L

H -1 27.71000 24.97000 -1.04000 L

C -1 26.71000 24.65000 0.89000 L

H -1 27.13000 24.56000 1.79000 L

H -1 26.41000 23.76000 0.57000 L

C -1 25.51000 25.56000 1.00000 L

O -1 25.25000 26.39000 0.15000 L

N -1 24.68000 25.29000 2.00000 L

H -1 24.95000 24.62000 2.70000 L

C -1 23.37000 25.96000 2.09000 L

H -1 22.93000 25.78000 1.21000 L

C -1 22.61000 25.38000 3.37000 L

H -1 23.10000 25.68000 4.19000 L

H -1 22.62000 24.38000 3.31000 L

C -1 21.06000 25.91000 3.43000 L

H -1 20.55000 25.49000 2.68000 L

H -1 21.05000 26.90000 3.32000 L

C -1 20.30000 25.56000 4.79000 L

H -1 19.43000 26.05000 4.78000 L

H -1 20.86000 25.89000 5.55000 L

C -1 20.03000 23.94000 4.98000 L

H -1 19.24000 23.80000 5.58000 L

H -1 20.84000 23.51000 5.38000 L

N -1 19.71000 23.15000 3.70000 L

H -1 19.57000 22.19000 3.93000 L

H -1 18.89000 23.52000 3.27000 L

H -1 20.48000 23.22000 3.06000 L

C -1 23.41000 27.46000 2.05000 L

O -1 22.56000 28.07000 1.39000 L

N -1 24.39000 28.12000 2.72000 L

C -1 25.55000 27.62000 3.52000 L

H -1 26.34000 27.46000 2.93000 L

H -1 25.31000 26.78000 4.00000 L

C -1 25.78000 28.80000 4.49000 L

H -1 26.71000 28.80000 4.86000 L

H -1 25.12000 28.79000 5.25000 L

C -1 25.53000 30.03000 3.49000 L

H -1 26.33000 30.20000 2.91000 L

H -1 25.29000 30.87000 3.98000 L

C -1 24.33000 29.56000 2.62000 L

H -1 23.40000 29.81000 2.91000 L

C -1 24.55000 30.14000 1.21000 L

O -1 24.28000 31.32000 1.01000 L

N -1 25.11000 29.35000 0.29000 L

H -1 25.39000 28.43000 0.56000 L

C -1 25.32000 29.77000 -1.10000 L

H -1 25.53000 30.75000 -1.06000 L

C -1 26.50000 28.91000 -1.70000 L

H -1 26.32000 27.95000 -1.49000 L

H -1 27.35000 29.19000 -1.27000 L

C -1 26.72000 29.00000 -3.21000 L

H -1 25.88000 28.70000 -3.68000 L

C -1 27.10000 30.47000 -3.59000 L

H -1 27.24000 30.53000 -4.58000 L

H -1 26.35000 31.09000 -3.32000 L

H -1 27.93000 30.73000 -3.11000 L

C -1 27.89000 28.10000 -3.59000 L

H -1 28.04000 28.15000 -4.58000 L

H -1 28.71000 28.41000 -3.11000 L

H -1 27.69000 27.16000 -3.34000 L

C -1 24.04000 29.46000 -1.94000 L

O -1 23.84000 29.97000 -3.02000 L

N -1 23.16000 28.64000 -1.44000 L

H -1 23.18000 28.40000 -0.46000 L

C -1 22.16000 28.07000 -2.29000 L

H -1 22.73000 27.53000 -2.92000 L

C -1 21.14000 27.20000 -1.46000 L

H -1 20.70000 27.78000 -0.77000 L

H -1 21.63000 26.46000 -1.01000 L

C -1 20.05000 26.61000 -2.38000 L

H -1 20.49000 26.03000 -3.07000 L

H -1 19.57000 27.35000 -2.84000 L

C -1 19.04000 25.76000 -1.56000 L

H -1 19.51000 25.06000 -1.03000 L

H -1 18.36000 25.33000 -2.16000 L

N -1 18.35000 26.72000 -0.65000 L

H -1 18.26000 27.68000 -0.93000 L

C -1 17.85000 26.36000 0.53000 L

N -1 17.35000 27.30000 1.40000 L

H -1 17.36000 28.27000 1.15000 L

H -1 16.98000 27.02000 2.28000 L

N -1 17.79000 25.02000 0.81000 L

H -1 18.10000 24.35000 0.14000 L

H -1 17.42000 24.71000 1.69000 L

C -1 21.35000 29.17000 -3.08000 L

O -1 21.25000 29.11000 -4.33000 L

N -1 20.70000 30.11000 -2.40000 L

H -1 20.83000 30.21000 -1.41000 L

C -1 19.78000 30.99000 -3.16000 L

H -1 19.36000 30.41000 -3.85000 L

C -1 18.72000 31.63000 -2.19000 L

H -1 18.27000 32.39000 -2.66000 L

H -1 19.19000 31.96000 -1.37000 L

C -1 17.65000 30.56000 -1.77000 L

O -1 17.87000 29.37000 -2.09000 L

O -1 16.63000 30.91000 -1.17000 L

C -1 20.52000 32.07000 -3.94000 L

O -1 20.06000 32.49000 -4.95000 L

N -1 21.68000 32.48000 -3.46000 L

H -1 21.98000 32.19000 -2.55000 L

C -1 22.51000 33.36000 -4.24000 L

H -1 21.98000 34.20000 -4.37000 L

C -1 23.84000 33.57000 -3.47000 L

H -1 24.28000 32.68000 -3.34000 L

H -1 23.62000 33.97000 -2.57000 L

C -1 24.81000 34.54000 -4.24000 L

H -1 24.92000 34.20000 -5.18000 L

C -1 24.24000 35.99000 -4.34000 L

H -1 24.88000 36.57000 -4.84000 L

H -1 23.36000 35.97000 -4.82000 L

H -1 24.11000 36.35000 -3.41000 L

C -1 26.15000 34.62000 -3.32000 L

H -1 26.82000 35.22000 -3.75000 L

H -1 25.91000 34.99000 -2.42000 L

H -1 26.54000 33.71000 -3.21000 L

C -1 22.82000 32.70000 -5.58000 L

O -1 22.78000 33.33000 -6.63000 L

N -1 23.16000 31.41000 -5.57000 L

H -1 23.26000 30.93000 -4.70000 L

C -1 23.39000 30.66000 -6.86000 L

H -1 24.08000 31.17000 -7.38000 L

C -1 23.82000 29.22000 -6.53000 L

H -1 23.22000 28.80000 -5.85000 L

H -1 24.77000 29.18000 -6.21000 L

O -1 23.73000 28.48000 -7.71000 L

H -1 24.01000 27.53000 -7.52000 L

C -1 22.12000 30.56000 -7.68000 L

O -1 22.10000 30.84000 -8.89000 L

N -1 21.02000 30.20000 -7.03000 L

H -1 21.05000 29.99000 -6.06000 L

C -1 19.75000 30.13000 -7.78000 L

H -1 19.94000 29.43000 -8.47000 L

C -1 18.56000 29.74000 -6.88000 L

H -1 18.46000 30.38000 -6.12000 L

C -1 17.21000 29.59000 -7.73000 L

H -1 16.46000 29.33000 -7.13000 L

H -1 17.00000 30.46000 -8.18000 L

H -1 17.33000 28.87000 -8.43000 L

O -1 18.87000 28.48000 -6.32000 L

H -1 18.12000 28.18000 -5.73000 L

C -1 19.40000 31.42000 -8.43000 L

O -1 18.95000 31.45000 -9.63000 L

N -1 19.63000 32.54000 -7.72000 L

H -1 20.04000 32.49000 -6.81000 L

C -1 19.24000 33.84000 -8.30000 L

H -1 18.30000 33.70000 -8.61000 L

C -1 19.34000 35.01000 -7.24000 L

H -1 19.30000 35.88000 -7.73000 L

H -1 20.21000 34.94000 -6.76000 L

C -1 18.18000 35.00000 -6.18000 L

H -1 18.33000 35.73000 -5.52000 L

H -1 18.17000 34.12000 -5.71000 L

C -1 16.80000 35.21000 -6.85000 L

O -1 16.20000 34.28000 -7.35000 L

N -1 16.29000 36.45000 -6.80000 L

H -1 16.80000 37.19000 -6.35000 L

H -1 15.40000 36.65000 -7.22000 L

C -1 20.17000 34.15000 -9.48000 L

O -1 19.80000 34.86000 -10.43000 L

N -1 21.46000 33.80000 -9.36000 L

H -1 21.79000 33.41000 -8.50000 L

C -1 22.40000 34.00000 -10.50000 L

H -1 22.31000 34.97000 -10.73000 L

C -1 23.87000 33.64000 -10.08000 L

H -1 23.99000 32.66000 -9.92000 L

C -1 24.87000 34.13000 -11.15000 L

H -1 25.80000 33.90000 -10.87000 L

H -1 24.66000 33.69000 -12.02000 L

H -1 24.78000 35.12000 -11.25000 L

O -1 24.18000 34.34000 -8.86000 L

H -1 25.12000 34.12000 -8.58000 L

C -1 21.98000 33.19000 -11.76000 L

O -1 22.05000 33.64000 -12.95000 L

N -1 21.60000 31.98000 -11.53000 L

H -1 21.67000 31.58000 -10.62000 L

C -1 21.03000 31.18000 -12.69000 L

H -1 21.74000 31.14000 -13.39000 L

C -1 20.56000 29.77000 -12.17000 L

H -1 20.00000 29.88000 -11.35000 L

C -1 19.68000 28.97000 -13.27000 L

H -1 19.41000 28.08000 -12.89000 L

H -1 18.86000 29.50000 -13.49000 L

H -1 20.22000 28.83000 -14.09000 L

C -1 21.81000 28.98000 -11.77000 L

H -1 22.46000 29.61000 -11.35000 L

H -1 22.22000 28.60000 -12.60000 L

C -1 21.45000 27.80000 -10.75000 L

H -1 22.28000 27.31000 -10.51000 L

H -1 21.04000 28.19000 -9.93000 L

H -1 20.80000 27.18000 -11.19000 L

C -1 19.85000 31.88000 -13.31000 L

O -1 19.73000 32.00000 -14.57000 L

N -1 18.95000 32.30000 -12.45000 L

H -1 19.09000 32.16000 -11.47000 L

C -1 17.74000 32.99000 -12.91000 L

H -1 17.24000 32.37000 -13.51000 L

C -1 16.90000 33.34000 -11.65000 L

H -1 17.41000 34.00000 -11.10000 L

H -1 16.76000 32.51000 -11.12000 L

C -1 15.51000 33.96000 -12.03000 L

H -1 15.00000 33.30000 -12.59000 L

H -1 15.66000 34.80000 -12.55000 L

C -1 14.66000 34.30000 -10.69000 L

H -1 13.69000 34.43000 -10.92000 L

H -1 15.01000 35.14000 -10.27000 L

N -1 14.69000 33.26000 -9.62000 L

H -1 15.26000 33.44000 -8.82000 L

C -1 14.03000 32.11000 -9.64000 L

N -1 13.27000 31.72000 -10.72000 L

H -1 13.21000 32.32000 -11.52000 L

H -1 12.79000 30.85000 -10.70000 L

N -1 14.05000 31.29000 -8.62000 L

H -1 14.58000 31.53000 -7.81000 L

H -1 13.55000 30.43000 -8.66000 L

C -1 18.07000 34.22000 -13.79000 L

O -1 17.54000 34.41000 -14.89000 L

N -1 18.98000 35.08000 -13.31000 L

H -1 19.36000 34.94000 -12.39000 L

C -1 19.41000 36.22000 -14.10000 L

H -1 18.58000 36.72000 -14.33000 L

C -1 20.42000 37.04000 -13.25000 L

H -1 21.23000 36.48000 -13.08000 L

H -1 19.98000 37.27000 -12.37000 L

C -1 20.86000 38.34000 -13.90000 L

H -1 20.07000 38.75000 -14.36000 L

H -1 21.57000 38.13000 -14.57000 L

C -1 21.44000 39.36000 -12.84000 L

O -1 21.60000 38.96000 -11.60000 L

O -1 21.64000 40.57000 -13.22000 L

C -1 20.10000 35.77000 -15.40000 L

O -1 19.84000 36.38000 -16.46000 L

N -1 21.05000 34.79000 -15.35000 L

H -1 21.25000 34.31000 -14.49000 L

C -1 21.80000 34.45000 -16.61000 L

H -1 22.16000 35.32000 -16.95000 L

C -1 22.98000 33.47000 -16.32000 L

H -1 23.39000 33.20000 -17.19000 L

H -1 22.61000 32.65000 -15.87000 L

C -1 24.10000 34.04000 -15.42000 L

H -1 24.85000 33.38000 -15.50000 L

H -1 23.72000 34.01000 -14.50000 L

S -1 24.62000 35.75000 -15.90000 L

C -1 25.28000 35.29000 -17.56000 L

H -1 25.63000 36.11000 -18.02000 L

H -1 26.02000 34.62000 -17.45000 L

H -1 24.54000 34.89000 -18.11000 L

C -1 20.83000 33.83000 -17.65000 L

O -1 20.99000 34.02000 -18.87000 L

N -1 19.84000 33.07000 -17.17000 L

H -1 19.75000 32.95000 -16.18000 L

C -1 18.90000 32.42000 -18.05000 L

H -1 19.40000 31.78000 -18.63000 L

C -1 17.90000 31.68000 -17.23000 L

H -1 17.39000 32.35000 -16.70000 L

H -1 18.41000 31.08000 -16.61000 L

C -1 16.92000 30.82000 -18.06000 L

C -1 17.30000 29.58000 -18.63000 L

H -1 18.23000 29.23000 -18.51000 L

C -1 16.31000 28.82000 -19.40000 L

H -1 16.55000 27.93000 -19.79000 L

C -1 15.03000 29.36000 -19.59000 L

O -1 14.07000 28.70000 -20.27000 L

H -1 13.24000 29.26000 -20.29000 L

C -1 14.67000 30.56000 -19.09000 L

H -1 13.76000 30.95000 -19.28000 L

C -1 15.62000 31.26000 -18.27000 L

H -1 15.34000 32.12000 -17.84000 L

C -1 18.12000 33.57000 -18.81000 L

O -1 17.92000 33.50000 -20.04000 L

N -1 17.66000 34.56000 -18.06000 L

H -1 17.81000 34.54000 -17.07000 L

C -1 16.91000 35.70000 -18.67000 L

H -1 16.21000 35.26000 -19.23000 L

C -1 16.25000 36.69000 -17.53000 L

H -1 15.76000 37.44000 -17.98000 L

H -1 15.61000 36.16000 -16.97000 L

H -1 16.98000 37.06000 -16.95000 L

C -1 17.83000 36.50000 -19.60000 L

O -1 17.45000 36.82000 -20.73000 L

N -1 19.05000 36.80000 -19.21000 L

H -1 19.39000 36.49000 -18.32000 L

C -1 19.89000 37.59000 -20.08000 L

H -1 19.32000 38.39000 -20.25000 L

C -1 21.22000 37.90000 -19.41000 L

H -1 21.85000 38.27000 -20.09000 L

H -1 21.60000 37.05000 -19.03000 L

C -1 21.08000 38.92000 -18.27000 L

H -1 20.28000 38.65000 -17.73000 L

C -1 22.31000 38.92000 -17.34000 L

H -1 22.18000 39.60000 -16.61000 L

H -1 22.42000 38.02000 -16.93000 L

H -1 23.13000 39.16000 -17.87000 L

C -1 20.96000 40.37000 -18.89000 L

H -1 20.87000 41.05000 -18.15000 L

H -1 21.78000 40.58000 -19.43000 L

H -1 20.15000 40.42000 -19.48000 L

C -1 20.27000 36.81000 -21.36000 L

O -1 20.69000 37.43000 -22.33000 L

N -1 20.22000 35.48000 -21.34000 L

H -1 19.97000 34.99000 -20.50000 L

C -1 20.56000 34.73000 -22.58000 L

H -1 20.98000 35.40000 -23.19000 L

C -1 21.51000 33.49000 -22.21000 L

H -1 21.64000 32.85000 -22.98000 L

C -1 22.87000 33.97000 -21.73000 L

H -1 23.44000 33.17000 -21.50000 L

H -1 23.31000 34.50000 -22.45000 L

H -1 22.76000 34.53000 -20.91000 L

O -1 20.90000 32.68000 -21.22000 L

H -1 21.50000 31.91000 -21.00000 L

C -1 19.29000 34.19000 -23.25000 L

O -1 19.32000 33.29000 -24.12000 L

N -1 18.16000 34.70000 -22.81000 L

H -1 18.17000 35.35000 -22.05000 L

C -1 16.88000 34.33000 -23.42000 L

H -1 16.23000 34.78000 -22.81000 L

C -1 16.78000 34.80000 -24.88000 L

H -1 15.95000 34.41000 -25.28000 L

H -1 17.58000 34.47000 -25.38000 L

C -1 16.72000 36.33000 -25.01000 L

H -1 16.01000 36.70000 -24.41000 L

H -1 16.52000 36.59000 -25.96000 L

C -1 17.96000 36.99000 -24.64000 L

O -1 19.06000 36.64000 -25.14000 L

N -1 17.86000 37.98000 -23.77000 L

H -1 16.96000 38.23000 -23.41000 L

H -1 18.67000 38.47000 -23.47000 L

C -1 16.58000 32.90000 -23.37000 L

O -1 15.81000 32.40000 -24.22000 L

N -1 17.05000 32.18000 -22.34000 L

H -1 17.58000 32.61000 -21.61000 L

C -1 16.77000 30.74000 -22.33000 L

H -1 15.78000 30.62000 -22.42000 L

H -1 17.06000 30.39000 -21.43000 L

C -1 17.46000 29.93000 -23.41000 L

O -1 17.21000 28.75000 -23.52000 L

N -1 18.34000 30.51000 -24.20000 L

H -1 18.62000 31.46000 -24.03000 L

C -1 18.91000 29.76000 -25.31000 L

H -1 18.22000 29.06000 -25.48000 L

C -1 19.12000 30.67000 -26.50000 L

H -1 19.58000 30.16000 -27.22000 L

H -1 19.69000 31.44000 -26.21000 L

C -1 17.73000 31.20000 -27.00000 L

H -1 17.20000 31.55000 -26.23000 L

H -1 17.23000 30.46000 -27.45000 L

C -1 17.97000 32.33000 -28.02000 L

H -1 18.41000 31.97000 -28.84000 L

H -1 18.53000 33.05000 -27.62000 L

N -1 16.66000 32.89000 -28.39000 L

H -1 15.86000 32.29000 -28.31000 L

C -1 16.45000 34.14000 -28.81000 L

N -1 17.47000 34.97000 -28.96000 L

H -1 18.41000 34.67000 -28.75000 L

H -1 17.31000 35.91000 -29.28000 L

N -1 15.22000 34.55000 -29.07000 L

H -1 14.45000 33.92000 -28.94000 L

H -1 15.06000 35.48000 -29.39000 L

C -1 20.28000 29.13000 -24.99000 L
[truncated: 33,764 more chars]
